# Blood pressure thresholds in pregnancy for identifying maternal and infant risk: a secondary analysis of Community-Level Interventions for Pre-eclampsia (CLIP) trial data

**DOI:** 10.1016/S2214-109X(21)00219-9

**Published:** 2021-07-05

**Authors:** Jeffrey N Bone, Laura A Magee, Joel Singer, Hannah Nathan, Rahat N Qureshi, Charfudin Sacoor, Esperança Sevene, Andrew Shennan, Mrutyunjaya B Bellad, Shivaprasad S Goudar, Ashalata A Mallapur, Khátia Munguambe, Marianne Vidler, Zulfiqar A Bhutta, Peter von Dadelszen, CLIP study group, Mai-Lei Woo Kinshella, Hubert Wong, Faustino Vilanculo, Anifa Vala, Ugochi V Ukah, Domena K Tu, Lehana Thabane, Corsino Tchavana, Jim Thornton, John O Sotunsa, Sana Sheikh, Sumedha Sharma, Nadine Schuurman, Diane Sawchuck, Amit P Revankar, Farrukh Raza, Umesh Y Ramdurg, Rosa Pires, Beth A Payne, Vivalde Nobela, Cláudio Nkumbula, Ariel Nhancolo, Zefanias Nhamirre, Geetanjali I Mungarwadi, Dulce Mulungo, Sibone Mocumbi, Craig Mitton, Mario Merialdi, Javed Memon, Analisa Matavele, Sphoorthi S Mastiholi, Ernesto Mandlate, Sónia Maculuve, Salésio Macuacua, Eusébio Macete, Marta Macamo, Mansun Lui, Jing Li, Gwyneth Lewis, Simon Lewin, Tang Lee, Ana Langer, Uday S Kudachi, Bhalachandra S Kodkany, Marian Knight, Gudadayya S Kengapur, Avinash J Kavi, Geetanjali M Katageri, Chirag Kariya, Chandrappa C Karadiguddi, Namdev A Kamble, Anjali M Joshi, Eileen Hutton, Amjad Hussain, Zahra Hoodbhoy, Narayan V Honnungar, William Grobman, Emília Gonçálves, Tabassum Firoz, Veronique Fillipi, Paulo Filimone, Susheela M Engelbrecht, Dustin T Dunsmuir, Guy Dumont, Sharla K Drebit, France Donnay, Shafik Dharamsi, Vaibhav B Dhamanekar, Richard Derman, Brian Darlow, Silvestre Cutana, Keval S Chougala, Rogério Chiaú, Umesh S Charantimath, Romano Nkumbwa Byaruhanga, Helena Boene, Ana Ilda Biz, Cassimo Bique, Ana Pilar Betrán, Shashidhar G Bannale, Orvalho Augusto, J Mark Ansermino, Felizarda Amose, Imran Ahmed, Olalekan O Adetoro

**Affiliations:** a Department of Obstetrics and Gynaecology and BC Children's Hospital Research Institute, University of British Columbia, Vancouver, BC, Canada; b Centre for Health Evaluation and Outcome Sciences, Providence Health Care Research Institute, University of British Columbia, Vancouver, BC, Canada; c Department of Women and Children's Health, School of Life Course Sciences, Faculty of Life Sciences and Medicine, King's College London, London, UK; d Centre of Excellence, Division of Woman and Child Health, Aga Khan University, Karachi, Pakistan; e Centro de Investigação em Saúde de Manhiça, Manhiça, Mozambique; f Department of Physiological Sciences, Clinical Pharmacology, Faculdade de Medicina, Universidade Eduardo Mondlane, Maputo, Mozambique; g KLE Academy of Higher Education and Research's J N Medical College, Belagavi, Karnataka, India; h S Nijalingappa Medical College, Hanagal Shree Kumareshwar Hospital and Research Centre, Bagalkote, Karnataka, India; i Centre for Global Child Health, Hospital for Sick Children, Toronto, ON, Canada

## Abstract

**Background:**

Blood pressure measurement is a marker of antenatal care quality. In well resourced settings, lower blood pressure cutoffs for hypertension are associated with adverse pregnancy outcomes. We aimed to study the associations between blood pressure thresholds and adverse outcomes and the diagnostic test properties of these blood pressure cutoffs in low-resource settings.

**Methods:**

We did a secondary analysis of data from 22 intervention clusters in the Community-Level Interventions for Pre-eclampsia (CLIP) cluster randomised trials (NCT01911494) in India (n=6), Mozambique (n=6), and Pakistan (n=10). We included pregnant women aged 15–49 years (12–49 years in Mozambique), identified in their community by trained community health workers, who had data on blood pressure measurements and outcomes. The trial was unmasked. Maximum blood pressure was categorised as: normal blood pressure (systolic blood pressure [sBP] <120 mm Hg and diastolic blood pressure [dBP] <80 mm Hg), elevated blood pressure (sBP 120–129 mm Hg and dBP <80 mm Hg), stage 1 hypertension (sBP 130–139 mm Hg or dBP 80–89 mm Hg, or both), non-severe stage 2 hypertension (sBP 140–159 mm Hg or dBP 90–109 mm Hg, or both), or severe stage 2 hypertension (sBP ≥160 mm Hg or dBP ≥110 mm Hg, or both). We classified women according to the maximum blood pressure category reached across all visits for the primary analyses. The primary outcome was a maternal, fetal, or neonatal mortality or morbidity composite. We estimated dose-response relationships between blood pressure category and adverse outcomes, as well as diagnostic test properties.

**Findings:**

Between Nov 1, 2014, and Feb 28, 2017, 21 069 women (6067 in India, 4163 in Mozambique, and 10 839 in Pakistan) contributed 103 679 blood pressure measurements across the three CLIP trials. Only women with non-severe or severe stage 2 hypertension, as discrete diagnostic categories, experienced more adverse outcomes than women with normal blood pressure (risk ratios 1·29–5·88). Using blood pressure categories as diagnostic thresholds (women with blood pressure within the category or any higher category *vs* those with blood pressure in any lower category), dose-response relationships were observed between increasing thresholds and adverse outcomes, but likelihood ratios were informative only for severe stage 2 hypertension and maternal CNS events (likelihood ratio 6·36 [95% CI 3·65–11·07]) and perinatal death (5·07 [3·64–7·07]), particularly stillbirth (8·53 [5·63–12·92]).

**Interpretation:**

In low-resource settings, neither elevated blood pressure nor stage 1 hypertension were associated with maternal, fetal, or neonatal mortality or morbidity adverse composite outcomes. Only the threshold for severe stage 2 hypertension met diagnostic test performance standards. Current diagnostic thresholds for hypertension in pregnancy should be retained.

**Funding:**

University of British Columbia, the Bill & Melinda Gates Foundation.

## Introduction

Hypertension in pregnancy has traditionally been defined as a systolic blood pressure (sBP) of at least 140 mm Hg or a diastolic blood pressure (dBP) of at least 90 mm Hg, or both.^1^ Hypertension defined in this way identifies pregnant women at increased risk of pre-eclampsia and other maternal and fetal or neonatal complications, including death, and these women are recommended to receive enhanced antenatal care and monitoring worldwide.

In 2017, the American College of Cardiology and American Heart Association recommended lowering the blood pressure thresholds for diagnosing hypertension outside of pregnancy, classified as: elevated blood pressure (or elevated sBP; defined as sBP 120–129 mm Hg and dBP <80 mm Hg), stage 1 hypertension (sBP 130–139 mm Hg or dBP 80–89 mm Hg, or both), and stage 2 hypertension (sBP ≥140 mm Hg or dBP ≥90 mm Hg, or both).^2^ Although the American College of Obstetricians and Gynecologists and WHO have retained a definition of blood pressure greater than or equal to 140/90 mm Hg for hypertension in pregnancy, several studies have reported a dose-response relationship between increasing blood pressure and adverse pregnancy outcomes, across gestational ages;^3^, ^4^ these findings provide potential support for the redefinition of hypertension in pregnancy. However, despite use of blood pressure as an essential screening test in pregnancy, no studies have yet reported the diagnostic test properties (such as sensitivity and specificity) of various blood pressure thresholds for hypertension diagnosis.

**Research in context**

**Evidence before this study**

We searched MEDLINE, PubMed, Embase, CINAHL, CNTRAL, LILACS, Web of Science, and Google Scholar databases, including reference lists of eligible studies, for studies published in English between Jan 1, 2017, and Dec 31, 2020, using the search terms “human” AND (“hypertension” OR “hypertensive disorders of pregnancy” OR “pregnancy-induced hypertension” OR “preeclampsia” OR “pregnancy toxemias” OR “gestational hypertension”) AND (“American College of Cardiology” OR “American Heart Association”) AND (“stage 1 hypertension” OR “prehypertension”) AND (“Pregnancy[mh]” OR “Pregnan*” OR “Gestation*” OR “pregnant women[mh]” OR “Pregnancy Complications[mh]” OR “Postpartum Period”[Mesh] OR “Puerperium” OR “postpartum” OR “Peripartum Period”[Mesh] OR “Peripartum*” OR “Perinatal Care[mh]” OR “perinatal”).

In 2017, the American College of Cardiology and American Heart Association recommended lowering blood pressure thresholds for diagnosing hypertension outside of pregnancy. Several studies have examined the risk of adverse pregnancy outcomes associated with these lower blood pressure values in pregnancy, compared with the established cutoff of 140/90 mm Hg or greater.

**Added value of this study**

Nearly all evidence for using the American College of Cardiology and American Heart Association thresholds to diagnose hypertension in pregnancy are from high-income settings and rely on retrospective, routinely collected clinical data. Furthermore, these studies have focused exclusively on associative measures (such as risk ratios) between blood pressure thresholds and adverse outcomes. By contrast, this study provides prospective blood pressure data from more than 20 000 pregnant women, in three low-income and middle-income countries (LMICs), using standardised measurement technique and a validated device, and examines not only associations between blood pressure thresholds and adverse outcomes, but also the diagnostic test properties of those thresholds. Associations were dependent on blood pressure greater than or equal to 160/110 mm Hg.

**Implications of all the available evidence**

Our findings suggest that there is an association between the American College of Cardiology and American Heart Association blood pressure thresholds and adverse pregnancy outcomes in LMIC settings, but there is no antenatal blood pressure threshold that is sensitive with regards to the adverse maternal, fetal, or neonatal outcomes studied, including data-driven cutoffs. However, severe stage 2 hypertension (blood pressure ≥160/110 mm Hg) is associated with a substantially increased risk of adverse maternal CNS outcomes and fetal or neonatal death, particularly stillbirth, and should be treated as per international guidance. Antenatal care must aim to provide more than accurate blood pressure measurement to achieve the Countdown 2030 goals.

We aimed to analyse the relationship between blood pressure thresholds to define hypertension in pregnancy and adverse maternal, fetal, or neonatal outcomes, as well as the diagnostic test properties of these cutoffs, in low-resource settings.

## Methods

### Study design and participants

We did a secondary analysis of data from 22 intervention clusters in the Community-Level Interventions for Pre-eclampsia (CLIP) cluster randomised trials (NCT01911494) in India (n=6), Mozambique (n=6), and Pakistan (n=10).^5^, ^6^, ^7^, ^8^ The unit of randomisation (cluster) was the local administrative unit. We included pregnant women aged 15–49 years (12–49 years in Mozambique), identified in their community by trained community health workers, who had data on blood pressure measurements and outcomes. All women provided written informed consent to participate. The trial was unmasked given the nature of the intervention, aimed at addressing the so-called three delays in triage, transport, and treatment related to mortality risk, particularly associated with pre-eclampsia.^9^

Ethics approvals were granted by the University of British Columbia, Canada (H12-03497) and relevant in-country research ethics boards (Aga Khan University, Pakistan, 2590-Obs-ERC-13; KLE University, India, MDC/IECHSR/2011-12/A-4, ICMR 5/7/859/12-RHN; Centro de Investigação em Saúde de Manhiça, Mozambique, CIBS-CISM/038/14; and Mozambique National Bioethic Committee, 219/CNBS/14).

### Procedures

First, community engagement addressed barriers and facilitators to accessing care. Second, existing cadres of community health workers were trained to task-share pregnancy hypertension-oriented care at CLIP contacts in women's homes, using the CLIP Pre-eclampsia Integrated Estimate of Risk Score (PIERS) On-the-Move (POM) digital health app for risk stratification.^10^ Community health workers responded to emergency conditions (if applicable); measured women's blood pressure and did dipstick urinalysis for proteinuria at the first and any subsequent contact where hypertension was identified; administered oral methyldopa (750 mg) if blood pressure was greater than or equal to 160/110 mm Hg and intramuscular magnesium sulphate (10 g) if severe pre-eclampsia (defined as at least one of: sBP ≥160 mm Hg, mini pre-eclampsia integrated estimate of risk [miniPIERS] probability ≥25%, eclampsia, or stroke) was suspected; and referred women to a comprehensive emergency obstetric care facility if advised by POM. Antenatal POM-guided visits (including blood pressure measurement) were scheduled monthly from enrolment.

Blood pressure measurement for all women in the intervention clusters (and, therefore, in this analysis) was standardised and taken by trained community health workers, using a semi-automated pregnancy-validated and pre-eclampsia-validated oscillometric device (Microlife 3AS1-2; Microlife, Taipei, Taiwan).^11^ Women were instructed to rest for 5 min, then their blood pressure was measured at least twice, with a third measurement taken if the first two readings differed by 10 mmHg or more. All readings were entered into the POM app, with blood pressure for the visit calculated as the mean of the first two readings, or of the second and third if three readings were taken. All readings were stored electronically in REDCap databases.

Trained surveillance teams did regular household surveys (every 3–6 months) in Mozambique and Pakistan; in India, a prospective population-based surveillance system was established. The PRE-eclampsia Eclampsia Monitoring, Prevention, and Treatment (PRE-EMPT) research group, University of British Columbia, Canada, was responsible for overall trial coordination and data management.

### Outcomes

The primary CLIP composite outcome was a composite of maternal, fetal, and neonatal mortality and morbidity, and all outcomes were adjudicated by an in-country team of clinicians. Maternal death and morbidity were assessed during pregnancy or within 42 days after pregnancy; morbidity was defined as one or more life-threatening pregnancy complications (a serious end-organ complication of pre-eclampsia [ie, eclampsia, stroke, coma, antepartum haemorrhage, or disseminated intravascular coagulation], another major maternal complication [ie, obstetric sepsis, or vesicovaginal or rectovaginal fistula], or receipt of a life-saving intervention [ie, cardiopulmonary resuscitation, mechanical ventilation, blood transfusion, interventions for major post-partum haemorrhage, or dialysis]). This analysis also included a maternal CNS composite outcome of one or more of maternal eclampsia, stroke, coma, or mortality. Fetal or neonatal death included stillbirth, early neonatal mortality, or late neonatal mortality (appendix p 4).

In this analysis, we included CLIP trial participants who were from intervention groups and had at least one POM app-guided antenatal contact with blood pressure measurement done by community health workers at a clinically estimated gestation of 44 weeks or less. Analyses including post-partum and perinatal outcomes were restricted to women who had delivered and provided outcome information.

We classified the antenatal blood pressure readings for each woman at each visit, on the basis of the American College of Cardiology and American Heart Association criteria,^2^ as: normal blood pressure (sBP <120 mm Hg and dBP <80 mm Hg), elevated blood pressure (sBP 120–129 mm Hg and dBP <80 mm Hg), stage 1 hypertension (sBP 130–139 mm Hg or dBP 80–89 mm Hg, or both), non-severe stage 2 hypertension (sBP 140–159 mm Hg or dBP 90–109 mm Hg, or both), and severe stage 2 hypertension (sBP ≥160 mm Hg or dBP ≥110 mm Hg, or both). We classified women according to the maximum blood pressure category reached across all visits for the primary analyses. Women who were hypertensive before 20 weeks of gestation were regarded as having chronic hypertension, and those who were hypertensive at 20 weeks of gestation or greater were regarded as having gestational hypertension or pre-eclampsia.^12^

### Statistical analysis

Descriptive statistics were used to summarise baseline maternal characteristics, and maximal blood pressure categories overall and according to gestational age at measurement (<20 weeks *vs* ≥20 weeks).

The possible dose-response relationship between blood pressure category and adverse outcomes was assessed in two ways. First, we treated each category as mutually exclusive and calculated the risk ratio (RR) between normal blood pressure and each category, using generalised estimating equations with a Poisson link function.^13^ Second, we fit analogous models, but treated the lower limit of each category as a blood pressure cutoff for diagnosis of hypertension. For example, for stage 1 hypertension, we compared women with sBP 130 mm Hg or greater or dBP 80 mm Hg or greater (or both), with those who had sBP less than 130 mm Hg and dBP less than 80 mm Hg. All models were adjusted for maternal age, maternal basic level of education (ie, ≥8 years of schooling in India, attainment of at least grade 5 in Mozambique, or ≥5 years of schooling in Pakistan), gestational age at enrolment, and nulliparity. SEs were based on the sandwich estimator to account for clustering.

The diagnostic test properties of blood pressure categories were assessed using sensitivity, specificity, and positive and negative likelihood ratios (LRs). Positive LR was calculated as: sensitivity / (1 – specificity). Negative LR was calculated as: (1 – sensitivity) / specificity. CIs were calculated by standard methods.^14^ For each calculation, women with blood pressure equal to or higher than the given blood pressure threshold were compared with those with blood pressure lower than the threshold. LRs describe the likelihood that a given test result would alter the probability of a diagnosis; positive LR values were interpreted as good if greater than or equal to 5·0 and negative LR values were interpreted as good if less than 0·2.

In a sensitivity analysis to determine if the sensitivity of blood pressure categories for adverse outcomes improved closer to term, we re-classified women according to the maximum blood pressure category reached within predefined gestational age categories (28 weeks to <32 weeks, 32 weeks to <37 weeks, and ≥37 weeks of gestation). Also, we estimated the optimal sBP and dBP cutoffs to maximise sensitivity of blood pressure for fixed false-positive rates of 5%, 10%, and 20%. All analyses were done using R version 4.0.2.^15^

### Role of the funding source

The funder of the study had no role in study design, data collection, data analysis, data interpretation, or writing of the report.

## Results

Between Nov 1, 2014, and Feb 28, 2017, 21 069 women (6067 in India, 4163 in Mozambique, and 10 839 in Pakistan) contributed 103 679 blood pressure measurements across the three CLIP trials (table 1). Most women were aged in their mid-20s, had less than a basic level of education, and were parous. In general, women were enrolled in CLIP late in the first trimester or early in the second trimester, with most enrolling at less than 20 weeks of gestation. About two-thirds of women had normal blood pressure throughout pregnancy. Slightly more than one-quarter of women had abnormal blood pressure classified as elevated blood pressure (2196 [10·4%] of 21 069) or non-severe stage 1 hypertension (3751 [17·8%]). Stage 2 hypertension (non-severe or severe) occurred in 1342 (6·4%) of 21 069 women. Most women delivered at term, but one-quarter delivered preterm. Another quarter suffered a maternal, fetal, or neonatal complication, which were mostly morbidity for the mother (about one in ten women) or death of the fetus (42 per 1000 livebirths) or neonate (41 per 1000 livebirths). Blood pressure was only 1–2 mmHg higher among women with adverse outcomes than in women without adverse outcomes (appendix p 5).

**Table 1:** Baseline characteristics

		**Participants (n=21 069)**
Country		
	India	6067 (28·8%)
	Mozambique	4163 (19·8%)
	Pakistan	10 839 (51·4%)
Maternal age, years		25·0 (22·0–30·0)
Maternal basic level of education*		8409 (39·9%)
Gestational age at enrolment, weeks		17·1 (11·2–24·1)
Enrolment at ≥20 weeks of gestation		7832 (37·2%)
Parous		15 099 (71·7%)
Blood pressure, mm Hg		
	Mean sBP	106·0 (100·4–112·0)
	Mean dBP	66·6 (62·5–71·0)
	Maximum sBP	113·0 (106·0–120·0)
	Maximum dBP	73·0 (67·0–79·0)
Maximal blood pressure category†		
	Normal blood pressure	13 780 (65·4%)
	Elevated blood pressure	2196 (10·4%)
	Stage 1 hypertension	3751 (17·8%)
	Non-severe stage 2 hypertension	1178 (5·6%)
	Severe stage 2 hypertension	164 (0·8%)
Gestational age at delivery, weeks		39·0 (37·0–40·4)
Preterm delivery (<37 weeks of gestation)		4651 (22·1%)
Outcomes‡		
	Primary CLIP composite	4816 (22·9%)
	Maternal mortality	43 (0·2%)
	Maternal morbidity	2006 (9·5%)
	Maternal CNS composite	282 (1·3%)
	Fetal or neonatal death	1657 (7·9%)
	Stillbirth	810 (3·8%)

Data are n (%) or median (IQR) unless otherwise stated. sBP=systolic blood pressure. dBP=diastolic blood pressure. CLIP=Community-Level Interventions for Pre-eclampsia.

* Basic level of education was defined as at least 8 years of schooling in India, at least attainment of grade 5 in Mozambique, or at least 5 years of schooling in Pakistan.

† Blood pressure was categorised as: normal blood pressure (sBP <120 mm Hg and dBP <80 mm Hg), elevated blood pressure (sBP 120–129 mm Hg and dBP <80 mm Hg), stage 1 hypertension (sBP 130–139 mm Hg or dBP 80–89 mm Hg, or both), non-severe stage 2 hypertension (sBP 140–159 mm Hg or dBP 90–109 mm Hg, or both), or severe stage 2 hypertension (sBP ≥160 mm Hg or dBP ≥110 mm Hg, or both).

‡ The primary CLIP composite outcome was a composite of maternal and perinatal mortality and morbidity. Maternal mortality or morbidity were assessed during pregnancy or within 42 days after pregnancy; morbidity was defined as one or more life-threatening pregnancy complications (ie, a serious end-organ complication of pre-eclampsia, another major cause of maternal mortality, or receipt of a life-saving intervention). The maternal CNS composite outcome was one or more of maternal eclampsia, stroke, coma, or mortality. Fetal or neonatal death included stillbirth and early or late neonatal mortality (appendix p 4).

**Figure 1:**
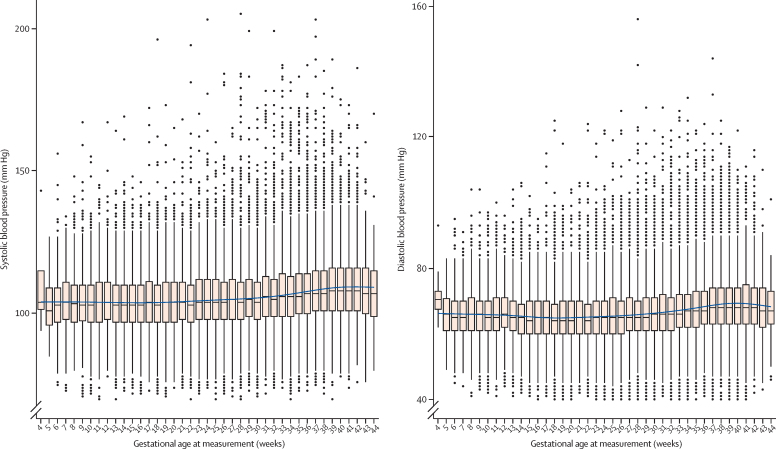
Maximal blood pressure measurements by gestational age Maximal blood pressure values per woman are shown, as a median with IQR for each gestational age week.

**Figure 2:**
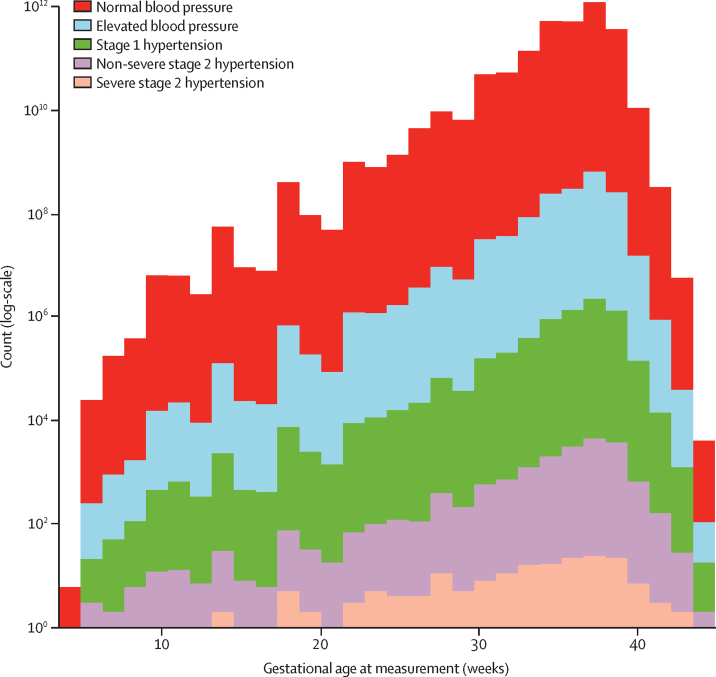
Maximal blood pressure measurements as blood pressure categories by gestational age A log-scale is used for the y-axis (number of women) for clarity. Blood pressure was categorised as: normal blood pressure (sBP <120 mm Hg and dBP <80 mm Hg), elevated blood pressure (sBP 120–129 mm Hg and dBP <80 mm Hg), stage 1 hypertension (sBP 130–139 mm Hg or dBP 80–89 mm Hg, or both), non-severe stage 2 hypertension (sBP 140–159 mm Hg or dBP 90–109 mm Hg, or both), or severe stage 2 hypertension (sBP ≥160 mm Hg or dBP ≥110 mm Hg, or both).

Blood pressure values overall were stable, without a clinically important mid-trimester decrease, until about 30 weeks of gestation, after which both sBP and dBP increased with advancing gestational age (figure 1). Maximal blood pressure values classified as elevated blood pressure, stage 1 hypertension, and stage 2 hypertension (non-severe or severe) were more common with advancing gestational age (figure 2). Blood pressure in most women remained at the same category or decreased after 20 weeks of gestation or longer, regardless of whether blood pressure at less than 20 weeks of gestation was classified as normal blood pressure (5160 [63·0%] of 8194 women), elevated blood pressure (336 [60·8%] of 552), stage 1 hypertension (527 [75·7%] of 696), or non-severe stage 2 hypertension (91 [75·8%] of 120; table 2). If the stage 1 hypertension category cutoffs were used as the new threshold for diagnosing hypertension in pregnancy, an additional 1681 (17·5%) of 9574 women would be diagnosed; if elevated blood pressure category cutoffs were used, an additional 1021 (10·7%) of women would be diagnosed (table 2).

**Table 2:** Maximal blood pressure categorisation according to American College of Cardiology and American Heart Association criteria, by gestational age

	**Normal blood pressure at ≥20 weeks (n=13 346)**	**Elevated blood pressure at ≥20 weeks (n=2024)**	**Stage 1 hypertension at ≥20 weeks (n=3420)**	**Non-severe stage 2 hypertension at ≥20 weeks (n=1095)**	**Severe stage 2 hypertension at ≥20 weeks (n=154)**	**No blood pressure values at ≥20 weeks (n=1030)**
Normal blood pressure at <20 weeks (n=8194)	5160	685*	1035*†	378	48	888
Elevated blood pressure at <20 weeks (n=552)	219*	117*	119*†	49	0	48
Stage 1 hypertension at <20 weeks (n=696)	206*†	87*†	234*†	83	11	75
Non-severe stage 2 hypertension at <20 weeks (n=120)	27	7	36	21	13	16
Severe stage 2 hypertension at <20 weeks (n=12)	2	1	1	3	2	3
No blood pressure values at <20 weeks (n=11 495)	7732	1127	1995	561	80	0

Data are n. Blood pressure was categorised as: normal blood pressure (sBP <120 mm Hg and dBP <80 mm Hg), elevated blood pressure (sBP 120–129 mm Hg and dBP <80 mm Hg), stage 1 hypertension (sBP 130–139 mm Hg or dBP 80–89 mm Hg, or both), non-severe stage 2 hypertension (sBP 140–159 mm Hg or dBP 90–109 mm Hg, or both), or severe stage 2 hypertension (sBP ≥160 mm Hg or dBP ≥110 mm Hg, or both). sBP=systolic blood pressure. dBP=diastolic blood pressure.

* Indicates women who would additionally be diagnosed with hypertension if elevated blood pressure was used as the threshold for diagnosing hypertension in pregnancy.

† Indicates women who would additionally be diagnosed with hypertension if stage 1 hypertension was used as the threshold for diagnosing hypertension in pregnancy.

There was a dose-response relationship between higher blood pressure category and greater RR for adverse outcomes compared with normal blood pressure, for most outcomes with non-severe stage 2 hypertension and for all outcomes with severe stage 2 hypertension, which increased risk by at least two times and up to six times (table 3). When the diagnostic criteria for each blood pressure category were used as a threshold for diagnosis of an abnormal blood pressure, the risk of all adverse outcomes, other than the maternal composite, increased from elevated blood pressure onwards; however, the point estimates for elevated blood pressure, stage 1 hypertension, and non-severe stage 2 hypertension were consistently higher than they were when these blood pressure categories were each compared with normal blood pressure, and RRs were less than 2·00 for all but non-severe stage 2 hypertension and stillbirth.

**Table 3:** Adjusted RRs for blood pressure categories and adverse outcomes

	**Normal blood pressure**	**Elevated blood pressure**	**Stage 1 hypertension**	**Non-severe stage 2 hypertension**	**Severe stage 2 hypertension**
**Comparison with normal blood pressure category**					
CLIP composite	1 (ref)	0·99 (0·94–1·03)	1·08 (1·00–1·16)*†	1·29 (1·13–1·47)†	2·48 (2·06–2·98)†
Maternal composite	1 (ref)	1·02 (0·89–1·17)	1·11 (0·93–1·33)	1·20 (0·90–1·59)	2·40 (1·60–3·59)†
Maternal CNS composite	1 (ref)	0·98 (0·63–1·51)	1·26 (0·91–1·75)	1·46 (1·00–2·13)†	6·05 (3·88–9·46)†
Fetal or neonatal death	1 (ref)	0·93 (0·79–1·09)	1·15 (0·96–1·38)	1·48 (1·10–1·97)†	4·09 (3·02–5·55)†
Stillbirth	1 (ref)	0·98 (0·74–1·29)	1·24 (0·97–1·58)	1·81 (1·30–2·54)†	5·88 (3·95–8·73)†
**Comparison of blood pressure category and all higher categories, with all lower blood pressure categories**‡					
CLIP composite	..	1·12 (1·05–1·19)†	1·17 (1·09–1·26)†	1·41 (1·24–1·60)†	2·48 (2·06–2·98)†
Maternal composite	..	1·13 (0·96–1·33)	1·17 (0·98–1·40)	1·32 (1·05–1·65)†	2·40 (1·60–3·59)†
Maternal CNS composite	..	1·32 (1·08–1·61)†	1·47 (1·13–1·90)†	1·91 (1·41–2·57)†	6·05 (3·88–9·46)†
Fetal or neonatal death	..	1·20 (1·02–1·43)†	1·33 (1·12–1·58)†	1·75 (1·42–2·15)†	4·09 (3·02–5·55)†
Stillbirth	..	1·36 (1·09–1·70)†	1·52 (1·24–1·87)†	2·19 (1·72–2·79)†	5·88 (3·95–8·73)†

Data are RR (95% CI). Blood pressure was categorised as: normal blood pressure (sBP <120 mm Hg and dBP <80 mm Hg), elevated blood pressure (sBP 120–129 mm Hg and dBP <80 mm Hg), stage 1 hypertension (sBP 130–139 mm Hg or dBP 80–89 mm Hg, or both), non-severe stage 2 hypertension (sBP 140–159 mm Hg or dBP 90–109 mm Hg, or both), or severe stage 2 hypertension (sBP ≥160 mm Hg or dBP ≥110 mm Hg, or both). RRs were estimated from modified Poisson and adjusted for maternal age, maternal basic education, gestational age at enrolment, and nulliparity. For outcome definitions, see the appendix (pp 3–4). RR=risk ratio. CLIP=Community-Level Interventions for Pre-eclampsia. sBP=systolic blood pressure. dBP=diastolic blood pressure.

* Unrounded lower limit of 95% CI is 1·002.

† 95% CI does not overlap 1.

‡ For example, for stage 1 hypertension, the comparison is for all women with sBP 130 mm Hg or greater or dBP 80 mm Hg or greater, or both, versus all women with sBP <130 mm Hg and dBP <80 mm Hg; the exception to this approach was for the normal blood pressure category which was compared with all higher blood pressure categories.

The only blood pressure category that was useful as a diagnostic test for the outcomes examined was severe stage 2 hypertension; sensitivity was low, but the positive LR was good for the maternal CNS composite outcome and fetal or neonatal death, particularly stillbirth (table 4). All other blood pressure categories, including non-severe stage 2 hypertension, had low sensitivity (<45%) for all outcomes, and uninformative positive LR and negative LR values.

**Table 4:** Sensitivity, specificity, and LRs for adverse outcomes by American College of Cardiology and American Heart Association blood pressure categories

	Events, n (%)*	**Sensitivity (95% CI)**	**Specificity (95% CI)**	**Positive LR (95% CI)**†	**Negative LR (95% CI)**‡
**CLIP composite**					
Normal blood pressure (n=13 441)	2987 (22·2%)	..	..	..	..
Elevated blood pressure (n=2149)	484 (22·5%)	38·0% (36·6–39·4)	66·3% (65·5–67·0)	1·13 (1·08–1·17)	0·94 (0·91–0·96)
Stage 1 hypertension (n=3679)	937 (25·5%)	27·9% (26·7–29·2)	76·8% (76·1–77·5)	1·20 (1·14–1·27)	0·94 (0·92–0·96)
Non-severe stage 2 hypertension (n=1163)	321 (27·6%)	8·5% (7·7–9·0)	94·2% (93·8–94·6)	1·46 (1·30–1·63)	0·97 (0·96–0·98)
Severe stage 2 hypertension (n=161)	87 (54·0%)	1·8% (1·4–2·2)	99·5% (99·4–99·6)	3·85 (2·83–5·24)	0·99 (0·98–0·99)
**Maternal composite**					
Normal blood pressure (n=13 424)	1253 (9·3%)	..	..	..	..
Elevated blood pressure (n=2146)	212 (9·9%)	38·2% (36·1–40·4)	65·7% (65·0–66·3)	1·11 (1·05–1·18)	0·94 (0·91–0·97)
Stage 1 hypertension (n=3674)	409 (11·1%)	27·8% (25·9–29·8)	76·1% (75·5–76·7)	1·16 (1·08–1·25)	0·95 (0·92–0·98)
Non-severe stage 2 hypertension (n=1163)	120 (10·3%)	7·6% (6·5–8·9)	93·7% (93·3–94·0)	1·21 (1·03–1·42)	0·99 (0·97–1·00)
Severe stage 2 hypertension (n=160)	35 (21·9%)	1·7% (1·2–2·4)	99·3% (99·2–99·4)	2·56 (1·76–3·71)	0·99 (0·98–1·00)
**Maternal CNS composite**					
Normal blood pressure (n=13 413)	161 (1·2%)	..	..	..	..
Elevated blood pressure (n=2145)	29 (1·4%)	42·9% (37·1–48·9)	65·4% (64·7–66·0)	1·24 (1·08–1·42)	0·87 (0·79–0·97)
Stage 1 hypertension (n=3671)	56 (1·5%)	32·6% (27·2–38·4)	75·8% (75·2–76·4)	1·35 (1·14–1·60)	0·89 (0·82–0·96)
Non-severe stage 2 hypertension (n=1163)	23 (2·0%)	12·8% (9·1–17·2)	93·7% (93·3–94·0)	2·01 (1·48–2·74)	0·93 (0·89–0·97)
Severe stage 2 hypertension (n=160)	13 (8·1%)	4·6% (2·5–7·8)	99·3% (99·1–99·4)	6·36 (3·65–11·07)	0·96 (0·94–0·99)
**Fetal or neonatal death**					
Normal blood pressure (n=12 902)	985 (7·6%)	..	..	..	..
Elevated blood pressure (n=2126)	155 (7·3%)	40·6% (38·2–43·0)	65·0% (64·3–65·7)	1·16 (1·09–1·23)	0·91 (0·88–0·95)
Stage 1 hypertension (n=3647)	337 (9·2%)	31·2% (29·0–33·5)	75·8% (75·1–76·4)	1·29 (1·19–1·39)	0·91 (0·88–0·94)
Non-severe stage 2 hypertension (n=1155)	130 (11·3%)	10·9% (9·4–12·5)	93·8% (93·5–94·2)	1·76 (1·51–2·04)	0·95 (0·93–0·97)
Severe stage 2 hypertension (n=159)	50 (31·4%)	3·0% (2·2–4·0)	99·4% (99·3–99·5)	5·07 (3·64–7·07)	0·98 (0·97–0·98)
**Stillbirth**					
Normal blood pressure (n=12 468)	457 (3·7%)	..	..	..	..
Elevated blood pressure (n=2066)	77 (3·7%)	43·6% (40·1–47·1)	65·0% (64·3–65·7)	1·24 (1·15–1·35)	0·87 (0·82–0·92)
Stage 1 hypertension (n=3496)	167 (4·8%)	34·1% (30·8–37·5)	76·2% (75·4–76·9)	1·43 (1·29–1·58)	0·87 (0·82–0·91)
Non-severe stage 2 hypertension (n=1112)	76 (6·8%)	13·5% (11·2–16·0)	94·8% (94·4–95·2)	2·59 (2·14–3·13)	0·91 (0·89–0·94)
Severe stage 2 hypertension (n=151)	33 (21·9%)	4·1% (2·8–5·7)	99·5% (99·4–99·6)	8·53 (5·63–12·92)	0·96 (0·95–0·98)

Diagnostic test properties are calculated for women with blood pressure at each threshold or higher (compared with women with blood pressure below that threshold, based on cumulative rates). For outcome definitions, see the appendix (pp 3–4). Blood pressure was categorised as: normal blood pressure (sBP <120 mmHg and dBP <80 mmHg), elevated blood pressure (sBP 120–129 mmHg and dBP <80 mmHg), stage 1 hypertension (sBP 130–139 mmHg or dBP 80–89 mmHg, or both), non-severe stage 2 hypertension (sBP 140–159 mmHg or dBP 90–109 mmHg, or both), or severe stage 2 hypertension (sBP ≥160 mmHg or dBP ≥110 mmHg, or both). LR=likelihood ratio. CLIP=Community-Level Interventions for Pre-eclampsia. sBP=systolic blood pressure. dBP=diastolic blood pressure.

* Events only include women in the category specified; the denominator is women with complete outcome data.

† A positive LR of 5·0 or greater was considered good.

‡ A negative LR of less than 0·20 was considered good.

In sensitivity analyses restricted to blood pressure measurements in specific gestational age ranges within the third trimester, sensitivity remained low for all blood pressure categories, including the elevated blood pressure category, although sensitivity was slightly higher for measurements from 32 weeks to less than 37 weeks (appendix p 8). Also, sensitivity remained low (<20%) for optimal sBP (123–136 mm Hg) and dBP (81–104 mm Hg) cutoffs, for fixed false-positive rates of 5%, 10%, and 20% (appendix p 6).

## Discussion

In the CLIP trial clusters in low-resource settings, women classified as having elevated blood pressure or stage 1 hypertension did not have an increased risk of adverse maternal, fetal, or neonatal outcomes compared with women with normal blood pressure (<120/80 mm Hg). Risk of adverse outcomes was increased with stage 2 hypertension, particularly with severe stage 2 hypertension, which was associated with a two to six times increased risk compared with normal blood pressure. When blood pressure categories higher than normal were used as diagnostic cutoffs for abnormal blood pressure (how 140/90 mm Hg is currently used), the risk of adverse outcomes was increased; however, this increase was driven by the risks associated with severe stage 2 hypertension. In addition, sensitivities for adverse outcomes were low (<45%), and no category other than severe stage 2 hypertension showed useful diagnostic test properties. Using optimal cutpoint analyses for false-positive rates considered clinically reasonable led to poor sensitivity (<30%) for all outcomes.

If elevated blood pressure was used as a diagnostic cutoff, an additional 28% of women would be labelled as having an abnormal blood pressure; if stage 1 hypertension was used, this would be an additional 11% of women, in addition to women already identified as having hypertension by current criteria (ie, stage 2 hypertension in this study). In our study population, the prevalence of hypertension was previously found to be 14·0% in India, 16·8% in Mozambique, and 11·6% in Pakistan, after inclusion of hypertensive diagnoses from household survey and facility records.^16^ Therefore, an additional 28% or 11% of women with diagnoses of hypertension (depending on the cutoff used) would substantially increase the burden on health systems under stress, and could be justified only by the ability to identify women and babies at risk.

To our knowledge, this is the first study to report on the diagnostic properties of using revised blood pressure thresholds for the diagnosis of hypertension in pregnancy, and the first to report on outcomes by specific blood pressure thresholds for women in under-resourced settings.

Numerous studies have reported that women with antenatal elevated blood pressure or stage 1 hypertension by American College of Cardiology and American Heart Association criteria, as used in our analyses, have an increased risk of adverse pregnancy outcomes;^3^, ^4^, ^17^, ^18^, ^19^ these included pre-eclampsia and other outcomes (eg, hospitalisation) not reported in our study. Many published RRs for adverse maternal and fetal or neonatal outcomes have been higher than in our study. It is possible that the relationship between blood pressure and adverse outcomes might be different in our study setting. Furthermore, we had fewer baseline characteristics with which to adjust our RRs compared with other studies,^4^, ^17^, ^18^, ^19^, ^20^ and we studied unselected pregnant women, not just those who were nulliparous^21^, ^22^ or primarily nulliparous.^23^ Alternatively, the differences in RRs might have been due to methodology. Our data collection was prospective and blood pressure measurement was standardised, using a device validated for pregnancy and pre-eclampsia.^11^ By contrast, most published data have been retrospective and from large urban referral centres with blood pressure measurements from routine clinical care.^4^, ^17^, ^18^, ^19^, ^20^, ^23^ One prospective study restricted blood pressure observations to women at less than 20 weeks of gestation,^21^, ^22^ by contrast to our blood pressure measurements that were done throughout pregnancy, the majority of which were done after 20 weeks of gestation. One prospective study in South Africa reported that, among 1116 women, an additional 37·1% would be classified as having abnormal blood pressure by the American College of Cardiology and American Heart Association criteria; although pregnancy outcomes were not reported separately for these women compared with those with stage 2 hypertension,^24^ it has been reported among pregnant teenagers in the same setting that eclampsia might follow antenatal blood pressure values of less than 140/90 mm Hg.^25^

Importantly, none of the aforementioned studies have reported the diagnostic test properties of various blood pressure categories to define abnormal blood pressure in pregnancy. As blood pressure measurement in pregnancy is a screening test, it should have high sensitivity for the adverse outcomes of interest, which can then lead to the established therapeutic care pathways for hypertension in pregnancy. However, blood pressure measurement is not sensitive, whether American College of Cardiology and American Heart Association categories are used (even elevated blood pressure) or an optimal blood pressure cutoff is estimated from the data and chosen. Perhaps this poor sensitivity is unsurprising, as the hypertensive disorders of pregnancy are one of three leading causes of adverse maternal outcomes (along with obstetric haemorrhage and sepsis), including spontaneous preterm birth, fetal growth restriction, and intrapartum complications for fetal or neonatal outcomes,^26^ and these other conditions could not be predicted by abnormal blood pressure. Nevertheless, although blood pressure measurement is regarded by WHO as good clinical practice that does not require evidence review,^27^ measurement is but one part of quality antenatal care and when normal, might provide minimal reassurance that adverse outcomes are less likely to occur.

The one blood pressure category that was useful in identifying women at increased risk of adverse outcomes (ie, maternal CNS outcomes and fetal or neonatal death) was severe stage 2 hypertension. This finding endorses severe stage 2 hypertension as a condition that should be avoided, as have previous data from well resourced settings,^28^ by contrast to regarding severe hypertension as a condition that can be treated if it occurs.^29^

Our study has several strengths, including our large sample size, community-based recruitment of unselected pregnant women in south Asia and sub-Saharan Africa, and the standardisation of blood pressure readings across the sample, using a pregnancy-validated device.^11^ These factors help to provide evidence for hypertension diagnostic thresholds that is applicable to a large set of pregnant women in these settings.

This study has several limitations. First, many women in Pakistan and Mozambique were enrolled after 20 weeks of gestation, consistent with timing of booking for antenatal care in these settings; therefore, a diagnosis of chronic hypertension could not be evaluated when blood pressure was measured after 20 weeks of gestation. Second, only basic maternal characteristics were available for our adjusted analyses. Third, many women did not have weekly blood pressure measurements from 36 weeks of gestation to delivery as specified in the CLIP protocol, so although the household survey and facility records ultimately informed diagnoses of hypertension, we did not have all relevant blood pressure values for this analysis, particularly from close to term. Women with stage 2 hypertension, whether severe or non-severe, were referred to facilities for antihypertensive treatment (about which we have no further information) and facility care; although not the focus of our analyses, such management is likely to have attenuated the relationship between stage 2 hypertension and outcomes and overestimated the strength of association between lower levels of blood pressure and adverse outcomes. Finally, we did not include in our analysis the outcome of pre-eclampsia given our community setting, and so could not evaluate this as an outcome with the revised hypertension definitions embedded in it.

Pregnant women in under-resourced settings with higher-than-normal blood pressure are at increased risk of adverse outcomes, due to the risks associated with blood pressure of 140/90 mm Hg or higher, particularly with severe stage 2 hypertension. No antenatal blood pressure threshold is sensitive with regards to the adverse maternal, fetal, or neonatal outcomes studied, including data-driven cutoffs. However, the occurrence of severe stage 2 hypertension (≥160/110 mm Hg) is associated with a substantially increased risk of adverse maternal CNS outcomes and fetal or neonatal death, particularly stillbirth, and should be treated as per international guidance.^30^ Antenatal care must aim to provide more than accurate blood pressure measurement to achieve the Countdown 2030 goals.

## Data sharing

A data sharing statement for the CLIP trials is provided in the appendix (pp 294–95).

## Declaration of interests

LAM and PvD report grants from the Bill & Melinda Gates Foundation, during the conduct of the study. All other authors declare no competing interests.
