# Correction to *Lancet Glob Health* 2021; 9: e1119–28

**DOI:** 10.1016/S2214-109X(21)00439-3

**Published:** 2021-09-15

*Bone JN, Magee LA, Singer J, et al. Blood pressure thresholds in pregnancy for identifying maternal and infant risk: a secondary analysis of Community-Level Interventions for Pre-eclampsia (CLIP) trial data.* Lancet Glob Health *2021;* 9: *e1119–28*—In table 3 of this Article, in the comparison of blood pressure category and all higher categories, with all lower blood pressure categories, the risk ratios with severe stage 2 hypertension for all adverse outcomes were incorrect and have been updated. These corrections have been made as of Sept 15, 2021.

