# Supplementary material for: Blood pressure thresholds in pregnancy for identifying maternal and infant risk: a secondary analysis of Community-Level Interventions for Pre-eclampsia (CLIP) trial data
Source: Lancet Glob Health. 2021 Jul 5;9(8):e1119–28. doi: 10.1016/S2214-109X(21)00219-9 (PMC8295039; doi:10.1016/S2214-109X(21)00219-9)
Supplement: Supplementary appendix [file mmc1.pdf]

THE LANCET
Global Health

Supplementary appendix

This appendix formed part of the original submission and has been peer reviewed. We post it as supplied by the authors.

Supplement to: Bone JN, Magee LA, Singer J, et al. Blood pressure thresholds in pregnancy for identifying maternal and infant risk: a secondary analysis of Community-Level Interventions for Pre-eclampsia (CLIP) trial data. *Lancet Glob Health* 2021; published online July 5. https://doi.org/10.1016/S2214-109X(21)00219-9.

Blood pressure thresholds in pregnancy for identifying maternal and infant risk: a secondary analysis of Community-Level Interventions for Pre-eclampsia (CLIP) trial data - Appendix

This appendix contains

Supplementary Tables

- Table S1: CLIP Trials Working Group 3
- Table S2: CLIP Trial primary outcome components 4
- Table S3: Summary of BP measurements for women with and without adverse outcomes 5
- Table S4: Optimal cut-point for maximising sensitivity for various false positive values 6

Supplementary Figure

- Figure S1: Sensitivity and specificity of BP thresholds restricted to BP measured at specific gestational week ranges in the third trimester of pregnancy 8

CLIP Trial Protocol (Original) 10-79

CLIP Trial Protocol (Final) 81-170

CLIP Trial Protocol – Summary of Changes 172-190

CLIP Trial Statistical Analysis Plan (Original) 192-206

CLIP Trial Statistical Analysis Plan (Final) 208-240

CLIP Trial Statistical Analysis Plan – Summary of Changes 242-292

CLIP Trial Data Sharing Statement 293-295

Blood pressure thresholds in pregnancy for identifying maternal and infant risk: a secondary analysis of Community-Level Interventions for Pre-eclampsia (CLIP) trial data - Appendix

Supplementary Tables

Table S1: CLIP Trials Working Group

CLIP Trials Working Group
CLIP Mozambique
Esperança Sevene, Eusébio Macete, Khátia Munguambe, Charfudin Sacoor, Anifa Vala, Helena Boene, Felizarda Amose, Rosa Pires, Zefanias Nhamirre, Marta Macamo, Rogério Chiaú, Analisa Matavele, Faustino Vilanculo, Ariel Nhancolo, Silvestre Cutana, Ernesto Mandlate, Salésio Macuacua, Cassimo Bique, Sibone Mocumbi, Emília Gonçálves, Sónia Maculuve, Ana Ilda Biz, Dulce Mulungo, Orvalho Augusto, Paulo Filimone, Vivalde Nobela, Corsino Tchavana, Cláudio Nkumbula
CLIP Pakistan
Rahat Qureshi, Zulfiqar A Bhutta, Zahra Hoodbhoy, Farrukh Raza, Sana Sheikh, Javed Memon, Imran Ahmed, Amjad Hussain
CLIP India
Mrutunjaya B Bellad, Umesh S Charantimath, Shivaprasad S Goudar, Geetanjali M Katageri, Avinash J Kavi, Amit P Revankar, Ashalata A Mallapur, Umesh Y Ramdurg, Shashidhar G Bannale, Vaibhav B Dhamanekar, Geetanjali I Mungarwadi, Narayan V Honnungar, Bhalachandra S Kodkany, Anjali M Joshi, Uday S Kudachi, Sphoorthi S Mastiholi, Chandrappa C Karadiguddi, Gudadayya S Kengapur, Namdev A Kamble, Keval S Chougala
UBC and KCL
Jeffrey N Bone, Dustin T Dunsmuir, Sharla K Drebit, Chirag Kariya, Mai-Lei Woo Kinshella, Tang Lee, Jing Li, Mansun Lui, Beth A Payne, Diane Sawchuck, Sumedha Sharma, Domena K Tu, Marianne Vidler, Ugochi V·Ukah, Laura A Magee, Peter von Dadelszen
**CLIP Trial Steering Committee**
J Mark Ansermino, Ana Pilar Betrán, Richard Derman, Shafik Dharamsi, France Donnay, Sharla Drebit, Guy Dumont, Susheela M Engelbrecht, Veronique Fillipi, Tabassum Firoz, William Grobman, Marian Knight, Ana Langer, Simon Lewin, Gwyneth Lewis, Craig Mitton, Nadine Schuurman, Andrew Shennan, Joel Singer, Jim Thornton, Hubert Wong
**CLIP Trial Executive Committee**
Olalekan O Adetoro, Mrutunjaya M Bellad, Zulfiqar A Bhutta, Peter von Dadelszen, Shivaprasad S Goudar, Jerker Liljestrand, Laura A Magee, Ashalata A Mallapur, Khátia Munguambe, Beth A Payne, Rahat Qureshi, Charfudin Sacoor, Esperança Sevene, Sumedha Sharma, John O Sotunsa, Marianne Vidler
**CLIP Data Safety and Monitoring Board (DSMB)**
Romano Nkumbwa Byaruhanga, Brian Darlow, Eileen Hutton, Mario Merialdi, Lehana Thabane

Table S2: CLIP primary outcome components

	Definition
**MATERNAL**	
**Maternal death***	Number of deaths during pregnancy or within 42 days of pregnancy (or last contact day if contact not maintained to 42 days)
**Maternal morbidity**	Number of women with one/more life-threatening complications of pregnancy during pregnancy or within 42 days of pregnancy or last contact day if contact not maintained to 42 day. These are the serious end-organ complications of pre-eclampsia, other major causes of maternal mortality, or life-saving interventions related to one of the aforementioned.
Serious end-organ complications of preeclampsia	
Eclampsia*	Occurrence of generalised convulsions during pregnancy, labour or within 42 days of delivery in the absence of epilepsy or another condition predisposing to convulsions
Stroke*	Hemiparesis and/or blindness developed during pregnancy or in the 42 days postpartum lasting greater than 48 hours
Coma*	Prolonged unconsciousness ≥12 hours
Antepartum haemorrhage	Vaginal bleeding ≥ 15 mL with or without pain before the onset of labour
Disseminated intravascular coagulation	Abnormal bleeding from mucosa (mouth and/or ears)
Other major maternal complication	
Obstetric sepsis	In the community, defined as fever and one of: abdominal/uterine tenderness, foul smelling vaginal discharge/lochia, productive cough and shortness of breath, dysuria or flank pain, headache and neck stiffness
Vesicovaginal or rectovaginal fistula	Continuous loss of urine and/or faeces after delivery
Life-saving interventions	
Cardiopulmonary resuscitation	A set of emergency procedures including chest compressions and lung ventilation applied in cardiac arrest victims
Mechanical ventilation	Intubation and ventilation not related to Caesarean section or anaesthesia
Blood transfusion	Of one or more units
Interventions for major postpartum haemorrhage	Brace sutures, external and internal uterine compression, anti-shock garment use, internal iliac artery ligation and/or hysterectomy with or without transfusion
Dialysis	Haemodialysis and/or peritoneal dialysis
**FETAL/NEWBORN**	
**Fetal/neonatal mortality**	One or more of stillbirth, early neonatal mortality, or late neonatal mortality per 1000 identified pregnancies
Stillbirth	Fetal death at $\geq 20^{+0}$ weeks' gestation and/or ≥500g birthweight
Early neonatal mortality	Newborn death at 0-7 days of postnatal life
Late neonatal mortality	Newborn death at 8-28 days of postnatal life

** These are the components of the maternal CNS composite.*

Table S3: Summary of BP measurements for women with and without adverse outcomes (presented as median [IQR])

Outcome ǂ	Present	N women	Mean sBP* (mmHg)	Mean dBP* (mmHg)	Maximum sBP (mmHg)	Maximum dBP (mmHg)
**CLIP composite**	No	15,893	105·8 [99·7, 110·8]	66·4 [62·0, 70·0]	113 [105, 119]	72 [66, 77]
	Yes	4860	107·0 [100·0, 112·7]	67.3 [62·5, 71·8]	114 [105, 120]	73 [67, 79]
**Maternal composite**	No	18,679	106·0 [99·7, 111·0]	66·5 [62·0, 70·2]	113 [105, 119]	73 [67, 78]
	Yes	2047	107·5 [100·2, 113·2]	67·5 [62·5, 72·0]	114 [105, 121]	73 [67, 79]
Maternal CNS composite	No	20,426	106·0 [99·8, 111·0]	66·6 [62·0, 70·5]	113 [105, 119]	73 [67, 78]
	Yes	284	108·3 [99·5, 114·3]	67·0 [61·4, 71·0]	114 [104, 123]	72 [65, 81]
**Fetal/newborn death**	No	18,469	106·0 [99·8, 111·0]	66·5 [62·0, 70·3]	113 [106, 120]	73 [67, 78]
	Yes	1670	107·2 [100·0, 114·0]	67·5 [62·2, 73·0]	114 [105, 121]	74 [66, 80]
Stillbirth	No	18,624	106·0 [99·8, 111·0]	66·5 [62·0, 70·2]	113 [106, 120]	73 [67, 78]
	Yes	815	108·3 [100·2, 115·7]	68·2 [62·5, 73·6]	115 [105, 122]	74 [67, 81]

CLIP (Community-Level Interventions for Pre-eclampsia), CNS (central nervous system), dBP (diastolic blood pressure), IQR (interquartile range), sBP (systolic blood pressure)

** Mean sBP and mean dBP refer to the value per woman, with the median [IQR] as the statistic used to summarise the mean BP values for the group overall.*

*ǂ For outcome definitions, see **Table S2**.*

Table S4: Optimal cut-point for maximising sensitivity for various false positives

sBP						
	5% False positives		10% False positives		20% False positives	
**Outcome**	Sensitivity (%)	Optimal cut-off (mmHg)	Sensitivity (%)	Optimal cut-off (mmHg)	Sensitivity (%)	Optimal cut-off (mmHg)
CLIP composite	7	135	13	128	22	123
Maternal composite	7	135	12	129	22	123
Maternal CNS composite	10	136	19	129	28	123
Fetal/newborn death	9	135	15	129	24	123
Stillbirth	12	135	18	129	28	123
**dBP**						
	5% False positives		10% False positives		20% False positives	
**Outcome**	Sensitivity (%)	Optimal cut-off (mm Hg)	Sensitivity (%)	Optimal cut-off (mm Hg)	Sensitivity (%)	Optimal cut-off (mm Hg)
CLIP composite	7	91	13	85	23	81
Maternal composite	3	94	8	87	16	82
Maternal CNS composite	1	104	5	92	10	85
Fetal/newborn death	8	92	14	86	26	81
Stillbirth	11	92	18	86	29	81

CNS (central nervous system), dBP (diastolic blood pressure), sBP (systolic blood pressure)

Blood pressure thresholds in pregnancy for identifying maternal and infant risk: a secondary analysis of Community-Level Interventions for Pre-eclampsia (CLIP) trial data - Appendix

Supplementary Figure

Figure S1: Sensitivity and specificity of blood pressure thresholds restricted to blood pressures measured at specific gestational week ranges.

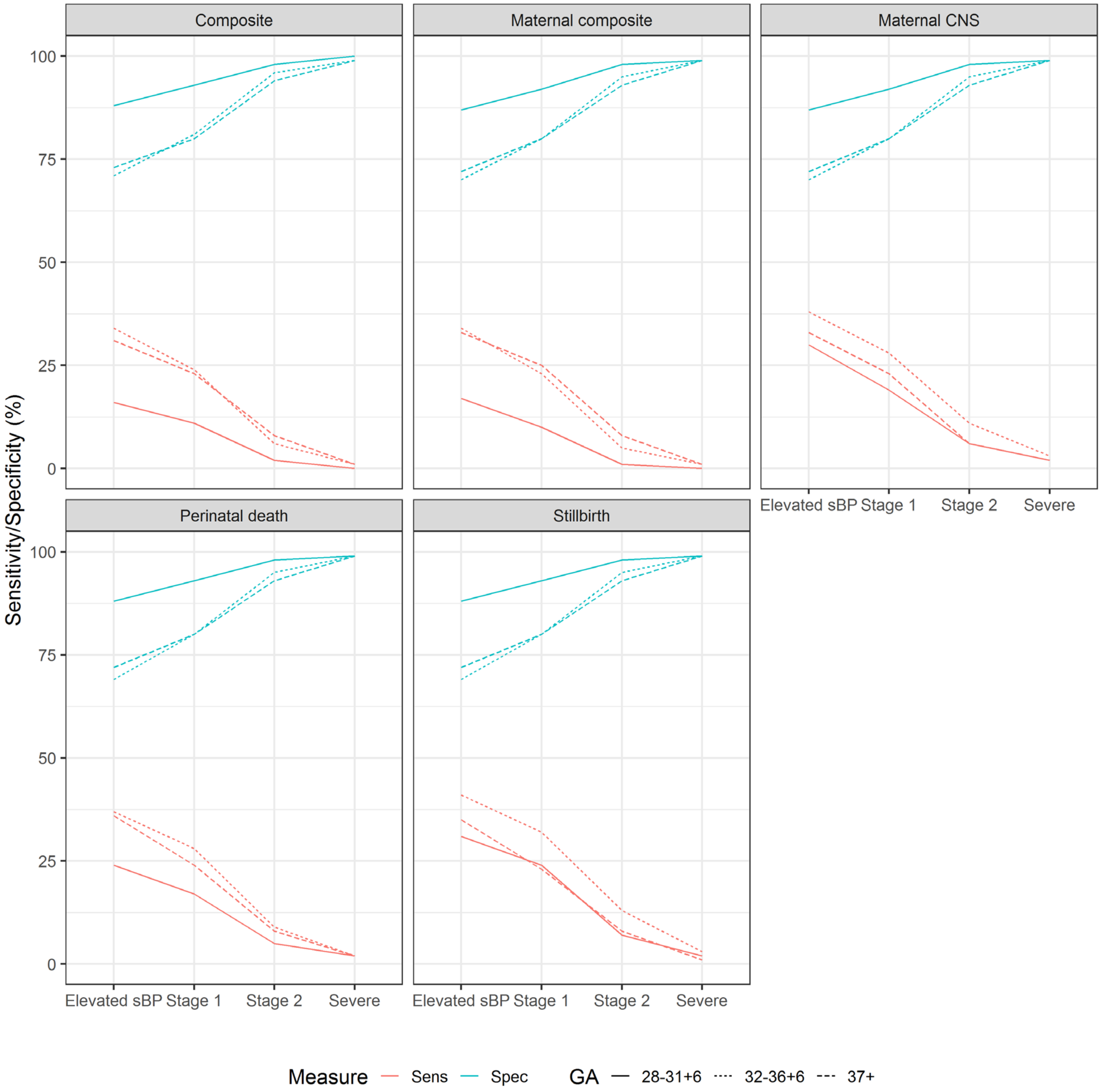

dBP (diastolic blood pressure), sBP (systolic blood pressure), sens (sensitivity), spec (specificity)

Elevated sBP (sBP 120-129 mmHg and dBP <80 mmHg)
Stage 1 (sBP 130-139 mmHg or dBP 80-89 mmHg, or both)
Stage 2 (non-severe; sBP 140-159 mmHg or dBP 90-109 mmHg, or both)
Severe (Stage 2; sBP ≥160 mmHg or dBP ≥110 mmHg, or both)

Blood pressure thresholds in pregnancy for identifying maternal and infant risk: a secondary analysis of Community-Level Interventions for Pre-eclampsia (CLIP) trial data - Appendix

CLIP Trial Protocol (Original)

THE CLIP (COMMUNITY LEVEL INTERVENTIONS FOR PRE-ECLAMPSIA) CLUSTER RANDOMIZED CONTROLLED TRIAL

SECTION 1: BACKGROUND

1.1 WHAT IS THE PROBLEM TO BE ADDRESSED?

Pre-eclampsia remains a leading cause of maternal and perinatal mortality and morbidity [1]. It is a pregnancy-specific disease characterised by *de novo* development of endothelial dysfunction resulting in the most common diagnostic features, concurrent hypertension and proteinuria, sometimes progressing into a multiorgan cluster of varying clinical features (Figure 1) [1]. Poor early placentation is especially associated with early-onset disease [1]. Predisposing cardiovascular or metabolic risks for endothelial dysfunction, as part of an exaggerated systemic inflammatory response, might dominate in the origins of late onset pre-eclampsia [1]. Because the multifactorial pathogenesis of different pre-eclampsia phenotypes has not been fully elucidated, prevention and prediction are still not possible, thus symptomatic clinical management should be focussed on the prevention of maternal morbidity (e.g., eclampsia) and mortality.

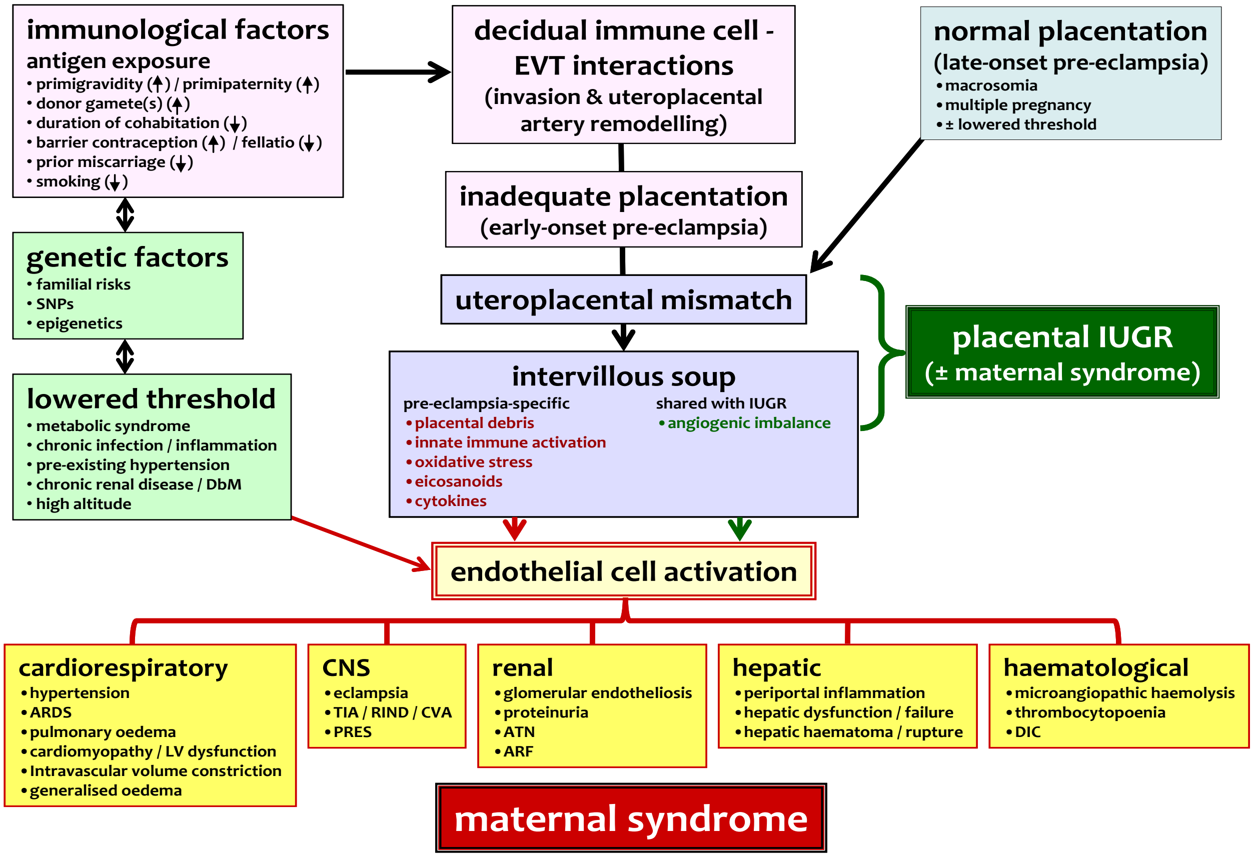

Figure 1 The origins and consequences of pre-eclampsia

ARDS acute respiratory distress syndrome; **ARF** acute renal failure; **ATN** acute tubular necrosis; **CVA** cerebrovascular accident; **DbM** diabetes mellitus; **DIC** disseminated intrasvascular coagulation; **EVT** extravillous trophoblast; **IUGR** intrauterine growth restriction; **LV** left ventricular; **PRES** posterior reservible leukoencephalopathy syndrome; **RIND** reversible ischaemic neurological deficit; **SNPs** single nucleotide polymorphisms; **TIA** transient ischaemic attack

Expectant management of women with early-onset disease to improve perinatal outcome should not preclude timely delivery - the only definitive step to initiate cure [1]. Pre-eclampsia foretells raised rates of

cardiovascular and metabolic disease in later life, which could be a reason for subsequent lifestyle education and intervention [1].

Globally, pre-eclampsia is the second-leading cause of maternal mortality, resulting in an estimated 76,000 maternal deaths annually. In addition, 500,000 fetal and newborn lives are lost annually due to the perinatal consequences of pre-eclampsia. Over 99% of these deaths occur in low and middle income countries (LMICs), primarily in South Asia and Sub-Saharan Africa [2].

As a result of the focus on postpartum haemorrhage (PPH; the leading cause of maternal death) over the past decade, rates of PPH-related maternal mortality are falling. This fall in PPH-related maternal mortality is driving the improvements in the maternal mortality ratio (MMR; maternal deaths per 100,000 live births) observed in countries such as Bangladesh and Pakistan. Therefore, the proportional contribution of pre-eclampsia-related mortality to the MMR is increasing. It may be that some of the residual PPH-related mortality arises from the consumptive coagulopathy of severe pre-eclampsia, especially when complicated by abruption [1].

Clearly, improvements in PPH-related outcomes have no direct impact on perinatal mortality as the woman is delivered before she bleeds. However, we recognise that maternal death places infants at a 6-fold increased risk of infant mortality.

In general, previous research in this field has focussed on institutional level interventions with $MgSO_4$ (eclampsia prevention and treatment [3-6]) and the treatment of severe pregnancy hypertension [7]. However, if we limit ourselves to studying inpatient, facility-level, interventions with fully assessed treatment options, many women will die or be irreversibly affected by pre-eclampsia (e.g., either moribund or having suffered a stroke) prior to arriving at the inpatient facility.

Of all the Millennium Development Goals (MDGs), MDG 5 (a 75% reduction in maternal mortality from 1990 levels by 2015) is that which is proving hardest to achieve. Maternal lives lost from pre-eclampsia and eclampsia result from delays in triage, transport and treatment. As such, the solution has to reside in getting care to women in the community as current health systems either never see affected women (who die either in their community or en route to formalised care) or receive women who are moribund and beyond salvage regardless of the quality of care being offered.

Currently, Bangladesh is one of the few countries on target to achieve MDG 5; however, the remaining issues to be addressed (such as pre-eclampsia) will be harder to achieve – partly because women with pre-eclampsia feel well until they are critically ill. It is probable that in Nigeria, Mozambique, Pakistan and India, MDG 5 will not be achieved.

The CLIP trial is a singular step towards addressing the excess maternal and perinatal mortality that derive from the failure to identify and rapidly manage pre-eclampsia and eclampsia at the community level in LMICs.

1.2 WHAT ARE THE PRINCIPAL RESEARCH QUESTIONS TO BE ADDRESSED?

Hypothesis

That implementing community-level evidence-based care will reduce pre-eclampsia-related maternal and perinatal mortality and major morbidity by addressing 'three delays' in triage, transport, and treatment.

Objective

To reduce pre-eclampsia-related, and all-cause, maternal and perinatal mortality and major morbidity by 20% or more in intervention clusters in Ogun State (Nigeria), Maputo and Gaza Province (Mozambique), Sindh Province (Pakistan) and in Karnataka State (India).

1.3 Relevant Convergent Activities

***Pre-requisite knowledge: CLIP Feasibility Study** (co-PIs: P von Dadelszen & R Qureshi)*

The Feasibility Study is being conducted in Nigeria, Mozambique, Pakistan, and India in preparation for the CLIP cluster randomised controlled trial (cRCT; CW UBC REB # H12-00132). In Pakistan we are building on the previous community- and primary health centre (PHC)-level perinatal intervention studies and trials led by Professor Bhutta [8-12].

The purpose of each national CLIP Feasibility Study is to understand the health care system, explore key methodological issues for CLIP, and engage in a dialogue with the key stakeholders (to inform the development of culturally appropriate tools and educational materials for use in CLIP).

The *health care system organisation and infrastructure capacity* must be clear, related to antenatal care models and pre-eclampsia/eclampsia monitoring, triage, management, referral and maternal transfer, and PHC and referral facility pre-eclampsia/eclampsia treatment.

Key methodological issues for CLIP include: community demographics, pre-eclampsia/eclampsia prevalence rates and rates of associated maternal and perinatal morbidity and mortality; data collection methods and informational systems for population surveillance; specific barriers to conducting a cRCT, including recruitment feasibility, capacity to implement community intervention, and accurate data collection; and cost identification to conduct the CLIP trial and an adequately powered cRCT in the identified country.

Finally, we must receive input from *key community stakeholders* (cultural and/or community beliefs/practices/influences/attitudes), *community-based health care providers (cHCP)* (professional scope of practice regulations and/or legal barriers and potential for task shifting), *facility care providers* (provider knowledge and competency related to pre-eclampsia/eclampsia and resource/informational capacity for provider training), and *key decision-making stakeholders* (support, commitment, and financial/schedule feasibility to remedy identified barriers). By engaging relevant stakeholders from the beginning of the trial planning process, we will work towards long term sustainability of the intervention, should it prove to be effective.

Each national Feasibility Study is using a mixed methods approach (quantitative, participatory, and formative, with community mapping) based on the normalization process model and will utilize literature reviews, target interviews, focus groups, and survey tools. Target interviews and focus group data are being recorded and transcribed; observations and assessments written up as field notes. The core approach is similar across the four study sites, but will allow for tailoring according to individual setting and cultural context. We are drawing on models of how interventions are embedded in practice (e.g., the normalisation process [13-15] and psychological theory [16]) as frameworks for this assessment.

A summary of key findings from the Feasibility Studies to date, including stakeholders engaged and information on health care system organisation and current scope of practice of cHCPs can be found in Appendix A of this document.

***Tools & Funding: PIERS On the Move (POM)** (co-PIs: JM Ansermino & P von Dadelszen [funded by Saving Lives at Birth])*

Risk stratification using the miniPIERS model aims to address three delays that lead to the increased incidence of maternal mortality in LMICs: delays in triage, transport, and treatment (adapted from Thadeus and Maine)[17].

The *delays in triage* relate to the presentation of women late in the clinical course of their hypertensive

disorder of pregnancy (HDP) and the failure to have the nature and potential severity of their condition recognised. In many settings, women receive no antenatal care; therefore achieving some degree of practical oversight and getting a triage mechanism into the hands of women and their immediate caregivers is a priority. Often, immediate care is given by cHCPs and at PHCs. Once women enter the formal health care system [hospitals providing either basic or comprehensive emergency obstetric care (EmOC)], the miniPIERS and fullPIERS models will identify those most at risk of adverse outcomes so that they can receive appropriate interventions in the timeliest manner possible [18;19].

To identify women for whom ***transport*** is a particular priority, cHCPs and the nurses, medical assistants, and physicians staffing PHCs need to have available to them tools that risk stratify women with pre-eclampsia; the miniPIERS model will do this.

Testing the ***hypothesis*** that the PIERS models, based on a combination of maternal and fetal predictors, will accurately identify women at incremental risk of maternal complications of pre-eclampsia, we have developed the fullPIERS model (see below) in eight tertiary academic centres in Canada, UK, NZ, and Australia[19]; and developed and validated the miniPIERS model in five LMICs (see below). The fullPIERS model identifies risk most accurately over the following 48h, but also for up to 7 days. The miniPIERS model performs with less accuracy, but may have greater impact as it is more generalisable to all levels of care in high, middle, and low income settings. Only miniPIERS will be utilised as a triage tool in the CLIP trial, as we anticipate that a model solely derived from, and validated in, LMICs will be the most accurate and have greatest validity for the CLIP trial.

The fullPIERS model (Figures 2 & 3) In the fullPIERS data set, we have identified those women with pre-

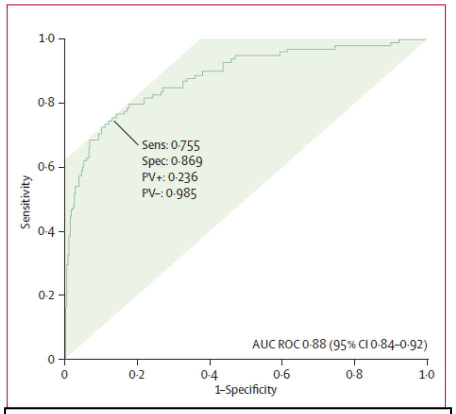

Figure 2: Performance of the fullPIERS model

Combined adverse maternal outcomes predicted within 48 h of eligibility on the basis of only data recorded before the outcome (an online tool to calculate fullPIERS probabilities is available on the study website). AUC ROC=area under the curve of the receiver operating characteristic. PV–=negative predictive value. PV+=positive predictive value. Sens=sensitivity. Spec=specificity.

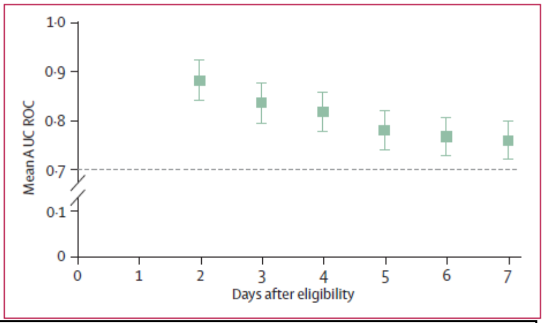

Figure 3: Mean fullPIERS AUC from 2-7 days

Error bars show 95% CIs. AUC ROC=area under the curve of the receiver operating characteristic.

eclampsia who are at increased risk of maternal complications and have been able to grade this risk [18-28]. The independent predictors of adverse maternal outcome are: gestational age at eligibility, chest pain/dyspnea, SpO_2, creatinine, aspartate transaminase (AST), and platelet count. fullPIERS assesses risk up to 7 days after eligibility (AUC ROC 0.76 [95% CI 0.72, 0.80] [19].

An ancillary, and cost neutral, benefit of this project will be to externally validate the fullPIERS model in LMIC settings.

The miniPIERS model (Figures 4 & 5) Based on data from 2081 women admitted to a miniPIERS centre in Brazil, South Africa, Uganda, Pakistan and Fiji, the miniPIERS model predicts adverse maternal events in women with any HDP. The components of the miniPIERS model are: parity (muliparity vs. Nuliparity) gestational age at identification; symptoms (Y/N) of headache/ visual disturbances, chest pain/ dyspnoea,

or abdominal pain with vaginal bleeding; systolic blood pressure (sBP); and dipstick proteinuria. The AUC ROC is 0.8788 [95% CI 0.4744, 0.80], decreasing to 73 on internal validation.

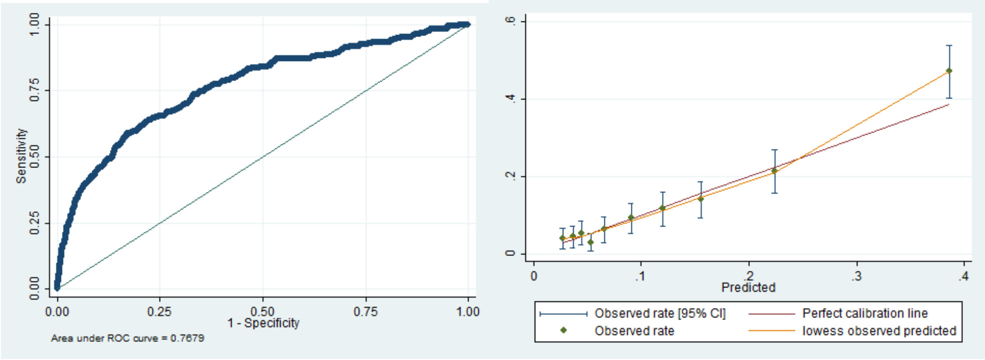

Figures 4 & 5: miniPIERS model ROC curve and calibration curve

Using funds from within the wider PRE-EMPT grant, miniPIERS modelling (development and validation) was completed in September 2012. The final model is a prerequisite for development of the management algorithms for the CLIP cRCT. miniPIERS will be a component of the CLIP trial hosted on a mobile platform.

The miniPIERS mobile phone application to be used by cHCPs in the CLIP trial: (i) provides local, rapid and accurate risk assessment, referral, and treatment advice for pre-eclampsia, and (ii) transmits information to referral centres for co-ordination of triage, transportation, and treatment.

By harnessing the processing and battery power of the phone and widespread availability of cellular services in Africa and South Asia, we will empower cHCPs to rapidly and reliably assess a woman's risk of pre-eclampsia complications in real-time, and to take action before complications arise.

Tools & pre-requisite knowledge: Blood pressure monitoring in LMICs *(PI: A Shennan)*

Using parallel funding from the Bill and Melinda Gates Foundation (BMGF), and building on two decades' experience, we have identified the optimal automated sphygmomanometer for use in LMIC settings, the Microlife BP 3AS-2, developed for Professor Shennan's parallel CRADLE (Community blood pressure monitoring in Rural Africa: Detection of underLying pre-Eclampsia) research programme in South Africa and Tanzania. Through his relationship with Microlife, Professor Shennan has arranged for us to bulk purchase 900 machines that have been purpose-modified for CLIP. See Appendix D for details on the validation study for the Microlife BP 3AS-2.

Pre-requisite knowledge: Oral antihypertensive therapy for severe pregnancy hypertension - review *(PI: LA Magee)*

Traditionally, severe hypertension (usually defined as sBP≥160mmHg and/or dBP≥110mmHg) has been treated with short-acting parenteral antihypertensive agents, most frequently, intravenous (i.v.) hydralazine or labetalol [29]. Parenteral agents require more resources than do oral antihypertensive agents, in terms of equipment (i.e., i.v. tubing, syringes, and needles) and personnel (as administration is by nurses or doctors). Also, parenteral agents require more monitoring and supervision as they are rapidly-acting and have the potential to lower BP quickly and cause maternal hypotension and fetal compromise.

Oral antihypertensive therapy is used for hypertensive urgencies in pregnancy in some jurisdictions, with apparently good effect. In the regional pre-eclampsia guidelines from Yorkshire, UK, labetalol 200 mg is administered orally before i.v. access is secured, with a repeat dose given if no response is seen after 30 minutes [30]. The UK National Institute for Health and Clinical Excellence (NICE) Hypertension in

Pregnancy guideline recommends oral labetalol or nifedipine for the treatment of severe hypertension in critically ill women during pregnancy or after birth [31] .

In our literature review of oral antihypertensive therapy for severe hypertension in and outside pregnancy, we identified 15 RCTs (915 women) in pregnancy, one postpartum and 34 outside pregnancy (Firoz *et al*; manuscript in preparationpreparation).preparation Most trials in pregnancy compared oral/sublingual (SL) nifedipine capsules (8-10mg) with another agent, usually parenteral hydralazine or labetalol. 84-100% of women achieved successful treatment with nifedipine with less than 1% experiencing hypotension. Target BP was achieved ~50% of the time with oral labetalol and methyldopa, suggesting that each may be a reasonable alternative to nifedipine. Outside pregnancy, most trials compared short-acting nifedipine with SL captopril (6 trials, 251 subjects) with similar rates of success (88% vs. 76%). The results are presented in detail in Appendix B.

In our review of the pharmacokinetics of antihypertensive agents found commonly on the essential medicines lists of LMICs (Lalani *et al. JOGC*), the onset, peak, and duration of action of agents were very similar (Table 1).

Table 1 Oral antihypertensives for severe pregnancy hypertension

Drug	Dosage	Onset	Peak	Duration
Atenolol	25 – 50 mg	1hr	2-4hr	24hr (dose dependent)
Labetalol	200 mg	20min – 2hr	1-4 hr	8-12hr (dose dependent)
Methyldopa	500 mg – 2 g	40 min	3-6hr	12-24hr
Nifedipine intermediate-acting	10 mg	30min	4hr	12hr
Nifedipine capsule	5 -10 mg	5-10min	30min	6.5hr

In summary, 750mg (alpha-)methyldopa has been chosen as the optimal antihypertensive to treat severe pregnancy hypertension in CLIP for several reasons: (i) it has been well-studied in RCTs in pregnancy for non-severe hypertension, and more limited RCT data for severe hypertension suggest that the drug is effective; (ii) it has an acceptable onset, peak, and duration of action for treatment of severe hypertension; (iii) there is a high level of comfort with methydopa due to its long history of use as an antihypertensive of first choice in pregnancy, making it an acceptable choice to most practitioners; (iv) it is likely to face fewer barriers in terms of acceptability during implementation as it does not interact with $MgSO_4$, whereas unfounded misconceptions persist about a nifedipine-magnesium interaction [32]; (v) it is widely available on nearly all LMIC essential medicines lists, and it is affordable, which speaks to feasibility during post-trial implementation and scale-up; and (vi) as an oral treatment it is more acceptable for administration by cHCPs than complex intravenous medications.

***Pre-requisite knowledge: Options for safe community administration of $MgSO_4$** (PI: LA Magee)*

We have reviewed the current literature that pertains to intramuscular (i.m.)-only administration of a $MgSO_4$ loading dose at the community level (Gordon *et al*; JOGCJOGCJOGC [in press]). We have determined that a single 10g i.m. dose of $MgSO_4$ would be safe (even in the presence of anuria) and would achieve therapeutic Mg^{2+}

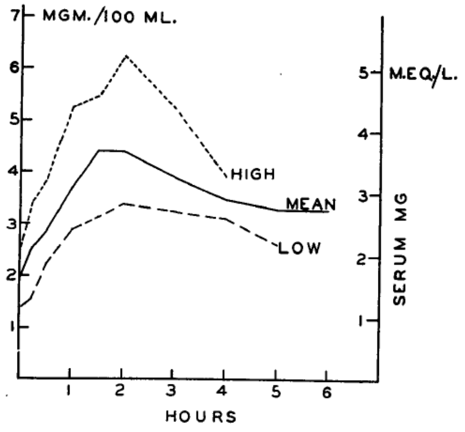

Figure 6 Effect of 10g IM $MgSO_4$ loading dose on serum Mg^{2+} concentration

levels within 45 minutes and result in sustained levels for 4-6 hr (Figure 6) [33]. A more thorough description of the results of this systematic review is provided in Appendix B.

Pre-requisite knowledge: Current CLIP-related maternity services provision in Nigeria, Mozambique, Pakistan & India (PI: P von Dadelszen)

We have reviewed the current availability of CLIP-relevant personnel, drugs, devices and interventions at all levels of the public, NGO and private health care systems in Nigeria, Mozambique, Pakistan and India.

Table 2 CLIP-related maternity care service provision

	Nigeria	Mozambique	Pakistan	India
**Community-based care**				
**HOME-BASED CARE**				
**cHCPs**	**Community Health Extension Workers (CHEWs; cover 2500 population each)**	**Agente Communitarios de Saude (ACSs; cover 500-2000 population each)**	**Lady Health Workers (LHWs; cover 1000 population each)**	**Auxiliary Nurse Midwives (ANMs; cover 3000-5000 population each) Accredited Social Health Activists (ASHAs; cover 1000 population each)**
**Delivery in the home**	**70% births at home**	**30% births at home**	**40% births at home**	**5-10% births at home**
**PHC-based care**				
**Medical officers**	**Sometimes**	**No**	**Yes**	**Yes**
**Nurses**	**Yes**	**Yes**	**Yes**	**Yes**
**cHCPs**	**CHEWs, Health Assistants (HA)**	**ACSs**	**LHWs**	**ANMs, Lady Health Visitors (LHV)**
**BP device**	**Yes (93%)**	**Yes**	**Yes (100%)**	**Yes (100%)**
**Urine dipsticks**	**Sometimes (20%)**	**Yes**	**Few (37%)**	**Yes (90%)**
**Laboratory testing**	**Sometimes (11%)**	**Some**	**Yes (100%)**	**No**
**Ultrasound**	**Sometimes (5%)**	**No**	**Sometimes**	**No**
**Methyldopa**	**Yes (purchased by patient at pharmacy)**	**Yes**	**Yes**	**Yes**
**MgSO4**	**Availability of some anticonvulsant (82%)**	**Yes**	**Sometimes (38%)**	**Sometimes (56%)**
**Attend deliveries**	**Yes**	**Yes**	**Yes**	**Yes**
**BEmOC**	**Varies**	**No**	**Yes**	**No**
**Hours of available care**	**Information pending**	**Information pending**	**Day time only**	**Some services are 24/7**
**Facility-based care**				
**Number of facilities serving CLIP clusters**	**936 (32 public, 904 private)**	**Information pending**	**12 (3 public, 9 private)**	**96**
**BEmOC in CLIP clusters**	**Information pending**	**Information pending**	**17 (14 public, 3 private)**	**Information pending**
**CEmOC in CLIP clusters**		**1**	**12**	**Majority**
**General practitioners/ medical officers**	**Yes**	**Information pending**	**Yes**	**Yes**
**Specialists**	**Rarely**	**Information pending**	**Information pending**	**Yes**

**Nurses**	**Yes**	**Information pending**	**Information pending**	**Yes**
**cHCPs**	**Information pending**	**Information pending**	**Information pending**	**ANMs**
**BP device**	**Yes**	**Yes**	**Yes (100%)**	**Yes (100%)**
**Urine dipsticks**	**Yes**	**Yes**	**Sometimes**	**Yes**
**Laboratory testing**	**Yes**	**Yes**	**Yes (100%)**	**Yes**
**Ultrasound**	**Yes**	**Yes**	**Yes**	**Yes**
**Fetal heart assessment**	**Pinard; US**	**Pinard, Doppler, US**	**Pinard, CTG, US**	**Pinard, Doppler, CTG, US**
**Methyldopa**	**Sometimes (30%)**	**Yes**	**Yes**	**Yes**
**Other antihypertensives**	**Sometimes (7%)**	**Yes**	**Yes**	**Yes**
**MgSO4**	**Sometimes (20% stock outs)**	**Yes**	**Some**	**Yes**

ANM auxilary nurse midwife; **APE** agente polivalente elementares; **ASHA** accredited social health advocate; **BEmOC** basic emergency obstetric care; **BP** blood pressure; **CEmOC** comprehensive EmOC, **cHCP** community health care provider; **CHEW** community health extension worker; **CTG** cardiotocograph; **LHW** lady health worker; **MO** medical officer; **PHC** primary health centre; **US** ultrasound

SECTION 2: THE CLIP TRIAL

2.1 THE CLIP TRIAL DESIGN

We have designed a two-phased community (including PHC-level) cRCT encompassing both rural and urban settings to be fully powered in each of:

- Ogun State, Nigeria
- Maputo and Gaza Province, Mozambique
- Hyderabad and Matiari districts in Sindh Province, Pakistan.
- Belgaum and Bagalkot districts in Karnataka State, India

The trial will be phased from the Pilot CLIP trial to Definitive CLIP trial on the basis of a satisfactory rate of use (≥50%) of the CLIP 'package of care' in appropriate women n all countries but Mozambique. Mozambique will be unique in that they will rely on an extended period of feasibility to pilot test all Trial systems and tools before directly beginning a definitive trial. Foregoing the Pilot in Mozambique was felt to be appropriate based on their experience with community-based surveillance and will ensure timelines of the trial are met within a manageable budget.

For all other countries, use of the package in the Pilot phase will be defined as appropriate referral (urgent or non-urgent) to a facility able to provide comprehensive emergency obstetric care (CEmOC) in appropriate women during the first six months of the Pilot CLIP trial (Figure 2). This accelerated transition from Pilot CLIP trials to Definitive CLIP trials will: (i) save resources in the long term as the research infrastructure will be maintained in all countries and, thereby, start-up costs will be reduced as will core infrastructure costs at UBC, and (ii) deliver more rapidly the primary CLIP research question, does the CLIP community intervention improve outcomes for mothers and babies?

Full details of the sample size calculation for both Pilot and Definitive trial phases can be found in Table 3.

Monitoring during the Pilot phase of the trial to determine rate of use of the CLIP package of care will be performed by the cHCPs in an ongoing manner after the Pilot trial begins. In the Pilot Trial, if the percentage use of the CLIP 'package of care' is <50% in any given jurisdiction at 6 months after the trial start date, but climbing, we plan to re-assess at 7, 8, and 9 months before making a decision whether or not to continue the Trial. Similarly, if there is an identifiable barrier or number of barriers found then we will attempt to overcome the barrier(s) and reassess, deferring the commencement of the Definitive CLIP trial in that jurisdiction, while proceeding in the others. All decision will be made in consultation with the BMGF (sponsor), after reviewing the advice of the CLIP trial DSMB, and complying with relevant REB guidance. The rationale for continuing the Pilot CLIP trial beyond the initial nine months is to beta-test the whole Trial process through at least two household surveillance cycles, as well as testing and validating any tools designed specifically for use in the Definitive CLIP trial but not related to the primary outcome.

All tools related to the Definitive CLIP trial primary outcome will be field-tested prior to the Pilot CLIP trial.

For CLIP Pilot and Definitive trials

Control clusters Women receive the current pattern of care

Intervention clusters Women and their communities receive the CLIP intervention

The primary implementers of the CLIP intervention will be cHCPs, the nature of whom will differ between countries. In Nigeria, these are Community Health Extension Workers (CHEWs; in Mozambique, these are Agente Polivalente Elementares (APEs); in Pakistan, these are Lady Health Workers (LHWs); and in India, these are Accredited Social Health Advocates (ASHAs) and Auxiliary Nurse Midwives (ANMs).

Unless context-specific, we will use the term cHCP to describe these cadres of health providers for simplicity. The training and experience of these groups are described in Table 2.

As the primary outcome changes between the Pilot and Definitive Trials, the women in the Pilot Trials will contribute to the sample size of the Definitive Trials. We plan that each pair of Pilot and Definitive CLIP Trials will span 48 months (36 months' recruitment) as shown in Figure 7 below. In India, the Pilot phase

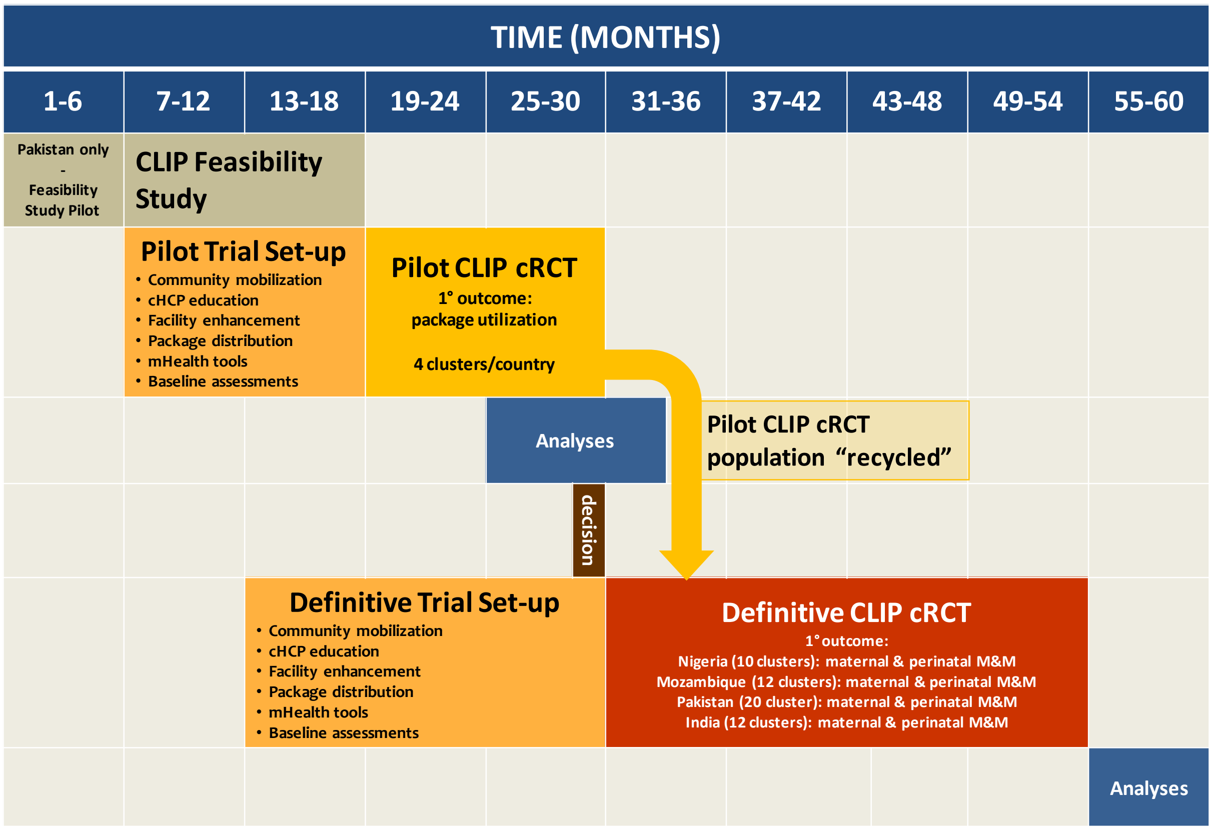

will be shorter due to the difference in surveillance process as described later in this protocol.

Figure 7: CLIP trial timeline
cHCP community health care provider, **cRCT** cluster randomised controlled trial, **mHealth** mobile health

2.2 Interventions

The interventions will be:

Control group: current practice (around antenatal care, referral to facility, and initiation of therapy).

Study group: The CLIP intervention consists of (i) *community engagement* including community leaders, the women of the communities themselves, and their mothers, husbands, and mothers-in-law, regarding pre-eclampsia, its origins, symptoms, signs, and potential consequences, pre-permissions for maternal transport, and fundraising activities around transport and treatment costs; (ii) *provision of HDP oriented antenatal care through CLIP visits and use of CLIP POM tool* (for risk stratification), and (iii) *use of the CLIP package for women with a CLIP 'trigger'* (i.e., oral antihypertensive therapy when indicated, intramuscular (i.m.) $MgSO_4$ when indicated; and appropriate referral to an CEmOC facility when indicated) (Figure 8). cHCPs will assess pregnant women with a target frequency of every 4 weeks at a minimum, and according to protocol (Appendix C: CLIP cHCP Working Protocol). These visits can occur in the home or PHC as both are considered part of the community for the purpose of the CLIP Trial. They

will be trained to enquire about women's symptoms (using country-specific pictograms), take women's BP (using sBP as it more closely reflects the risk for hypertensive stroke than does dBP[11;12]), and check urine for protein using dipstick on the first visit or on any subsequent visits for sBP ≥140 mmHg. This will inform the diagnosis and risk assessment of women with pre-eclampsia.

Both groups: CEmOC facility enhancement to promote evidence-based care of women with pre-eclampsia/eclampsia who are referred to CEmOC facilities, focussing on the WHO guidelines [34].

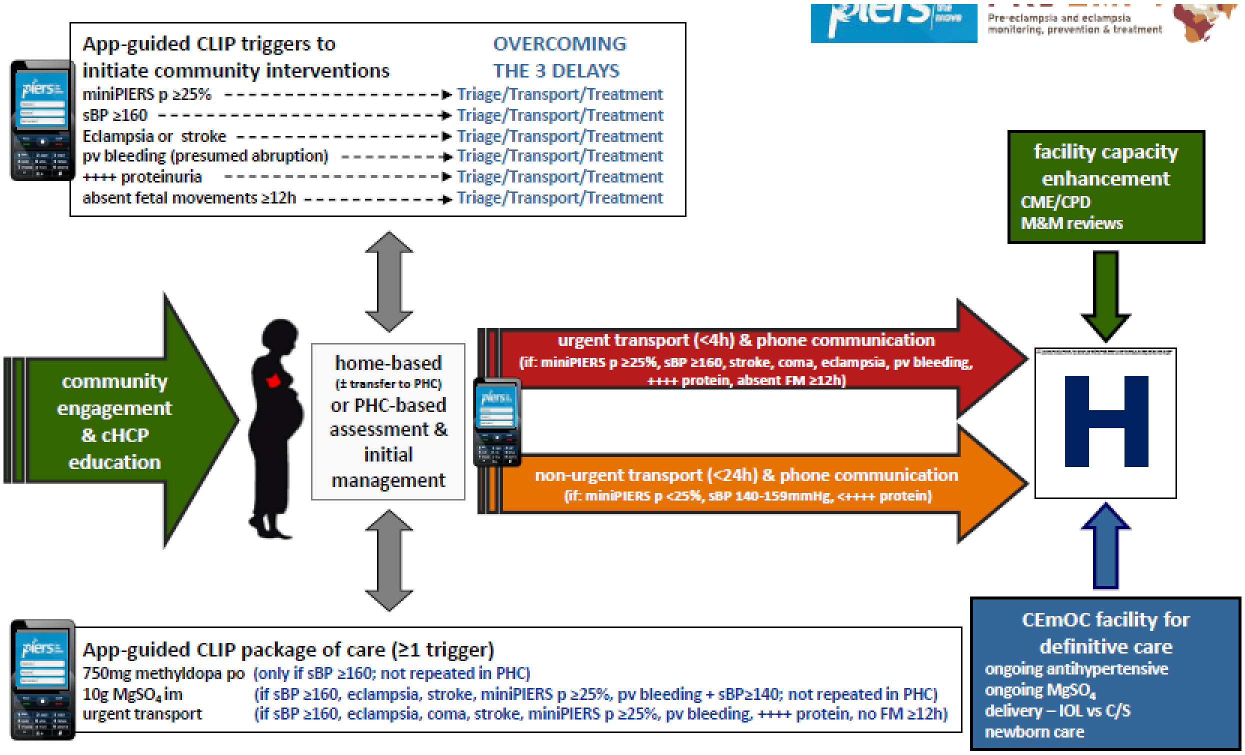

Figure 8: CLIP intervention diagram

CEmOC comprehensive emergency obstetric care, **cHCP** community health care provider, **CME/CPD** continuous medical education/professional development, **C/S** Caesarean section, **FM** fetal movements, **im** intramuscular, **IOL** induction of labour, **i.v.** intravenous, **M&M** morbidity and mortality, **p** probability, **PHC** primary health centre, **po** by mouth, **pv** vaginal, **sBP** systolic blood pressure

Component 1: Community engagement

The primary objective of the community engagement activities in CLIP will be to create awareness and action around the prevention of maternal morbidity and mortality due to pre-eclampsia/eclampsia. Community engagement involves the collective action of individuals, families, religious leaders, policy makers, health care providers and community members toward the creation of meaningful and sustainable change. Studies indicate that successful health behaviour change occurs when interventions create positive social, individual, and environmental conditions. Furthermore, community-based interventions that include women's and men's support groups, education, counselling, home visits, emergency transport initiatives and fundraising activities, have shown significant improvements in maternal and perinatal morbidity and mortality.

For each of the CLIP trials, a community engagement strategy will be developed with the participation of local stakeholders and community members that aims to be culturally and contextually appropriate, meaningful, sustainable, and effective in improving maternal health in CLIP intervention communities.

The first step is to determine what past and current activities related to community engagement around maternal health are occurring in control and intervention clusters. A specific tool has been developed for the purposes of identifying these community engagement activities so that the CLIP trial strategy can build upon them (Appendix C).

The second step follows the community mapping exercise. A community-specific engagement strategy will be developed, with direct input from community members, for the purpose of creating awareness and action around pre-eclampsia/eclampsia and the prevention of the associated maternal and perinatal morbidity and mortality, including but not limited to:

The CLIP community engagement activities must include (but are not limited to) the following nine topics:

1. Warning symptoms and signs of pregnancy complications, particularly pre-eclampsia & eclampsia
    - Relate the association of danger symptoms/signs with the occurrence of pre-eclampsia/eclampsia, using the warning symptoms of pre-eclampsia and eclampsia pictograms.
    - Identify the need for referral when danger symptoms/signs associated with pre-eclampsia/eclampsia occur.
    - Considering discussing postpartum haemorrhage (PPH), a 'visible' cause of maternal death, as a segway into discussion of pre-eclampsia/eclampsia as a 'silent' killer of pregnant/ postpartum women
2. Permission for women to seek care
    - Recognise the need for decision-making power and/or prior permissions in the event of obstetric emergencies.
    - Discuss how women can obtain prior permission to seek that care
3. Identification of skilled birth attendant
4. Identification of facility for delivery
5. Transport and treatment funds
    - Recognise the need to develop plans for financial resources when required in emergency conditions associated with pre-eclampsia/eclampsia. Funds may be personal or from the community The educator should the plan developed by individual communities.
    - Encourage the identification of existing community resources (if applicable) and the development of community funds for seeking emergency care. The community should be told that CLIP will supplement any existing funds, but fund-raising activities must build on those funds to make this sustainable
    - Identify available and appropriate modes of transport, the associated costs, and the means by which these modes can be accessed in emergencies.
6. Feedback mechanisms about adverse outcomes and 'great saves'
    - Country-specific CLIP Team could request that families of sufferers share their experiences with the community
7. CLIP protocol (brief)
    - The description should be brief, and focus on who is eligible and the CLIP intervention (of which community engagement is an important component)
8. CLIP visits and the CLIP triggers for treatment and transport (brief)
    - Review the frequency of the visits, assessments to be done as part of each visit, and the 'triggers' that will prompt the cHCP to recommend treatment and transport to facility.

9. Discussion about, and trouble-shooting designed to address, the barriers identified in the country-specific CLIP Feasibility Study.

Community engagement strategies used in the CLIP trial intervention clusters may take many forms and will be unique to the culture and context of each community as well as the community's resources and constraints. The CLIP team aims to utilise knowledge gained from an update to the Cochrane systematic review of the literature on community mobilisation in LMICs (being led by Susan Munabi-Babigumira), in co-ordination with the results of community mapping, and direct community input, to develop a community engagement strategy that is both evidence-based and tailored to the unique needs of CLIP intervention clusters.

It is recognised that sustainable community engagement activities more frequently involve an element of fund raising activity, than activities that are not sustained [35-38]. Therefore, the focus on transport and treatment fundraising activities is a singularly important element of the community engagement activity, and one that the CLIP Trial will pump prime funds so that every intervention cluster community has access to funds as the Trial commences to support the transport and care of women from the initiation of trial activity in that cluster. Maintenance and strengthening of that funding pool will be the responsibility of the intervention cluster communities

Component 2a: cHCP training in home-based maternity surveillance

We will develop an intervention package that involves the relevant main providers of primary maternal care in the community: LHWs (Pakistan), CHEWs (Nigeria), ANMs and ASHAs (India), and ACSs (Mozambique). These health care providers are termed cHCPs for this protocol. A description of the current scopes of practice in maternity care for these cHCPs is provided in Appendix A. In addition, in all countries, PHC-based health workers will be included in formal CPD activities provided in parallel to the CEmOC facilities to which women will be referred (see immediately below).

The cHCPs will be encouraged to identify pregnant women in their area, and will have pregnant women referred to them when identified by the surveillance team during regular surveillance cycles. The regular CLIP visits will occur approximately: 4-weekly during pregnancy until 28 weeks, fortnightly from 28-35 weeks and weekly thereafter, within 24 hours of birth, and on approximately days 3, 7, and 14 after delivery. 4-weekly visits over the course of a pregnancy in women enrolled in the study will be considered a minimum standard for exposure to this aspect of the intervention; anything less than 4-weekly will be recorded as non-compliance with the intervention. These visits are not meant to supplement regular ANC clinical visits, but will be performed in coordination with those existing programs to achieve the frequency as previously described. At each of those visits, the CLIP assessment will place women in one of three care trajectories: (i) usual antenatal care, (ii) non-urgent referral (within 24hr), or (iii) urgent referral (within 4 hr) to a CEmOC facility. Visits may occur in the woman's home or at the nearest PHC.

The CLIP intervention package (including the oral antihypertensive, $MgSO_4$ and all relevant safety and disposal devices) will be provided to the cHCPs in addition to incentives for the increased workload. Relevant incentives (e.g., monetary, gifts, or plaques) will be determined in conjunction with national site investigators.

For cHCP training in Pakistan, the enhanced education module will be developed as an adjunct to the regular LHW training programme; this module will be developed in collaboration with either the Sindh Ministry of Health or national Directorate of Health (depending on which entity has responsibility for the LHW programme after the current devolution to provinces is complete). In Nigeria, India and Mozambique, we will undertake parallel processes in collaboration with our local partners and relevant Ministries of Health.

This enhanced education module will include information about:

1. Understanding pre-eclampsia and eclampsia
2. Overview of the CLIP Trial
3. Basics of effective communication
4. Consent taking procedures
5. CLIP protocols
    a. CLIP materials
    b. CLIP visit schedule and protocols
        i. Counselling of women about:
            1. Warning signs of pre-eclampsia/eclampsia (involving use of pictograms)
            2. Advanced permission to seek care
            3. Transport plan
        ii. Proteinuria measurement
        iii. Blood pressure measurement using Microlife AS1-2 device
        iv. Estimating gestational age
        v. Assessment of warning signs of pre-eclampsia/eclampsia utilizing pictograms
    c. CLIP treatment
        i. Triggers for treatment
        ii. Administration of methyldopa
        iii. Administration of $MgSO_4$
        iv. Appropriate referrals, utilizing the referral form
        v. Recognizing adverse events
        vi. Triggers for treatment and appropriate medication administration
    d. Use of PIERS on the Move application
6. Contact Information

We anticipate that CLIP training will take an initial 2-3 days with an extra day for refresher training every six months or as needed.

Training of cHCPs will be evaluated at the initial training session, at each subsequent refresher session and throughout the CLIP Trial. Remedial action will be offered in the case of substandard performance with the evaluation. This evaluation will include pre and post assessment of both competence and self-efficacy on each training objective by means of demonstration of CLIP skills and completion of a survey.

2b: Diagnosis & triage – miniPIERS & CLIP POM

The final miniPIERS cohort used for analysis included 2081 women. The worst clinical values within the first 24hr after admission were used to develop the model. Variables included in the final miniPIERS model are parity (multiparity vs. nulliparity), gestational age on admission (or delivery if assessing a postpartum woman), the symptoms of chest pain and/or dyspnoea, headache and/or visual disturbances, vaginal bleeding with abdominal pain, and right upper quadrant pain; sBP; and dipstick proteinuria. The AUC ROC for this model was 0.6876868 (95% CI 0.37353 – 0.18011) which demonstrates a great ability to discriminate between women with and without adverse maternal outcomes. The stratification capacity of the model is good. Using a predicted probability cut-off of 25% resulted in a positive likelihood ratio of 5.09 [4.12, 6.29] and classified women with 85.000% accuracy, suggesting moderate utility of the model as a rule-in test for adverse maternal outcomes. The CLIP version of the PIERS on the Move tool (CLIP POM) integrates the miniPIERS predictive score and a clinical data collection system into a single application. cHCPs will assess women according to the visit protocol (Appendix D), entering clinical data into the CLIP POM mobile application. The application will provide recommendations for care according

to Figure 9 below, as per this protocol. Triggers identified that will indicate treatment and/or transport (urgently, defined as within 4hrs) to a CEmOC facility are as follows:

1. Unconsciousness ($MgSO_4$ if sBP ≥160 mmHg [to be reasonably sure that the unsciousness is associated with severe pre-eclampsia and not due to obstetric sepsis], urgent transport)
2. Signs of recent stroke or seizure (methyldopa if sBP ≥160 mmHg [to ensure BP is not lowered too much], $MgSO_4$, urgent transport)
3. Significant vaginal bleeding ($MgSO_4$ if sBP ≥140 mmHg [presumed abruption associated with severe pre-eclampsia], urgent transport).
4. No fetal movements felt in the previous 12 hrs (urgent transport [a threshold for identifying at risk fetuses that are alive at the time of screening] [39])
5. sBP ≥160 mmHg (methyldopa, $MgSO_4$, urgent transport [consistent with severe pre-eclampsia])
6. Heavy proteinuria (≥4+ by dipstick – predictive of stillbirth in miniPIERS cohort) (urgent transport)
7. miniPIERS predicted probability ≥25% ($MgSO_4$, urgent transport)

Non-urgent transport (by non-ambulance services), meaning assessment at a CEmOC facility within 24 hours, will be advised for all women with non-severe hypertension (sBP 140-159 mmHg) who do not meet criteria for one of the above 7 triggers.

The CLIP POM data and clinical decisions will be transmitted to the referral CEmOC facilities so that those centres can both: (i) provide support, advice, and feedback to cHCPs, and (ii) anticipate and prepare for the arrival of women who are referred with severe disease through automatic notification by SMS texting.

There will be *three models of referral* within the Pilot and Definitive CLIP Trials, depending on the country. In Nigeria and Pakistan, women, their families and communities will be responsible for identifying and paying for transport to the CEmOC facility unless the woman is found in critical condition. In these cases an emergency trial transport fund will be made available to ensure no undue harm comes to women found in critical condition during a CLIP visit. The related fundraising activity will be a focus of the community engagement, particularly in these two countries to ensure in non-urgent situations women can get to care. In Mozambique, women, their families and communities will be responsible for identifying and paying for transport to the nearest PHC, where i.m. $MgSO_4$ will be administered (if relevant) and where women will be transferred by ambulance (free service provided 24/7) to a CEmOC facility. In Belgaum and Bagalkot Districts, Karnataka, India, there is a functioning ambulance service available free of charge 24/7. Women will be taken from their home or local subcentre (if ANM not available to go to their home) directly to the CEmOC facility.

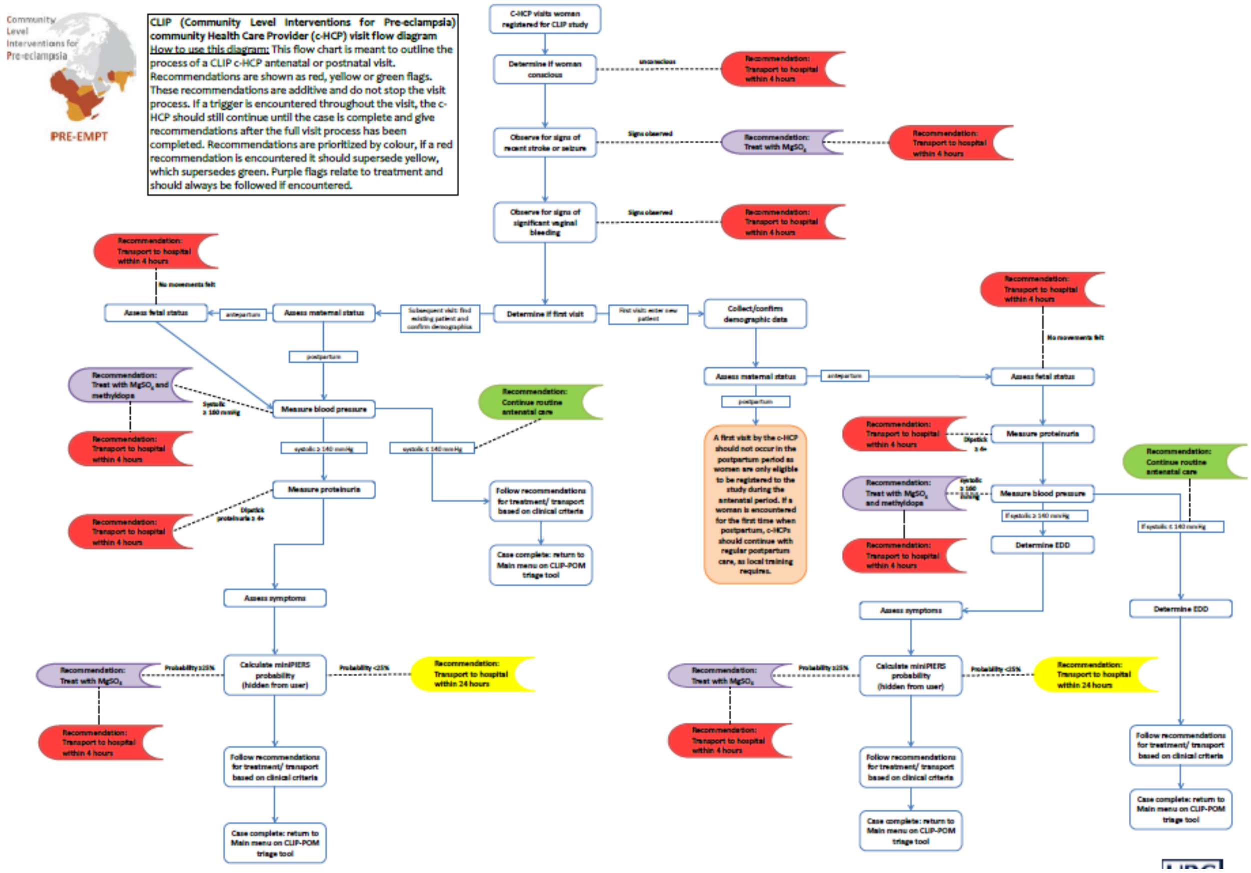

Figure 9 CLIP POM decision model for use in the mobile application for the CLIP trial

2c: Lowering severe hypertension – oral methyldopa

In the absence of a clearly preferred oral agent, we have opted for ***methyldopa 750mg***. Our rationale is that oral methyldopa has been well-studied in pregnancy hypertension RCTs, and although possibly less effective than some agents[5], is less likely to cause hypotension[5], has no common medical contraindications, and can be administered orally[5]. Also, methyldopa is readily available on nearly all LMIC essential medicines lists and it is cheap and acceptable for use by most practitioners [Lalani *et al. JOGC*]. As shown in the decision tree (Figure 9), methyldopa will be administered to women when:

- sBP is ≥160 mmHg, or

Women will not receive further methyldopa until they reach a CEmOC facility. As part of facility enhancement, personnel at CEmOC facilities will be educated about how to deal with those women enrolled in CLIP who are transported to facilities. As part of these enhancement activities described below, we will encourage facility staff to provide further antihypertensive therapy if at least 40 minutes has passed and sBP has not started to decrease; if so, clinicians will be advised to wait and monitor BP until the peak effect of methyldopa occurs (between 3 and 6 hr). Otherwise, additional antihypertensive therapy (such as oral nifedipine or i.v. hydralazine) may be administered.

2d: Preventing & treating eclampsia – intramuscular $MgSO_4$

$MgSO_4$ is the treatment of choice to prevent and manage eclampsia in high, middle, and low income countries [3;40-43]. In the International Eclampsia and Magpie Trials, the loading dose was either: (i) 4g i.v. given over 10-15 minutes plus 10g i.m. (followed by i.m. maintenance therapy of 5g i.m. every 4 hr), or

(ii) 4g i.v. (followed by i.v. maintenance therapy at the rate of 1g/hr).

We have elected to use only ***10g $MgSO_4$ via the i.m. route***. Our rationale for this is as follows. First, the choice of a 10g loading dose is supported by the WHO (Integrated Management of Pregnancy and Childbirth, 2009), where, in the absence of i.v. access, the expert committee advised loading with only 10g i.m. of $MgSO_4$[44]. Second, the use of i.v. medication by cHCPs is not feasible and the i.m.-only regimen results in therapeutic serum Mg^{2+} concentrations by 45 minutes after i.m. administration [33]. Third, although there are limited RCT data for this regimen, there is substantial regional experience with i.m. loading dose administration in the community (Hall & Theron, personal communications) as well as with 10g total loading dose regimens[45-51]. Professors Hall and Theron have over 10 years' experience with community i.m. $MgSO_4$ loading in the Cape Flats, Cape Town, South Africa, without apparent maternal harm. In Shahjadpur, Bangladesh, the ICDDR,B community-level (community skilled birth attendants) intervention for women with eclampsia includes a total 10g loading dose (4g i.v. and 6g i.m.) based upon the Dhaka protocol [45;46;52;53]. Currently, in our study areas in India and Pakistan, ANMs and LHWs areadministering i.m. dexamethasone within the context of an RCT (clinicaltrials.gov/ct2/showNCT01084096 [accessed 28 Jan 2013]) which shows capacity for skill training in this area.

Based on the approach used in the successful Eclampsia and Magpie Trials, we will develop '***pre-eclampsia boxes***' for use by cHCPs. These boxes will include single doses of oral methyldopa (750mg) and i.m. $MgSO_4$ (10g in two 5g vials of 50% solution), along with all necessary supplies and disposal measures.

Women will not receive further $MgSO_4$ until they reach a CEmOC facility. There, they will receive a further dose according to local protocols. As part of facility enhancement, personnel at CEmOC facilities will be educated about how to deal with those women enrolled in CLIP who are transported to facilities. Through these enhancement activities, described below, we will encourage standardised dosing regimens in line with international guidelines such as 2g i.v. $MgSO_4$ over 10-15 minutes for recurrent eclampsia (that occurred en route or at facility). Through CPD activities, we will suggest optimal treatment protocols for CLIP women referred to facilities. Following administration of 10g i.m of $MgSO_4$ in the community:

- Women will not receive further $MgSO_4$ therapy until they have reached a CEmOC facility
- At the CEmOC facility, these women will receive more $MgSO_4$ ONLY after at least 4 hr have passed since their initial 10g i.m $MgSO_4$ loading dose given in the community.
- If women arrive at the CEmOC facility 4-6hr after receiving a 10g i.m $MgSO_4$ loading dose in the community, these women should receive $MgSO_4$ maintenance therapy, consisting of either: (i) 5g i.m. every 4hr, or (ii) 1g/hr i.v.
- If women arrive at the CEmOC facility >6hr after receiving a 10g i.m $MgSO_4$ loading dose in the community, these women should receive another loading dose of $MgSO_4$ following by maintenance therapy, as follows: (i) 10g i.m. plus 4 g i.v. loading dose, followed by 5g i.m. every 4 hr as maintenance therapy, or (ii) 4g i.v. loading dose, followed by 1g/hr i.v. maintenance therapy.

Component 3: Facility enhancement

We will undertake enhancement activities at facilities able to provide EmOC, to ensure a minimum standard of care will be provided to all inpatient women in both intervention and control clusters. In addition, although health workers based in PHCs are care providers at the community level, their training and experience mandates that they receive their CLIP-related training within the context of facility-based continuous professional development.

In India and Pakistan, EmOC facilities may be shared by intervention and control clusters, whereas they are

distinct in Nigeria. This detail is yet to be determined for Mozambique.

Facility enhancement activities will be undertaken in collaboration with local and international partners (e.g., MCHIP, RCOG, SOGC). The objectives of facility enhancement are to:

1) Provide facilities with ***general HDP-related educational resources***, including WHO guidelines on pre-eclampsia and eclampsia [34], textbooks, and posters.
2) Provide health providers with ***special clinical training in basic and intermediate care of women with HDP***
   a. CLIP protocol (including nature of cHCP CLIP visits with respect to counselling provided, tasks performed, and medications administered) and how to appropriately manage these women once they reach a facility
   b. Warning signs of pre-eclampsia/eclampsia
   c. BP measurement
   d. Proteinuria measurement
   e. miniPIERS assessment on admission
   f. Management of severe hypertension
   g. Management of severe pre-eclampsia and eclampsia, including $MgSO_4$ and timing of delivery
3) Enhance health provider ***respectful communication skills*** with women, their families, and other maternity care providers
4) Inform ***adverse outcome identification*** related to the CLIP intervention. Through this mechanism, we will use facility-level (and, therefore, more accurate) information for all women in the CLIP Trials to assess the accuracy of data collected through the household surveillance approach. This comparative analysis will be important as a validation exercise should the planned difference in adverse outcome rates be achieved.

2.3 RISKS TO THE SAFETY OF PARTICIPANTS

The main risk of the CLIP intervention is that it may lull cHCPs, families, and the women themselves into a false sense of security, thereby delaying the initiation of transport for definitive and life-saving treatment. It is for this reason that the CLIP package of care will include education about appropriate transport to facility, as well as instructions about how quickly that transport should occur.

For methyldopa safety, we will identify the percentage of women with an 'out of target' BP on admission to facility (i.e., sBP ≥160mmHg indicating lack of effect, or sBP <110mmHg indicating excessive effect).

$MgSO_4$ toxicity is not a major concern. Mg^{2+} is renally cleared but even if a woman were anuric, one 10g i.m. loading dose of $MgSO_4$ would not result in serum Mg^{2+} concentrations sufficient to cause neuromuscular blockade[54]. However, given that repeat doses would have the capacity to do so, no repeat doses of i.m. $MgSO_4$ will be given in the community by cHCPs (or at a PHC with only BEmOC capabilities), even if the indication for therapy persists. Practically, we will assess $MgSO_4$ injection site haematomas, infections and pain using standardised assessment tools in women who received the particular intervention. cHCPs will be trained in proper methods of administration of i.m. injections and all women who receive an injection will be referred to a local facility for follow-up in order to minimize any risks related to these side effects

2.4 Method of randomisation

Clusters

The unit of randomisation will be defined by criteria relevant to each setting.

In Nigeria, the unit of randomisation will be Local Government Areas (LGAs). Each has a Department of Health headed by a Medical Officer of Health. All villages and PHCs within the LGA will be included in that cluster.

For the pilot phase, we will include four LGAs.

For the definitive phase, we will include 10 (5 intervention and 5 control) clusters in the trial with a public and private sector referral facility in the Ogun area. The four pilot trial clusters will remain in their assigned groups.

In Mozambique, the unit of randomisation is the Administrative Post. All villages and PHCs within the unit of randomisation will be included in that cluster.

There will be no Pilot phase in Mozambique. For definitive phase, we will include 12 (6 intervention and 6 control) clusters in the trial with a public and private sector referral facility in the provinces of Maputo and Gaza.

In Pakistan, the unit of randomisation will be defined by their union council. The villages and PHCs within that union council will be included in the cluster. We will recruit in the catchment area of adjacent union councils of Hyderabad and Matiari, Sindh, Pakistan.

For the pilot phase, we will include four union councils.

For the definitive phase, we will include 20 (10 intervention and 10 control) clusters in the trial with a public and private sector referral facility in the area adjacent to Hyderabad and Matiari (the latter to give patients choice in terms of referral options beyond the rural health centres (RHCs)). The four pilot trial clusters will remain in their assigned groups.

In India, the unit of randomisation will be the PHC. All villages within the PHC catchment area will be included in that cluster.

For the pilot phase, we will include four PHCs.

For the definitive phase, we will include 12 (6 study and 6 control) clusters in the trial with a public and private sector referral facility in the area adjacent to Belgaum and Bagalkot. The four pilot trial clusters will remain in their assigned groups.

Figure 10 Trial organisation by country and clusters
cHCP community health care provider

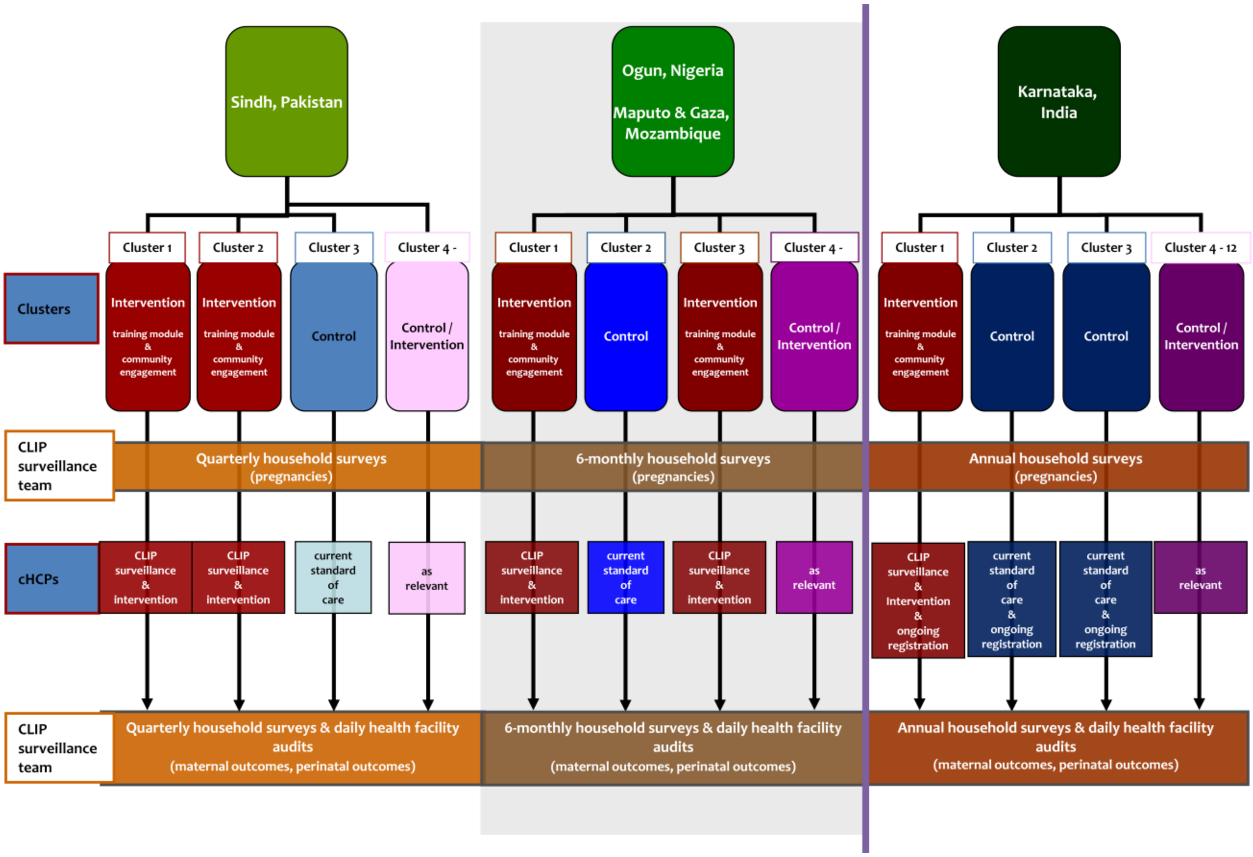

To ensure reasonable balance between the two groups, we will use restricted, stratified randomisation to allocate clusters to the intervention and control groups [12].We will be using only one continuous stratification variable for the randomization of clusters in each country. For all countries this will be Population size. The randomization algorithm ensures adequate balance on the stratification variable over the Intervention arm and the Control arm. Allocations that fail any of the balance criteria will be excluded, and only allocations that meet all criteria are eligible for consideration. The final allocation scheme is a random selection from the list of eligible allocations.

The current algorithm is implemented using R Programming and can consider the following balance criteria:

1. Means ratios: the mean value over intervention clusters and the mean value over control clusters have a ratio no less than 0.9 and no greater than 1.1 (10%).

2. Mean relative to overall mean: the overall mean is the mean over all clusters; mean value in each arm is within 10% of overall mean.

3. Absolute difference in sums: the absolute difference between sums in the two arms is within 10,000.

4. Wilcoxin rank sum: Allocations where the Wilcoxin rank sum test have p-value <0.01 will be excluded.

5. Kolmogorov-smirnov test: Allocations where the Kolmogorov-smirnov test have p-value <0.01 will be excluded.

In the pilot phase of the trial we randomize 4 clusters per country, except Mozambique where no Pilot phase is occurring. When we approach the definitive phase of the trial, these 4 clusters will be included in the randomization algorithm together with the new clusters, and their previous allocations will serve as an additional requirement for the algorithm. The set of eligible allocations will be those that meet the list of balance criteria and the pilot-allocation requirement.

We may or may not use all of the balance criteria listed, and the cut-points listed may be modified. These are common criteria and cut-points used in the literature, but adjustments may be needed if the resulting number of eligible allocations is deemed inadequate.

At the stage of analysis other cluster level factorsidentified as having an impact on the outcome will be adjusted for, including those used for stratification, such as (i) cHCP density by cluster, (ii) population density, (iii)distance to referral facilities (public or private), (vvi) home birth numbers, (v) skilled birth attendant numbers, and (vi) mortality data. Once the decision has been made to proceed to the Definitive Trial phase the population numbers will be re-confirmed for all remaining non-Pilot clusters and the stratification and randomization process will be performed as described above. Clusters participating in the Pilot Trial phase will not be re-randomised for the Definitive Trial phase but will retain their Pilot Trial phase designation of intervention or control.

2.5 Protecting against bias

We will *not* undertake a hospital level implementation trial, but rather, focus on community-level (i.e., cHCP and PHC) interventions. It is at those levels that the burden of disease-related risk lies. However, informed by the results of the Feasibility Study in each country, we will have determined that, at first level inpatient facilities (BEmOC) and the CEmOC facilities, both: (i) effective antihypertensive therapy is used in >80% of women with severe hypertension (sBP ≥160mmHg and/or dBP ≥110mmHg), and (ii) $MgSO_4$ is on formulary and used in >90% of women with eclampsia. Ensuring a standard and consistent level of care across both intervention and control clusters will reduce any bias that may have existed due to variability in facility based quality of care at study baseline.

Specifying and selecting study clusters

No cluster will have participated (or be currently participating) in either antenatal intervention trials or community mobilisation.

Execution of the intervention

Contamination bias

Communities in control clusters will not be engaged in any community engagement activities.

cHCPs in control clusters will receive no additional training about pre-eclampsia/eclampsia diagnosis or management, or the CLIP protocol. At present, cHCPs in these jurisdictions do not measure both BP and proteinuria and in Pakistan, they measure neither. cHCPs in control clusters will not be taught how to measure BP or proteinuria because their measurements are components of the miniPIERS risk stratification tool, an important element of the CLIP intervention. cHCPs in control clusters will have access to neither the mHealth tools or the CLIP intervention package.

There is no risk of contamination at the CEmOC facility level as both intervention and control clusters will be engaged in capacity enhancement activities at CEmOC facilities in order to ensure that women who are transported to a CEmOC facility with pre-eclampsia/eclampsia will receive standardised care. This is an

important element as it is anticipated that the CLIP intervention will result in more women sent to facility, because of diagnosis and treatment of pre-eclampsia/eclampsia that either: would not have been otherwise made (and could have resulted in death or morbidity that went unrecognised because they did not go to facility), or would have been made at a later stage of their disease (at which point these women would have been transferred to facility). PHC workers in the intervention clusters (not those in control clusters) will be educated within the BEmOC and CEmOC capacity enhancement.

Compliance bias

We will assess cHCP compliance with administration of the intervention using the mHealth technology.

Measurement bias

We will reduce the risk of measurement bias by using measurement tools tested for validity and reliability during the CLIP Feasibility Studies or by others in unrelated studies (Appendix D).

BP measurement will be standardised through use of a standard protocol and an automated measurement device (Microlife BP 3AS-2).

2.6 Inclusion / exclusion criteria

Participants will be ***all consenting pregnant women** aged 15-49 (except in Mozambique where reproductive age begins at 12)* identified by the cHCPs assigned to their community. No such pregnant women will be excluded from the analysis which will be by intention-to-treat.

In Nigeria and Mozambique, all women in both control and intervention clusters will give verbal or written consent for the entire CLIP trial. In India, consent will be written. In Pakistan, all women in both control and intervention clusters will give: verbal consent to participate in the quarterly household surveillance, and written consent (personal or proxy [if obtunded]) for administration of the intervention package, if indicated.

2.7 Proposed duration of treatment

The ***community engagement*** aspect of the CLIP intervention will be applied from the time of randomisation of the clusters until completion of recruitment to the Trial.

The ***provision of HDP-oriented antenatal care through CLIP visits and use of the CLIP POM tool***, as well as use of the ***CLIP package for women with a 'trigger'***, will be applied from the identification of each woman's pregnancy (and her consent to participate in CLIP), through transport to an CEmOC facility (as indicated), and until six weeks postpartum. From the time of diagnosis of a CLIP trigger consistent with pre-eclampsia, women may remain pregnant for an average 10-14 days depending on the gestational age at diagnosis [1]. After diagnosis all women are referred to care at a local facility where they will receive the standard of care in place at that facility. The standard that we will promulgate through our facility enhancement rounds will specify that induction of labour (or semi-elective Caesarean if obstetrically indicated) will be offered routinely to women at or beyond 37^{+0} weeks' gestation [55].

2.8 Frequency and duration of follow-up

Clinically, women will be followed up by cHCPs up to 14 days postpartum. In addition, women will be followed until 42 days after their delivery by CLIP research staff. In all clusters, this research follow-up will be by means of regular household survey over the 18 months of the trial (i.e., baseline and 9 follow-up surveys) and daily facility audit (as outlined in Figure 10).

2.9 Outcomes

- ***Pilot CLIP Trials***

The ***primary outcome*** for the Pilot CLIP Trials will be common for all participating countries, and will be appropriate use of the CLIP package of care (methyldopa and/or $MgSO_4$ and urgent/non-urgent transport to CEmOC facility) in women with the appropriate CLIP trigger. To justify continuing on with the Definitive CLIP Trial, by 8 months into the 12 month Pilot (with the exception of India were assessment will occur at 6 months), at least 50% of eligible women (i.e., with a trigger) should have received the package of care.

The Pilot CLIP Trials will recruit for 12 months, primarily to field test: application of the CLIP intervention, data collection, and research infrastructure through four quarterly surveillance cycles (in Nigeria and Pakistan).

The ***secondary outcomes*** for the Pilot CLIP Trials will be the adverse maternal and perinatal outcomes described below for the Definitive CLIP Trials.

The ***other outcomes*** will be the secondary and other outcomes described below for the Definitive CLIP Trials.

As the primary outcome will change between the Pilot and Definitive CLIP Trials, women recruited to the Pilot Trial will be included in the total sample analysed for the Definitive Trial.

- ***Definitive CLIP Trials***

The ***primary outcome*** will be a combined maternal and/or perinatal outcome (either maternal, fetal or neonatal death, or severe morbidity for the mother or baby). We have chosen a combined maternal and/or perinatal outcome as powering a trial for maternal death alone would require a prohibitively large sample size.

Maternal death (defined as the number of deaths during pregnancy or within 42 days of pregnancy (or last contact day if contact not maintained to 42 days) / 1,000 identified pregnancies), termed Maternal Death Rate. We have not chosen the classical WHO maternal mortality ratio (MMR) definition of events per 100,000 live births because we hope that the CLIP intervention will reduce stillbirth in women with pre-eclampsia. If so, the MMR per 100,000 live births would fall artificially as the proportion of live born infants increased, without any absolute change in the maternal adverse event rates (per pregnancy). We will be basing the number of pregnancies on the household surveys; although the timing of public declaration of pregnancy varies between cultures (first trimester in India and up to 20-24 weeks in Pakistan, Nigeria, and Mozambique), the manner of identifying pregnancies will be similar between control and intervention clusters in each country.

Maternal morbidity (defined as the number of women with one or more life-threatening complications of pregnancy during pregnancy or within 42 days of pregnancy or last contact day if contact not maintained to 42 days) / 1,000 identified pregnancies). These are the serious end-organ complications of pre-eclampsia, other major causes of maternal mortality, or life-saving interventions related to one of the aforementioned:

Serious end-organ complications of pre-eclampsia:

- *Eclampsia:* occurrence of generalised convulsions during pregnancy, labour or within 42 days of delivery in the absence of epilepsy or another condition predisposing to convulsions
- *Stroke:* hemiparesis and/or blindness developed during pregnancy or in the 42 days postpartum lasting greater than 48 hours
- *Coma:* prolonged unconsciousness ≥12 hours

- *Antepartum haemorrhage:* vaginal bleeding ≥ 15 mL with or without pain before the onset of labour
- *Disseminated intravascular coagulation* (DIC): abnormal bleeding from mucosa(mouth and/or ears)

Other major causes of maternal mortality:

- *Obstetric sepsis:* In the community, defined as fever and one of: abdominal/uterine tenderness, foul smelling vaginal discharge/lochia, productive cough and shortness of breath, dysuria or flank pain, headache and neck stiffness. In the facility, defined as presence of fever (>38°C), a confirmed or suspected infection (e.g., chorioamnionitis, septic abortion, endometritis, pneumonia) and at least one of the following: heart rate >90/min, respiratory rate >20/min, leukopoenia (total leukocyte count [TLC] <4 x 10^9/L) or leukocytosis (TLC >12 x 10^9/L)
- *Vesicovaginal or rectovaginal fistula:* continuous loss of urine and/or faeces after delivery

Life-saving interventions:

- *Cardiopulmonary resuscitation:* a set of emergency procedures including chest compressions and lung ventilation applied in cardiac arrest victims
- *Dialysis:* haemodialysis and/or peritoneal dialysis
- *Mechanical ventilation* (other than for Caesarean section): intubation and ventilation not related to anaesthesia
- *Blood transfusion:* ≥1 unit
- *Interventions for major postpartum haemorrhage:* brace sutures, external and internal uterine compression, anti-shock garment use, internal iliac artery ligation and/or hysterectomy with or without transfusion

Perinatal & late neonatal death (defined as stillbirth [≥20^{+0} and/or ≥500g], early neonatal mortality [d 0-7 of postnatal life] and late neonatal mortality [d 8-28 of postnatal life] / 1,000 identified pregnancies), or

Neonatal morbidity (defined as non-lethal events of seizure and coma[30] during d 0-28 of postnatal life / 1,000 identified pregnancies). The following are the primary neonatal morbidities:

- *Feeding difficulty*
- *Breathing difficulty*
- *Seizure*
- *Lethargy*
- *Coma*
- *Fever*
- *Hypothermia*
- *Umbilical cord infection*
- *Skin infection*
- *Bleeding*
- *Jaundice*
- *Vomiting/diarrhoea*

Secondary outcomes for the Definitive CLIP Trials have been defined to measure the impact of the CLIP intervention on the delays around triage and transport. The effect of the CLIP intervention on treatment delays will be determined by the primary outcome and does not need further evaluation as a secondary outcome.

<u>Birth preparedness and complication readiness</u> as measured by any three of the following: (1) arranged for transport; (2) obtained prior permission for transport should emergency arise; (3) saved money for obstetric care; (4) identified skilled birth attendant; (5) identified facility for delivery. This will evaluate the success of community engagement.

<u>Number of women presenting for care at a CEmOC facility</u> and <u>number of facility births</u>: This number should increase if triage (using the miniPIERS risk stratification and POM) is effective, and if there is compliance with transport to CEmOC facility.

Other outcomes will be evaluated such as knowledge of pre-eclampsia/eclampsia among women of reproductive age; total number of seizures; number of pre-admission and post-admission seizures; adverse events; functional disability; and cost-effectiveness of the CLIP intervention. In addition, other outcomes for the perinatal morbidities are congenital anomaly and birth injury.

Functional disability for any reason: Inability to perform usual daily duties at specific points in time during the postpartum period that would be acceptable and expected culturally (we are determining these normal limits through local focus group discussions; these will be defined for each country prior to the commencement of each Pilot CLIP Trial)

2.10 Measuring the outcome measures at follow up

In total, 54 village and urban clusters will be identified and randomised in the four countries (Nigeria: 10; Mozambique: 12; Pakistan: 20; India: 12). Each cluster will contain a health unit or regional health centre which has provided the training base for the corresponding cHCPs. Subsequently, the 27 clusters chosen to receive the intervention will be analysed against 27 control clusters.

A ***baseline household and facility survey*** is being carried out within the context of the CLIP Feasibility Study in all trial clusters to assess their socioeconomic characteristics and baseline maternal, perinatal, and neonatal mortality rates. In all countries except India this will be performed based on retrospective information on pregnancies occurring in the previous year. In India, the baseline data will draw from the existing prospective Maternal and Neonatal Health Registry (MNH Registry). In Nigeria, due to the large cluster populations we will perform baseline surveillance on a random sample of households while in all other countries all households will be surveyed. This addresses the human resource constraints identified in Nigeria during the CLIP Feasibility study.

Subsequent ***cross-sectional surveys of all households*** will be conducted at varying frequency depending on current registration and surveillance activities in the relevant country: quarterly (Pakistan), 6-monthly (Nigeria and Mozambique), while in India, household surveys will continue prospectively using the MNH Registry. Although the cHCPs will routinely record information on maternal/perinatal births and deaths, the cross-sectional surveys will be conducted by a separate team to collect data on births, deaths, and care-seeking behaviour in the preceding study epoch. In Nigeria, due to population sizes and human resource constraints the cross-sectional surveys will focus on collection of maternal/ perinatal mortality and morbidity for all households and a random sample of these households will receive a more indepth survey including questions around birth preparedness, care-seeking and pre-ecalmpsia knowledge. The cross-sectional surveys will be considered the gold standard for the Trial as they will be applied equally between control and intervention clusters.

These surveys will permit us to accrue outcome data about women who had a CLIP primary outcome but who did not seek care at a CEmOC facility. These data will be necessary for us to undertand fully the impact of the CLIP intervention on mothers and babies. It is our hope that the CLIP intervention will cause

women from the intervention clusters to be treated earlier in the course of their disease and, as a result, have less complicated clinical courses and lower rates of severe morbidity. As such, fewer women may die unknown to the formal health care system but more such women may be referred to the PHC and both BEmOC and CEmOC hospital levels. If that referral occurs in a timely fashion, then it is our hope that women will receive more timely, evidence-based, care (given facility enhancement). As such, it is hoped that overall, maternal and perinatal mortality and morbidity will be decreased. However, it is plausible that despite identification, triage, community treatment, and transport of women with a CLIP trigger, and despite EmOC facility enhancement about pre-eclampsia/eclampsia care, the number of inpatient pre-eclampsia-related maternal and perinatal deaths may rise if women and/or fetuses who previously would have died in their community might now reach hospital moribund and beyond help.

Information on referrals will be collected from the cHCPs and community health committees by the surveillance teams. Surveillance teams will carry out ***verbal and social autopsies of maternal deaths, stillbirths, neonatal deaths***, and similarly constructed reviews of maternal and neonatal morbidity.

In the event of significant barriers to application of the CLIP package or unforeseen events, focus group discussions and/or in-depth interviews and/or surveys will be conducted to better understand relevant barriers or events, in an effort to remedy the situation. Case analysis may also be employed on a select number of maternal or perinatal morbidities or mortalities to uncover greater detail of context of the outcome.

A final aspect of follow-up will be ***facility-based chart audit*** of pregnant women admitted to CEmOC facilities in both intervention and control clusters, throughout the Trial will occur in all but India where the MNH registry system already incorporates a facility chart review for each registered woman. This will allow for triangulation of mortality and morbidity data collected through the the cHCPs and cross-sectional household surveys.

Data collection using mHealth technology

We will develop three mHealth applications to support the CLIP trial. The first will be used in all countries and the second and third will be used in Nigeria, Mozambique and Pakistan during the definitive trial phase. The first application will be mobile phone-based while the other two will use a tablet-based device.

For intervention clusters only, the first mHealth application is the CLIP POM (described in detail, previously). This will be used to collect routine antenatal visit data and miniPIERS variables. These data will be entered into an integrated decision guide to give real-time advice to cHCPs in terms of appropriate patient management and decisions for interventions (e.g., appropriateness and timing of transport to CEmOC facility, and community initiation of antihypertensive and $MgSO_4$ therapy, as discussed above). Women will be identified both by demographics information and by unique surveillance IDs to enhance identification through the system.

For both intervention and control clusters, the second mHealth application will be a tablet device used by the field team for routine data collection during household surveys (see above). In this way, survey data will be automatically synchronised with a centralised data warehouse, and collected efficiently (given no double handling of paper forms and efficient enforcement of complete data entry). GPS co-ordinates will be provided in real-time to confirm the place and time of each survey event.

For both intervention and control clusters, the third mHealth application will be a tablet device used by an independent team of skilled interviewers who will conduct a detailed verbal and social autopsy / morbidity review when the quarterly surveys reveal the occurrence of an adverse maternal and/or perinatal event (death or major morbidity). The application will be adapted from that developed by the WHO.

Each CLIP site will participate in the design and development of mHealth tools and will conduct usability testing of the final application prototypes before the trial begins in order to ensure acceptability and usability of the tools by users (cHCPs and surveillance teams).

Data on individual women entered into all mHealth applications will be linked using a unique identifier code. This code will be provided to each woman after they consent for the trial on a study ID card. The key for these unique identifiers will be developed after the baseline survey by the Data Manager in each country and will not be shared with the Trial Coordinating Centre at UBC, ensuring privacy and confidentiality of the women is maintained at all times.

Patient data security is at all times of utmost importance, and will thus be maintained throughout mHealth system operations.

- Valid usernames and passwords will be used to limit unauthorized access to device data as well as access to the database and central document archives. The relevant password rules and encryption protocols will be applied.
- Encryption of data is required for any form of storage or transmission between devices and systems.
- Data validation will be performed at every stage of data input as well as on all data access requests to minimize false scripting.

In addition, all safety regulations pertaining to medical diagnostic and monitoring tools will be upheld. Any interfaces between patient, device and user will have the necessary protection systems to ensure the safety of the patient and operator.

2.11 Sample size calculations

Each country is independently powered to assess the chosen primary outcome. The data upon which the estimates have been made are routinely at the conservative end of the published or available range, and were generally provided by the site investigators. However, these calculations will be revisited once the CLIP Feasibility Study maternal and perinatal morbidity and mortality data are in hand. We have planned an individual patient data (IPD) meta-analysis at the completion of each of the individual country trials to ensure adequate power for an analysis of maternal mortality and morbidity.

Table 3 Sample size calculations (Mozambique to be determined)

Country	Nigeria	Mozambique	Pakistan	India
**Unit of randomization**	LGA	AP	Union Council	PHC
**Population per unit of randomization**	70,000	25,000	32,000	27,000
**Annual birth rate (/1000/yr)**	16	40	14	22
**Births/2yr**	2240	2000	900	1200
**MMR (/100,000 live births)**	800	388	267	150
**Intra-cluster co-efficient**	0.006†	0.002‡	0.002*	0.001**
**Incidence of maternal & perinatal/neonatal M&M**				
**control clusters**	28.8%	14.0%	9.6%	5.4%
**intervention clusters**	23.0%	11.1%	7.7%	4.3%
**Number of clusters (total)**	10	12	20	12
**Number of births in Definitive CLIP Trial (2 years)**	22,400	24,000	18,000	13,000
**Additional births from Pilot CLIP Trial (4 clusters/1 year)**	4480	nnn/a	1800	2400
**Total number of births (Pilot & Definitive Trials)**	26,880	4244,000	19,800	19,200
**Expected referrals at month 6 of Pilot Trial assuming 5% incidence of HDP**	112 [92, 132]	100 [81, 119]	45 [32, 58]	60 [45, 75]
**Number of women to be referred at month 6 of Pilot Trial to meet 50% use target**	56	50	23	30
**Number of health facilities (total)**		TBD		
**PHC/RHC**	120		42	22
**EmOC**	10			22
**Number of cHCPs (total)**	250 CHEWs	TBD	400 LHWs	60 ANMs & 360 ASHAs

ANM: assistant nurse midwife; **AM: Administrative Posts; ASHA**: accredited social health activist; **CHEW**: community health extension worker; **cHCPs**: community health care providers; **CHW** community health worker; **EmOC**, emergency obstetric care; **LGA**: Local Government Area; **LHW**: Lady Health Worker; **M&M**: morbidity and mortality; **MMR**: maternal mortality ratio; **PHC**: Primary Health Centre; **RHC**, rural health centre.

* calculated from Sindhi miniPIERS facility data. † assumes 3-fold increase in ICC over Sindh; ‡ assumes same ICC as Sindh (urban population in Maputo); ** assumes half the ICC of Sindh. In these calculations, we have used risks at the lower end of the published ranges for that risk and milieu.

Assumptions
- 10% loss of individual women to follow-up
- no loss of clusters to follow-up
- alpha of 0.05, power ≥0.80
- anticipated effect size of a 20% reduction in all cause maternal and perinatal/neonatal morbidity and mortality
- Also, we have assumed a ratio of maternal morbidity : mortality of 5:1 (may be as high as 10:1), and a ratio of perinatal/neonatal : maternal events of 5:1 (may be as high as 10:1 – permits overlap in outcomes)
- All sample sizes will be supplemented by the data collected in each single country Pilot CLIP Trial.

The combined Definitive CLIP Trials cohort of 878787,480 deliveries in 56 clusters over 2 years (+16 cluster years in Pilot CLIP Trial), averaging 1376 deliveries per cluster per year, will provide the following power for the IPD meta-analysis:

Maternal & perinatal/neonatal mortality & morbidity: assuming a 20% effect size (incidence reduced from 15.6% to 12.4%), we would have 80% power with an ICC of 0.015.

Maternal mortality & *morbidity* (primary outcome for the IPD meta-analysis): assuming a 20% effect size (incidence reduced from 2.6% to 2.1%), we would have 80% power with an ICC of 0.001. Alternatively, assuming a 30% effect size (incidence reduced from 2.6% to 1.8%), we would have 80%

power with an ICC of 0.004. We hope to reduce the adverse maternal event rate by ≥30%, primarily through community mobilisation and antenatal care by cHCPs.

Finally, we will pool adjusted results for the individual CLIP Trials using the generic inverse variance method developed by the Cochrane Collaboration.

This sample size will be supplemented by the women recruited during the pilot phase (primary outcome: package utilisation), who will be 'recycled' into the Definitive Trial phase.

2.12 RECRUITMENT RATE

Given the populations in these centres and the fertility rates in these communities, we are confident that the sample size will be achieved in a 24 month period (see section 2.11, above) and the Trials will complete recruitment by March 2017. We will pilot the use of *contact tools* to maintain contact, with quarterly contact maintenance rate reports (e.g., birthday cards and newsletters). Thus, we will maintain a group of mothers and children available for piloting further assessment of the impact of the CLIP intervention at school age (funding for follow-up studies will be sought).

2.13 COMPLIANCE WITH THE CLIP INTERVENTION

Through the trial, we will measure ***compliance with community mobilisation*** in terms of the number of community meetings held around pre-eclampsia/eclampsia content, prior permission to seek care, and/or community transport plans/funds.

Throughout the trial period, we will measure ***compliance with the CLIP visits, use of the POM tool, and use of the CLIP package for women with a CLIP trigger*** based on the following criteria: (i) appropriate frequency of CLIP visits by the cHCP (i.e., 4 ± 2 weeks); (ii) N (%) of women who had a CLIP trigger and who were administered treatment as indicated (methyldopa and/or $MgSO_4$); (iii) N (%) of women who had a CLIP trigger and were referred for transport as appropriate, and (iv) N (%) of women who were seen at an CEmOC facility within the appropriate period of time after referral advice given (i.e., 4hr for urgent transport or 24hr for non-urgent transport).

We have assumed a non-compliance rate in appropriate use of the intervention package of 10% in our power calculation. The primary reason for this will be lack of confidence by the cHCP in administering the intervention. We believe this to be a probable overestimate given the existing experience in Sindh where compliance with more technically challenging protocols exceeded 90%.

2.14 LOSS TO FOLLOW-UP

We have assumed a 10% loss to follow-up in our power calculations. The primary reason for this will be women leaving the marital home in one community for their childhood home in another community for labour and delivery.

2.15 TYPES OF ANALYSES/STATISTICAL PLAN

Note that this is an intent-to-treat analysis; all analyses will include all patients randomised.

Trial information and patient disposition

The following information will be presented:

- Number of patients randomised
- Number of patients completing each visit of follow-up

- Median follow-up time
- Frequency table for the reasons for going off-study
- Frequency table for loss to follow-up

Baseline and demographic variables

Summary statistics such as the mean, median, standard deviation, minimum, maximum and inter-quartile range for continuous variables and the number and percentage of patients with various levels of categorical variables will be calculated for ***all subjects randomised within each treatment group for each country*** for variables such as:

- Demographics: maternal age, parity, gestational age at diagnosis, distance from facility
- Socioeconomic status (measured by poverty index), level of education
- Cluster level variables: cHCP density, population density
- Interventions: number of antenatal visits, type of care provider, route of birth, medications given

All summary statistics will be calculated for each treatment group individually as well as for all treatment groups combined.

Primary outcome

The primary outcome is the proportion of pregnancies resulting in at least one of maternal death, stillbirth, neonatal death, or severe morbidity in the mother or child.

Primary analysis

A hierarchical regression model will be used to take into account the clustering of women within the units of randomisation. The model will take into account key determinants of outcome, as defined by the investigators, at both the individual and cluster level.

Sensitivity analyses

We will use multiple imputation to account for those lost to follow-up and examine whether this has any impact on the between-group comparisons.

Secondary outcome

Similar methods will be applied to the individual components of the primary outcome. In addition, the proportion of women achieving birth preparedness and complication readiness will be compared as will be the proportion of women presenting for care at an EmONC facility, and the proportion of facility births.

Other outcomes

Knowledge of pre-eclampsia and eclampsia; total number of seizures or number of pre-admission and post-admission seizures and cost-effectiveness of the intervention.

Safety data

Adverse events will be tracked in intervention and control clusters related to:

- **Methyldopa** administration in the community: relative maternal hypotension on arrival at facility (defined as sBP<110 mmHg)
- **MgSO4** administration in the community: either respiratory depression, coma or death during transport, as diagnosed upon arrival at facility
- **Transport**: Transport-related injury (life or limb) or death during transport

- injection site haematoma or infection (following either community or facility administration of i.m. $MgSO_4$)
- ≥ 20% of women referred to facility being sent back to their communities without follow-up (monitoring community engagement and the CLIP POM).
- The following will be presented by treatment group: number of adverse events (overall and by type), number of women with one or more adverse event(s) (overall and by type).

Additional methodological details

Statistical tests

All statistical tests will be two-sided, with significance levels of 0.05. Comparisons will be by means of risk ratios and mean differences with 95% confidence intervals for each site.

Software to be used

SAS / R / S-plus.

Individual patient data meta-analysis (IPD)

Each country will be analysed separately, with a planned IPD meta-analysis (conducted at UBC) after the completion of all four trials. For the IPD meta-analysis, we will analyse data for women recruited at ≥20 weeks, to standardise data to the latest public declaration of pregnancy (i.e., 20 weeks in Pakistan).

Economic analyses

An economic analysis of the intervention and its impact will be performed from a health systems perspective to guide health services decision-making.

Geo-temporal analyses

We will develop and test a location sensitive maternal health risk index (development funded by CIHR). This index will chart risk for adverse maternal outcomes across multiple communities with different geographic characteristics. We will use Geographical Information Systems to identify 'resilient' communities and individuals and then link environmental correlates related to maternal health with pockets of resilience. Likewise, we will use environmental correlates and outcome data to identify areas and individuals who have poorer than expected outcomes. We will use the data collected in different communities over time to create temporally dynamic map products that chart environmental correlates in relation to shifting maternal morbidity and mortality.

Geospatial analysis will be conducted in each country by an individual from the PRE-EMPT Vancouver team with help from local GIS experts. This analysis will entail using the GPS tagged survey record to chart trends in maternal mortality and morbidity while cross referencing these outcomes to aspects of the built environment like transport and access to health care. Prior to this analysis, all personal identifiers would have been removed from the data. The data will be aggregated to describe frequencies for each of the captured variables at village level. This second set of aggregate data will be then be further analysed at UBC.

2.16 FREQUENCY OF ANALYSES

An interim analysis is planned for each of the four countries once complete data (until 42 days postpartum) has been received for women making up half of the planned sample size for that country.

The stopping rule for both benefit and harm will require an observed difference between groups associated with an alpha <0.001 (power: 80%). Reporting and handling of adverse events will be in accordance with Good Clinical Practice (GCP) guidelines.

A single economic analysis will be performed.

2.17 Subgroup analyses

We will analyse the interventions and outcomes by country and locality (i.e., Ogun State, Maputo and Gaza Provinces, Sindh Province, and Karnataka State).

SECTION 3 TRIAL MANAGEMENT

3.1 PRE-RECRUITMENT PHASE

Currently, we are conducting the CLIP Feasibility Study in the relevant Nigerian, Mozambican, Pakistani, and Indian sites. We will finalize a tailored intervention that will be culturally sensitive, effective at reducing barriers, and increase enthusiasm for the CLIP package amongst decision makers through respectful dialogue with professional associations and government decision makers.

An educational package will be developed, and the cHCPs, midwives and physicians working in the intervention clusters will be trained.

Having spent the first 30 months of the PRE-EMPT grant completing the PIERS modelling, trial design and registration, feasibility and barriers assessment, cHCP, nurse, midwife and physician training, and ethics and contracts processes, we will commence recruiting to the trial after July 2013.

3.2 DATA SAFETY AND MONITORING BOARD

The *DSMB* will be created, including methodological and content expertise and will be comprised mainly of investigators from LMICs.

The role of DSMB will be to deal with any ethical issues that may arise while the trial is in progress and to decide either benefit or harm at the planned interim analyses (½ planned sample size). SAEs will be reported to the DSMB as outlined below. SAEs should be: **(i) serious, (ii) unexpected (in nature, severity, or frequency), and (iii) thought to be related to the study intervention.** This plan reflects the expected nature of the other AE, which are events of note but that do not necessitate stoppage of the trial due to safety concerns prior to the interim analysis.

SAE reporting

When all required information has been gathered in the reporting field site, the field site supervisor will forward 'blocked' copies (without patient, site or allocated intervention group identifiers) of all documentation and an updated 'Serious Adverse Event Form' to the National CLIP Trial Co-ordinating Centre. That centre will assess whether or not: (i) additional information is required, and (ii) the National Principal Investigator and Co-ordinator should be informed immediately, or at the next scheduled meeting. *The* ***documents to be reviewed*** *are: (i) all data forms (including those derived from the POM tool) for each women with a reported primary outcome (for mother or baby), masked to the cluster allocation, and (ii) copies of the relevant facility documents (if applicable) that serve as the 'source documents' and detail the outcome for the woman/baby. If the outcome were reported in the community with no associated facility admission, then the data forms should be reviewed for consistency and to ensure that the woman's story make sense from a clinical perspective.*

After review (and collection of additional information, as applicable) of a reported SAE by the relevant National CLIP PI and National CLIP Trial Co-ordinating Centre, the 'blocked' documentation (including the SAE Form and summary of the case) **should be reviewed by the in-country National Outcomes Adjudication Committee. That Committee will decide whether or not the reported SAE is a true SAE or a pre-specified Trial outcome. Then**, the 'blocked' documentation (including the SAE Form and summary of the case) and the Committee's opinion should be sent to the UBC Trial Co-ordinating Centre (TCC) for review by the UBC Outcomes Adjudication Committee; if UBC agrees that the reported SAE is a true SAE, UBC will send the report to the DSMB for urgent review. However, if UBC agrees that the reported SAE is a pre-specified Trial outcome, then the report will be sent to the DSMB at the time of the next analysis.

The UBC Outcomes Adjudication Committee decision will be reviewed by the CLIP Trial Steering Committee (SC) at the next scheduled quarterly teleconference, or earlier if the UBC Outcomes

Adjudication Committee feels that this is appropriate. Possible reasons for urgent review by the SC are because the reported SAE (i) is not a pre-specified trial outcome (i.e. it is deemed to be a true SAE), or because (ii) is a pre-specified trial outcome but the UBC Outcomes Adjudication Committee has further concerns. After review of the SAE by these groups, the UBC TCC will generate and distribute a summary report of the Serious Unexpected Event(s) to be reported and reviewed by the DSMB at the time of the interim analyses, or earlier if either the UBC Outcomes Adjudication Committee and/or SC have additional concerns.

The summary report should include the 'Serious Unexpected Event Form' received from the site, and a typed summary of all accumulated supporting documentation for each reported event. Ensure all patient, site or allocated intervention group identifiers have been removed. The DSMB can then request further information if they wish to take a closer look at any particular event.

For reported events ruled by the Outcomes Adjudication Committee to be pre-specified trial outcomes (and NOT true SAEs), the UBC TCC will: (i) communicate with the reporting site to let them know about the outcome of the evaluation of their reported Serious Unexpected Event by the UBC Outcomes Adjudication Committee; (ii) inform them that the reported event will also be reviewed by the SC and included in a summary report made to the DSMB at the time of the next scheduled analysis (after DSMB review, a written summary of all reported Serious Unexpected Events, and the DSMB's ultimate assessment of these events, will be distributed to their site (and all sites)); and (iii) invite the site to inform the TCC if they have any concerns.

For reported events ruled by the UBC Outcomes Adjudication Committee and SC to be true SAEs, UBC TCC will: (i) communicate with the reporting site to let them know about the outcome of the evaluation of their reported Serious Unexpected Event by the UBC Outcomes Adjudication Committee and Steering Committee; (ii) inform them that the reported event will also be included in a summary report made to the DSMB (either immediately or at the time of the next scheduled analysis, as decided by the Steering Committee) (after DSMB review, a written summary of all reported Serious Unexpected Events, and the DSMB's ultimate assessment of these events, will be distributed to their site (and all sites)); and (iii) invite the site to inform the UBC TCC if they have any concerns.

For all reported events, following review by the DSMB, the Principal Investigator will prepare a written summary of the reported Serious Unexpected Event(s), and the DSMB's assessment of these event(s). If the event(s) is (are) deemed to be a true SAE, the anonymised SAE Report Form(s) will also be attached. This report will be submitted to: (i) UBC REB. As the Clinical and Data Co-ordinating Centre, UBC will inform the UBC REB of all reported Serious Unexpected Events; (ii) the study sponsor, BMGF, will be informed through the routine monthly/alternate monthly dialogue; and (iii) all Site Investigators, including the reporting Investigator. Each Site Investigator will be responsible for reviewing this Serious Unexpected Event summary report and forwarding it to their local REB. It will be recommended that each Site Investigator check with their local REB for other local reporting requirements.

Membership of the DSMB is:

- **Professor Eileen Hutton (Chair)**

  Associate Professor, Department of Obstetrics and Gynaecology, Assistant Dean, Faculty Health Sciences, and Director, Midwifery Education Program, McMaster University, Hamilton, ON, Canada

  (http://fhs.mcmaster.ca/ceb/faculty_member_hutton.htm).

- **Lehana Thabane**

Professor and Associate Chair, Department of Clinical Epidemiology & Biostatistics, Director, Biostatistics Unit, Centre for Evaluation of Medicine, and Senior Scientist, Population Health Research Institute (PHRI), Hamilton Health Sciences, McMaster University (http://fhs.mcmaster.ca/ceb/faculty_member_thabane.htm).

- **Romano Byaruhanga**

  Consultant Obstetrician, Nsambya, Uganda and President of the Association of Obstetricians and Gynaecologists of Uganda.

  (http://www.sogc.org/aogu/index.aspx?contentID=41).

3.3 Outcome Adjudication committee

Each country will perform a masked review of all occurrences of the primary outcome that is embedded in the data collection process; the national team will review outcomes where there is uncertainty. *The* ***National Outcomes Adjudication Committee*** *will consist of an uneven number of members of the national CLIP Team, including but not limited to one obstetrician, one paediatrician, and one methodologist/trialist.* The UBC management team will provide final arbitration if required.

3.4 Trial registration

The CLIP Trial has been registered with clinicaltrials.gov (ID NCT01911494).

3.5 General assumptions

We assume that the results of the CLIP Feasibility Studies being conducted in Nigeria, Mozambique, Pakistan, and India will inform some of the details of the CLIP trial, including costs. Therefore, each Pilot CLIP Trial will not be started until the relevant CLIP Feasibility Study is completed.

We have assumed that the clinical and data co-ordinating centre will be located at UBC, Vancouver, where the statistical analyses will also be performed. We do not anticipate any delays related to gaining IRB approval for this intervention.

For the CLIP Feasibility Studies, we have received firm stakeholder support and involvement at the ministerial level of health care in the identified countries. We assume that each of the three stages of the relevant CLIP Feasibility Study will be completed according to the specified timelines and budget. In addition, we have assumed the established pattern of community level cRCTs inNigeria, Mozambique, Pakistan, and India will support the CLIP Trial, including cHCP-based BP control.

It is our intention that the results of the household surveys and registries will provide a replete community-level dataset for each participating centre. These datasets will span not only HDP-related deaths and morbidities, but also the other leading causes of maternal mortality (obstetric haemorrhage, obstructed labour, and puerperal sepsis), and will be invaluable resources for local communities, local investigators, demographers, and scientists. Whatever the results of the CLIP Trial, the data will be novel and important and we anticipate the acceptance of resulting paper(s) for publication. We have assumed that no natural or human-caused disaster will occur to curtail our work.

References

(1) Steegers EA, von Dadelszen P, Duvekot JJ, Pijnenborg R. Pre-eclampsia. *Lancet* 2010.

(2) Hutcheon JA, Lisonkova S, Magee LA, von Dadelszen P, Woo HL, Liu S et al. Optimal timing of delivery in pregnancies with pre-existing hypertension. *BJOG* 2011; 118(1):49-54.

(3) Duley L, Gulmezoglu AM, Henderson-Smart DJ, Chou D. Magnesium sulphate and other anticonvulsants for women with pre-eclampsia. *Cochrane Database Syst Rev* 2010;(11):CD000025.

(4) Duley L, Henderson-Smart DJ, Walker GJ, Chou D. Magnesium sulphate versus diazepam for eclampsia. *Cochrane Database Syst Rev* 2010;(12):CD000127.

(5) Duley L, Henderson-Smart DJ, Chou D. Magnesium sulphate versus phenytoin for eclampsia. *Cochrane Database Syst Rev* 2010;(10):CD000128.

(6) Duley L, Gulmezoglu AM, Chou D. Magnesium sulphate versus lytic cocktail for eclampsia. *Cochrane Database Syst Rev* 2010;(9):CD002960.

(7) Magee LA, Abalos E, von Dadelszen P, Sibai B, Easterling T, Walkinshaw S. How to manage hypertension in pregnancy effectively. *Br J Clin Pharmacol* 2011; 72(3):394-401.

(8) Bhutta ZA, Soofi S, Memon Z. Training for Lady Health Workers clarified. *Bull World Health Organ* 2008; 86(12):B.

(9) Bhutta ZA, Memon ZA, Soofi S, Salat MS, Cousens S, Martines J. Implementing community-based perinatal care: results from a pilot study in rural Pakistan. *Bull World Health Organ* 2008; 86(6):452-459.

(10) Bhutta ZA, Rizvi A, Raza F, Hotwani S, Zaidi S, Moazzam HS et al. A comparative evaluation of multiple micronutrient and iron-folic acid supplementation during pregnancy in Pakistan: impact on pregnancy outcomes. *Food Nutr Bull* 2009; 30(4 Suppl):S496-S505.

(11) Bhutta ZA, Lassi ZS. Empowering communities for maternal and newborn health. *Lancet* 2010; 375(9721):1142-1144.

(12) Bhutta ZA, Soofi S, Cousens S, Mohammad S, Memon ZA, Ali I et al. Improvement of perinatal and newborn care in rural Pakistan through community-based strategies: a cluster-randomised effectiveness trial. *Lancet* 2011; 377(9763):403-412.

(13) Elwyn G, Legare F, van der Weijden T, Edwards A, May C. Arduous implementation: does the Normalisation Process Model explain why it's so difficult to embed decision support technologies for patients in routine clinical practice. *Implement Sci* 2008; 3:57.

(14) May C. A rational model for assessing and evaluating complex interventions in health care. *BMC Health Serv Res* 2006; 6:86.

(15) May C, Finch T, Mair F, Ballini L, Dowrick C, Eccles M et al. Understanding the implementation of complex interventions in health care: the normalization process model. *BMC Health Serv Res* 2007; 7:148.

(16) Michie S, Johnston M, Abraham C, Lawton R, Parker D, Walker A. Making psychological theory useful for implementing evidence based practice: a consensus approach. *Qual Saf Health Care* 2005; 14(1):26-33.

(17) Thaddeus S, Maine D. Too far to walk: maternal mortality in context. *Soc Sci Med* 1994; 38(8):1091-1110.

(18) Payne B, Hutcheon JA, Qu Z, Haniff F, Bhutta Z, Biryabarema C et al. miniPIERS (Pre-eclampsia Integrated Estimate of RiSk): development of a clinical prediction model for use in low and middle income countries (LMICs) [abstract]. *Pregn Hypertens* 2012; 2(3):195-196.

(19) von Dadelszen P, Payne B, Li J, Ansermino JM, Broughton PF, Cote AM et al. Prediction of adverse maternal outcomes in pre-eclampsia: development and validation of the fullPIERS model. *Lancet* 2011; 377(9761):219-227.

(20) Cote AM, Firoz T, Mattman A, Lam EM, von Dadelszen P, Magee LA. The 24-hour urine collection: gold standard or historical practice? *Am J Obstet Gynecol* 2008; 199(6):625-626.

(21) Firoz T, Magee LA, Payne BA, Menzies JM, von Dadelszen P. The PIERS experience: research or quality improvement? *J Obstet Gynaecol Can* 2012; 34(4):379-381.

(22) Kozic JR, Benton SJ, Hutcheon JA, Payne BA, Magee LA, von Dadelszen P. Abnormal liver function tests as predictors of adverse maternal outcomes in women with preeclampsia. *J Obstet Gynaecol Can* 2011; 33(10):995-1004.

(23) Laskin S, Payne B, Hutcheon JA, Qu Z, Douglas MJ, Ford J et al. The role of platelet counts in the assessment of inpatient women with preeclampsia. *J Obstet Gynaecol Can* 2011; 33(9):900-908.

(24) Millman AL, Payne B, Qu Z, Douglas MJ, Hutcheon JA, Lee T et al. Oxygen saturation as a predictor of adverse maternal outcomes in women with preeclampsia. *J Obstet Gynaecol Can* 2011; 33(7):705-714.

(25) Payne B, Magee LA, Cote AM, Hutcheon JA, Li J, Kyle PM et al. PIERS proteinuria: relationship with adverse maternal and perinatal outcome. *J Obstet Gynaecol Can* 2011; 33(6):588-597.

(26) Payne B, Hodgson S, Hutcheon J, Joseph K, Li J, Lee T et al. Performance of the fullPIERS model in predicting adverse maternal outcomes in pre-eclampsia using patient data from the PIERS (Pre-eclampsia Integrated Estimate of RiSk) cohort, collected on admission. *BJOG* 2012.

(27) Payne BA, Kyle PM, Lisonkova S, Magee LA, Pullar B, Qu Z et al. An assessment of predictive value of the biophysical profile in women with preeclampsia using data from the fullPIERS database. *Pregnancy Hypertens* 2013; [in press].

(28) Yen TW, Payne B, Qu Z, Hutcheon JA, Lee T, Magee LA et al. Using clinical symptoms to predict adverse maternal and perinatal outcomes in women with preeclampsia: data from the PIERS (Pre-eclampsia Integrated Estimate of RiSk) study. *J Obstet Gynaecol Can* 2011; 33(8):803-809.

(29) Magee LA, von Dadelszen P. The management of severe hypertension. *Semin Perinatol* 2009; 33(3):138-142.

(30) Tuffnell DJ, Jankowicz D, Lindow SW, Lyons G, Mason GC, Russell IF et al. Outcomes of severe pre-eclampsia/eclampsia in Yorkshire 1999/2003. *BJOG* 2005; 112(7):875-880.

(31) National Collaborating Centre for Women's and Children's Health. Hypertension in pregnancy: the management of hypertensive disorders during pregnancy. CG107. 2010. London, National Institute for Health and Clinical Excellence.
Ref Type: Report

(32) Magee LA, Miremadi S, Li J, Cheng C, Ensom MH, Carleton B et al. Therapy with both magnesium sulfate and nifedipine does not increase the risk of serious magnesium-related maternal side effects in women with preeclampsia. *Am J Obstet Gynecol* 2005; 193(1):153-163.

(33) Chesley LC, TEPPER I. Plasma levels of magnesium attained in magnesium sulfate therapy for preeclampsia and eclampsia. *Surg Clin North Am* 1957; 37(2):353-367.

(34) World Health Organization. World Health Organization recommendations for preventation and treatment of pre-eclampsia and eclampsia. 2011. Geneva, World Health Organization.
Ref Type: Report

(35) Chiwuzie J, Okojie O, Okolocha C, Omorogbe S, Oronsaye A, Akpala W et al. Emergency loan funds to improve access to obstetric care in Ekpoma, Nigeria. The Benin PMM Team. *Int J Gynaecol Obstet* 1997; 59 Suppl 2:S231-S236.

(36) Essien E, Ifenne D, Sabitu K, Musa A, Alti-Mu'azu M, Adidu V et al. Community loan funds and transport services for obstetric emergencies in northern Nigeria. *Int J Gynaecol Obstet* 1997; 59 Suppl 2:S237-S244.

(37) Fofana P, Samai O, Kebbie A, Sengeh P. Promoting the use of obstetric services through community loan funds, Bo, Sierra Leone. The Bo PMM Team. *Int J Gynaecol Obstet* 1997; 59 Suppl 2:S225-S230.

(38) Shehu D, Ikeh AT, Kuna MJ. Mobilizing transport for obstetric emergencies in northwestern Nigeria. The Sokoto PMM Team. *Int J Gynaecol Obstet* 1997; 59 Suppl 2:S173-S180.

(39) Sadovsky E, Ohel G, Havazeleth H, Steinwell A, Penchas S. The definition and the significance of decreased fetal movements. *Acta Obstet Gynecol Scand* 1983; 62(5):409-413.

(40) Duley L, Henderson-Smart DJ, Walker GJ, Chou D. Magnesium sulphate versus diazepam for eclampsia. *Cochrane Database Syst Rev* 2010;(12):CD000127.

(41) Duley L, Henderson-Smart DJ, Chou D. Magnesium sulphate versus phenytoin for eclampsia. *Cochrane Database Syst Rev* 2010;(10):CD000128.

(42) Duley L, Gulmezoglu AM, Chou D. Magnesium sulphate versus lytic cocktail for eclampsia. *Cochrane Database Syst Rev* 2010;(9):CD002960.

(43) Duley L, Matar HE, Almerie MQ, Hall DR. Alternative magnesium sulphate regimens for women with pre-eclampsia and eclampsia. *Cochrane Database Syst Rev* 2010;(8):CD007388.

(44) Department of Making Pregnancy Safer. Pregnancy, childbirth, postpartum and newborn care: a guide for essential practice. B13. 2006. Geneva, World Health Organization. Integrated management of pregnancy and childbirth.
Ref Type: Serial (Book,Monograph)

(45) Begum MR, Begum A, Quadir E. Loading dose versus standard regime of magnesium sulfate in the management of eclampsia: a randomized trial. *J Obstet Gynaecol Res* 2002; 28(3):154-159.

(46) Begum R, Begum A, Johanson R, Ali MN, Akhter S. A low dose ("Dhaka") magnesium sulphate regime for eclampsia. *Acta Obstet Gynecol Scand* 2001; 80(11):998-1002.

(47) Bhattacharjee N, Saha SP, Ganguly RP, Patra KK, Dhali B, Das N et al. A randomised comparative study between low-dose intravenous magnesium sulphate and standard intramuscular regimen for treatment of eclampsia. *J Obstet Gynaecol* 2011; 31(4):298-303.

(48) Darngawn L, Jose R, Regi A, Bansal R, Jeyaseelan L. A shortened postpartum magnesium sulfate prophylaxis regime in pre-eclamptic women at low risk of eclampsia. *Int J Gynaecol Obstet* 2012; 116(3):237-239.

(49) Duley L, Matar HE, Almerie MQ, Hall DR. Alternative magnesium sulphate regimens for women with pre-eclampsia and eclampsia. *Cochrane Database Syst Rev* 2010;(8):CD007388.

(50) Malapaka SV, Ballal PK. Low-dose magnesium sulfate versus Pritchard regimen for the treatment of eclampsia imminent eclampsia. *Int J Gynaecol Obstet* 2011; 115(1):70-72.

(51) Regmi MC, Aggrawal A, Pradhan T, Rijal P, Subedi A, Uprety D. Loading dose versus standard regimen of magnesium sulphate in eclampsia--a randomized trial. *Nepal Med Coll J* 2010; 12(4):244-247.

(52) Al Haque N, Chowdhury ME, Huq NL, Ahmed A, DasGupta SK, Quaiyum MA. Baseline findings: Shahjadpur integrated maternal and neonatal health project. 2010. Dhaka, ICDDR,B.
Ref Type: Report

(53) Huq NL, Moran AC, Quaiyum MA, Chowdhury ME, Ahmed A, Al Haque N. Formative research findings: Shahjadpur integrated maternal and neonatal health project. 2010. Dhaka, ICCDR,B.
Ref Type: Report

(54) Lu JF, Nightingale CH. Magnesium sulfate in eclampsia and pre-eclampsia: pharmacokinetic principles. *Clin Pharmacokinet* 2000; 38(4):305-314.

(55) Koopmans CM, Bijlenga D, Groen H, Vijgen SM, Aarnoudse JG, Bekedam DJ et al. Induction of labour versus expectant monitoring for gestational hypertension or mild pre-eclampsia after 36 weeks' gestation (HYPITAT): a multicentre, open-label randomised controlled trial. *Lancet* 2009; 374(9694):979-988.

(56) Mozambique. *Popul Policy Compend* 1982;1-7.

(57) Chapman RR. Endangering safe motherhood in Mozambique: prenatal care as pregnancy risk. *Soc Sci Med* 2003; 57(2):355-374.

(58) Sevene E, Lewin S, Mariano A, Woelk G, Oxman AD, Matinhure S et al. System and market failures: the unavailability of magnesium sulphate for the treatment of eclampsia and pre-eclampsia in Mozambique and Zimbabwe. *BMJ* 2005; 331(7519):765-769.

(59) Bhutta ZA, Ali N, Hyder AA, Wajid A. Perinatal and newborn care in Pakistan: seeing the unseen. In: Bhutta ZA, editor. Maternal and child health in Pakistan: challenges and opportunities. Karachi: Oxford University Press; 2004.

(60) Duley L, Henderson-Smart DJ, Meher S. Drugs for treatment of very high blood pressure during pregnancy. *Cochrane Database Syst Rev* 2006; 3:CD001449.

(61) Magee LA, Cham C, Waterman EJ, Ohlsson A, von Dadelszen P. Hydralazine for treatment of severe hypertension in pregnancy: meta-analysis. *BMJ* 2003; 327(7421):955-960.

(62) Souza LM, Riera R, Saconato H, Demathe A, Atallah AN. Oral drugs for hypertensive urgencies: systematic review and meta-analysis. *Sao Paulo Med J* 2009; 127(6):366-372.

(63) Brown MA, Buddle ML, Farrell T, Davis GK. Efficacy and safety of nifedipine tablets for the acute treatment of severe hypertension in pregnancy. *Am J Obstet Gynecol* 2002; 187(4):1046-1050.

(64) Altman D, Carroli G, Duley L, Farrell B, Moodley J, Neilson J et al. Do women with pre-eclampsia, and their babies, benefit from magnesium sulphate? The Magpie Trial: a randomised placebo-controlled trial. *Lancet* 2002; 359(9321):1877-1890.

Table 4 CLIP interventions

Level of care/foci	**Care component**
**cHCPs**	
**Triage**	◊ Community education: symptoms, signs, & consequences of pre-eclampsia<br>◊ Community engagement & mobilization: transport funds; prior permissions from proxy decision-makers; health system managers & funders<br>◊ Menstrual calendars to be held by married women of reproductive age (South Asia) or all women of reproductive age (Africa)<br>◊ Urine dipstick at first antenatal encounter<br>◊ Symphysis-fundal height at first antenatal encounter $\geq 24^{+0}$ weeks to assess GA if unknown<br>◊ Ongoing antenatal surveillance (BP [& urine dipsticks, if indicated] as described in the protocolprotocolprotocol)<br>◊ Opportunistic screening & triage of symptomatic women<br>◊ Diagnostic & triage tool (miniPIERS)<br>◊ PIERS on the Move decision aid Android application
**Treatment**	◊ 750mg α-methyldopa p.o. for severe hypertension (sBP ≥160mmHg); single dose only<br>◊ 10g $MgSO_4$ IM loading dose (single dose only) for women with stroke, eclampsia (single or recurrent seizures), vaginal bleeding (presumed severe pre-eclampsia), severe hypertension (presumed severe pre-eclampsia), or a miniPIERS probability ≥25%
**Transport**	◊ Arrangements made for patient transfer to nearest referral centre<br>o High risk: within 4h<br>o Low risk: within 24h<br>◊ Discussion with referral centre<br>◊ Patient transferred
**Primary health centre**	
**Triage**	◊ Menstrual calendars to be held by married women of reproductive age (South Asia) or all women of reproductive age (Africa)<br>◊ Symphysis-fundal height at first antenatal encounter $\geq 24^{+0}$ weeks to assess GA<br>◊ Ongoing antenatal surveillance (BP and urine dipsticks every 4 weeks)<br>◊ Opportunistic screening & triage of symptomatic women<br>◊ Diagnostic & triage tool (miniPIERS)<br>◊ PIERS on the Move decision aid Android application
**Treatment**	◊ 750mg α-methyldopa p.o. for severe hypertension (sBP ≥160mmHg); single dose only (not repeated if already administered by cHCP in community)<br>◊ 10g $MgSO_4$ IM loading dose (single dose ONLY if not already administered by cHCP; no repeat dosing) for women with stroke, eclampsia (single or recurrent seizures), vaginal bleeding (presumed severe pre-eclampsia), severe hypertension (presumed severe pre-eclampsia), or a miniPIERS probability ≥25%
**Transport**	◊ Arrangements made for patient transfer to nearest EmOC facility<br>◊ Patient transferred
**Hospital providing CEmOC– unfunded by CLIP but expected standard of care (supported by planned facility enhancement)**	
**Triage**	◊ Triage tool (miniPIERS and/or fullPIERS)
**Treatment**	◊ 200mg labetalol p.o. or 10mg nifedipine p.o. or hydralazine 5mg IV for severe hypertension (sBP ≥160mmHg); maintenance therapy titrated against response; parenteral antihypertensives as required<br>◊ $MgSO_4$ loading dose (IV/IM if not already administered in the community or 10g IM MgSO4 was administered more than 4 hr prior to facility admission) for women with severe hypertension or vaginal bleeding (as indicators of severe pre-eclampsia) or eclampsia (single); MgSO4 dose of 2g IV for women who had a seizure after the 10g IM MgSO4 was administered in the community. Then, maintenance therapy (usually 1g/h) until 24 hours postpartum.<br>◊ Arrangements made for definitive therapy<br>◊ If >local threshold for viability (e.g., 28 weeks' by best clinical estimate), <34+0 weeks', and PIERS probability <25%: administer 12mg dexamethasone i.m. x 2 12h apart, wait 48h and deliver (transfer to regional centre, if relevant)<br>◊ If <viability, ≥34+0 weeks', or PIERS probability ≥25%: deliver
**Transport (as required)**	◊ Arrangements made for patient transfer to regional (e.g., Hyderabad DHQ) or supraregional (e.g., AKU Medical Centre/JPMC) referral centres, if indicated<br>◊ Patient transferred

AKU Aga Khan University; **BP** blood pressure; **CPD** continuing professional development; **DHQ** district headquarter hospital; **CEmOC** emergency obstetric care; **GA** gestational age;; **i.m.** intramuscularly; **JPMC** Jinnah Postgraduate Medical College; **p.o.** orally ; **sBP** systolic blood pressure

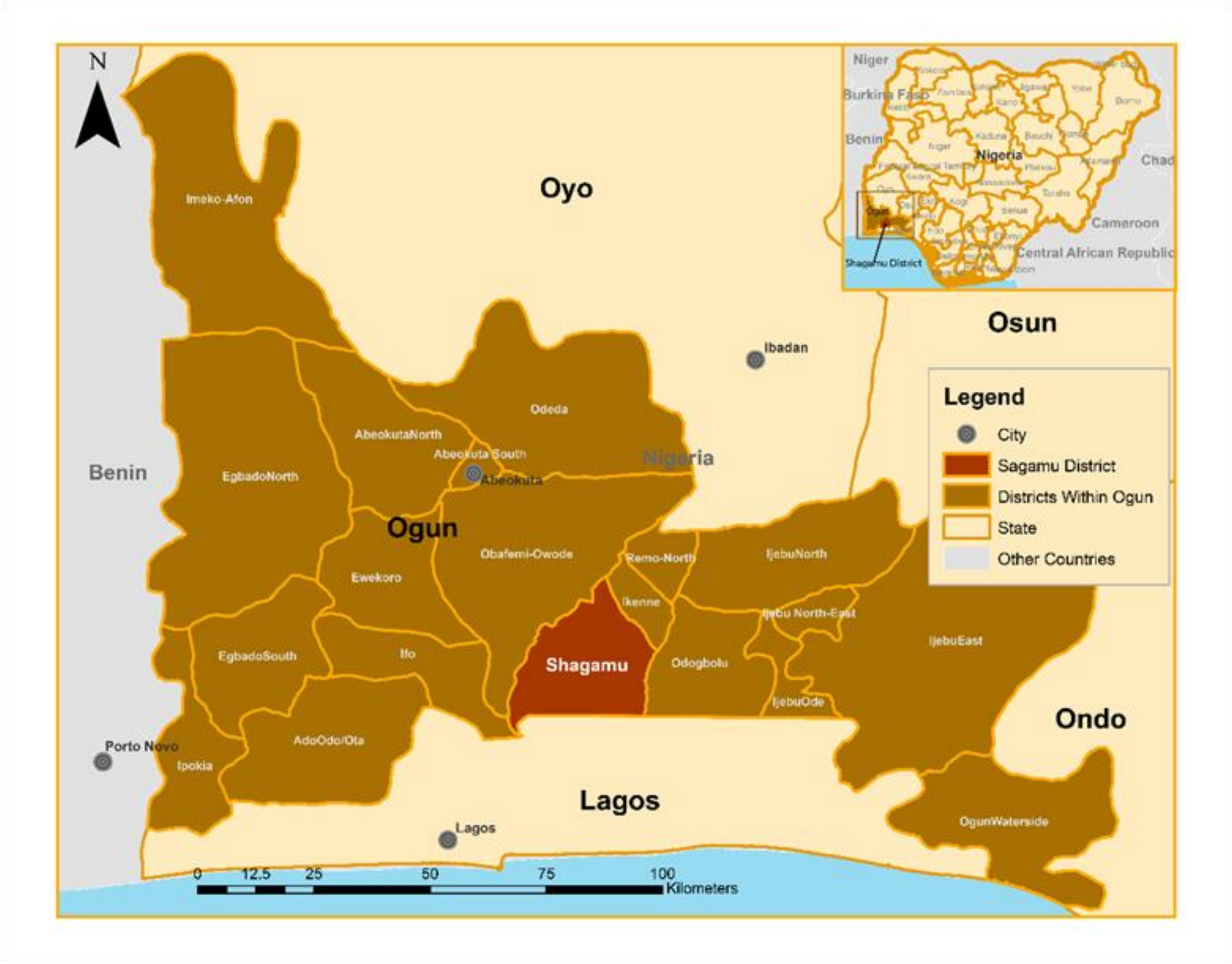

FIGURE 11. Ogun State, Nigeria

Putting the physical location of the CLIP Trial into context

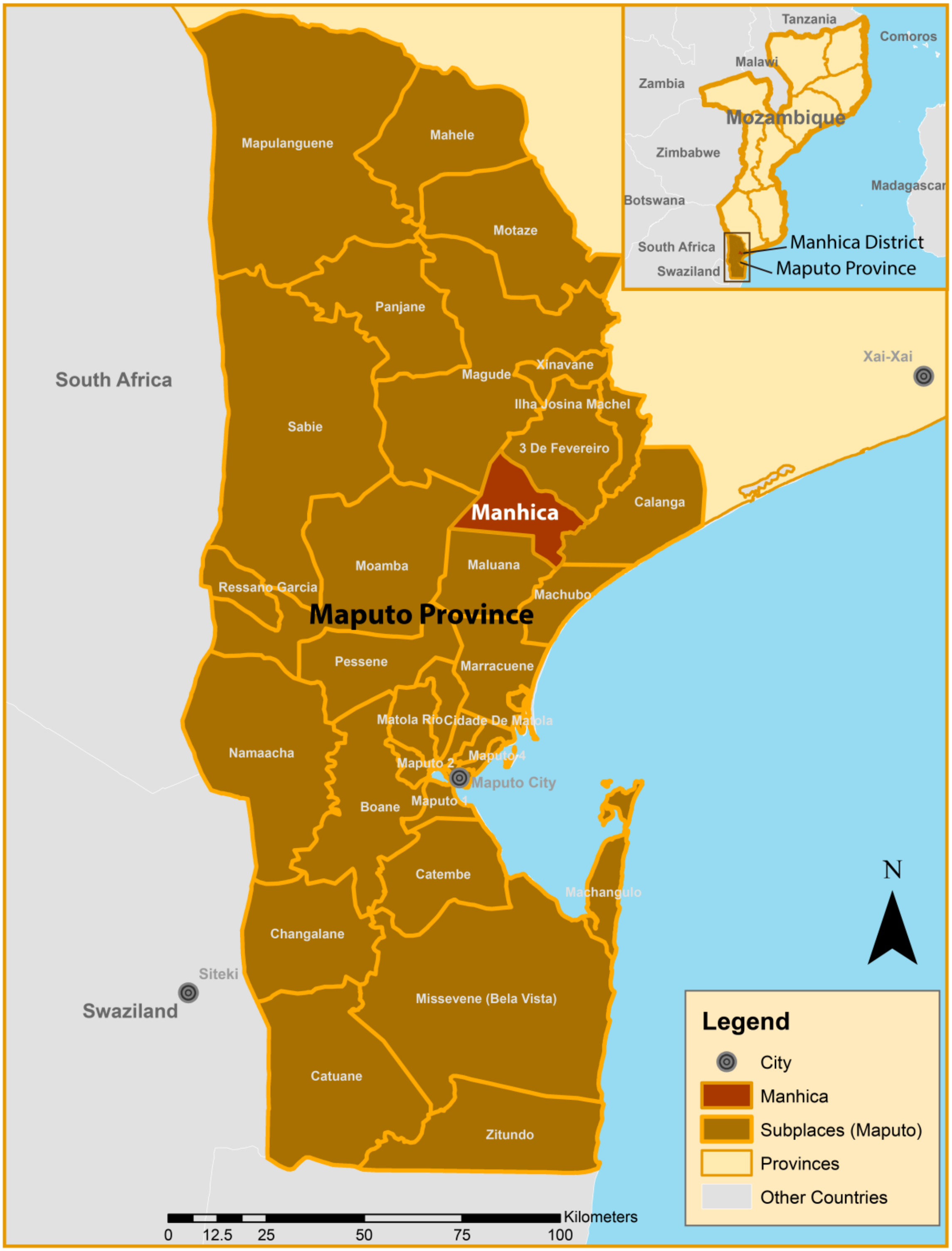

Figure 12 Maputo Province, Mozambique

Putting the physical location of the CLIP Trial into context

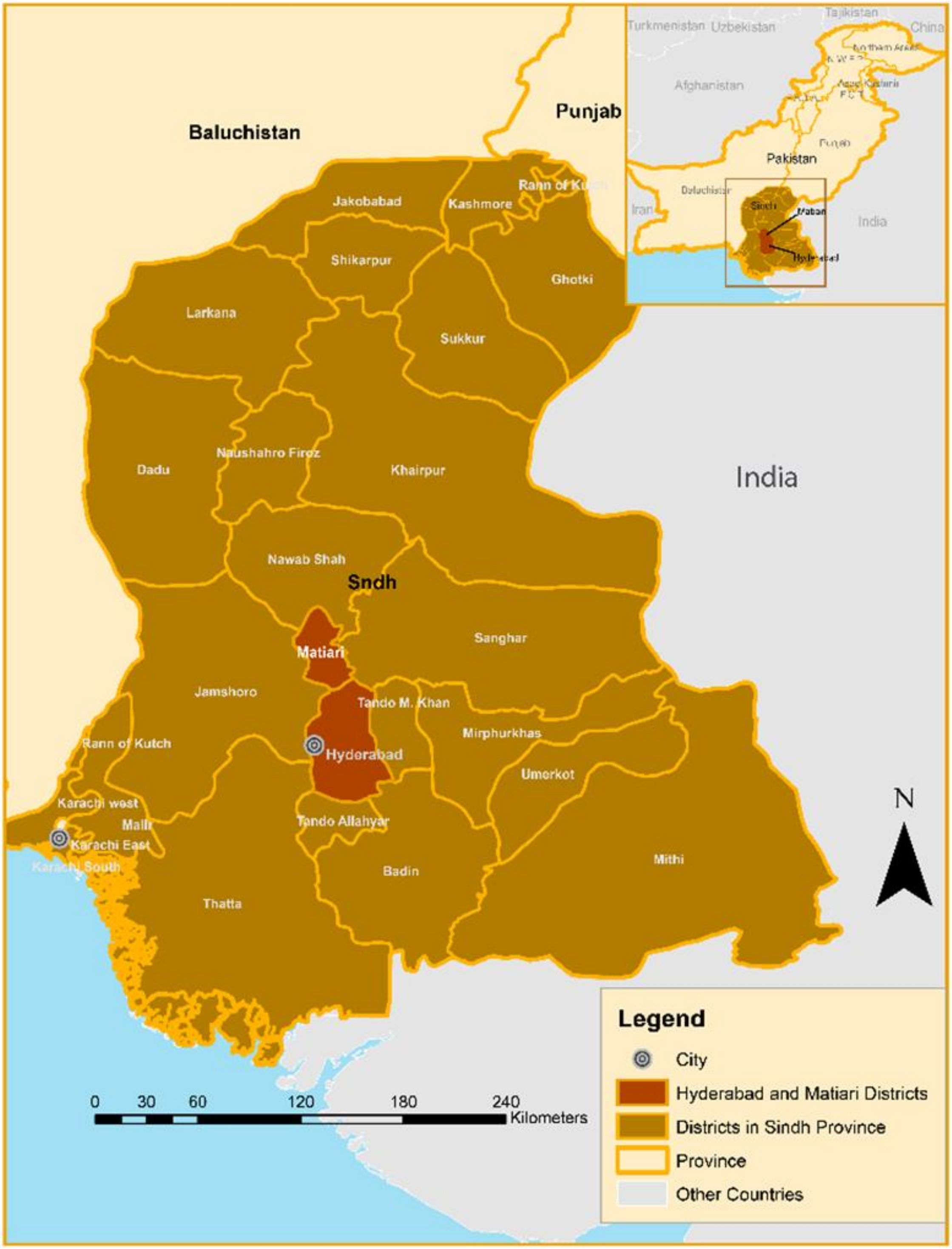

Figure 13. Hyderabad and Matiari Districts, Sindh Province, Pakistan

Putting the physical location of the CLIP Trial into context

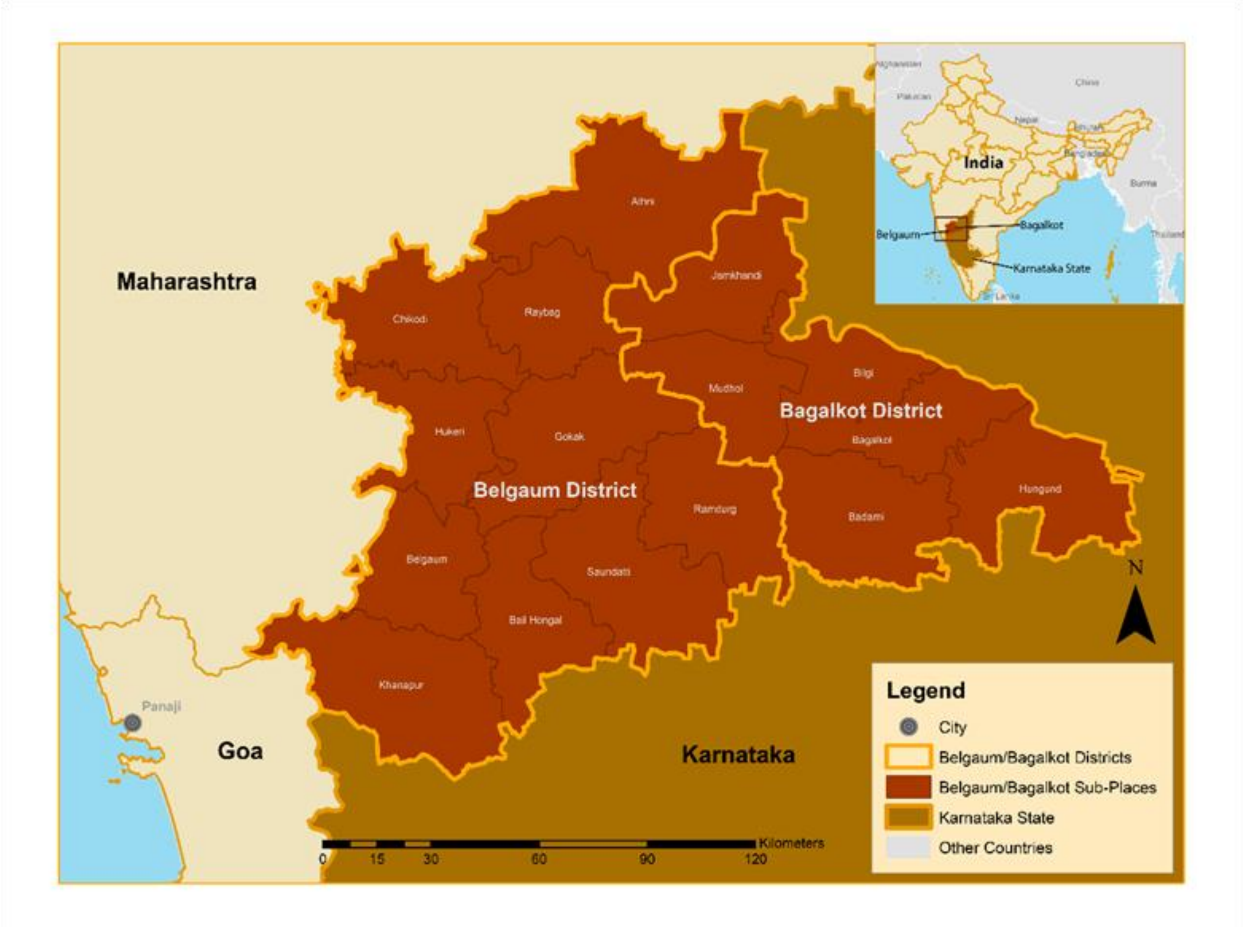

Figure 14. Belgaum and Bagalkot Districts, Karnataka State, India

Putting the physical location of the CLIP Trial into context

Appendix A – Summary of site visit and initial Feasibility Study findings

Partnerships

The following stakeholders have been engaged and have provided support for the trial:

Nigeria – Academic

Centre for Research in Reproductive Health, Sagamu, Ogun State, Nigeria.

Nigeria - Regional & national

Commissioner of Health, Ogun State; Ogun State Ministry of Health; Olabisi Onabanjo University Teaching Hospital; Medical Officers of Health, Ogun State LGAs; Society of Gynaecology and Obstetrics of Nigeria (SOGON); Association of Maternal and Fetal Medicine Specialists of Nigeria; Yewa South Local Government (Ilaro State Hospital, Leysley PHC, Igbo-gidi PHC); Remo North Local Government (General Hospital, Isara, Ode-Remo PHC, Ipara PHC, Idi-Aba Health Post; Sagamu Local Government (Ogijo PHC, Sagamu LGA) and Imeko Local Government (General Hospital, Imeko, Imeko PHC).

Mozambique - Academic

Eduardo Mondlane University, Maputo, Mozambique

Mozambique - Regional & National

Mozambique Ministry of Health; Maputo Municipal Council (responsible for health); Mozambique Association of Obstetricians and Gynecologists (AMOG); Eduardo Mondlane University, Maputo; Maputo Central Hospital; Clinton Foundation.

Pakistan - Academic

Aga Khan University and Jinnah Postgraduate Medical College, Karachi, Pakistan

Pakistan - Regional & national

Sindh Ministry of Health; National Lady Health Worker Programme; Society of Obstetricians and Gynaecologists of Pakistan; Isra University Hospital; Liaquat University Medical Health Sciences Hospital; Aga Khan Maternal & Child Care Centre, Hyderabad; Taluka Headquarter Hospital; Aga Khan University Hospital, Karachi; Jinnah Postgraduate Medical College Hospital, Karachi.

India - Academic

Jawaharlal Nehru Medical College, Karnataka Lingayat Education University, Belgaum, India.

India - Regional & national

District Health Officers, Belgaum and Bagalkot; Chief Executive Officer, Zilla Panchayat (local self government), Belgaum and Bagalkot; Programme Director, RCH, Ministry of Health and Family Welfare, Government of Karnataka, Bangalore; Reproductive Health and Nutrition Division, Indian Council of Medical Research, New Delhi; Representatives of Federation of Obstetric and Gynaecological Societies of India (FOGSI) and Indian Academy of Paediatrics (IAP).

World Health Organization Department of Maternal, Newborn, Child and Adolescent Health (MNCAH)

The chair of the PRE-EMPT objective 5 (knowledge translation) group is Dr Matthews Mathai, from this WHO department. The MNCAH group was instrumental in co-ordinating the development of the 2011 WHO pre-eclampsia guidelines, and will be instrumental in updating the guideline to reflect the findings of

the CLIP trial, and in promulgating the results of the CLIP trial to key decision makers in LMICs. Dr Lale Say is a member of the advisory group advising on maternal mortality and morbidity methods for CLIP.

UNDP/UNFPA/WHO/World Bank Special Programme of Research, Development and Research Training in Human Reproduction (RHR)

The WHO RHR group have been important partners in the PIERS research agenda (Mario Merialdi and Mariana Widmer), as well as for objectives 1 (Ana Pilar Bertràn) and 5 (Metin Gülmezoglu) of the PRE-EMPT project.

Royal College of Obstetricians and Gynaecologists (RCOG)

We have developed a partnership with the RCOG (London) International Office (Senior Vice-President Professor James Walker). An element of the RCOG's international mandate is to strengthen the capacity of inpatient facilities in LMICs in terms of the use of evidence-based care. We will co-ordinate our activities in Nigeria, Mozambique, Pakistan, and India so that the RCOG will support public and private facility capacity building in the referral centres in Nigeria (Sagamu), Mozambique (Manihça and Maputo), Pakistan (Matiari, Hyderabad and Karachi), and India (Belgaum and Bagalkot). This commitment of time, energy and expertise will ensure that women in the study clusters will receive timely and effective care in the centres to which they are referred.

Society of Obstetricians and Gynaecologists of Canada (SOGC)

We have developed a partnership with the SOGC International ALARM Course team. An element of the SOGC's international mandate is to strengthen the capacity of inpatient facilities in LMICs in terms of the use of evidence-based care. We will co-ordinate our activities in Nigeria, Mozambique, Pakistan, and India so that the SOGC will support public and private facility capacity building in the referral centres in Nigeria (Sagamu), Mozambique (Manihça and Maputo), Pakistan (Matiari, Hyderabad and Karachi), and India (Belgaum and Bagalkot). This commitment of time, energy and expertise will ensure that women in the study clusters will receive timely and effective care in the centres to which they are referred.

USAID/MCHIP (Maternal & Child Health Integrated Program)

We have developed a partnership with MCHIP, particulary through Jeffrey Smith (PRE-EMPT TAG member). MCHIP is the USAID Bureau for Global Health's flagship programme focussed on maternal, neonatal and child mortality reduction and acceleration of progress towards MDGs 4 and 5. An element of MCHIP's mandate is to strengthen the capacity of inpatient facilities in LMICs in terms of the use of evidence-based care. We will co-ordinate our activities in Nigeria, Mozambique, Pakistan, and India so that the MCHIP will support public and private facility capacity building in the referral centres in Nigeria (Sagamu), Mozambique (Manihça and Maputo), Pakistan (Matiari, Hyderabad and Karachi), and India (Belgaum and Bagalkot). This commitment of time, energy and expertise will ensure that women in the study clusters will receive timely and effective care in the centres to which they are referred.

Current practice - the provision of maternity care and pre-eclampsia interventions

Nigeria

Antenatal care is delivered at the primary health centre level. Most women attend antenatal care every 3-4 weeks up to 28 weeks gestation, every 2 weeks till 36weeks gestation and weekly thereafter. A typical antenatal visit does include blood pressure measurement and proteinuria testing by indication. Few clinics have a laboratory or ultrasound capacity. Much of obstetric services are paid out of pocket. Few PHCs have ambulance services for referral of complex cases.

Community Health Extension Worker (CHEW) training & current scope of practice

Community Health Extension Workers (CHEW) are responsible for delivery of primary health care

services in Nigeria. They work both in the community and clinic settings; however due to workload restraints CHEWs often remain stationed predominantly at the PHC. The training and regulation of practice is under the jurisdiction of Community Health Practitioners' Registration Board of Nigeria. The CHEW program includes certification in community health, practical exercises and examinations, supervised clinical experience and supervised community experience. The areas of focus for CHEW training relevant to CLIP are: English training, computer education, medical sociology, reproductive health, clinical skills, and referral system and outreach services. The current CHEW curriculum does not include specific training for management or diagnosis of pre-elcampsia/eclampsia or any other pregnancy complication.

Mozambique

The Mozambique health system is largely supported by foreign aid [56]. There are significant human resource shortages which require task shifting for obstetric care services. Countrywide, there is a low number of a available skilled birth attendants. Access to primary health care facilities, where antenatal care is delivered, is also low [56]. The first antenatal visit is typically delayed until the 2nd or 3rd trimester, with 84% coverage for at least one antenatal visit during pregnancy [57]. As a result of these constraints the majority of births are rural many without skilled attendants.

Magnesium sulphate has long been used in Mozambique for the management of pre-eclampsia and eclampsia, however, its use has been primarily at central hospitals and alternative anti-seizure medications have been used in the periphery [58].

Agente Communitarios de Saude (ACS) training & current scope of practice

Agente Communitarios de Saude (or basic multi-task health care helpers) help to promote health in their communities; constituting the link between them and the national health system. ACSs are recruited from within the community and receive six months' training to provide basic preventive (community health education) and curative community based-care for diarrhoea, pneumonia and malaria, focussing on the widespread use of diagnostics and appropriate treatment. The ACSs allow isolated populations to have access to basic health care and essential drugs for high burden diseases like malaria, and act as an entry point for referral to higher level health services, providing basic first aid before transfer where needed.

India

The Government of India has recently committed to the National Rural Health Mission. This initiative aims to obtain universal skilled birth attendance across the country. The National Rural Health Mission highlights the role of ANMs in providing antenatal care, timely referrals, and provision of treatment in emergency obstetric cases. Health care workers providing obstetric care at the community level are ANMs, ASHAs and Anganwadi Workers. Each antenatal visit should include: a medical history, physical exam, abdominal exam, laboratory investigations, clinical interventions (IFA supplement, TT injection, malaria), and health promotion counselling. A minimum of four antenatal visits for all pregnancies is encouraged: before 12 weeks, 12-26 weeks, 28-34 weeks and 36 weeks-term.

Facilities are generally equipped to manage cases of pre-eclampsia and eclampsia. Due to concerns regarding safety and availability diazepam is frequently used in place of $MgSO_4$.

Auxiliary Nurse Midwife (ANM) training & current scope of practice

Training for Assistant Nurse Midwives includes a wide range of health competencies: understanding of holistic health/social determinants of health, ability to mobilize communities, provide emergency care, treatment of minor ailments, midwifery services and referral, basic neonatal care and referral, child services, guide and train TBAs and AWWs, health counselling, participate in National programmes, collaborate with community organizations, manage health care settings. Their midwifery services include

care during pregnancy, intrapartum and postpartum.

They are expected to measure blood pressure at every visit and test for albumin in the cases of high blood pressure. If a woman is identified with high blood pressure the ANM is responsible to referral to a 24 hour PHC to initiate antihypertensive therapy. ANMs also follow-up and advise of the warning symptoms. In cases of eclampsia the ANM should ensure safety, give $MgSO_4$ (10g i.m.) and refer to a facility within two hours.

Accredited Social Health Activisit (ASHA) training & current scope of practice

ASHAs are local women trained to act as health educators and promoters in their communities. They receive a total period of 28 days' training in five episodes. However, this core training is supplemented by ongoing CPD activity in parallel with the development of necessary skills and expertise through on the job training. After a period of 6 months of functioning in her village, she is then sensitised to issues related to HIV, AIDS, and STIs (prevention and referral), as well as trained on newborn care.

Their tasks include motivating women to have a facility birth, bringing children to immunisation clinics, encouraging family planning, treating basic illness and injury with first aid, keeping demographic records, and improving village sanitation. In addition, ASHAs are central to communication between the health care system and rural populations.

Pakistan

Pakistan's public health system is centralized under the Federal Government and provincial Health Ministries. The public health sector employs LHWs and Lady Health Visitors (LHV) in rural sectors and nurses and doctors in health facilities and hospitals.[1] According to the 2008 Demographic and Health Survey: Pakistan, less than 30% of women receive four antenatal visits during pregnancy. In many rural settings, staffing levels are inadequate and referral systems function poorly. Almost 70% of births take place at home, usually attended by a Dai (traditional birth attendant [TBA]) [59]. Rural and urban public facilities provide free consultation for obstetric care, whereas consultation charges in private facilities range from $o.5-3USD in rural areas and $4-16USD in urban areas. Some public and private facilities provide ambulance services, additional ambulance services are provided by non-profit organizations in some regions. The majority of health facilities in Hydrabad and Matiari do not have protocols/guidelines for managing pre-eclampsia and eclampsia. There is awareness of $MgSO_4$ for the management of pre-eclampsia and eclampsia however, many continue to use diazepam.

Lady Health Worker (LHW) training & current scope of practice

The Lady Health Worker Programme commenced in 1994 and currently employs 96000 LHWs as part of the federal healthcare system. Each LHW has to cover a population of around 1000-1500 individuals. This existing number of LHWs is insufficient as WHO suggests the requirement of 150,000 LHW's to cater to the country's healthcare needs.[9] The Government of Pakistan is the major contributor to the funds for this program, external sources only contribute around 11% of the total amount.[9] The role of LHWs is pivotal in Pakistan's healthcare system. They have 15 months of training in MCH [2]. The LHW role in antenatal care includes: health promotion, hospital referrals, and basic neonatal care. Currently LHW is not responsible for immediate management of eclampsia /pre-eclampsia and refers such patients. LHW don't carry any antihypertensive medicine for pregnant woman, and don't have BP apparatus, their role was found to be limited 'to advice to seek care from nearest referral facility' in case if a pregnant woman complained about severe headache with dizziness. An external evaluation of the LHW programme concluded that it was effective in delivering family planning services and immunization services, in the management of diarrhoea, and, most recently through a cRCT, reducing the burden of stillbirth in Sindh Province [9;12].

Appendix B – relevant systematic reviews

Antihypertensives for severe hypertension in pre-eclampsia and the other HDP

Short-acting parenteral agents, such as i.v. hydralazine and labetalol, have been most widely studied in RCTs, although systematic reviews have failed to reveal clear differences between agents [60;61]. We undertook a comprehensive search for RCTs of oral antihypertensive therapy to assess the effectiveness for treatment of severe pregnancy/postpartum hypertension. Also, we reviewed additional relevant RCTs of oral antihypertensive therapy for severe hypertension outside pregnancy to supplement effectiveness data in pregnancy (Firoz, submitted).

In pregnancy and postpartum, we identified 15 trials in pregnancy (914 women), one of which was a postpartum trial (38 women). Trials were generally small with a median of 50 women (range 20 to150) and fair in quality. There was a wide range in the type of HDP at inclusion and gestational age at enrolment. When specified, the BP treatment goal was usually a dBP<100-110mmHg and the success of treatment was evaluated over a short time frame ranging from 20-120 min. Here we summarise the analyses that are relevant to CLIP.

Nifedipine

Twelve RCTs in pregnancy compared oral/SL nifedipine capsules (8-10mg, 12 trials, 724 women) with another agent. Most compared nifedipine with i.v. hydralazine (5-20mg, 7 trials, 350 women) or i.v. labetalol (20mg, 2 trials, 100 women). Other trials compared short-acting nifedipine to oral nifedipine 10mg PA tablets (1 trial), oral prazosin 1mg (1 trial)), or i.v./i.m. chlorpromazine 12.5mg (1 trial). The postpartum RCT (38 women) compared SL nifedipine with i.v. hydralazine.

When short acting nifedipine was compared with i.v. hydralazine in pregnancy, there was no difference in effectiveness as seen by achievement of target BP [84% (nifedipine) vs. 79% hydralazine; RR1.07 95% CI 0.98, .17; 5 trials, 273 women] or time taken to achieve it [WMD -1.36 hours, 95% CI -6.64, 4.14], and the number of doses needed [51% vs. 55%; RR 0.97 95% CI 0.50, 1.88; 4 trials, 246 women]. There was also no difference in maternal mortality [as no studies reported maternal deaths RD 0.00 95%CI -0.03, 0.03; 3 trials, 96 women] or maternal hypotension [1.6% vs. 0%; RD 0.00 95% -0.02, 0.03; 4 trials, 246 women]. There were no differences in perinatal outcomes including adverse fetal heart rate effects, stillbirth and perinatal/neonatal death.

When short-acting nifedipine was compared with i.v. labetalol (N=2 trials), results were similar and there was no difference seen in achievement of target blood pressure, number of doses needed or the requirement for additional antihypertensive therapy. There was no difference in maternal hypotension, maternal mortality and other adverse effects. Similarly, there were no differences in perinatal outcomes including caesarean section [RR 1.13 95% CI 0.76, 1.96; 1 trial, 50 women], stillbirth¸ and perinatal death [WMD 0.00 95% CI -0.07, 0.07; 1 trial, 50 women].

Nifedipine capsules (10mg orally), compared with nifedipine PA tablets (10mg orally), were associated with more maternal hypotension at 90 minutes [35% vs. 9%; RD 0.26, 95% CI 0.07, 0.46, 1 trial, 64 women]. The absolute rate of hypotension with nifedipine capsules in this trial (35%) was higher than that seen in other nifedipine capsule trials of similar dosage (8-10mg) where the rate of maternal hypotension was 0.8%.

Labetalol and methyldopa

There was a single trial (74 women) that compared oral labetalol 100 QID with oral methyldopa 250 mg QID. There was no difference in achieving target BP [47% vs. 56%; RR 0.85 95% CI 0.54, 1.33],

Caesarean section [50% vs. 59%; RR 0.85, 95% CI 0.56, 1.30], or perinatal death [5% vs. 0%; RD 0.05 95% CI -0.03, 0.14]. A three-arm trial compared oral methyldopa with either atenolol (50-200mg) or oral ketanserin (80-120 mg). This trial did not report on effectiveness in lowering BP. Perinatal outcomes did not differ between the groups including perinatal death [WMD -0.04 95% CI -0.10, 0.03; 2 trials, 192 women] and stillbirth [WMD 0.01 95% CI -0.03, 0.05; 2 trials, 192 women]

Other antihypertensive agents

One small trial (36 women) compared SL isosorbide with parenteral magnesium sulphate and found no difference between the groups with respect to additional antihypertensive therapy required but there was a significant difference in the rate of Caesarean section between the two groups [16% vs. 89%; RR 0.19, 95%CI 0.07, 0.53].

In summary, our results are similar to that of previous systematic reviews. The analysis of the evidence is complicated by the small numbers of patients, rare events as outcomes and variations in the drug administration regimens among other factors. We found that oral antihypertensive agents, particularly short-acting nifedipine, are a suitable option for treatment of severe hypertension in pregnancy/postpartum.

Oral antihypertensives in SEVERE NON-pregnancy hypertension

Thirty-four studies (1843 patients) met inclusion criteria. Generally, trials were small with a median of 36 patients (range 7 to 374). The dBP for inclusion in most studies was between 110-120 mmHg. When specified, the BP treatment goal was usually a dBP<100-110mmHg and the success of treatment was evaluated over a short time frame with between-trial variability in the time to reach the target BP ranging from 30 minutes to 24 hours. The quality of included studies was poor as there was an unclear risk of bias for sequence generation, allocation concealment, blinding, selective outcome reporting and incomplete outcome data.

Nifedipine

Eighteen trials (describing 21 comparisons) compared oral/SL (short-acting) nifedipine with either another antihypertensive or other doses/formulations of nifedipine. Compared with captopril, oral/SL nifedipine was associated with similar effectiveness in lowering BP: achievement of target BP at 60 minutes [88% (nifedipine) vs. 76% captopril; RR 1. 09 95% CI 0.87, 1.37; N=4 trials, 174 patients], number of doses of antihypertensive required [56% vs. 64%; RR 0.88 95% CI 0.56, 1.38; N=1 trial, 50 patients], or the need for additional antihypertensive therapy [50% vs. 70%; RR 0.71 95%CI 0.34, 1.50; N=1 trials, 20 patients]. The absolute rates of effectiveness were similar to that in pregnancy, but there was significant heterogeneity for achievement of blood pressure.

Only one trial evaluated hypotension and found that there were no episodes in either group. Also, there were no differences seen for other adverse effects including headache [4% vs. 10%; RR 0.86, 95% CI 0.16, 4.71; 4 trials, 145 patients] and cardiac arrhythmia [13% vs. 0%; RR 8.73, 95% CI 0.49, 155.62; 1 trial, 63 patients]. Nifedipine appeared to be associated with more flushing [13% vs. 0%; RR 6.75, 95% CI 1.26, 36.10; 3 trials, 133 patients].

In all other subgroups, nifedipine po/SL was compared with another drug or another dose/formulation of nifedipine in only one or two small trials that enrolled a median of 40 patients. No between-group differences in outcomes were seen.

Labetalol

Four trials (5 comparisons, 568 patients) compared oral labetalol to either: two other doses of oral labetalol (i.e., 100 mg vs. 200 mg vs. 300 mg/d)(66), either amlodipine or perindopril, clonidine, or nifedipine (as discussed above). There were no between-group differences in achievement of target BP or hypotension in

any of these comparisons. There was no difference in achievement of BP when 200 mg of oral labetalol was compared to 100 mg [58% vs. 75%; RR 0.78 95% CI 0.44, 1.39; 1 trial, 24 patients] and 300 mg [58% vs. 67%; RR 0.78 95% CI 0.44, 1.63; 1 trial, 24 patients] with no hypotension observed at either 100 mg, 200 mg and 300 mg doses.

Methyldopa

One trial (3 comparisons) compared oral methyldopa 500 mg with either oral captopril 50 mg, indapamide 2.5 mg, or placebo. Pre-treatment MAP in the methyldopa arm was 127.7 ± 6 mmHg and two hours after treatment, the MAP reduced to 119 ± 4 mmHg. This was comparable to the post-treatment MAP seen in the captopril (125.2 ± 13 mmHg), indapamide (119.3 ± 11 mmHg) and placebo (122.9 ± 12 mmHg) groups.

Other antihypertensive agents

There were one or two trials each of other comparisons including captopril versus place or other agents (methyldopa, indapamide, quinapril, telmisartan, nicardipine, urapidil), oral vs. aerosol isosorbide dinitrate, different doses of isradipine and ketanserin verus placebo or agents. These trials did not report on outcomes related to effectiveness but reported on adverse effects.

Our results are similar to that found in the 2009 systematic review by Souza *et al* with similar challenges included poor quality, small trials and few trials reporting outcomes of interest [62].

Summary

Based on RCTs in pregnancy/postpartum, oral nifedipine (10mg) is a suitable oral agent for treatment of severe hypertension in pregnancy/postpartum, with treatment success rates of at least 84%, low rates of maternal hypotension (upper 95% CI 3%), and similar maternal and perinatal outcomes compared with parenteral hydralazine or labetalol. Although it should be noted that the one 10mg nifedipine capsule vs. 10mg PA tablet trial that did report more hypotension with the capsule formulation found much higher rates of hypotension in *both* arms of the trial (35% in the capsule arm and 9% in the 10mg tablet arm) compared with the six other nifedipine capsule trials of similar dosage (<1%) [63]; also, that hypotension was not necessarily associated with adverse clinical effects.

The few, small comparative trials of other antihypertensive agents in pregnancy/postpartum preclude any firm conclusions. However, the limited data suggest that oral labetalol and methyldopa may be effective in approximately 50% of pregnant women, and each may be a reasonable alternative to nifedipine. Caution should be exercised if considering use of oral prazosin given its association with more Caesarean deliveries and, possibly, stillbirth.

The data from trials outside pregnancy lend further support to the effectiveness of short-acting nifedipine for treatment of severe hypertension (at least 88% success) without significant hypotension. Similar rates of success were also seen with SL captopril (76% success) with which nifedipine was most frequently compared. These data would suggest that oral captopril, which is acceptable for use in breastfeeding, should be considered for use postpartum in the absence of renal impairment.

To our knowledge, this is the first systematic review to specifically examine oral antihypertensive therapy for severe hypertension in pregnancy and postpartum. However, there are other meta-analyses of trials of short-acting parenteral agent or oral nifedipine in pregnancy/postpartum, and the results of the oral nifedipine vs. parenteral hydralazine subgroup are consistent with our analysis.

In the systematic review of oral antihypertensive therapy for severe hypertension outside pregnancy, captopril was associated with fewer minor side-effects than nifedipine [i.e., flushing (RR 0.22 95% CI 0.02, 0.72) and headache (RR 0.34 95% CI 0.13, 0,92)]; the difference in results may be explained by Souza *et*

al.'s inclusion of several Spanish-language papers as well as a study (that we excluded) of several patients with phaeochromocytoma.

Our review presents reasonable options for oral antihypertensive therapy. Options are key as there may be contraindications to use of a given drug (or women may already be on high doses of an oral agent when they present with severe hypertension). For example, there are published concerns about heightened cardiovascular morbidity/mortality associated with use of short-acting nifedipine outside pregnancy, and neuromuscular blockade with contemporaneous use of magnesium sulphate and nifepidine in pregnancy (although the risk was estimated to be <1% in a controlled study that incorporated data from RCTs [32]). The usefulness of beta-blockers may be limited in areas where reactive airways disease is prevalent and air quality is poor (such as in Pakistan).

$MgSO_4$ for the prevention and treatment of eclampsia

Prevention of eclampsia

A Cochrane systematic review of 15 RCTs investigated the relative effects of $MgSO_4$ and other anticonvulsants when used for prevention of eclampsia [3]. Notable comparisons in this review were between $MgSO_4$ and placebo or no anticonvulsants (six trials, 11, 444 women); phenytoin (four trials, 2345 women); diazepam (two trials, 66 women); and nimodipine (one trial, 1750 women). One small trial (36 women) compared $MgSO_4$ with isosorbide and another trial (33 women) compared magnesium chloride with methyldopa.

$MgSO_4$ versus placebo or no anticonvulsant Six RCTs (11,444 women) including the large multicentre RCT [64] that involved 10,141 participants provided the evidence related to this comparison. About half of the women recruited into the Magpie trial had the maintenance regimen for $MgSO_4$ through the intravenous route (1g/hr) and the other half through the intramuscular route. Maintenance was strictly by intravenous route for four trials and intramuscular for one trial. For most trials, clinical monitoring for potential adverse effects were reported and none of the six trials reported using serum monitoring of $MgSO_4$.

When compared with placebo or no anticonvulsant, $MgSO_4$ was associated with statistically and clinically significant reduction in the risk of eclampsia by almost 60% (six trials, 11,444 women; RR 0.41 [0.29, 0.58]). This effect was consistent for women who were antepartum at trial entry (six trials, 10,109 women; RR 0.40 [0.27, 0.57]) but non significant for those who were postpartum at trial entry (one trial, 1335, RR 0.54 [0.16, 1.80]). The effect was also consistent and more pronounced among women who were ≥34 weeks' pregnant (two trials, 6498 women; RR 0.37 [0.24, 0.59]) and those who had received no anticonvulsants prior to trial entry (three trials,10,086 women; RR 0.33 [0.22, 0.48]). It was consistent regardless of the route of administration for $MgSO_4$ maintenance.

No statistically significant differences were observed between $MgSO_4$ and placebo regarding the risks of maternal death, any serious maternal morbidity, respiratory arrest and toxicity as shown by respiratory depression and absent tendon reflexes and calcium gluconate administration. Any reported side effects were significantly more common among women treated with $MgSO_4$ rather than placebo (one trial, 9992 women; RR 5.26 [4.59, 6.03]). For the baby, no clear difference were observed in the risks of stillbirth or neonatal death, admission to NICU and Apgar score <7 at 5min.

$MgSO_4$ versus phenytoin $MgSO_4$ was compared with phenytoin for prevention of eclampsia in four RCTs (2343 women). Compared with phenytoin, $MgSO_4$ significantly reduced the risk of eclampsia (three trials, 2291 women; RR 0.08 [0.01, 0.60]). No statistical differences were observed between

the two groups in terms of stillbirth, neonatal death, Apgar score <7 at 5min and admission to NICU.

$MgSO_4$ versus diazepam A small trial involving 66 women compared $MgSO_4$ and diazepam for prevention of eclampsia. The sample size and the events recorded were too small for any reliable conclusions.

$MgSO_4$ versus nimodipine In the one trial comparing $MgSO_4$ with nimodipine (1650 women), there were fewer cases of eclampsia among women allocated $MgSO_4$ (RR 0.33 [0.14, 0.77]).

Treatment of eclampsia

$MgSO_4$ versus diazepam for women with eclampsia A Cochrane systematic review of seven RCTs, involving 1396 women, provided the evidence on the differential effects of $MgSO_4$ when compared with diazepam for the care of women with eclampsia [4]. Most women in the trials had eclampsia either before or after delivery and about half of them received an anticonvulsant before trial entry. All regimens used in the trials for both $MgSO_4$ and diazepam included loading and maintenance dose. $MgSO_4$ faired better than diazepam regarding priority maternal outcomes of death (seven trials; 1396 women; RR 0.59 [0.38, 0.92]) and recurrence of convulsions (seven trials; 1390 women; RR 0.43 [0.33, 0.55]). There were no statistical differences between the two drugs for any serious maternal morbidity or any of its proxies addressed in this comparison. Regarding fetal outcomes, no clear difference was demonstrated between the comparison groups for perinatal death and admission to NICU. $MgSO_4$ was associated with fewer cases of babies born with Apgar scores <7 at 5min (three trials, 643 infants; RR 0.70 [0.54, 0.90]).

Comparison of the two treatment groups according to route of administration of $MgSO_4$ maintenance showed that intramuscular maintenance significantly reduced the risks of maternal respiratory depression (two trials, 120 women; RR 0.30 [0.10, 0.13]) and maternal ventilation (two trials, 120 women; RR 0.20 [0.05, 0.88]) but no statistical difference for maternal cardiac arrest. The two contributing trials had moderate risk of bias, small sample size and few events and resultant imprecise observations.

$MgSO_4$ versus phenytoin for women with eclampsia Evidence related to the effects of $MgSO_4$ compared with phenytoin for care of women with eclampsia came from a Cochrane systematic review of six RCTs, involving a total of 972 women [5]. Most of the women had eclampsia before delivery and had received anticonvulsants prior to trial entry.

Compared with those treated with phenytoin, women treated with $MgSO_4$ were at reduced risk of recurrence of convulsions (six trials, 972 women; RR 0.34 [0.24, 0.49]), admission to intensive care (one trial, 775 women; RR 0.67 [0.50, 0.89]) and need for ventilatory support (two trials, 825 women; RR 0.68 [0.50, 0.91]). There were no statistically significant differences between the two treatment groups for maternal death, any serious maternal morbidity and the reported proxy outcomes for severe maternal morbidity. Babies born to women treated with $MgSO_4$, rather than phenytoin, were less likely to be admitted for NICU (one trial, 518 infants, RR 0.73 [0.58, 0.91]) but no clear differences was observed between the two treatment groups in the risks of perinatal death and Apgar score <7 at 5min.

$MgSO_4$ versus lytic cocktail for women with eclampsia The evidence on the differential effects of $MgSO_4$ compared with 'lytic cocktail' (usually a combination of chlorpromazine, promethazine and pethidine/meperidine) was derived from a Cochrane systematic review of three small trials involving a total of 397 women [6]. Compared with lytic cocktail, $MgSO_4$ was associated with significantly fewer

cases of maternal death (three trials, 397 women; RR 0.14 [0.03, 0.59]), recurrent convulsions (three trials, 397 women; RR 0.06 [0.03, 0.12]), coma for >24h (one trial, 108 women; RR 0.04 [0.00, 0.74]) and respiratory depression (two trials, 198 women; RR 0.12 [0.02, 0.91]). No clear differences were observed for any other proxy outcome for severe maternal morbidity. The risks of stillbirth and neonatal mortality were also similar between the two treatment groups.

Alternative regimens of $MgSO_4$ for treatment of pre-eclampsia and eclampsia Evidence related to the comparative effects of alternative $MgSO_4$ regimens for treatment of preeclampsia and eclampsia came from a Cochrane systematic review of six RCTs involving 866 women [49]. Two of the trials (451 women) compared regimens for eclampsia while the other four (415 women) compared regimens for pre-eclampsia. None of the trials used dosages shown to be effective in large RCTs demonstrating effectiveness of $MgSO_4$.

When loading dose alone was compared with loading dose plus maintenance regimen for women with eclampsia, one trial (401 women) showed no statistical differences in the critical outcomes of recurrent convulsions and maternal death and the proxy outcome for perinatal death, stillbirth. The loading dose employed in this trial was 4g intravenous (i.v.) plus 6g intramuscular (i.m.), while the maintenance was 2.5 g i.m. every 4h for 24h. The trial had very serious limitations in its quality and the observations were generally imprecise. A small trial (50 women) compared low dose (similar to the regimen above) with 'standard' regimen (4 g i.v. + 8 g i.m. as loading dose, then 4 g IM every 4 hours for 24 hours) for women with eclampsia). The only case of recurrent convulsion in the trial was reported among women treated with the low dose regimen thus generating highly imprecise and unreliable data for this critical outcome. No statistically significant difference was observed between the two treatment groups for admission to NICU and proxy outcomes of oliguria and any baby death.

One small trial (17 women) compared i.v. (2g hourly for 24 hours) and i.m. (5g. 4-hourly for 24 hours) maintenance regimens for women with pre-eclampsia. There was no case of eclampsia in either arm of the trials. The trial was too small to yield any reliable conclusions regarding other priority and proxy outcomes reported [$MgSO_4$ toxicity; renal failure; and stillbirth.

Three trials involving 398 women evaluated short versus 24h postpartum regimens for women with mild and severe pre-eclampsia or imminent eclampsia. Two of these trials, accounting for approximately two-thirds of the participants, were at low or no risk of bias while one was at moderate risk of bias. None of the women in both arms of these trials developed any of the critical outcomes addressed: eclampsia (two trials, 394 women); $MgSO_4$ toxicity (one trial, 196 women).

Summary of the trial evidence:

Therefore, we have identified that $MgSO_4$ is the agent of choice for the prevention and treatment of eclampsia – however, it has never been examined as a community-level intervention (either in isolation or as a package of care). In addition, we have identified that there is no clear choice of oral antihypertensive for the management of severe pregnancy hypertension – however, we are completing the relevant trial as part of the PRE-EMPT project in preparation for CLIP.

Appendix C – Community Engagement Mapping

Intervention Cluster (name/identifier)			
Activities related to:	Do current activities exist targeting this objective? Y/N<br>If yes, please describe current activities (what types of activities, for whom, how often, where, and who leads them)	Contact person	Source of information
Pregnancy-related complications			
General Maternal Health			
Household decision-making around pregnancy and childbirth (eg. husband's and mother in-law's permission to go to hospital when necessary)			
Transportation initiatives			
Fundraising, insurance schemes or other initiatives related to reducing the barrier of cost of transport and treatment			
Other activities at the individual, household or community level related to maternal health			

Appendix D – cHCP Working Protocol

THE CLIP (COMMUNITY LEVEL INTERVENTIONS FOR PRE-ECLAMPSIA) CLUSTER RANDOMIZED CONTROLLED TRIAL – cHCP WORKING PROTOCOL

SECTION 1: BACKGROUND

1.1 WHAT IS THE PROBLEM TO BE ADDRESSED?

The hypertensive disorders of pregnancy (HDP) are defined by high blood pressure in pregnancy. Globally, the HDP are the second leading direct cause of maternal death; every year the HDP are responsible for the death of an estimated 75,000 women and over 500,000 babies. Almost all of these deaths occur in Africa and South Asia and are preventable. Maternal deaths relate primarily to delays in triage (ability of care provider and women to identify who is severely ill and requires urgent care), transport (ability to get women to appropriate care when needed) and treatment (ability to provide appropriate treatment when care accessed).

The CLIP trial approach to reducing these delays involves task-shifting the monitoring for complications related to the HDP to community health care providers (cHCPs); a cadre of health workers that is currently available in low-resourced settings. The health workforce shortage is a significant global problem that is preventing many countries from reducing child and maternal mortality associated with HDP. Care of these pregnancy disorders previously relied on doctors and midwives in facilities. Treatment for HDP in facilities involves costly medication for reducing blood pressure and, because delivery is the only way to stop the disorder, it often requires emergency Caesarean section when the severity of the disorder is identified too late. By identifying those women and babies at highest risk of complications through community-level screening and monitoring, transportation and treatment can be targeted to those most in need and timed to allow induction of labour instead of Caesarean delivery. There are two key benefits for poor and vulnerable populations: first, at the individual level women are not suffering the cost and time away from their families for unnecessary referrals or interventions when safe, increased community surveillance would be appropriate; second, at the health systems level, moving monitoring from the facility to the community increases the potential for broad population based screening, and more efficient use of already burdened acute care facilities.

1.2 CLIP TRIAL HYPOTHESIS AND OBJECTIVES

Hypothesis

That implementing community-level evidence-based care will reduce pre-eclampsia-related maternal mortality and major morbidity.

Objectives

To reduce pre-eclampsia-related, and all-cause, maternal and perinatal mortality and major morbidity by 20% in intervention clusters in Ogun (Nigeria), Maputo and Gaza Provinces (Mozambique), Sindh (Pakistan) and in Karnataka (India).

1.3 DOCUMENT PURPOSE AND SCOPE

Purpose

This document provides full descriptions of daily CLIP activities to be performed by cHCPs in all intervention clusters.

Scope

This document covers the following cHCP activities:

- Collecting demographic information on new patients
- Measuring blood pressure
- Estimating gestational age using symphysis-fundal height measurement
- Measuring proteinuira
- Assessing symptoms of pre-eclamspia
- Administering treatment for severe hypertension (oral methyldopa)
- Administering treatment to prevent seizures (intramuscular $MgSO_4$)
- Providing recommendations for transport to hospital

SECTION 2: CHCP ANTENATAL VISIT PROTOCOL

The cHCPs will be encouraged to identify pregnant women in their area, will have pregnant women referred to them when identified by the surveillance team during regular surveillance cycles, and will provide CLIP assessment during regular antenatal visits that place women in one of three care trajectories: 1) usual antenatal care 2) non-urgent referral, or 3) urgent referral. The regular CLIP visits will occur: 4-weekly during pregnancy until 28 weeks, fortnightly from 28-35 weeks and weekly thereafter, within 24 hours of birth, and on approximately days 3, 7, and 14 after delivery.

This section provides a description of each component of a CLIP assessment. These steps are meant to be performed in addition to any regular antenatal or postnatal care processes routinely performed by the ccHCPc.

NOTE: CLIP visits should only occur after the woman being assessed has registered for the trial and provided informed consent for her participation.

Figure 9 provides a flow-chart outlining the assessment protocol and decision points used to generate recommendations for care.

2. FIRST VISIT PROTOCOL

Overview

Each CLIP assessment visit will first involve an assessment of obvious signs of maternal risk including significant vaginal bleeding, stroke, lack of consciousness and seizures. All visits will also include a component of education by the cHCP regarding warning signs and symptoms of pregnancy.

On the first CLIP visit all women will have their blood pressure and proteinuria measured and, if antenatal, will have an assessment of gestational age performed based on last menstrual period dates and symphysis-fundal height measurement. If the woman is found to be hypertensive (systolic BP≥ 140 mmHg) she will also be asked targeted questions about symptoms as described below.

2.1.1 Step 1: Observe for overt signs of maternal compromise

- **signs of unconsciousness**

cHCP will observe to determine if the woman is unconscious at the time of the visit. If a woman is found to be unconscious her family members should be asked to determine if she had been observed to show signs of seizure or stroke prior to losing consciousness.

> PLEASE NOTE: Any woman found unconscious at the time of the cHCP visit will not be evaluated as described in the remaining protocol. The immediate concern when a cHCP encounters an unconscious woman will be to position the woman on her left side by placing a rolled blanket or hard pillow under her right hip and lumbar area. Once the woman is positioned this way the cHCP should attempt to measure the unconscious woman's blood pressure (see 2.3.5) and treat with $MgSO_4$ if sBP≥ 160mmHg before urgently referring for follow-up at a CEmOC facility. While transport is being arranged the cHCP should ensure the woman is placed in the recovery position on her left side and have nothing placed in the woman's mouth.

- **signs of stroke**

cHCP will visually assess for obvious signs of stroke at the beginning of the visit. Signs of stroke include either paralysis of one side of the body or a report of recent sudden loss of vision.

- **signs of eclampsia**

cHCP will visually assess for obvious signs of eclampsia at the beginning of the visit. Signs of eclampsia are defined as a sudden, violent, uncontrollable contraction of a group of muscles or a subtler sign as a brief loss of consciousness.

- **signs of vaginal bleeding**

The cHCP will visually assess for obvious signs of significant vaginal bleeding at the beginning of the visit defined as antepartum vaginal bleeding with pain before the onset of labour or rupture of membranes

2.1.2 Step 2: Introduction and greeting

The cHCP should introduce her/himself and provide an acceptable introduction to the purpose of the visit, as defined by local ethics board. At this stage the cHCP will use the warning signs and symptoms of pregnancy pictoral aide to describe the intervention and provide information to the woman and her family about signs and symptoms to watch out for. The woman should be encouraged to seek care from the C-HCP or at a primary health centre should they note any of the warning signs or symptoms during the pregnancy.

2.1.3 Step 3: Wash hands

The ccHCPc should wash their hands before performing any assessments on the woman.

2.1.4 Step 4: Record demographic data

Demographic data will be collected at the first visit only. If the woman has already been registered for the study by the surveillance team, the first step will be to locatelocatelocate this woman based on her surveillance ID in the CLIP-POM tool database. This will ensure new evaluations of the woman being evaluated are added to any existing data collected through CLIP trial activities. If the surveillance ID is unknown or the woman cannot be found, this information along with all demographic data can be manually entered.

1 Name
2 Directions to household
3 Age of pregnant woman
4 Phone number of pregnant woman or head of household
5 History of previous deliveries

2.1.5 Step 5: Assess maternal status

Determine if the woman is antepartum (has not delivered yet) or postpartum (has delivered a baby recently).

PLEASE NOTE: Women who are visited for the first time postpartum are ineligible for inclusion in the trial and should be provided with routine postpartum care according to local protocols.

2.1.6 Step 6: Assess fetal status

cHCP asks:

- "Have you noticed fetal movements in the last 12 hours?"

2.1.7 Step 7: Measure proteinuria

1. Provide woman with small cup/bottle for urine sample collection
2. Pregnant woman urinates into a small cup provided, all samples should be "clean catch" and collected in a clean container
3. Immerse the dipstick completely in fresh urine and withdraw immediately, drawing edge along rim of container to remove excess
4. Hold dipstick horizontally before reading

*ccHCPsc should wear clean gloves when measuring proteinuria; gloves to be discarded when measurement complete

2.1.8 Step 8: Take blood pressure

* Let the woman rest (seated, no talking) for at least 5min before taking the first measurement.

1. Place cuff on either arm supported at heart level – on a table (or arm rest of the chair) with woman sitting with back against a chair, no tight clothing around upper arm, both feet on the floor, cuff 1-2cm above elbow.

**Women must remain still (i.e. no movement or talking) while the measurement is being taken for an accurate reading.

2. Turn on machine and inflate cuff by hand, the cuff will then deflate automatically. If the cuff has not been inflated to the correct pressure, the device will indicate this with a 'beeping' sound - inflate the cuff to 30mmHg higher than the previous inflation pressure. Try to keep the device as still as possible during cuff deflation or alternatively let it rest on the table during deflation.
3. Note measurement

4. Wait 1 minute (during which the woman should remain still i.e. no movement or talking) and repeat - all women will receive two blood pressure measurements; an average of the two readings will be taken.
5. If the second measurement differs significantly (>10mmHg) from the first, a third measurement is required. In this case the second and third measurements will be averaged to determine blood pressure.

***If the result is an error readout, repeat the above process

2.1.9 Step 9: Determine estimated date of delivery

1. Determine if ultrasound is available, if this information is available record it in the space provided.
2. Estimate LMP, if available record.
3. Measure symphysis-fundal height (SFH)
    1. Lie semi-recumbent on a firm surface
    2. Place tape at base of pubic bone
    3. Measure from pubic bone up to the top of the fundus and note measurement

    To be taken at every visit until 24 weeks gestation is identified. This measurement will be taken in all women hypertensive and non-hypertensive.

    The SFH will be used to estimate gestational age if an ultrasound is unavailable.

    **This will only be done at the first antepartum visits

2.1.10 Step 10: Assess warning symptoms of pre-eclampsia (if systolic blood pressure found to be ≥ 140 mmHg)

cHCP asks specifically about each warning symptom for pre-eclampsia:

"Are you experiencing the following?"

a. Headache: the patient has experienced any headache, as specifically stated to the community health care provider
b. Visual disturbances: the patient has experienced visual disturbances (blind spots, blurry vision, scintillations/flashing lights, transient blindness), as specifically stated to the cHCP
c. Chest pain: the patient has experienced chest pain or tightness, as specifically stated to the cHCP
d. Dyspnoea: the patient has experienced dyspnoea (difficult, laboured breathing or shortness of breath), as specifically stated to the cHCP
e. Abdominal pain: the patient has experienced abdominal pain (not described as right upper quadrant/epigastric pain), as specifically stated to the cHCP

NOTES

** all recording should take place in the cell phone as well as the woman's personal antenatal card and the cHCP log book

** this visit does not REPLACE an existing protocol for antenatal assessment but should be purely in addition to.

2.2 SUBSEQUENT VISIT PROTOCOL

Overview

Each CLIP assessment visit will first involve an assessment of obvious signs of maternal risk including significant vaginal bleeding, stroke, lack of consciousness and seizures. In addition, all visits will include a component of education by the cHCP regarding warning signs and symptoms of pregnancy.

On all subsequent CLIP visits (after the first visit) all women will have their blood pressure measured. If the woman is found to be hypertensive (systolic BP≥ 140 mmHg) she will also have her proteinuria measured and will be asked targeted questions about symptoms as described below.

2.2.1 Step 1: Observe for overt signs of maternal compromise

- **signs of unconsciousness**

cHCP will observe to determine if the woman is unconscious at the time of the visit. If a woman is found to be unconscious her family members should be asked to determine if she had been observed to show signs of seizure or stroke prior to loosing consciousness.

PLEASE NOTE: Any woman found unconscious at the time of the cHCP visit will not be evaluated as described in the remaining protocol. The immediate concern when a cHCP encounters an unconscious woman will be to position the woman on her left side by placing a rolled blanket or hard pillow under her right hip and lumbar area. Once the woman is positioned this way the cHCP should attempt to measure the unconscious woman's blood pressure (see 2.3.5) and treat with $MgSO_4$ if sBP≥ 160mmHg before urgently referring for follow-up at a CEmOC facility. While transport is being arranged the cHCP should ensure the woman is placed in the recovery position on her left side and ensure nothing is placed in the woman's mouth.

- **signs of stroke**

cHCP will visually assess for obvious signs of stroke at the beginning of the visit. Signs of stroke include either paralysis of one side of the body or a report of recent sudden loss of vision.

- **signs of eclampsia**

cHCP will visually assess for obvious signs of eclampsia at the beginning of the visit. Signs of eclampsia are defined as a sudden, violent, uncontrollable contraction of a group of muscles or a subtler sign as a brief loss of consciousness.

- **signs of vaginal bleeding**

cHCP will visually assess for obvious signs of significant vaginal bleeding at the beginning of the visit defined as antepartum vaginal bleeding with pain before the onset of labour or rupture of membranes

2.2.2 Step 2: Introduction and greeting

The ccHCPc should introduce her/himself and provide an acceptable introduction to the purpose of the visit, as defined by local ethics board. At this stage the ccHCPc will use the warning signs and symptoms of pregnancy pictoral aide to describe the intervention and provide information to the woman and her family about signs and symptoms to watch out for. The woman should be encouraged to seek care from the cHCP or at a primary health centre should they note any of the warning signs or symptoms during the pregnancy.

2.2.3 Step 3: Wash hands

The cHCP should wash their hands before performing any assessments on the woman.

2.2.4 Step 4: Assess maternal status

Determine if the woman is antepartum (has not delivered yet) or postpartum (has delivered a baby recently).

2.2.5 Step 5: Assess fetal status (if antenatal)

cHCP asks:

- "Have you noticed fetal movements in the last 12 hours?"

2.2.6 Step 6: Take blood pressure

* Let the woman rest (seated, no talking) for at least 5min before taking the first measurement.

1. Place cuff on either arm supported at heart level – on a table (or arm rest of the chair) with woman sitting with back against a chair, no tight clothing around upper arm, both feet on the floor, cuff 1-2cm above elbow.

**Women must remain still (i.e. no movement or talking) while the measurement is being taken for an accurate reading.

2. Turn on machine and inflate cuff by hand, the cuff will then deflate automatically. If the cuff has not been inflated to the correct pressure, the device will indicate this with a 'beeping' sound - inflate the cuff to 30mmHg higher than the previous inflation pressure. Try to keep the device as still as possible during cuff deflation or alternatively let it rest on the table during deflation.
3. Note measurement
4. Wait 1 minute (during which the woman should remain still i.e. no movement or talking) and repeat - all women will receive two blood pressure measurements; an average of the two readings will be taken.
5. If second measurement differs significantly (>10mmHg) from the first a third measurement is required. In this case the second and third measurements will be averaged to determine blood pressure.

***If the result is error readout, repeat the above process

PLEASE NOTE: If the woman has a systolic blood pressure <140 mmHg the visit is complete at this stage and no other CLIP assessments should be performed.

2.2.7 Step 7: Measure proteinuria (if systolic blood pressure found to be ≥ 140 mmHg)

1. Provide woman with small cup/bottle for urine sample collection
2. Pregnant woman urinates into a small cup provided, all samples should be clear catch and collected in a clean container
3. Immerse the dipstick completely in fresh urine and withdraw immediately, drawing edge along rim of container to remove excess
4. Hold dipstick horizontally before reading

*cHCPs should wear clean gloves when measuring proteinuria, to be discarded

2.2.8 Step 8: Assess warning symptoms of pre-eclampsia (if systolic blood pressure found to be ≥ 140 mmHg)

1. cHCP asks specifically about each warning symptom:
   "Are you experiencing the following?"
   a. Headache: the patient has experienced any headache, as specifically stated to the community health care provider
   b. Visual disturbances: the patient has experienced visual disturbances (blind spots, blurry vision, scintillations/flashing lights, transient blindness), as specifically stated to the community health care provider
   c. Chest pain: the patient has experienced chest pain or tightness, as specifically stated to the community health care provider
   d. Dyspnoea: the patient has experienced dyspnoea (difficult, laboured breathing or shortness of breath), as specifically stated to the community health care provider
   e. Abdominal pain: the patient has experienced abdominal pain (not described as right upper quadrant/epigastric pain), as specifically stated to the community health care provider

NOTES

** all recording should take place in the cell phone as well as the woman's personal antenatal card and the cHCP log book, if these final two documents are in common use

** this visit does not REPLACE an existing protocol for antenatal assessment but should be purely in addition to.

SECTION 3: cHCP PROTOCOL FOR TREATMENT

As outlined in the CLIP visit flow-chart, several clinical measures will trigger recommendations for treatment and transport to be performed by the cHCP. This section provides instruction on how to administer the oral antihypertensive treatment (methyldopa), intramuscular magnesium sulphate, how to give advice on recommendations for referral to a facility and how to follow-up once these treatment or transport recommendations have been provided.

3.1 METHYLDOPA ADMINISTRATION INSTRUCTIONS

In the CLIP trial, any woman found to have a systolic blood pressure reading of ≥160 mmHg will be recommended to receive 750 mg of the oral antihypertensive, methyldopa. The following instructions outline how to provide this medication to an eligible woman:

1. Open the oral antihypertensive medication container contained in the CLIP intervention box
2. Check for expiry date and any changes in colour, consistency etc. of the medication.
3. Read the label
4. Explain to the woman the purpose of administration is to reduce her blood pressure, which was found to be very high and puts her and her baby at risk of severe complications in her pregnancy
5. Allow woman to ask any question about drugs.
6. Offer water or juice to help swallow medication.
7. Provide woman with 3 methyldopa tablets of 250mg each
8. Stay with the woman to arrange transport to a recommended facility, if possible .
9. Complete the referral card by selecting all conditions experienced by the woman and medications given. This card should be given to the woman or her accompanying family to be brought to the facility with her.
10. Save the tablet strip in the same box with woman's name, date of administration and the other details

***Pregnant women should not receive oral medication if unconscious

3.2 MAGNESIUM SULPHATE ADMINISTRATION INSTRUCTIONS

In the CLIP trial, all woman who are found to be experiencing signs of recent seizure or stroke, who have a systolic BP ≥160 mmHg or a miniPIERS predicted probability ≥25% will be recommended to receive two, 5g intramuscular injections of magnesium sulphate, one in each buttocks. The following instructions outline how to administer this drug to an eligible woman:

1. Explain the reason and procedure briefly to the woman or attendant (as appropriate)
2. Wash hands
3. Put on gloves
4. Take the pre-prepared syringe from the medicine box
5. Check for expiry date and any change in colour, consistency etc
6. Attach needle to syringe and ensure needle is securely attached x2
7. Fill syringe with vial contents x2
8. Clean the injection site
9. Inspect skin surface for bruises, oedema or inflammation
10. Have patient assume a position appropriate for the site selected
11. Administer intra-muscularly in upper and outer quadrant of buttock x2

12. Discard needle and syringe in the designated disposal container
13. Apply pressure to the injection site for 2 minutes
14. Store the used syringe in the box with the name of the woman and reason for administration
15. Stay with the woman asasas long as possible to help arrange transport, accompany the woman if possible.
16. Complete the referral card by selecting all conditions experienced by the woman and medications given. This card should be given to the woman or her accompanying family to be brought to the facility with her.

.

**Normotensive women with vaginal bleeding should not be given $MgSO_4$

3.3 FACILITY REFERRAL INSTRUCTIONS

Women in the CLIP trial will be recommended to go to a facility within 4 hours of a visit for follow-up care if they meet any treatment criteria outlined in sections 3.1 and 3.2 above or if there have been no fetal movements felt within the last 12 hours. In addition, any woman who has a systolic blood pressure measured ≥ 140 mmHg but who does not qualify for referral within 4 hours will be recommended to go to a facility for follow-up care within 24 hours of the visit. The process of recommending referral should include the following steps:

1 Refer to health facility
2 Assist the woman and family to identify the reason for referral by completing the referral card and providing it to them.
3 Have a discussion with the family in order to raise awareness of complications and danger signs and receive permission for transport from the pregnant woman and/or the family decision maker
4 Contact the appropriate/available mode of transport for the transfer
5 Carry out and report the assessment of the woman at the time of referral (blood pressure, proteinuria, seizures, and other signs and symptoms like frontal headache, visual changes, right upper quadrant pain). Enable completion of appropriate documentation and feedback to referring practitioner.
6 Contact referral facility to notify of transfer and current condition of pregnant woman

Appendix E – Microlife BP 3AS2 Validation test results

1.1 Background

Accurate and regular blood pressure (BP) monitoring is a cost-effective screening tool for the early identification and management of pre-eclampsia in pregnancy. In low- and middle-income countries (LMICS), pre-eclampsia is frequently under-detected not only because attendance to antenatal care is often low, but due to inadequate training in how to take accurate blood pressure measurements, and insufficient, poorly functioning equipment. Firstly, training in the use of a cheap yet technically challenging method, the traditional sphygmomanometer is often lacking amongst health care workers staffing antenatal clinics. Secondly, there is concern over the robustness and safety of these traditional devices (those containing mercury), while the next generation aneroid instruments ideally require six-monthly re-calibration to maintain accuracy. Thirdly, the use of 'manual' BP devices such as these which require manual inflation, ausculatory skills and the observer recording the BP from a calibrated scale is associated with user-bias resulting in inaccurate measurement. This has been attributed to terminal digit preference, wrong Korrotkoff sound interpretation, threshold avoidance, and incorrect deflation speeds. Third generation BP devices which automatically inflate the cuff and provide a digital reading, whilst circumventing some of these issues, also have problems since they depend on a reliable power supply or source of batteries and have poor resistance to shock and fluctuations in temperature. In addition, for all devices, staff may not always be trained in the interpretation of the BP readings, especially in those settings where it is seldom taken due to lack of equipment.

1.2 The Microlife 3AS 1-2 Model

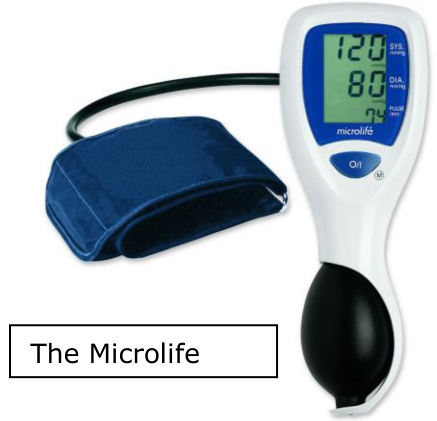

The Microlife

The Microlife 3As 1-2 semi-automatic handheld blood pressure device will be used to measure all blood pressures for participants enrolled in the CLIP cluster randomized control trial. It has been designed and validated specifically for use in developing countries by the CRADLE (Community Blood Pressure Measurement in Rural Africa: Detection of Underlying Pre-Eclampsia, Kings College London, PI: Professor Andrew Shennan). This small handheld device can be used effectively by unskilled personnel after minimal training. We have shown it to be easy to use, reliable and accurate. It can be manufactured at low cost and the manual pump cuff inflation (rather than battery operated) means that battery supply (2 AA alkaline batteries) is only used to power the liquid crystal display showing the BP reading, allowing it to generate 1000-1500 readings on one battery set, with a lifetime use of >20,000 cycles i.e. > decade with daily use (x5). This device was uniformly acceptable to clinic health-care workers in Tanzania (formative research, CRADLE project) and functional after 3 years of extensive use in harsh environments.

1.2.1 Validation of the device

Whilst many BP monitors are validated for general adult populations, few are validated in pregnancy. Common electronic BP monitors have significant difficulties in measuring during pregnancy, and are thought to significantly underestimate pressure by up to 50 mm HG. This means that even when measurements are taken with these devices, it may not be accurate in a pregnant population. The CRADLE research group, in collaboration with the Kimberly Hospital Complex, South Africa, validated the Microlife 3AS1-2 has for use in a pregnant population according to the requirements of the British Hypertension Society

(BHS) protocol in a South African population. A total of 45 pregnant women were recruited. The first 30 women to fulfil the BHS Protocol criteria were selected for the analysis of systolic and diastolic pressures respectively. The Microlife 3AS1-2 device achieved an overall B/A grade in the analysis of 45 pregnant women (Table 4). Overall, the device achieved the AAMI standard for mean difference and standard deviation (<5±8 mmHg) in pregnancy. Mean-against-difference plots are used to illustrate the data graphically for systolic (Figure 1) and diastolic pressures (Figure 2). The poorer performance in the higher blood pressures is consistent with all BP devices and does not preclude its recommendation for use in pre-eclampsia

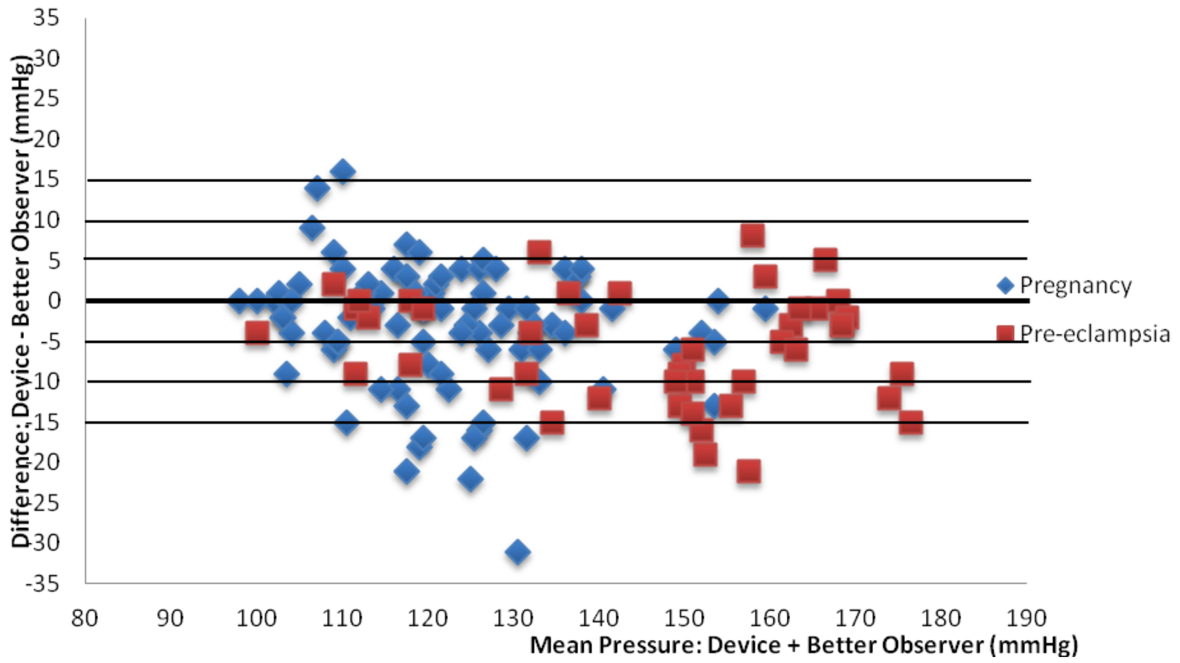

Figure 1. Mean-against-difference plot of the *systolic pressures* of the better observer and the device plotted against their difference in pregnancy and preeclampsia (n=135)

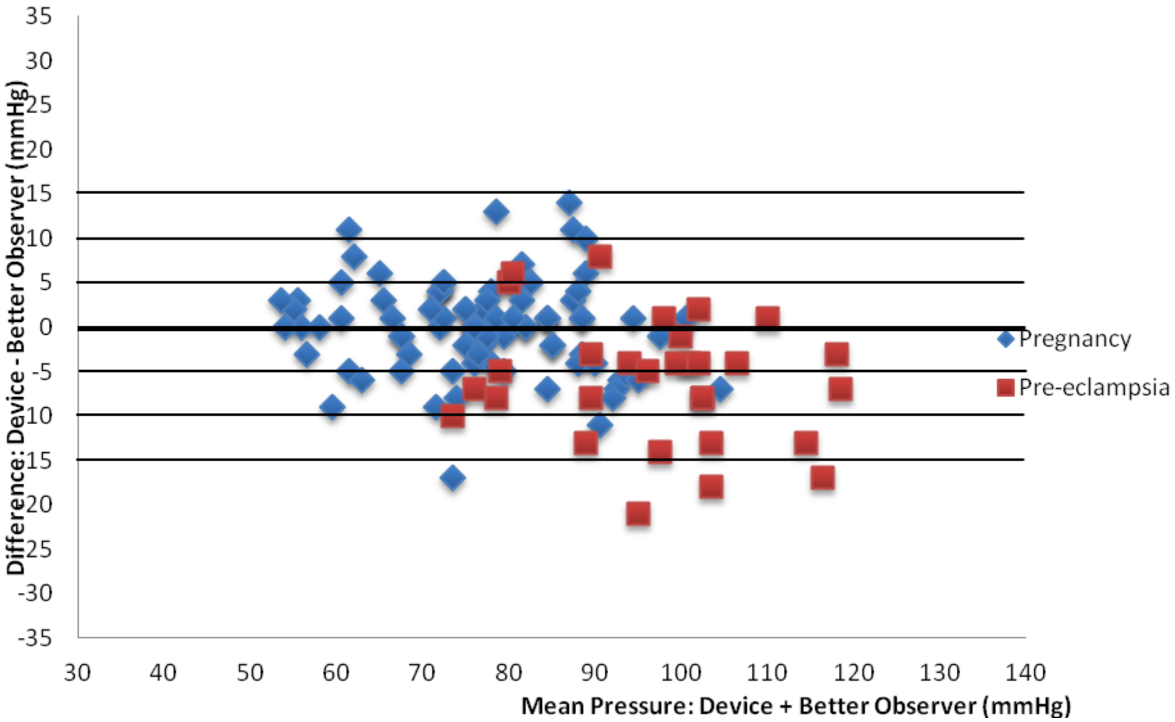

Figure 2. Mean-against-difference plot of the *diastolic pressures* of the better observer and the device plotted against their difference in pregnancy and preeclampsia (N-135)

1.2.2 Planned modifications to the device

CLIP and CRADLE research groups are currently working with our partners at Microlife to adapt this device to further suit the environment. We plan to equip the current handheld device with a micro-USB adapter (generic) to allow the device to be charged with generic mobile phone charging technology. Rechargeable batteries will be sealed in the device to prevent theft or loss. We plan to adapt the monitor to include a 'traffic light' approach to BP readings whereby readings above particular thresholds will be highlighted with red or amber LED displays to alert a provider to act on the result. A traffic light approach will greatly simplify the decision making process for community health care providers regardless of literacy or dialect spoken.

Blood pressure thresholds in pregnancy for identifying maternal and infant risk: a secondary analysis of Community-Level Interventions for Pre-eclampsia (CLIP) trial data - Appendix

CLIP Trial Protocol (Final)

The CLIP (Community Level Interventions for Pre-eclampsia) cluster randomized controlled trial

Section 1: Background

1.1 What is the problem to be addressed?

Pre-eclampsia remains a leading cause of maternal and perinatal mortality and morbidity [1]. It is a pregnancy-specific disease characterised by *de novo* development of endothelial dysfunction resulting in the most common diagnostic features, concurrent hypertension and proteinuria, sometimes progressing into a multiorgan cluster of varying clinical features (Figure 1) [1]. Poor early placentation is especially associated with early-onset disease [1]. Predisposing cardiovascular or metabolic risks for endothelial dysfunction, as part of an exaggerated systemic inflammatory response, might dominate in the origins of late onset pre-eclampsia [1]. Because the multifactorial pathogenesis of different pre-eclampsia phenotypes has not been fully elucidated, prevention and prediction are still not possible, thus symptomatic clinical management should be focussed on the prevention of maternal morbidity (e.g., eclampsia) and mortality.

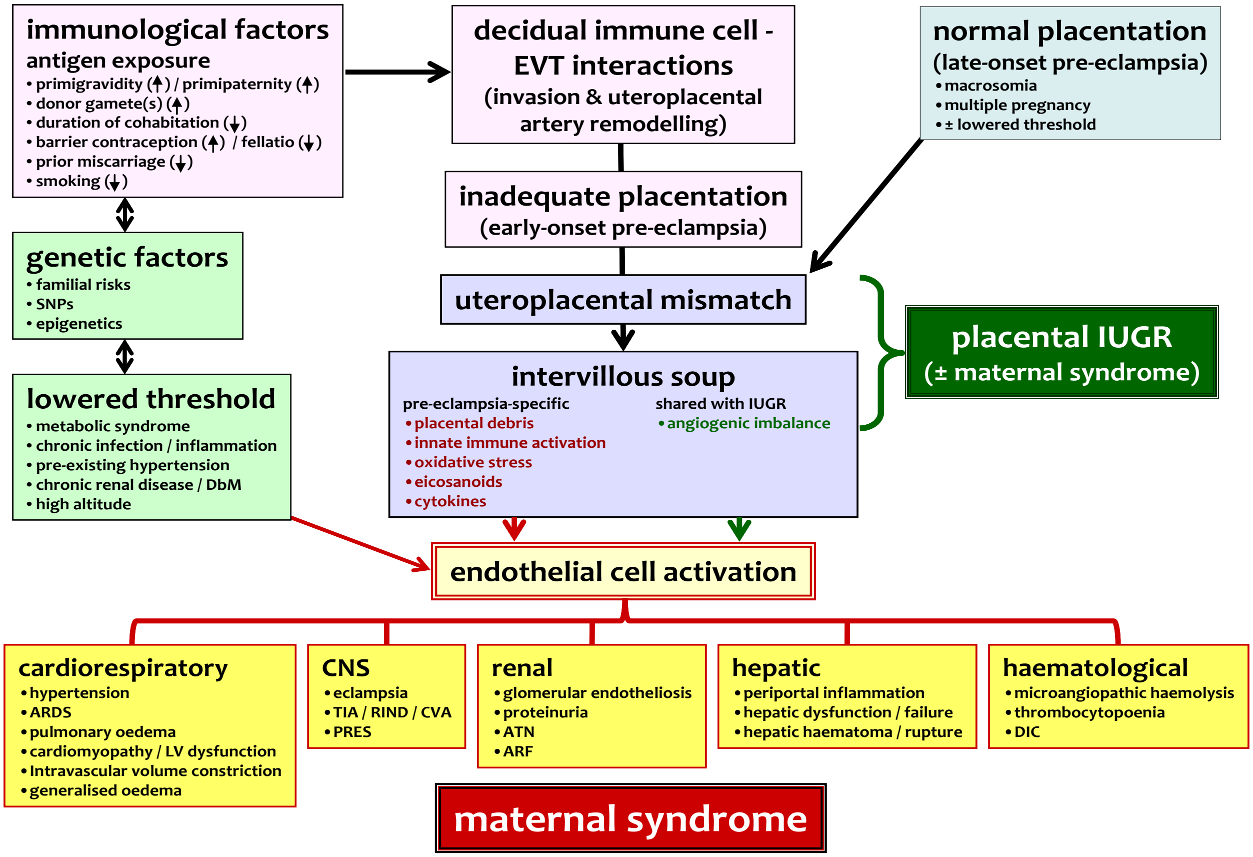

Figure 1 The origins and consequences of pre-eclampsia

ARDS acute respiratory distress syndrome; **ARF** acute renal failure; **ATN** acute tubular necrosis; **CVA** cerebrovascular accident; **DbM** diabetes mellitus; **DIC** disseminated intrasvascular coagulation; **EVT** extravillous trophoblast; **IUGR** intrauterine growth restriction; **LV** left ventricular; **PRES** posterior reservible leukoencephalopathy syndrome; **RIND** reversible ischaemic neurological deficit; **SNPs** single nucleotide polymorphisms; **TIA** transient ischaemic attack

Expectant management of women with early-onset disease to improve perinatal outcome should not preclude timely delivery - the only definitive step to initiate cure [1]. Pre-eclampsia foretells raised rates of

cardiovascular and metabolic disease in later life, which could be a reason for subsequent lifestyle education and intervention [1].

Globally, pre-eclampsia is the second-leading cause of maternal mortality, resulting in an estimated 76,000 maternal deaths annually. In addition, 500,000 fetal and newborn lives are lost annually due to the perinatal consequences of pre-eclampsia. Over 99% of these deaths occur in low and middle income countries (LMICs), primarily in South Asia and Sub-Saharan Africa [2].

As a result of the focus on postpartum haemorrhage (PPH; the leading cause of maternal death) over the past decade, rates of PPH-related maternal mortality are falling. This fall in PPH-related maternal mortality is driving the improvements in the maternal mortality ratio (MMR; maternal deaths per 100,000 live births) observed in countries such as Bangladesh and Pakistan. Therefore, the proportional contribution of pre-eclampsia-related mortality to the MMR is increasing. It may be that some of the residual PPH-related mortality arises from the consumptive coagulopathy of severe pre-eclampsia, especially when complicated by abruption [1].

Clearly, improvements in PPH-related outcomes have no direct impact on perinatal mortality as the woman is delivered before she bleeds. However, we recognise that maternal death places infants at a 6-fold increased risk of infant mortality.

In general, previous research in this field has focussed on institutional level interventions with $MgSO_4$ (eclampsia prevention and treatment [3-6]) and the treatment of severe pregnancy hypertension [7]. However, if we limit ourselves to studying inpatient, facility-level, interventions with fully assessed treatment options, many women will die or be irreversibly affected by pre-eclampsia (e.g., either moribund or having suffered a stroke) prior to arriving at the inpatient facility.

Of all the Millennium Development Goals (MDGs), MDG 5 (a 75% reduction in maternal mortality from 1990 levels by 2015) is that which is proving hardest to achieve. Maternal lives lost from pre-eclampsia and eclampsia result from delays in triage, transport and treatment. As such, the solution has to reside in getting care to women in the community as current health systems either never see affected women (who die either in their community or en route to formalised care) or receive women who are moribund and beyond salvage regardless of the quality of care being offered.

Currently, Bangladesh is one of the few countries on target to achieve MDG 5; however, the remaining issues to be addressed (such as pre-eclampsia) will be harder to achieve – partly because women with pre-eclampsia feel well until they are critically ill. It is probable that in Nigeria, Mozambique, Pakistan and India, MDG 5 will not be achieved.

The CLIP trial is a singular step towards addressing the excess maternal and perinatal mortality that derive from the failure to identify and rapidly manage pre-eclampsia and eclampsia at the community level in LMICs.

1.2 What are the principal research questions to be addressed?

Hypothesis

That implementing community-level evidence-based care will reduce pre-eclampsia-related maternal and perinatal mortality and major morbidity by addressing 'three delays' in triage, transport, and treatment.

Objective

To reduce pre-eclampsia-related, and all-cause, maternal and perinatal mortality and major morbidity by 20% or more in intervention clusters in Ogun State (Nigeria), Maputo and Gaza Province (Mozambique), Sindh Province (Pakistan) and in Karnataka State (India).

1.3 RELEVANT CONVERGENT ACTIVITIES

Pre-requisite knowledge: CLIP Feasibility Study *(co-PIs: P von Dadelszen & R Qureshi)*

The Feasibility Study is being conducted in Nigeria, Mozambique, Pakistan, and India in preparation for the CLIP cluster randomised controlled trial (cRCT; CW UBC REB # H12-00132). In Pakistan we are building on the previous community- and primary health centre (PHC)-level perinatal intervention studies and trials led by Professor Bhutta [8-12]**.** A process evaluation may be conducted in all the sites to provide information on mechanisms of impact and delivery of intervention.

The purpose of each national CLIP Feasibility Study is to understand the health care system, explore key methodological issues for CLIP, and engage in a dialogue with the key stakeholders (to inform the development of culturally appropriate tools and educational materials for use in CLIP).

The *health care system organisation and infrastructure capacity* must be clear, related to antenatal care models and pre-eclampsia/eclampsia monitoring, triage, management, referral and maternal transfer, and PHC and referral facility pre-eclampsia/eclampsia treatment.

Key methodological issues for CLIP include: community demographics, pre-eclampsia/eclampsia prevalence rates and rates of associated maternal and perinatal morbidity and mortality; data collection methods and informational systems for population surveillance; specific barriers to conducting a cRCT, including recruitment feasibility, capacity to implement community intervention, and accurate data collection; and cost identification to conduct the CLIP trial and an adequately powered cRCT in the identified country.

Finally, we must receive input from *key community stakeholders* (cultural and/or community beliefs/practices/influences/attitudes), *community-based health care providers (cHCP)* (professional scope of practice regulations and/or legal barriers and potential for task shifting), *facility care providers* (provider knowledge and competency related to pre-eclampsia/eclampsia and resource/informational capacity for provider training), and *key decision-making stakeholders* (support, commitment, and financial/schedule feasibility to remedy identified barriers). By engaging relevant stakeholders from the beginning of the trial planning process, we will work towards long term sustainability of the intervention, should it prove to be effective.

Each national Feasibility Study is using a mixed methods approach (quantitative, participatory, and formative, with community mapping) based on the normalization process model and will utilize literature reviews, target interviews, focus groups, and survey tools. Target interviews and focus group data are being recorded and transcribed; observations and assessments written up as field notes. The core approach is similar across the four study sites, but will allow for tailoring according to individual setting and cultural context. We are drawing on models of how interventions are embedded in practice (e.g., the normalisation process [13-15] and psychological theory [16]) as frameworks for this assessment.

A summary of key findings from the Feasibility Studies to date, including stakeholders engaged and information on health care system organisation and current scope of practice of cHCPs can be found in Appendix A of this document.

Tools & Funding: PIERS On the Move (POM) *(co-PIs: JM Ansermino & P von Dadelszen [funded by Saving Lives at Birth])*

Risk stratification using the miniPIERS model aims to address three delays that lead to the increased incidence of maternal mortality in LMICs: delays in triage, transport, and treatment (adapted from Thadeus and Maine)[17].

The *delays in triage* relate to the presentation of women late in the clinical course of their hypertensive disorder of pregnancy (HDP) and the failure to have the nature and potential severity of their condition recognised. In many settings, women receive no antenatal care; therefore achieving some degree of practical oversight and getting a triage mechanism into the hands of women and their immediate caregivers is a priority. Often, immediate care is given by cHCPs and at PHCs. Once women enter the formal health care system [hospitals providing either basic or comprehensive emergency obstetric care (EmOC)], the miniPIERS and fullPIERS models will identify those most at risk of adverse outcomes so that they can receive appropriate interventions in the timeliest manner possible [18;19].

To identify women for whom *transport* is a particular priority, cHCPs and the nurses, medical assistants, and physicians staffing PHCs need to have available to them tools that risk stratify women with pre-eclampsia; the miniPIERS model will do this.

Testing the *hypothesis* that the PIERS models, based on a combination of maternal and fetal predictors, will accurately identify women at incremental risk of maternal complications of pre-eclampsia, we have developed the fullPIERS model (see below) in eight tertiary academic centres in Canada, United Kingdom, New Zealand, and Australia[19]; and developed and validated the miniPIERS model in five LMICs (see below). The fullPIERS model identifies risk most accurately over the following 48h, but also for up to 7 days. The miniPIERS model performs with less accuracy, but may have greater impact as it is more generalisable to all levels of care in high, middle, and low income settings. Only miniPIERS will be utilised as a triage tool in the CLIP trial, as we anticipate that a model solely derived from, and validated in, LMICs will be the most accurate and have greatest validity for the CLIP trial.

The fullPIERS model (Figures 2 & 3) In the fullPIERS data set, we have identified those women with pre-eclampsia who are at increased risk of maternal complications and have been able to grade this risk [18-28]. The independent predictors of adverse maternal outcome are: gestational age at eligibility, chest pain/dyspnea, SpO_2, creatinine, aspartate transaminase (AST), and platelet count. fullPIERS assesses risk up to 7 days after eligibility (AUC ROC 0.76 [95% CI 0.72, 0.80] [19].

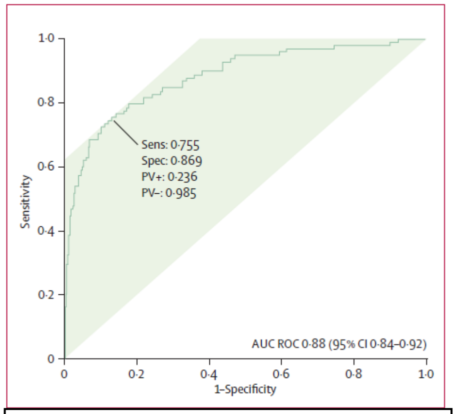

Figure 2: Performance of the fullPIERS model

Combined adverse maternal outcomes predicted within 48 h of eligibility on the basis of only data recorded before the outcome (an online tool to calculate fullPIERS probabilities is available on the study website). AUC ROC=area under the curve of the receiver operating characteristic. PV–=negative predictive value. PV+=positive predictive value. Sens=sensitivity. Spec=specificity.

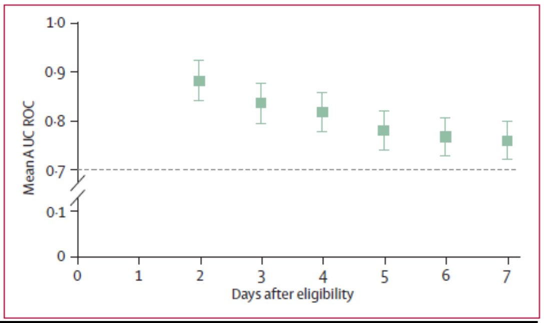

Figure 3: Mean fullPIERS AUC from 2-7 days

Error bars show 95% CIs. AUC ROC=area under the curve of the receiver operating characteristic.

An ancillary, and cost neutral, benefit of this project will be to externally validate the fullPIERS model in LMIC settings.

The miniPIERS model (Figures 4 & 5) Based on data from 2081 women admitted to a miniPIERS centre in Brazil, South Africa, Uganda, Pakistan and Fiji, the miniPIERS model predicts adverse maternal events in women with any HDP. The components of the miniPIERS model are: parity (muliparity vs. nuliparity)

gestational age at identification; symptoms (Y/N) of headache/ visual disturbances, chest pain/ dyspnoea, or abdominal pain with vaginal bleeding; systolic blood pressure (sBP); and dipstick proteinuria. The AUC ROC is 0.8788 [95% CI 0.4744, 0.80], decreasing to 73 on internal validation.

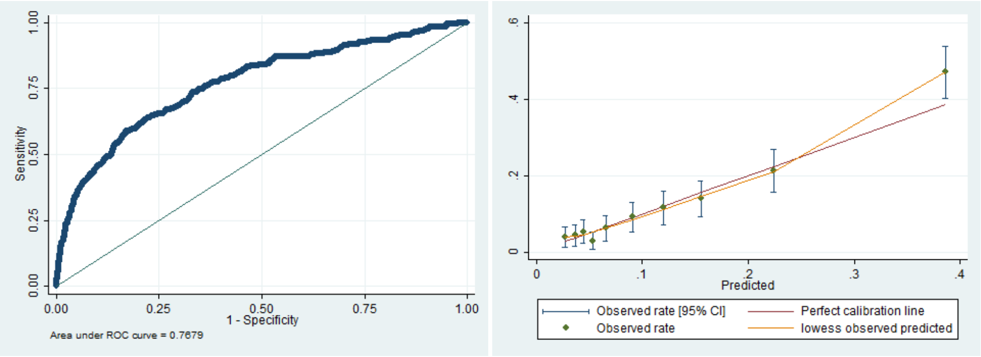

Figures 4 & 5: miniPIERS model ROC curve and calibration curve

Using funds from within the wider PRE-EMPT grant, miniPIERS modelling (development and validation) was completed in September 2012. The final model is a prerequisite for development of the management algorithms for the CLIP cRCT. miniPIERS will be a component of the CLIP trial hosted on a mobile platform.

In Mozambique and Pakistan an additional component to the miniPIERS mobile phone application will be the use of a low-cost mobile phone adapted pulse oximetry sensor to measure blood oxygen saturation (SpO_2) in the pregnant population under study. This sensor is being provided by our industry partner LionsGate Technologies (LGT) Medical. LGT Medical developed and manufactures a low-cost audio-port enabled pulse oximetry sensor, called the audio oximeter, for use with mobile phones and tablets. The raw photoplethysmogram (PPG) will be recorded to facilitate the optimal estimation of hearth rate, heart rate variability, pulse pressure variation, oxygen saturation and other parameters that may be used to optimize future signal quality estimates and to develop novel methods to characterize and display the information contained in the PPG. These waveform characteristics may be combined with other waveform or clinical information.

The raw pressure waveforms will be recorded during the measurement of blood pressure to estimate the optimal thresholds for future therapeutic decisions, to optimize future signal quality estimates and to develop novel methods to characterize and display the information contained in the non-invasive pressure waveform. These waveform characteristics may be combined with other waveform or clinical information

Using data from both the miniPIERS and fullPIERS studies we have previously demonstrated that blood oxygen saturation <93% is associated with significantly increased risk for the mother (OR fullPIERS: 18.0 95% CI 8.1 – 40.1; miniPIERS: 30.7 95% CI 13.9 - 67.7). Addition of SpO_2 as a variable in the miniPIERS model significantly improves the model sensitivity and has the potential to further improve risk stratification in the CLIP trial. Testing this modified model in two countries will allow us to determine if the added value in model sensitivity translates to real improvements in maternal outcomes compared to use of the original miniPIERS triage tool.

The miniPIERS mobile phone application to be used by cHCPs in the CLIP trial: (i) provides local, rapid and accurate risk assessment, referral, and treatment advice for pre-eclampsia, and (ii) transmits information to referral centres for co-ordination of triage, transportation, and treatment.

By harnessing the processing and battery power of the phone and widespread availability of cellular services

in Africa and South Asia, we will empower cHCPs to rapidly and reliably assess a woman's risk of pre-eclampsia complications in real-time, and to take action before complications arise.

Tools & pre-requisite knowledge: Blood pressure monitoring in LMICs (PI: A Shennan)

Using parallel funding from the Bill and Melinda Gates Foundation (BMGF), and building on two decades' experience, we have identified the optimal automated sphygmomanometers for use in LMIC settings, the Microlife BP 3AS1-2 and the Microlife CRADLE VSA, developed for Professor Shennan's parallel CRADLE (Community blood pressure monitoring in Rural Africa: Detection of underLying pre-Eclampsia) research programme in South Africa and Tanzania. Through his relationship with Microlife, Professor Shennan has arranged for us to bulk purchase 900 machines that have been purpose-modified for CLIP. See Appendix E for details on the validation study for the Microlife BP 3AS-2 and the Microlife CRADLE VSA.

As part of our collaboration with Microlife and Dr. Shennan, we will undertake qualitative analysis relating to the use of this tool in CLIP. Plans for qualitative evaluation fall within the CRADLE research programme and are funded separately from CLIP. Qualitative analysis plans are outlined in Appendix E of this protocol.

Pre-requisite knowledge: Oral antihypertensive therapy for severe pregnancy hypertension - review (PI: LA Magee)

Traditionally, severe hypertension (usually defined as sBP≥160mmHg and/or dBP≥110mmHg) has been treated with short-acting parenteral antihypertensive agents, most frequently, intravenous (i.v.) hydralazine or labetalol [29]. Parenteral agents require more resources than do oral antihypertensive agents, in terms of equipment (i.e., i.v. tubing, syringes, and needles) and personnel (as administration is by nurses or doctors). Also, parenteral agents require more monitoring and supervision as they are rapidly-acting and have the potential to lower BP quickly and cause maternal hypotension and fetal compromise.

Oral antihypertensive therapy is used for hypertensive urgencies in pregnancy in some jurisdictions, with apparently good effect. In the regional pre-eclampsia guidelines from Yorkshire, UK, labetalol 200 mg is administered orally before i.v. access is secured, with a repeat dose given if no response is seen after 30 minutes [30]. The UK National Institute for Health and Clinical Excellence (NICE) Hypertension in Pregnancy guideline recommends oral labetalol or nifedipine for the treatment of severe hypertension in critically ill women during pregnancy or after birth [31] .

In our literature review of oral antihypertensive therapy for severe hypertension in and outside pregnancy, we identified 15 RCTs (915 women) in pregnancy, one postpartum and 34 outside pregnancy (Firoz *et al*; manuscript in preparation).Most trials in pregnancy compared oral/sublingual (SL) nifedipine capsules (8-10mg) with another agent, usually parenteral hydralazine or labetalol. 84-100% of women achieved successful treatment with nifedipine with less than 1% experiencing hypotension. Target BP was achieved ~50% of the time with oral labetalol and methyldopa, suggesting that each may be a reasonable alternative to nifedipine. Outside pregnancy, most trials compared short-acting nifedipine with SL captopril (6 trials, 251 subjects) with similar rates of success (88% vs. 76%). The results are presented in detail in Appendix B.

In our review of the pharmacokinetics of antihypertensive agents found commonly on the essential medicines lists of LMICs (Lalani *et al. JOGC*), the onset, peak, and duration of action of agents were very similar (Table 1).

Table 1 Oral antihypertensives for severe pregnancy hypertension

Drug	Dosage	Onset	Peak	Duration
Atenolol	25 – 50 mg	1hr	2-4hr	24hr (dose dependent)
Labetalol	200 mg	20min – 2hr	1-4 hr	8-12hr (dose dependent)

Methyldopa	500 mg – 2 g	40 min	3-6hr	12-24hr
Nifedipine intermediate-acting	10 mg	30min	4hr	12hr
Nifedipine capsule	5 -10 mg	5-10min	30min	6.5hr

In summary, 750mg (alpha-)methyldopa has been chosen as the optimal antihypertensive to treat severe pregnancy hypertension in CLIP for several reasons: (i) it has been well-studied in RCTs in pregnancy for non-severe hypertension, and more limited RCT data for severe hypertension suggest that the drug is effective; (ii) it has an acceptable onset, peak, and duration of action for treatment of severe hypertension; (iii) there is a high level of comfort with methydopa due to its long history of use as an antihypertensive of first choice in pregnancy, making it an acceptable choice to most practitioners; (iv) it is likely to face fewer barriers in terms of acceptability during implementation as it does not interact with $MgSO_4$, whereas unfounded misconceptions persist about a nifedipine-magnesium interaction [32]; (v) it is widely available on nearly all LMIC essential medicines lists, and it is affordable, which speaks to feasibility during post-trial implementation and scale-up; and (vi) as an oral treatment it is more acceptable for administration by cHCPs than complex intravenous medications.

Pre-requisite knowledge: Options for safe community administration of $MgSO_4$ *(PI: LA Magee)*

We have reviewed the current literature that pertains to intramuscular (i.m.)-only administration of a $MgSO_4$ loading dose at the community level (Gordon *et al*; JOGC [in press]). We have determined that a single 10g i.m. dose of $MgSO_4$ would be safe (even in the presence of anuria) and would achieve therapeutic Mg^{2+} levels within 45 minutes and result in sustained levels for 4-6 hr (Figure 6) [33]. A more thorough description of the results of this systematic review is provided in Appendix B.

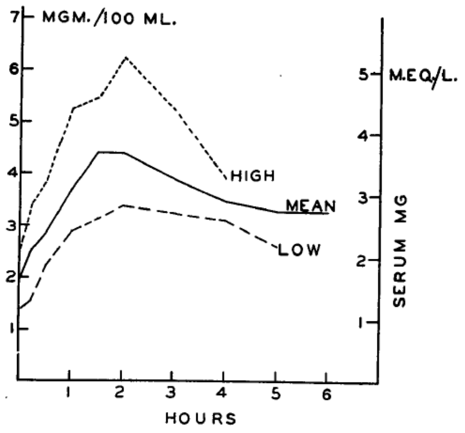

Figure 6 Effect of 10g IM $MgSO_4$ loading dose on serum Mg^{2+} concentration

Pre-requisite knowledge: Current CLIP-related maternity services provision in Nigeria, Mozambique, Pakistan & India *(PI: P von Dadelszen)*

We have reviewed the current availability of CLIP-relevant personnel, drugs, devices and interventions at all levels of the public, NGO and private health care systems in Nigeria, Mozambique, Pakistan and India.

**Table 2** CLIP-related maternity care service provision	**Nigeria**	**Mozambique**	**Pakistan**	**India**
**Community-based care**				
**HOME-BASED CARE**				
**cHCPs**	**Community Health Extension Workers (CHEWs; cover 2500 population each)**<br>**Health Assistants (HAs)**	**Agente Communitarios de Saude (APEs; cover 500-2000 population each)**	**Lady Health Workers (LHWs; cover 1000 population each)**	**Auxiliary Nurse Midwives**<br>**(ANMs; cover 3000-5000 population each)**<br>**Accredited Social Health Activists (ASHAs; cover 1000**

**Table 2** CLIP-related maternity care service provision	**Nigeria**	**Mozambique**	**Pakistan**	**India**
				**population each)**
**Delivery in the home**	**70% births at home**	**30% births at home**	**40% births at home**	**5-10% births at home**
**PHC-based care**				
**Medical officers**	**Sometimes**	**No**	**Yes**	**Yes**
**Nurses**	**Yes**	**Yes**	**Yes**	**Yes**
**cHCPs**	**CHEWs, Health Assistants (HA)**	**ACSs**	**LHWs**	**ANMs, Lady Health Visitors (LHV)**
**BP device**	**Yes (93%)**	**Yes**	**Yes (100%)**	**Yes (100%)**
**Urine dipsticks**	**Sometimes (20%)**	**Yes**	**Few (37%)**	**Yes (90%)**
**Laboratory testing**	**Sometimes (11%)**	**Some**	**Yes (100%)**	**No**
**Ultrasound**	**Sometimes (5%)**	**No**	**Sometimes**	**No**
**Methyldopa**	**Yes (purchased by patient at pharmacy)**	**Yes**	**Yes**	**Yes**
**MgSO4**	**Availability of some anticonvulsant (82%)**	**Yes**	**Sometimes (38%)**	**Sometimes (56%)**
**Attend deliveries**	**Yes**	**Yes**	**Yes**	**Yes**
**BEmOC**	**Varies**	**No**	**Yes**	**No**
**Hours of available care**	**Information pending**	**Information pending**	**Day time only**	**Some services are 24/7**
**Facility-based care**				
**Number of facilities serving CLIP clusters**	**936**<br>**(32 public, 904 private)**	**56**	**12**<br>**(3 public, 9 private)**	**96**
**BEmOC in CLIP clusters**	**Information pending**	**10**	**17**<br>**(14 public, 3 private)**	**Information pending**
**CEmOC in CLIP clusters**		**1**	**12**	**Majority**
**General practitioners/ medical officers**	**Yes**	**Yes**	**Yes**	**Yes**
**Specialists**	**Rarely**	**Rarely**	**At some highest level facilities**	**Yes**
**Nurses**	**Yes**	**Yes**	**Yes**	**Yes**
**cHCPs**	**Yes**	**Yes**	**No**	**ANMs**
**BP device**	**Yes**	**Yes**	**Yes (100%)**	**Yes (100%)**
**Urine dipsticks**	**Yes**	**Yes**	**Sometimes**	**Yes**
**Laboratory testing**	**Yes**	**Yes**	**Yes (100%)**	**Yes**
**Ultrasound**	**Yes**	**Yes**	**Yes**	**Yes**
**Fetal heart assessment**	**Pinard; US**	**Pinard, Doppler, US**	**Pinard, CTG, US**	**Pinard, Doppler, CTG, US**
**Methyldopa**	**Sometimes (30%)**	**Yes**	**Yes**	**Yes**
**Other antihypertensives**	**Sometimes (7%)**	**Yes**	**Yes**	**Yes**
**MgSO4**	**Sometimes (20% stock outs)**	**Yes**	**Some**	**Yes**

ANM auxilary nurse midwife; **APE** agente polivalente elementares; **ASHA** accredited social health advocate; **BEmOC** basic emergency obstetric care; **BP** blood pressure; **CEmOC** comprehensive EmOC, **cHCP** community health care provider; **CHEW** community health extension worker; **CTG** cardiotocograph; **LHW** lady health worker; **MO** medical officer; **PHC** primary health centre; **US** ultrasound

Section 2: The CLIP trial

2.1 The CLIP trial design

We have designed a two-phased community (including PHC-level) cRCT encompassing both rural and urban settings to be fully powered in each of:

- Ogun State, Nigeria
- Maputo and Gaza Province, Mozambique
- Hyderabad and Matiari districts in Sindh Province, Pakistan.
- Belgaum and Bagalkot districts in Karnataka State, India

The trial will be phased from the Pilot CLIP trial to Definitive CLIP trial on the basis of a satisfactory rate of use (≥50%) of the CLIP 'package of care' in appropriate women n all countries but Mozambique (see section 2.2 intervention for details on the 'package of care'). Mozambique will be unique in that they will rely on an extended period of feasibility to pilot test all Trial systems and tools before directly beginning a definitive trial. Foregoing the Pilot in Mozambique was felt to be appropriate based on their experience with community-based surveillance and will ensure timelines of the trial are met within a manageable budget.

For all other countries, use of the package in the Pilot phase will be defined as appropriate referral (urgent or non-urgent) to a facility able to provide comprehensive emergency obstetric care (CEmOC) in appropriate women during the first six months of the Pilot CLIP trial (Figure 2). This accelerated transition from Pilot CLIP trials to Definitive CLIP trials will: (i) save resources in the long term as the research infrastructure will be maintained in all countries and, thereby, start-up costs will be reduced as will core infrastructure costs at UBC, and (ii) deliver more rapidly the primary CLIP research question, does the CLIP community intervention improve outcomes for mothers and babies?

Full details of the sample size calculation for both Pilot and Definitive trial phases can be found in Table 3.

Monitoring during the Pilot phase of the trial to determine rate of use of the CLIP package of care will be performed by the cHCPs using the POM mobile application in an ongoing manner after the Pilot trial begins. In the Pilot Trial, if the percentage use of the CLIP 'package of care' is <50% in any given jurisdiction at 6 months after the trial start date, but climbing, we plan to re-assess at 7, 8, and 9 months before making a decision whether or not to continue the Trial. Similarly, if there is an identifiable barrier or number of barriers found then we will attempt to overcome the barrier(s) and reassess, deferring the commencement of the Definitive CLIP trial in that jurisdiction, while proceeding in the others. All decision will be made in consultation with the Bill &Melinda Gates Foundation (sponsor), after reviewing the advice of the CLIP trial Data Safety Monitoring Board (DSMB), and complying with relevant REB/ethical board guidance. The rationale for continuing the Pilot CLIP trial beyond the initial nine months is to beta-test the whole Trial process through at least two household surveillance cycles, as well as testing and validating any tools designed specifically for use in the Definitive CLIP trial but not related to the primary outcome.

All tools related to the Definitive CLIP trial primary outcome will be field-tested prior to the Pilot CLIP trial.

For CLIP Pilot and Definitive trials

Control clusters Women receive the current pattern of care

Intervention clusters Women and their communities receive the CLIP intervention

The primary implementers of the CLIP intervention will be cHCPs, the nature of whom will differ between countries. In Nigeria, these are Community Health Extension Workers (CHEWs) and Health Assistants (HAs); in Mozambique, these are Agente Polivalente Elementares (APEs); in Pakistan, these are Lady

Health Workers (LHWs); and in India, these are Accredited Social Health Advocates (ASHAs) and Auxiliary Nurse Midwives (ANMs). Unless context-specific, we will use the term cHCP to describe these cadres of health providers for simplicity. The training and experience of these groups are described in Table 2.

As the primary outcome changes between the Pilot and Definitive Trials, the women in the Pilot Trials will contribute to the sample size of the Definitive Trials. We plan that each pair of Pilot and Definitive CLIP Trials will span 48 months (36 months' recruitment) as shown in Figure 7 below. In India, the Pilot phase will be shorter due to the difference in surveillance process as described later in this protocol.

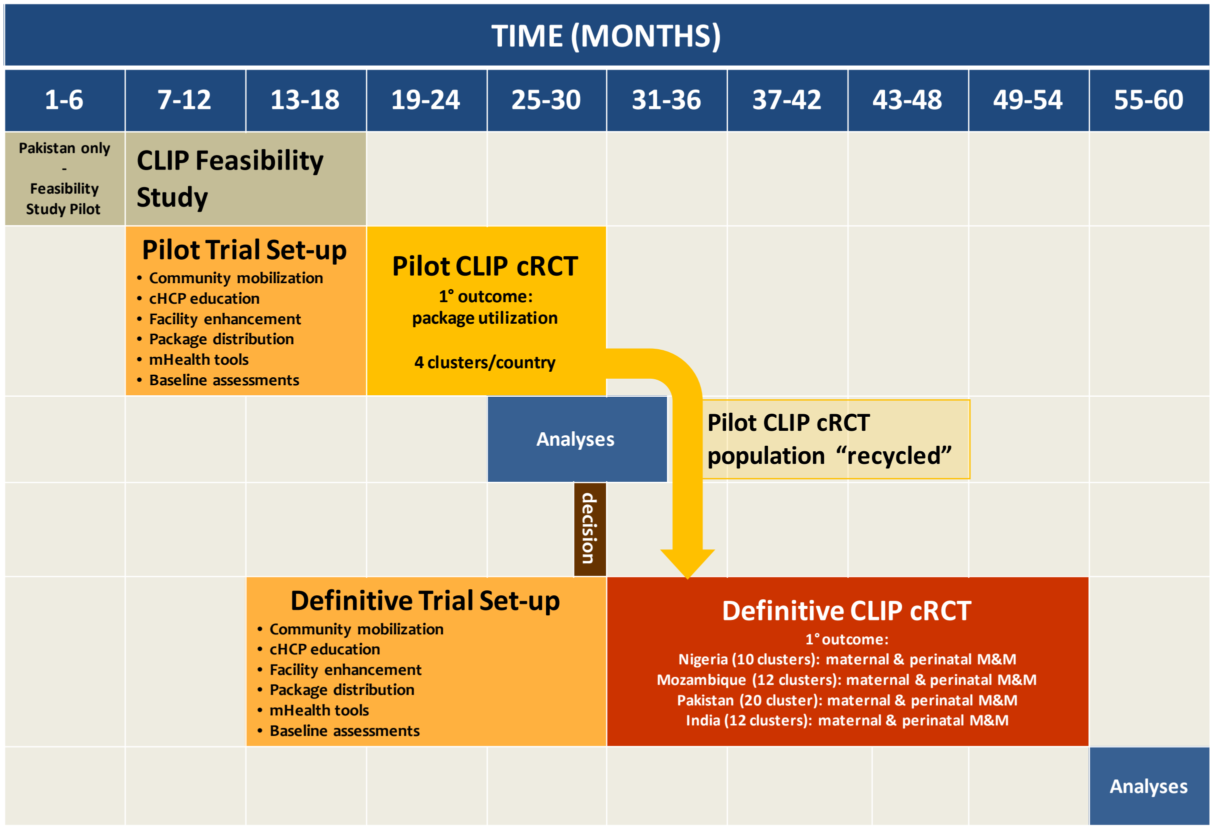

Figure 7: CLIP trial timeline

cHCP community health care provider, **cRCT** cluster randomised controlled trial, **mHealth** mobile health

2.2 Interventions

The interventions will be:

Control group: current practice (around antenatal care, referral to facility, and initiation of therapy).

Study group: The CLIP intervention consists of (i) *community engagement* including community leaders, the women of the communities themselves, and their mothers, husbands, and mothers-in-law, regarding pre-eclampsia, its origins, symptoms, signs, and potential consequences, pre-permissions for maternal transport, and fundraising activities around transport and treatment costs; (ii) *provision of HDP oriented antenatal care through CLIP visits and use of CLIP POM tool* (for risk stratification), and (iii) *use of the CLIP package for women with a CLIP 'trigger'* (i.e., oral antihypertensive therapy when indicated, intramuscular (i.m.) $MgSO_4$ when indicated; and appropriate referral to an CEmOC facility when indicated) (Figure 8). cHCPs will assess pregnant women with a target frequency of every 4 weeks at a

minimum, and according to protocol (Appendix C: CLIP cHCP Working Protocol). These visits can occur in the home or PHC as both are considered part of the community for the purpose of the CLIP Trial. They will be trained to enquire about women's symptoms (using country-specific pictograms), take women's BP (using sBP as it more closely reflects the risk for hypertensive stroke than does dBP[11;12]), and check urine for protein using dipstick on the first visit or on any subsequent visits for sBP ≥140 mmHg. This will inform the diagnosis and risk assessment of women with pre-eclampsia.

Both groups: CEmOC facility enhancement to promote evidence-based care of women with pre-eclampsia/eclampsia who are referred to CEmOC facilities, focussing on the WHO guidelines [34].

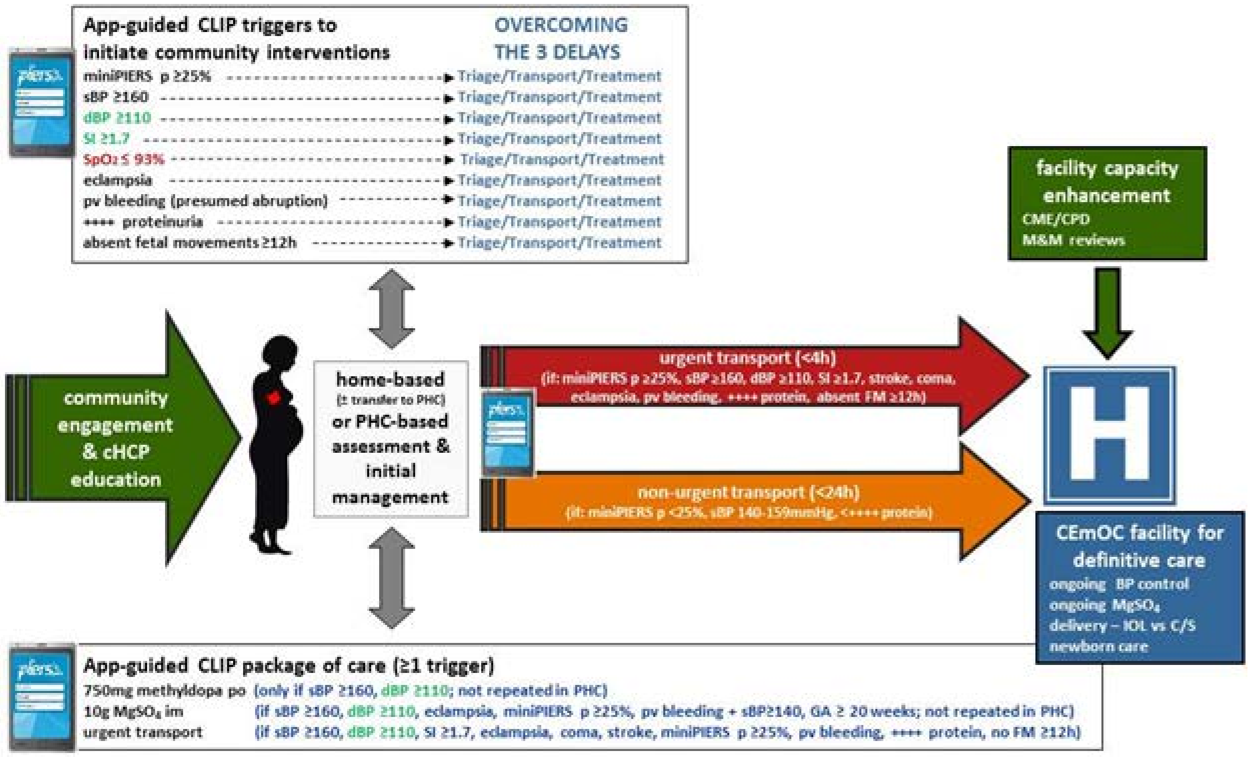

Figure 8: CLIP intervention diagram

CEmOC comprehensive emergency obstetric care, **cHCP** community health care provider, **CME/CPD** continuous medical education/professional development, **C/S** Caesarean section, **FM** fetal movements, **im** intramuscular, **IOL** induction of labour, **i.v.** intravenous, **M&M** morbidity and mortality, **p** probability, **PHC** primary health centre, **po** by mouth, **pv** vaginal, **sBP** systolic blood pressure

In green (for Nigeria), there are additional triggers for severe diastolic hypertension and shock. In red (Mozambique and Pakistan), there is an additional trigger for SpO_2 ≤93%.

Component 1: Community engagement

The primary objective of the community engagement activities in CLIP will be to create awareness and action around the prevention of maternal morbidity and mortality due to pre-eclampsia/eclampsia. Community engagement involves the collective action of individuals, families, religious leaders, policy makers, health care providers and community members toward the creation of meaningful and sustainable change. Studies indicate that successful health behaviour change occurs when interventions create positive social, individual, and environmental conditions. Furthermore, community-based interventions that

include women's and men's support groups, education, counselling, home visits, emergency transport initiatives and fundraising activities, have shown significant improvements in maternal and perinatal morbidity and mortality.

For each of the CLIP trials, a community engagement strategy will be developed with the participation of local stakeholders and community members that aims to be culturally and contextually appropriate, meaningful, sustainable, and effective in improving maternal health in CLIP intervention communities.

The first step is to determine what past and current activities related to community engagement around maternal health are occurring in control and intervention clusters. A specific tool has been developed for the purposes of identifying these community engagement activities so that the CLIP trial strategy can build upon them (Appendix C).

The second step follows the community mapping exercise. A community-specific engagement strategy will be developed, with direct input from community members, for the purpose of creating awareness and action around pre-eclampsia/eclampsia and the prevention of the associated maternal and perinatal morbidity and mortality, including but not limited to:

The CLIP community engagement activities must include (but are not limited to) the following nine topics:

1. Warning symptoms and signs of pregnancy complications, particularly pre-eclampsia & eclampsia
    - Relate the association of danger symptoms/signs with the occurrence of pre-eclampsia/eclampsia, using the warning symptoms of pre-eclampsia and eclampsia pictograms.
    - Identify the need for referral when danger symptoms/signs associated with pre-eclampsia/eclampsia occur.
    - Considering discussing postpartum haemorrhage (PPH), a 'visible' cause of maternal death, as a segway into discussion of pre-eclampsia/eclampsia as a 'silent' killer of pregnant/ postpartum women
2. Permission for women to seek care
    - Recognise the need for decision-making power and/or prior permissions in the event of obstetric emergencies.
    - Discuss how women can obtain prior permission to seek that care
3. Identification of skilled birth attendant
4. Identification of facility for delivery
5. Transport and treatment funds
    - Recognise the need to develop plans for financial resources when required in emergency conditions associated with pre-eclampsia/eclampsia. Funds may be personal or from the community. The community engager should facilitate the individual communities to form a plan for transport and treatment funds.
    - Encourage the identification of existing community resources (if applicable) and the development of community funds for seeking emergency care. The community should be told that CLIP will supplement any existing funds, but fund-raising activities must build on those funds to make this sustainable
    - Identify available and appropriate modes of transport, the associated costs, and the means by which these modes can be accessed in emergencies.
6. Feedback mechanisms about adverse outcomes and 'great saves'
    - Country-specific CLIP Team could request that families of sufferers share their experiences with the community

7. CLIP protocol (brief)

    - The description should be brief, and focus on who is eligible and the CLIP intervention (of which community engagement is an important component)

8. CLIP visits and the CLIP triggers for treatment and transport (brief)

    - Review the frequency of the visits, assessments to be done as part of each visit, and the 'triggers' that will prompt the cHCP to recommend treatment and transport to facility.

9. Discussion about, and trouble-shooting designed to address, the barriers identified in the country-specific CLIP Feasibility Study.

Community engagement strategies used in the CLIP trial intervention clusters may take many forms and will be unique to the culture and context of each community as well as the community's resources and constraints. The CLIP team aims to utilise knowledge gained from an update to the Cochrane systematic review of the literature on community mobilisation in LMICs (being led by Susan Munabi-Babigumira), in co-ordination with the results of community mapping, and direct community input, to develop a community engagement strategy that is both evidence-based and tailored to the unique needs of CLIP intervention clusters.

It is recognised that sustainable community engagement activities more frequently involve an element of fund raising activity, than activities that are not sustained [35-38]. Therefore, the focus on transport and treatment fundraising activities is a singularly important element of the community engagement activity, and one that the CLIP Trial will pump prime funds so that every intervention cluster community has access to funds as the Trial commences to support the transport and care of women from the initiation of trial activity in that cluster. Maintenance and strengthening of that funding pool will be the responsibility of the intervention cluster communities.

Component 2a: cHCP training in home-based maternity surveillance

We will develop an intervention package that involves the relevant main providers of primary maternal care in the community: LHWs (Pakistan), CHEWs and HAs (Nigeria), ANMs and ASHAs (India), and APEss (Mozambique). These health care providers are termed cHCPs for this protocol. A description of the current scopes of practice in maternity care for these cHCPs is provided in Appendix A. In addition, in all countries, PHC-based health workers will be included in formal CPD activities provided in parallel to the CEmOC facilities to which women will be referred (see immediately below).

The cHCPs will be encouraged to identify pregnant women in their area, and will have pregnant women referred to them when identified by the surveillance team during regular surveillance cycles. The regular CLIP visits will occur approximately: 4-weekly during pregnancy until 28 weeks, fortnightly from 28-35 weeks and weekly thereafter, within 24 hours of birth, and on approximately days 3, 7, and 14 after delivery. 4-weekly visits over the course of a pregnancy in women enrolled in the study will be considered a minimum standard for exposure to this aspect of the intervention; anything less than 4-weekly will be recorded as non-compliance with the intervention. These visits are not meant to supplement regular ANC clinical visits, but will be performed in coordination with those existing programs to achieve the frequency as previously described. At each of those visits, the CLIP assessment will place women in one of three care trajectories: (i) usual antenatal care, (ii) non-urgent referral (within 24hr), or (iii) urgent referral (within 4 hr) to a CEmOC facility. Visits may occur in the woman's home or at the nearest PHC.

The CLIP intervention package (including the oral antihypertensive, $MgSO_4$ and all relevant safety and

disposal devices) will be provided to the cHCPs in addition to incentives for the increased workload. Relevant incentives (e.g., monetary, gifts, or plaques) will be determined in conjunction with national site investigators.

For cHCP training in Pakistan, the enhanced education module will be developed as an adjunct to the regular LHW training programme; this module will be developed in collaboration with either the Sindh Ministry of Health or national Directorate of Health (depending on which entity has responsibility for the LHW programme after the current devolution to provinces is complete). In Nigeria, India and Mozambique, we will undertake parallel processes in collaboration with our local partners and relevant Ministries of Health.

This enhanced education module will include information about:

1. Understanding pre-eclampsia and eclampsia
2. Overview of the CLIP Trial
3. Basics of effective communication
4. Consent taking procedures
5. CLIP protocols
    a. CLIP materials
    b. CLIP visit schedule and protocols
        i. Counselling of women about:
            1. Warning signs of pre-eclampsia/eclampsia (involving use of pictograms)
            2. Advanced permission to seek care
            3. Transport plan
        ii. Proteinuria measurement
        iii. Blood pressure measurement using Microlife AS1-2 device in Mozambique, Pakistan and India and using the Microlife CRADLE VSA in Nigeria
        iv. SpO_2 measurement using the LGT Medical audio oximeter connected to the mobile phone (Mozambique and Pakistan only)
        v. Estimating gestational age
        vi. Assessment of warning signs of pre-eclampsia/eclampsia utilizing pictograms
    c. CLIP treatment
        i. Triggers for treatment
        ii. Administration of methyldopa
        iii. Administration of MgSO4
        iv. Appropriate referrals, utilizing the referral form
        v. Recognizing adverse events
        vi. Triggers for treatment and appropriate medication administration
    d. Use of PIERS on the Move application
6. Contact Information

We anticipate that CLIP training will take an initial 2-3 days with an extra day for refresher training every six months or as needed.

Training of cHCPs will be evaluated at the initial training session, at each subsequent refresher session and throughout the CLIP Trial. Remedial action will be offered in the case of substandard performance with the evaluation. This evaluation will include pre and post assessment of both competence and self-efficacy on each training objective by means of demonstration of CLIP skills and completion of a survey.

2b: Diagnosis & triage – miniPIERS & CLIP POM

The final miniPIERS cohort used for analysis included 2081 women. The worst clinical values within the first 24hr after admission were used to develop the model. Variables included in the final miniPIERS model are parity (multiparity vs. nulliparity), gestational age on admission (or delivery if assessing a postpartum woman), the symptoms of chest pain and/or dyspnoea, headache and/or visual disturbances, vaginal bleeding with abdominal pain, and right upper quadrant pain; sBP; and dipstick proteinuria. The AUC ROC for this model was 0.6876868 (95% CI 0.37353 – 0.18011) which demonstrates a great ability to discriminate between women with and without adverse maternal outcomes. The stratification capacity of the model is good. Using a predicted probability cut-off of 25% resulted in a positive likelihood ratio of 5.09 [4.12, 6.29] and classified women with 85.0% accuracy, suggesting moderate utility of the model as a rule-in test for adverse maternal outcomes. The CLIP version of the PIERS on the Move tool (CLIP POM) integrates the miniPIERS predictive score and a clinical data collection system into a single application. cHCPs will assess women according to the visit protocol (Appendix D), entering clinical data into the CLIP POM mobile application. The application will provide recommendations for care according to Figure 9 below, as per this protocol. Triggers identified that will indicate treatment and/or transport (urgently, defined as within 4hrs) to a CEmOC facility are as follows:

1. Unconsciousness ($MgSO_4$ if sBP ≥160 mmHg and gestational age is equal to or greater than 20 weeks (GA≥20 weeks) [to be reasonably sure that the unconsciousness is associated with severe pre-eclampsia and not due to obstetric sepsis], urgent transport)
2. Signs of recent stroke or seizure (methyldopa if sBP ≥160 mmHg [to ensure BP is not lowered too much], $MgSO_4$ (if GA≥20 weeks) urgent transport)
3. Significant vaginal bleeding ($MgSO_4$ if sBP ≥140 mmHg and GA≥20 weeks [presumed abruption associated with severe pre-eclampsia] , urgent transport).
4. No fetal movements felt in the previous 12 hrs (urgent transport [a threshold for identifying at risk fetuses that are alive at the time of screening] [39])
5. sBP ≥160 mmHg (or dBP ≥ 110 mmHg in Nigeria only) (methyldopa, $MgSO_4$ (if GA≥20 weeks) urgent transport [consistent with severe pre-eclampsia])
6. Heavy proteinuria (≥4+ by dipstick – predictive of stillbirth in miniPIERS cohort, urgent transport)
7. miniPIERS predicted probability ≥25% ($MgSO_4$ (if GA≥20 weeks)urgent transport)
8. Shock index ≥ 1.7 in Nigeria only (the Shock index is a ratio of pulse/sBP; high shock index is an indication of poor prognosis in women with postpartum haemorrhage)

Non-urgent transport (by non-ambulance services), meaning assessment at a CEmOC facility within 24 hours, will be advised for all women with non-severe hypertension (sBP 140-159 mmHg) who do not meet criteria for one of the above 7/8 triggers.

In Mozambique and Pakistan additional CLIP triggers based on use of the audio oximeter will also be included in the POM decision aid. As with the original miniPIERS model, the enhanced model including SpO_2 uses a risk threshold of ≥25% predicted probability to identify high-risk cases. Recommendations based on the updated miniPIERS model will include treatment with $MgSO_4$ and urgent referral. An additional independent trigger of SpO_2≤93% will also be used in Mozambique and Pakistan to indicate urgent referral.

In Nigeria where the updated Microlife CRADLE VSA blood pressure device is being used additional triggers will be included for severe diastolic blood pressure or severe shock index to coincide with the traffic light warning signs included in this device (see Appendix E).

There will be ***three models of referral*** within the Pilot and Definitive CLIP Trials, depending on the country. In Nigeria and Pakistan, women, their families and communities will be responsible for identifying and paying for transport to the CEmOC facility unless the woman is found in critical condition. In these cases an emergency trial transport fund will be made available to ensure no undue harm comes to women found in critical condition during a CLIP visit. The related fundraising activity will be a focus of the community engagement, particularly in these two countries to ensure in non-urgent situations women can get to care. In Mozambique, women, their families and communities will be responsible for identifying and paying for transport to the nearest PHC, where i.m. $MgSO_4$ will be administered (if relevant) and where women will be transferred by ambulance (free service provided 24/7) to a CEmOC facility. In Belgaum and Bagalkot Districts, Karnataka, India, there is a functioning ambulance service available free of charge 24/7. Women will be taken from their home or local subcentre (if ANM not available to go to their home) directly to the CEmOC facility.

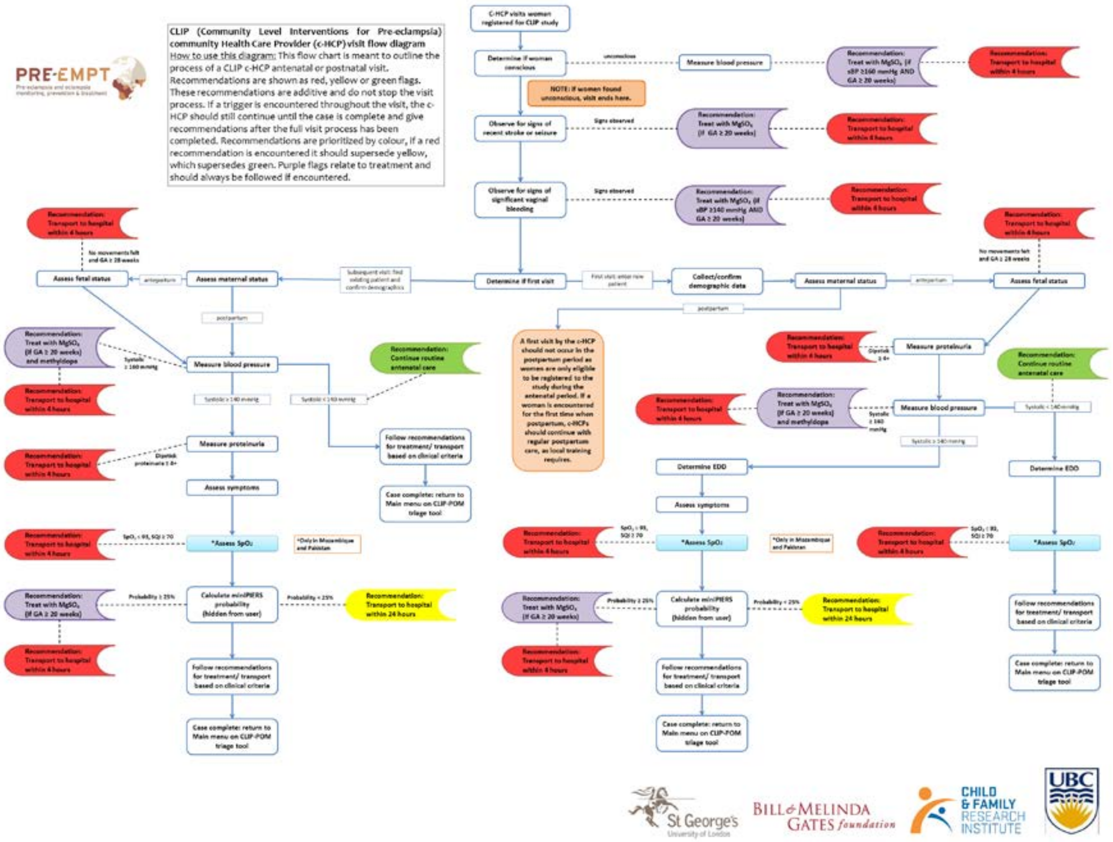

Figure 9 CLIP POM decision model for use in the mobile application for the CLIP trial

2c: Lowering severe hypertension – oral methyldopa

In the absence of a clearly preferred oral agent, we have opted for ***methyldopa 750mg***. Our rationale is that oral methyldopa has been well-studied in pregnancy hypertension RCTs, and although possibly less effective than some agents[5], is less likely to cause hypotension[5], has no common medical contraindications, and can be administered orally[5]. Also, methyldopa is readily available on nearly all LMIC essential medicines lists and it is cheap and acceptable for use by most practitioners [Lalani *et al. JOGC*]. As shown in the decision tree (Figure 9), methyldopa will be administered to women when:

- sBP is ≥160 mmHg, or dBP ≥110 mmHg (in Nigeria only)

Women will be given methyldopa if identified as having severe hypertension as defined above regardless of current medication use. This means that any woman encountered in the community who is already taking an antihypertensive should still be given a full dose of methyldopa if severe hypertension is measured.

Women will not receive further methyldopa until they reach a CEmOC facility. As part of facility enhancement, personnel at CEmOC facilities will be educated about how to deal with those women enrolled in CLIP who are transported to facilities. As part of these enhancement activities described below, we will encourage facility staff to provide further antihypertensive therapy if at least 40 minutes has passed and sBP has not started to decrease; if so, clinicians will be advised to wait and monitor BP until the peak effect of methyldopa occurs (between 3 and 6 hr). Otherwise, additional antihypertensive therapy (such as oral nifedipine or i.v. hydralazine) may be administered.

2d: Preventing & treating eclampsia – intramuscular $MgSO_4$

$MgSO_4$ is the treatment of choice to prevent and manage eclampsia in high, middle, and low income countries [3;40-43]. In the International Eclampsia and Magpie Trials, the loading dose was either: (i) 4g i.v. given over 10-15 minutes plus 10g i.m. (followed by i.m. maintenance therapy of 5g i.m. every 4 hr), or (ii) 4g i.v. (followed by i.v. maintenance therapy at the rate of 1g/hr).

We have elected to use only ***10g $MgSO_4$ via the i.m. route***. Our rationale for this is as follows. First, the choice of a 10g loading dose is supported by the WHO (Integrated Management of Pregnancy and Childbirth, 2009), where, in the absence of i.v. access, the expert committee advised loading with only 10g i.m. of $MgSO_4$[44]. Second, the use of i.v. medication by cHCPs is not feasible and the i.m.-only regimen results in therapeutic serum Mg^{2+} concentrations by 45 minutes after i.m. administration [33]. Third, although there are limited RCT data for this regimen, there is substantial regional experience with i.m. loading dose administration in the community (Hall & Theron, personal communications) as well as with 10g total loading dose regimens[45-51]. Professors Hall and Theron have over 10 years' experience with community i.m. $MgSO_4$ loading in the Cape Flats, Cape Town, South Africa, without apparent maternal harm. In Shahjadpur, Bangladesh, the ICDDR,B community-level (community skilled birth attendants) intervention for women with eclampsia includes a total 10g loading dose (4g i.v. and 6g i.m.) based upon the Dhaka protocol [45;46;52;53]. Currently, in our study areas in India and Pakistan, ANMs and LHWs areadministering i.m. dexamethasone within the context of an RCT (clinicaltrials.gov/ct2/showNCT01084096 [accessed 28 Jan 2013]) which shows capacity for skill training in this area.

Based on the approach used in the successful Eclampsia and Magpie Trials, we will develop '***pre-eclampsia boxes***' for use by cHCPs. These boxes will include single doses of oral methyldopa (750mg) and i.m. $MgSO_4$ (10g in two 5g vials of 50% solution), along with all necessary supplies and disposal measures.

Women will not receive further $MgSO_4$ until they reach a CEmOC facility. There, they will receive a further dose according to local protocols. As part of facility enhancement, personnel at CEmOC facilities will be educated about how to deal with those women enrolled in CLIP who are transported to facilities. Through these enhancement activities, described below, we will encourage standardised dosing regimens in line with international guidelines such as 2g i.v. $MgSO_4$ over 10-15 minutes for recurrent eclampsia (that occurred en route or at facility). Through CPD activities, we will suggest optimal treatment protocols for CLIP women referred to facilities. Following administration of 10g i.m of $MgSO_4$ in the community:

- Women will not receive further $MgSO_4$ therapy until they have reached a CEmOC facility
- At the CEmOC facility, these women will receive more $MgSO_4$ ONLY after at least 4 hr have passed since their initial 10g i.m $MgSO_4$ loading dose given in the community.

- If women arrive at the CEmOC facility 4-6hr after receiving a 10g i.m $MgSO_4$ loading dose in the community, these women should receive $MgSO_4$ maintenance therapy, consisting of either: (i) 5g i.m. every 4hr, or (ii) 1g/hr i.v.
- If women arrive at the CEmOC facility >6hr after receiving a 10g i.m $MgSO_4$ loading dose in the community, these women should receive another loading dose of $MgSO_4$ following by maintenance therapy, as follows: (i) 10g i.m. plus 4 g i.v. loading dose, followed by 5g i.m. every 4 hr as maintenance therapy, or (ii) 4g i.v. loading dose, followed by 1g/hr i.v. maintenance therapy.

Component 3: Facility enhancement

We will undertake enhancement activities at facilities able to provide EmOC, to ensure a minimum standard of care will be provided to all inpatient women in both intervention and control clusters. In addition, although health workers based in PHCs are care providers at the community level, their training and experience mandates that they receive their CLIP-related training within the context of facility-based continuous professional development.

In India and Pakistan, EmOC facilities may be shared by intervention and control clusters, whereas they are distinct in Nigeria and Mozambique.

Facility enhancement activities will be undertaken in collaboration with local and international partners (e.g., MCHIP, RCOG, SOGC). The objectives of facility enhancement are to:

1) Provide facilities with ***general HDP-related educational resources***, including WHO guidelines on pre-eclampsia and eclampsia [34], textbooks, and posters.
2) Provide health providers with ***special clinical training in basic and intermediate care of women with HDP***
    a. CLIP protocol (including nature of cHCP CLIP visits with respect to counselling provided, tasks performed, and medications administered) and how to appropriately manage these women once they reach a facility
    b. Warning signs of pre-eclampsia/eclampsia
    c. BP measurement
    d. Proteinuria measurement
    e. miniPIERS assessment on admission
    f. Management of severe hypertension
    g. Management of severe pre-eclampsia and eclampsia, including $MgSO_4$ and timing of delivery
3) Enhance health provider ***respectful communication skills*** with women, their families, and other maternity care providers
4) Inform ***adverse outcome identification*** related to the CLIP intervention. Through this mechanism, we will use facility-level (and, therefore, more accurate) information for all women in the CLIP Trials to assess the accuracy of data collected through the household surveillance approach. This comparative analysis will be important as a validation exercise should the planned difference in adverse outcome rates be achieved.

2.3 Risks to the Safety of Participants

The main risk of the CLIP intervention is that it may lull cHCPs, families, and the women themselves into a false sense of security, thereby delaying the initiation of transport for definitive and life-saving treatment. It is for this reason that the CLIP package of care will include education about appropriate transport to facility,

as well as instructions about how quickly that transport should occur.

For methyldopa safety, we will identify the percentage of women with an 'out of target' BP on admission to facility (i.e., sBP ≥160mmHg indicating lack of effect, or sBP <110mmHg indicating excessive effect).

$MgSO_4$ toxicity is not a major concern. Mg^{2+} is renally cleared but even if a woman were anuric, one 10g i.m. loading dose of $MgSO_4$ would not result in serum Mg^{2+} concentrations sufficient to cause neuromuscular blockade[54]. However, given that repeat doses would have the capacity to do so, no repeat doses of i.m. $MgSO_4$ will be given in the community by cHCPs (or at a PHC with only BEmOC capabilities), even if the indication for therapy persists. Practically, we will assess $MgSO_4$ injection site haematomas, infections and pain using standardised assessment tools in women who received the particular intervention. cHCPs will be trained in proper methods of administration of i.m. injections and all women who receive an injection will be referred to a local facility for follow-up in order to minimize any risks related to these side effects.

2.4 METHOD OF RANDOMISATION

Clusters

The unit of randomisation will be defined by criteria relevant to each setting.

In Nigeria, the unit of randomisation will be Local Government Areas (LGAs). Each has a Department of Health headed by a Medical Officer of Health. All villages and PHCs within the LGA will be included in that cluster.

For the pilot phase, we will include four LGAs.

For the definitive phase, we will include 10 (5 intervention and 5 control) clusters in the trial with a public and private sector referral facility in the Ogun area. The four pilot trial clusters will remain in their assigned groups.

In Mozambique, the unit of randomisation is the Administrative Post. All villages and PHCs within the unit of randomisation will be included in that cluster.

There will be no Pilot phase in Mozambique. For definitive phase, we will include 12 (6 intervention and 6 control) clusters in the trial with a public and private sector referral facility in the provinces of Maputo (3 de Fevereiro, Maluana/Maciana, Ilha Josina/Calanga, Magude) and Gaza (Xilembene, Chicumbane, Nhancutse, Chibuto, Macia, Chissano, Mazivila, Messano)..

In Pakistan, the unit of randomisation will be defined by their union council. The villages and PHCs within that union council will be included in the cluster. We will recruit in the catchment area of adjacent union councils of Hyderabad and Matiari, Sindh, Pakistan.

For the pilot phase, we will include four union councils.

For the definitive phase, we will include 20 (10 intervention and 10 control) clusters in the trial with a public and private sector referral facility in the area adjacent to Hyderabad and Matiari (the latter to give patients choice in terms of referral options beyond the rural health centres (RHCs)). The four pilot trial clusters will remain in their assigned groups.

In India, the unit of randomisation will be the PHC. All villages within the PHC catchment area will be included in that cluster.

For the pilot phase, we will include four PHCs.

For the definitive phase, we will include 12 (6 study and 6 control) clusters in the trial with a public and private sector referral facility in the area adjacent to Belgaum and Bagalkot. The four pilot trial clusters will remain in their assigned groups.

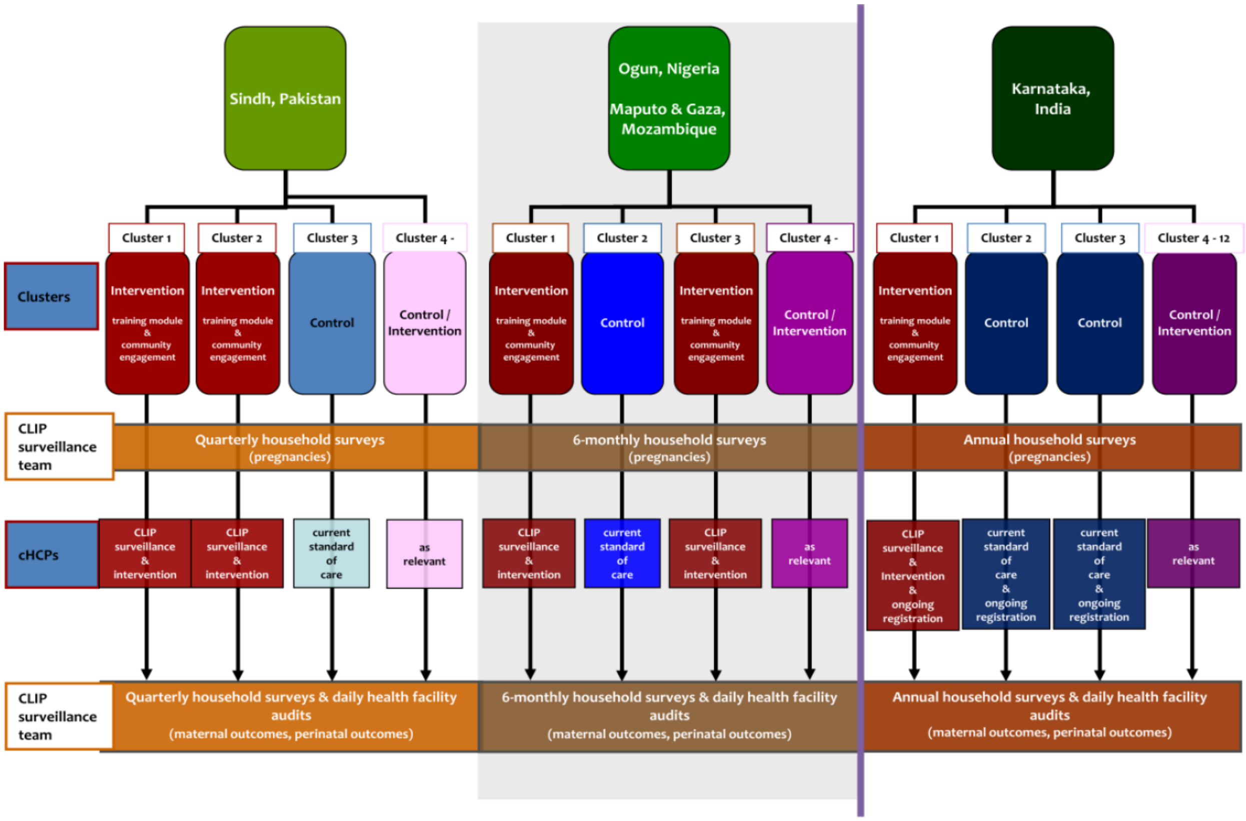

Figure 10 Trial organisation by country and clusters cHCP community health care provider

To ensure reasonable balance between the two groups, we will use restricted, stratified randomisation to allocate clusters to the intervention and control groups [12].We will be using only one continuous stratification variable for the randomization of clusters in each country. For all countries this will be Population size. The randomization algorithm ensures adequate balance on the stratification variable over the Intervention arm and the Control arm. Allocations that fail any of the balance criteria will be excluded, and only allocations that meet all criteria are eligible for consideration. The final allocation scheme is a random selection from the list of eligible allocations.

The current algorithm is implemented using R Programming and can consider the following balance criteria:

1. Means ratios: the mean value over intervention clusters and the mean value over control clusters have a ratio no less than 0.9 and no greater than 1.1 (10%).

2. Mean relative to overall mean: the overall mean is the mean over all clusters; mean value in each arm is within 10% of overall mean.

3. Absolute difference in sums: the absolute difference between sums in the two arms is within 10,000.

4. Wilcoxin rank sum: Allocations where the Wilcoxin rank sum test have p-value <0.01 will be excluded.

5. Kolmogorov-smirnov test: Allocations where the Kolmogorov-smirnov test have p-value <0.01 will be excluded.

In the pilot phase of the trial we randomize 4 clusters per country, except Mozambique where no Pilot phase is occurring. When we approach the definitive phase of the trial, these 4 clusters will be included in the randomization algorithm together with the new clusters, and their previous allocations will serve as an additional requirement for the algorithm. The set of eligible allocations will be those that meet the list of balance criteria and the pilot-allocation requirement.

We may or may not use all of the balance criteria listed, and the cut-points listed may be modified. These are common criteria and cut-points used in the literature, but adjustments may be needed if the resulting number of eligible allocations is deemed inadequate.

At the stage of analysis other cluster level factorsidentified as having an impact on the outcome will be adjusted for, including those used for stratification, such as (i) cHCP density by cluster, (ii) population density, (iii)distance to referral facilities (public or private), (vvi) home birth numbers, (v) skilled birth attendant numbers, and (vi) mortality data. Once the decision has been made to proceed to the Definitive Trial phase the population numbers will be re-confirmed for all remaining non-Pilot clusters and the stratification and randomization process will be performed as described above. Clusters participating in the Pilot Trial phase will not be re-randomised for the Definitive Trial phase but will retain their Pilot Trial phase designation of intervention or control.

2.5 Protecting against bias

We will *not* undertake a hospital level implementation trial, but rather, focus on community-level (i.e., cHCP and PHC) interventions. It is at those levels that the burden of disease-related risk lies. However, informed by the results of the Feasibility Study in each country, we will have determined that, at first level inpatient facilities (BEmOC) and the CEmOC facilities, both: (i) effective antihypertensive therapy is used in >80% of women with severe hypertension (sBP ≥160mmHg and/or dBP ≥110mmHg), and (ii) $MgSO_4$ is on formulary and used in >90% of women with eclampsia. Ensuring a standard and consistent level of care across both intervention and control clusters will reduce any bias that may have existed due to variability in facility based quality of care at study baseline.

Specifying and selecting study clusters

No cluster will have participated (or be currently participating) in either antenatal intervention trials or community mobilisation.

Execution of the intervention

Contamination bias

Communities in control clusters will not be engaged in any community engagement activities.

cHCPs in control clusters will receive no additional training about pre-eclampsia/eclampsia diagnosis or management, or the CLIP protocol. At present, cHCPs in these jurisdictions do not measure both BP and proteinuria and in Pakistan, they measure neither. cHCPs in control clusters will not be taught how to measure BP or proteinuria because their measurements are components of the miniPIERS risk stratification tool, an important element of the CLIP intervention. cHCPs in control clusters will have access to neither the mHealth tools or the CLIP intervention package.

There is no risk of contamination at the CEmOC facility level as both intervention and control clusters will be engaged in capacity enhancement activities at CEmOC facilities in order to ensure that women who are transported to a CEmOC facility with pre-eclampsia/eclampsia will receive standardised care. This is an important element as it is anticipated that the CLIP intervention will result in more women sent to facility, because of diagnosis and treatment of pre-eclampsia/eclampsia that either: would not have been otherwise made (and could have resulted in death or morbidity that went unrecognised because they did not go to facility), or would have been made at a later stage of their disease (at which point these women would have been transferred to facility). PHC workers in the intervention clusters (not those in control clusters) will be educated within the BEmOC and CEmOC capacity enhancement.

Compliance bias

We will assess cHCP compliance with administration of the intervention using the mHealth technology.

Measurement bias

We will reduce the risk of measurement bias by using measurement tools tested for validity and reliability during the CLIP Feasibility Studies or by others in unrelated studies (Appendix D).

BP measurement will be standardised through use of a standard protocol and an automated measurement device (Microlife BP 3AS-2).

2.6 Inclusion / exclusion criteria

Participants will be ***all consenting pregnant women aged 15-49 (except in Mozambique where reproductive age begins at 12)*** identified by the cHCPs assigned to their community. No such pregnant women will be excluded from the analysis which will be by intention-to-treat.

In Nigeria and Mozambique, all women in both control and intervention clusters will give written consent for surveillance (head of household/women of reproductive age) and verbal consent for the secondary visits for the entire CLIP trial. In India, consent will be written. In Pakistan, all women in both control and intervention clusters will give: verbal consent to participate in the quarterly household surveillance, and written consent (personal or proxy [if obtunded]) for administration of the intervention package, if indicated.

2.7 Proposed duration of treatment

The ***community engagement*** aspect of the CLIP intervention will be applied from the time of randomisation of the clusters until completion of recruitment to the Trial.

The ***provision of HDP-oriented antenatal care through CLIP visits and use of the CLIP POM tool***, as well as use of the ***CLIP package for women with a 'trigger'***, will be applied from the identification of each woman's pregnancy (and her consent to participate in CLIP), through transport to an CEmOC facility (as indicated), and until six weeks postpartum. From the time of diagnosis of a CLIP trigger consistent with pre-eclampsia, women may remain pregnant for an average 10-14 days depending on the gestational age at diagnosis [1]. After diagnosis all women are referred to care at a local facility where they will receive the standard of care in place at that facility. The standard that we will promulgate through our facility enhancement rounds will specify that induction of labour (or semi-elective Caesarean if obstetrically indicated) will be offered routinely to women at or beyond 37^{+0} weeks' gestation [55].

2.8 Frequency and duration of follow-up

Clinically, women will be followed up by cHCPs up to 14 days postpartum. In addition, women will be

followed until 42 days after their delivery by CLIP research staff. In all clusters, this research follow-up will be by means of regular household survey over the 18 months of the trial (i.e., baseline and 9 follow-up surveys) and daily facility audit (as outlined in Figure 10).

2.9 OUTCOMES

- ***Pilot CLIP Trials***

The ***primary outcome*** for the Pilot CLIP Trials will be common for all participating countries, and will be appropriate use of the CLIP package of care (methyldopa and/or $MgSO_4$ and urgent/non-urgent transport to CEmOC facility) in women with the appropriate CLIP trigger. To justify continuing on with the Definitive CLIP Trial, by 8 months into the 12 month Pilot (with the exception of India were assessment will occur at 6 months), at least 50% of eligible women (i.e., with a trigger) should have received the package of care.

The Pilot CLIP Trials will recruit for 12 months, primarily to field test: application of the CLIP intervention, data collection, and research infrastructure through four quarterly surveillance cycles (in Pakistan).

The ***secondary outcomes*** for the Pilot CLIP Trials will be the adverse maternal and perinatal outcomes described below for the Definitive CLIP Trials.

The ***other outcomes*** will be the secondary and other outcomes described below for the Definitive CLIP Trials.

As the primary outcome will change between the Pilot and Definitive CLIP Trials, women recruited to the Pilot Trial will be included in the total sample analysed for the Definitive Trial.

- ***Definitive CLIP Trials***

The ***primary outcome*** will be a combined maternal and/or perinatal outcome (either maternal, fetal or neonatal death, or severe morbidity for the mother or baby). We have chosen a combined maternal and/or perinatal outcome as powering a trial for maternal death alone would require a prohibitively large sample size.

Maternal death (defined as the number of deaths during pregnancy or within 42 days of pregnancy (or last contact day if contact not maintained to 42 days) / 1,000 identified pregnancies), termed Maternal Death Rate. We have not chosen the classical WHO maternal mortality ratio (MMR) definition of events per 100,000 live births because we hope that the CLIP intervention will reduce stillbirth in women with pre-eclampsia. If so, the MMR per 100,000 live births would fall artificially as the proportion of live born infants increased, without any absolute change in the maternal adverse event rates (per pregnancy). We will be basing the number of pregnancies on the household surveys; although the timing of public declaration of pregnancy varies between cultures (first trimester in India and up to 20-24 weeks in Pakistan, Nigeria, and Mozambique), the manner of identifying pregnancies will be similar between control and intervention clusters in each country.

Maternal morbidity (defined as the number of women with one or more life-threatening complications of pregnancy during pregnancy or within 42 days of pregnancy or last contact day if contact not maintained to 42 days) / 1,000 identified pregnancies). These are the serious end-organ complications of pre-eclampsia, other major causes of maternal mortality, or life-saving interventions related to one of the aforementioned:

Serious end-organ complications of pre-eclampsia:

- *Eclampsia:* occurrence of generalised convulsions during pregnancy, labour or within 42 days of

delivery in the absence of epilepsy or another condition predisposing to convulsions

- *Stroke:* hemiparesis and/or blindness developed during pregnancy or in the 42 days postpartum lasting greater than 48 hours
- *Coma:* prolonged unconsciousness ≥12 hours
- *Antepartum haemorrhage:* vaginal bleeding ≥ 15 mL with or without pain before the onset of labour
- *Disseminated intravascular coagulation* (DIC): abnormal bleeding from mucosa(mouth and/or ears)

Other major causes of maternal mortality:

- *Obstetric sepsis:* In the community, defined as fever and one of: abdominal/uterine tenderness, foul smelling vaginal discharge/lochia, productive cough and shortness of breath, dysuria or flank pain, headache and neck stiffness. In the facility, defined as presence of fever (>38°C), a confirmed or suspected infection (e.g., chorioamnionitis, septic abortion, endometritis, pneumonia) and at least one of the following: heart rate >90/min, respiratory rate >20/min, leukopoenia (total leukocyte count [TLC] <4 x 10^9/L) or leukocytosis (TLC >12 x 10^9/L)
- *Vesicovaginal or rectovaginal fistula:* continuous loss of urine and/or faeces after delivery

Life-saving interventions:

- *Cardiopulmonary resuscitation:* a set of emergency procedures including chest compressions and lung ventilation applied in cardiac arrest victims
- *Dialysis:* haemodialysis and/or peritoneal dialysis
- *Mechanical ventilation* (other than for Caesarean section): intubation and ventilation not related to anaesthesia
- *Blood transfusion:* ≥1 unit
- *Interventions for major postpartum haemorrhage:* brace sutures, external and internal uterine compression, anti-shock garment use, internal iliac artery ligation and/or hysterectomy with or without transfusion

Perinatal & late neonatal death (defined as stillbirth [≥20^{+0} and/or ≥500g], early neonatal mortality [d 0-7 of postnatal life] and late neonatal mortality [d 8-28 of postnatal life] / 1,000 identified pregnancies), or

Neonatal morbidity (defined as occurence of a primary neonatal morbidity[30] during d 0-28 of postnatal life / 1,000 identified pregnancies). The following are the primary neonatal morbidities:

- ***Feeding difficulty***
  - *Including inability to suckle normally or latch on to the mother's breast to feed even if the mother's milk is not let down*
- ***Breathing difficulty***
  - *Including grunting and in-drawing of the abdomen under the ribs*
- ***Seizure***
  - *Occurrence of any seizure event (fits)*
- ***Lethargy***
  - *Baby not appearing normally wakeful after activities such as feeding or sleeping*
- ***Coma***
  - *Not medically induced period of unconsciousness of any length*
- ***Hypothermia***

- *Cold to touch*

➤ ***Umbilical cord infection***

- *Characterized by discharge from and redness around the umbilical stump*

➤ ***Skin infection***

- *Any appearance of abnormally red, black, swollen and blistered skin with pus*

➤ ***Bleeding***

- *From anywhere*

➤ ***Jaundice***

- *Yellow skin and eyes*

➤ ***Central nervous system related morbidity***

- *Abnormal amount of vomiting as defined by the parents or caregiver with bulging or sunken fontanelle*

Secondary outcomes for the Definitive CLIP Trials have been defined to measure the impact of the CLIP intervention on the delays around triage and transport. The effect of the CLIP intervention on treatment delays will be determined by the primary outcome and does not need further evaluation as a secondary outcome.

Birth preparedness and complication readiness as measured by any three of the following: (1) arranged for transport; (2) obtained prior permission for transport should emergency arise; (3) saved money for obstetric care; (4) identified skilled birth attendant; (5) identified facility for delivery. This will evaluate the success of community engagement.

Number of women presenting for care at a CEmOC facility and *number of facility births*: This number should increase if triage (using the miniPIERS risk stratification and POM) is effective, and if there is compliance with transport to CEmOC facility.

Other outcomes will be evaluated such as knowledge of pre-eclampsia/eclampsia among women of reproductive age; total number of seizures; number of pre-admission and post-admission seizures; adverse events; functional disability; and cost-effectiveness of the CLIP intervention. In addition, other outcomes for the perinatal morbidities are neonatal fever, congenital anomaly and birth injury.

Functional disability for any reason: Inability to perform usual daily duties at specific points in time during the postpartum period that would be acceptable and expected culturally (we are determining these normal limits through local focus group discussions; these will be defined for each country prior to the commencement of each Pilot CLIP Trial)

2.10 MEASURING THE OUTCOME MEASURES AT FOLLOW UP

In total, 54 village and urban clusters will be identified and randomised in the four countries (Nigeria: 10;Mozambique: 12; Pakistan: 20; India: 12). Each cluster will contain a health unit or regional health centre which has provided the training base for the corresponding cHCPs. Subsequently, the 27 clusters chosen to receive the intervention will be analysed against 27 control clusters.

A ***baseline household and facility survey*** is being carried out within the context of the CLIP Feasibility Study in all trial clusters to assess their socioeconomic characteristics and baseline maternal, perinatal, and neonatal mortality rates. In all countries except India this will be performed based on retrospective information on pregnancies occurring in the previous year. In India, the baseline data will draw from the

existing prospective Maternal and Neonatal Health Registry (MNH Registry). In Nigeria, due to the large cluster populations we will perform baseline surveillance on a random sample of households while in all other countries all households will be surveyed. This addresses the human resource constraints identified in Nigeria during the CLIP Feasibility study.

Subsequent ***cross-sectional surveys of all households*** will be conducted at varying frequency depending on current registration and surveillance activities in the relevant country: quarterly (Pakistan), 6-monthly (Nigeria and Mozambique), while in India, household surveys will continue prospectively using the MNH Registry. Although the cHCPs will routinely record information on maternal/perinatal births and deaths, the cross-sectional surveys will be conducted by a separate team to collect data on births, deaths, and care-seeking behaviour in the preceding study epoch.In Nigeria, due to population sizes and human resource constraints the cross-sectional surveys will focus on collection of maternal/ perinatal mortality and morbidity for all households and a random sample of these households will receive a more indepth survey including questions around birth preparedness, care-seeking and pre-ecalmpsia knowledge. The cross-sectional surveys will be considered the gold standard for the Trial as they will be applied equally between control and intervention clusters.

These surveys will permit us to accrue outcome data about women who had a CLIP primary outcome but who did not seek care at a CEmOC facility. These data will be necessary for us to undertand fully the impact of the CLIP intervention on mothers and babies. It is our hope that the CLIP intervention will cause women from the intervention clusters to be treated earlier in the course of their disease and, as a result, have less complicated clinical courses and lower rates of severe morbidity. As such, fewer women may die unknown to the formal health care system but more such women may be referred to the PHC and both BEmOC and CEmOC hospital levels. If that referral occurs in a timely fashion, then it is our hope that women will receive more timely, evidence-based, care (given facility enhancement). As such, it is hoped that overall, maternal and perinatal mortality and morbidity will be decreased. However, it is plausible that despite identification, triage, community treatment, and transport of women with a CLIP trigger, and despite EmOC facility enhancement about pre-eclampsia/eclampsia care, the number of inpatient pre-eclampsia-related maternal and perinatal deaths may rise if women and/or fetuses who previously would have died in their community might now reach hospital moribund and beyond help.

Information on referrals will be collected from the cHCPs and community health committees by the surveillance teams. Surveillance teams will carry out ***verbal and social autopsies of maternal deaths, stillbirths, neonatal deaths***, and similarly constructed reviews of maternal and neonatal morbidity.

In the event of significant barriers to application of the CLIP package or unforeseen events, focus group discussions and/or in-depth interviews and/or surveys will be conducted to better understand relevant barriers or events, in an effort to remedy the situation. Case analysis may also be employed on a select number of maternal or perinatal morbidities or mortalities to uncover greater detail of context of the outcome.

A final aspect of follow-up will be ***facility-based chart audit*** of pregnant women admitted to CEmOC facilities in both intervention and control clusters, throughout the Trial will occur in all but India where the MNH registry system already incorporates a facility chart review for each registered woman. This will allow for triangulation of mortality and morbidity data collected through the the cHCPs and cross-sectional household surveys.

Data collection using mHealth technology

We will develop three mHealth applications to support the CLIP trial. The first will be used in all countries and the second and third will be used in Nigeria, Mozambique and Pakistan during the definitive trial phase.

The first application will be mobile phone-based while the other two will use a tablet-based device.

For intervention clusters only, the first mHealth application is the CLIP POM (described in detail, previously). This will be used to collect routine antenatal visit data and miniPIERS variables. These data will be entered into an integrated decision guide to give real-time advice to cHCPs in terms of appropriate patient management and decisions for interventions (e.g., appropriateness and timing of transport to CEmOC facility, and community initiation of antihypertensive and $MgSO_4$ therapy, as discussed above). Women will be identified both by demographics information and by unique surveillance IDs to enhance identification through the system.

For both intervention and control clusters, the second mHealth application will be a tablet device used by the field team for routine data collection during household surveys (see above). In this way, survey data will be automatically synchronised with a centralised data warehouse, and collected efficiently (given no double handling of paper forms and efficient enforcement of complete data entry). GPS co-ordinates will be provided in real-time to confirm the place and time of each survey event.

For both intervention and control clusters, the third mHealth application will be a tablet device used by an independent team of skilled interviewers who will conduct a detailed verbal and social autopsy / morbidity review when the quarterly surveys reveal the occurrence of an adverse maternal and/or perinatal event (death or major morbidity). The application will be adapted from that developed by the WHO.

Each CLIP site will participate in the design and development of mHealth tools and will conduct usability testing of the final application prototypes before the trial begins in order to ensure acceptability and usability of the tools by users (cHCPs and surveillance teams). Post-trial evaluation of health worker and stakeholder perspectives on the acceptability and usability of the mHealth tools will be performed.

Data on individual women entered into all mHealth applications will be linked using a unique identifier code. This code will be provided to each woman after they consent for the trial on a study ID card. The key for these unique identifiers will be developed after the baseline survey by the Data Manager in each country and will not be shared with the Trial Coordinating Centre at UBC, ensuring privacy and confidentiality of the women is maintained at all times.

Patient data security is at all times of utmost importance, and will thus be maintained throughout mHealth system operations.

- Valid usernames and passwords will be used to limit unauthorized access to device data as well as access to the database and central document archives. The relevant password rules and encryption protocols will be applied.
- Encryption of data is required for any form of storage or transmission between devices and systems.
- Data validation will be performed at every stage of data input as well as on all data access requests to minimize false scripting.

In addition, all safety regulations pertaining to medical diagnostic and monitoring tools will be upheld. Any interfaces between patient, device and user will have the necessary protection systems to ensure the safety of the patient and operator.

2.11 Sample size calculations

Each country is independently powered to assess the chosen primary outcome. The data upon which the estimates have been made are routinely at the conservative end of the published or available range, and were generally provided by the site investigators. However, these calculations will be revisited once the CLIP Feasibility Study maternal and perinatal morbidity and mortality data are in hand. We have planned an individual patient data (IPD) meta-analysis at the completion of each of the individual country trials to

ensure adequate power for an analysis of maternal mortality and morbidity.

Table 3 Sample size calculations (Mozambique to be determined)

**Country**	**Nigeria**	**Mozambique**	**Pakistan**	**India**
**Unit of randomization**	LGA	AP	Union Council	PHC
**Population per unit of randomization**	70,000	25,000	32,000	27,000
**Annual birth rate (/1000/yr)**	16	40	14	22
**Births/2yr**	2240	2000	900	1200
**MMR (/100,000 live births)**	800	388	267	150
**Intra-cluster co-efficient**	0.006†	0.002‡	0.002*	0.001**
**Incidence of maternal & perinatal/neonatal M&M**				
**control clusters**	28.8%	14.0%	9.6%	5.4%
**intervention clusters**	23.0%	11.1%	7.7%	4.3%
**Number of clusters (total)**	10	12	20	12
**Number of births in Definitive CLIP Trial (2 years)**	22,400	24,405	18,000	13,000
**Additional births from Pilot CLIP Trial (4 clusters/1 year)**	4480	n/a	1800	2400
**Total number of births (Pilot & Definitive Trials)**	26,880	24,405	19,800	19,200
**Expected referrals at month 6 of Pilot Trial assuming 5% incidence of HDP**	112 [92, 132]	100 [81, 119]	45 [32, 58]	60 [45, 75]
**Number of women to be referred at month 6 of Pilot Trial to meet 50% use target**	56	50	23	30
**Number of health facilities (total)**				
**PHC/RHC**	120	44	42	22
**EmOC**	10	10		22
**Number of cHCPs (total)**	250 CHEWs & 45 HAs	170	400 LHWs	60 ANMs & 360 ASHAs

ANM: assistant nurse midwife; **AM: Administrative Posts; ASHA**: accredited social health activist; **CHEW**: community health extension worker; **cHCPs**: community health care providers; **CHW** community health worker; **EmOC**, emergency obstetric care; **LGA**: Local Government Area; **LHW**: Lady Health Worker; **M&M**: morbidity and mortality; **MMR**: maternal mortality ratio; **PHC**: Primary Health Centre; **RHC**, rural health centre.

* calculated from Sindhi miniPIERS facility data. †assumes 3-fold increase in ICC over Sindh; ‡assumes same ICC as Sindh (urban population in Maputo); ** assumes half the ICC of Sindh. In these calculations, we have used risks at the lower end of the published ranges for that risk and milieu.

Assumptions

- 10% loss of individual women to follow-up
- no loss of clusters to follow-up
- alpha of 0.05, power ≥0.80
- anticipated effect size of a 20% reduction in all cause maternal and perinatal/neonatal morbidity and mortality
- Also, we have assumed a ratio of maternal morbidity : mortality of 5:1 (may be as high as 10:1), and a ratio of perinatal/neonatal : maternal events of 5:1 (may be as high as 10:1 – permits overlap in outcomes)
- All sample sizes will be supplemented by the data collected in each single country Pilot CLIP Trial.

The combined Definitive CLIP Trials cohort of 878787,480 deliveries in 56 clusters over 2 years (+16 cluster years in Pilot CLIP Trial), averaging 1376 deliveries per cluster per year, will provide the following power for the IPD meta-analysis:

Maternal & perinatal/neonatal mortality & morbidity: assuming a 20% effect size (incidence reduced from 15.6% to 12.4%), we would have 80% power with an ICC of 0.015.

Maternal mortality & *morbidity* (primary outcome for the IPD meta-analysis): assuming a 20% effect size (incidence reduced from 2.6% to 2.1%), we would have 80% power with an ICC of 0.001.

Alternatively, assuming a 30% effect size (incidence reduced from 2.6% to 1.8%), we would have 80% power with an ICC of 0.004. We hope to reduce the adverse maternal event rate by ≥30%, primarily through community mobilisation and antenatal care by cHCPs.

Finally, we will pool adjusted results for the individual CLIP Trials using the generic inverse variance method developed by the Cochrane Collaboration.

This sample size will be supplemented by the women recruited during the pilot phase (primary outcome: package utilisation), who will be 'recycled' into the Definitive Trial phase.

2.12 RECRUITMENT RATE

Given the populations in these centres and the fertility rates in these communities, we are confident that the sample size will be achieved in a 24 month period (see section 2.11, above) and the Trials will complete recruitment by March 2017. We will pilot the use of ***contact tools*** to maintain contact, with quarterly contact maintenance rate reports (e.g., birthday cards and newsletters). Thus, we will maintain a group of mothers and children available for piloting further assessment of the impact of the CLIP intervention at school age (funding for follow-up studies will be sought).

2.13 COMPLIANCE WITH THE CLIP INTERVENTION

Through the trial, we will measure ***compliance with community mobilisation*** in terms of the number of community meetings held around pre-eclampsia/eclampsia content, prior permission to seek care, and/or community transport plans/funds.

Throughout the trial period, we will measure ***compliance with the CLIP visits, use of the POM tool, and use of the CLIP package for women with a CLIP trigger*** based on the following criteria: (i) appropriate frequency of CLIP visits by the cHCP (i.e., 4 ± 2 weeks); (ii) N (%) of women who had a CLIP trigger and who were administered treatment as indicated (methyldopa and/or $MgSO_4$); (iii) N (%) of women who had a CLIP trigger and were referred for transport as appropriate, and (iv) N (%) of women who were seen at an CEmOC facility within the appropriate period of time after referral advice given (i.e., 4hr for urgent transport or 24hr for non-urgent transport).

We have assumed a non-compliance rate in appropriate use of the intervention package of 10% in our power calculation. The primary reason for this will be lack of confidence by the cHCP in administering the intervention. We believe this to be a probable overestimate given the existing experience in Sindh where compliance with more technically challenging protocols exceeded 90%.

2.14 LOSS TO FOLLOW-UP

We have assumed a 10% loss to follow-up in our power calculations. The primary reason for this will be women leaving the marital home in one community for their childhood home in another community for labour and delivery.

2.15 TYPES OF ANALYSES/STATISTICAL PLAN

Note that this is an intent-to-treat analysis; all analyses will include all patients randomised. A detail of analysis plan is included in the CLIP cRCT Statistical Analysis Plan.

Trial information and patient disposition

The following information will be presented:

- Number of patients randomised
- Number of patients completing each visit of follow-up
- Median follow-up time
- Frequency table for the reasons for going off-study
- Frequency table for loss to follow-up

Baseline and demographic variables

Summary statistics such as the mean, median, standard deviation, minimum, maximum and inter-quartile range for continuous variables and the number and percentage of patients with various levels of categorical variables will be calculated for ***all subjects randomised within each treatment group for each country*** for variables such as:

- Demographics: maternal age, parity, gestational age at diagnosis, distance from facility
- Socioeconomic status (measured by poverty index), level of education
- Cluster level variables: cHCP density, population density
- Interventions: number of antenatal visits, type of care provider, route of birth, medications given

All summary statistics will be calculated for each treatment group individually as well as for all treatment groups combined.

Primary outcome

The primary outcome is the proportion of pregnancies resulting in at least one of maternal death, stillbirth, neonatal death, or severe morbidity in the mother or child.

Primary analysis

A hierarchical regression model will be used to take into account the clustering of women within the units of randomisation. The model will take into account key determinants of outcome, as defined by the investigators, at both the individual and cluster level.

Sensitivity analyses

We will use multiple imputation to account for those lost to follow-up and examine whether this has any impact on the between-group comparisons.

Secondary outcome

Similar methods will be applied to the individual components of the primary outcome. In addition, the proportion of women achieving birth preparedness and complication readiness will be compared as will be the proportion of women presenting for care at an EmONC facility, and the proportion of facility births.

Other outcomes

Knowledge of pre-eclampsia and eclampsia; total number of seizures or number of pre-admission and post-admission seizures and cost-effectiveness of the intervention.

Safety data

Adverse events will be tracked in intervention and control clusters related to:

- **Methyldopa** administration in the community: relative maternal hypotension on arrival at facility (defined as sBP<110 mmHg)
- **MgSO4** administration in the community: either respiratory depression, coma or death during transport, as diagnosed upon arrival at facility

- **Transport**: Transport-related injury (life or limb) or death during transport
- injection site haematoma or infection (following either community or facility administration of i.m. $MgSO_4$)
- ≥ 20% of women referred to facility being sent back to their communities without follow-up (monitoring community engagement and the CLIP POM).
- The following will be presented by treatment group: number of adverse events (overall and by type), number of women with one or more adverse event(s) (overall and by type).

Additional methodological details

Statistical tests

All statistical tests will be two-sided, with significance levels of 0.05. Comparisons will be by means of risk ratios and mean differences with 95% confidence intervals for each site.

Software to be used

SAS / R / S-plus.

Individual patient data meta-analysis (IPD)

Each country will be analysed separately, with a planned IPD meta-analysis (conducted at UBC) after the completion of all four trials. For the IPD meta-analysis, we will analyse data for women recruited at ≥20 weeks, to standardise data to the latest public declaration of pregnancy (i.e., 20 weeks in Pakistan).

Economic analyses

An economic evaluation of the intervention and its impact will be performed alongside the trial from a societal perspective (accounting for both costs to health care system and families) to guide health services decision-making for post-trial programme scale-up in the selected countries. The cost to the health care system will comprise both direct and in-direct cost of patient care borne by health system, cost of the CLIP package of interventions, and follow-up household visits by cHCP. The cost to the families of pregnant women will comprise out-of-pocket expenses for patient care, transport, and cost of lost productivity resulting from morbidity or mortality of patients with or without paid jobs. The information about individual level resource/service utilization will be prospectively collected alongside the trial; whereas, secondary data sources (i.e., budgetary reviews) will be utilized to estimate the unit cost for each resource/service utilized in both intervention and control clusters. Besides, the qualitative focus group sessions will be conducted with women identified at risk of HDP, their husbands, community level health care providers, referral health facility care providers and district level health decision/policy makers to inform the design of cost modeling and interpret cost-benefit from community perspectives. (Full details of this plan are provided in Appendix F).

Economic analysis will be performed to calculate incremental cost-effectiveness ratio (ICERs) to compare the cost per unit maternal deaths/adverse pregnancy outcomes averted when switching from standard care to the CLIP package of interventions. Given the uncertainties involved in calculating the costs and trial outcomes, we will use simplistic sensitivity analysis to plot cost-effectiveness ratios. The confidence region surrounding the cost-effectiveness ratio will be estimated using appropriate statistical methods, including bootstrap and Monte-Carlo analyses. The qualitative data will be analyzed using QSR NVivo v10 software, and responses will be coded to form similar categories. Thematic analyses will be performed to underscore trial implementation challenges, perceptions of cost-benefit, strategies for knowledge translation and policy advocacy to be able to interpret the economic impact from societal perspective.

Geo-temporal analyses

We will develop and test a location sensitive maternal health risk index (development funded by CIHR). This index will chart risk for adverse maternal outcomes across multiple communities with different geographic characteristics. We will use Geographical Information Systems to identify 'resilient' communities and individuals and then link environmental correlates related to maternal health with pockets of resilience. Likewise, we will use environmental correlates and outcome data to identify areas and individuals who have poorer than expected outcomes. We will use the data collected in different communities over time to create temporally dynamic map products that chart environmental correlates in relation to shifting maternal morbidity and mortality.

Geospatial analysis will be conducted in each country by an individual from the PRE-EMPT Vancouver team with help from local GIS experts. This analysis will entail using the GPS tagged survey record to chart trends in maternal mortality and morbidity while cross referencing these outcomes to aspects of the built environment like transport and access to health care. Prior to this analysis, all personal identifiers would have been removed from the data. The data will be aggregated to describe frequencies for each of the captured variables at village level. This second set of aggregate data will be then be further analysed at UBC.

Qualitative analyses

We will use survey tools, FGDs and IDIs to explore feasibility and acceptability of use of the blood pressure device in CLIP in collaboration with the CRADLE study team. Full details of this plan are provided in Appendix E. In addition, a cross-sectional survey of cHCPs and physicians along with interviews of randomly selected women enrolled in the CLIP study and their families will be completed to understand the impact of the CLIP intervention package on cHCP decision making confidence.

A similar mixed methods approach will be utilized to evaluate the usability and acceptability of the PIERS on the Move tool and to explore the impact of use of the mobile tool on health worker knowledge, self-efficacy and role within the health system, from their perspectives.

Furthermore, to enhance the understanding of the health policy environment, we aim to undertake a policy analysis to evaluate whether the national policies in the state of Ogun, Nigeria respond to the needs of postpartum women i.e. how they address any health condition attributed to and/or aggravated by pregnancy and childbirth that has a negative impact on the woman's wellbeing following childbirth. Further details about this policy analyses can be seen in Appendix G.

2.16 Frequency of analyses

An interim analysis is planned for each of the three countries once complete data (until 42 days postpartum) has been received for women making up half of the planned sample size for that country. In the event that the site is unable to reach planned sample size, due to logistical and pragmatic challenges, then the interim analysis will be conducted once complete pregnancies (until 42 days postpartum) are expected for women making up half of the projected sample size for that country.

The stopping rule for both benefit and harm will require an observed difference between groups associated with an alpha <0.001 (power: 80%). Reporting and handling of adverse events will be in accordance with Good Clinical Practice (GCP) guidelines.

A single economic analysis will be performed.

2.17 Subgroup analyses

We will analyse the interventions and outcomes by country and locality (i.e., Ogun State, Maputo and Gaza Provinces, Sindh Province, and Karnataka State).

SECTION 3 TRIAL MANAGEMENT

3.1 PRE-RECRUITMENT PHASE

Currently, we are conducting the CLIP Feasibility Study in the relevant Nigerian, Mozambican, Pakistani, and Indian sites. We will finalize a tailored intervention that will be culturally sensitive, effective at reducing barriers, and increase enthusiasm for the CLIP package amongst decision makers through respectful dialogue with professional associations and government decision makers.

An educational package will be developed, and the cHCPs, midwives and physicians working in the intervention clusters will be trained.

Having spent the first 30 months of the PRE-EMPT grant completing the PIERS modelling, trial design and registration, feasibility and barriers assessment, cHCP, nurse, midwife and physician training, and ethics and contracts processes, we will commence recruiting to the trial after July 2013.

3.2 DATA SAFETY AND MONITORING BOARD

The *DSMB* will be created, including methodological and content expertise.

The role of DSMB will be to deal with any ethical issues that may arise while the trial is in progress and to decide either benefit or harm at the planned interim analyses. SAEs will be reported to the DSMB as outlined below. SAEs should be: **(i) serious, (ii) unexpected (in nature, severity, or frequency), and (iii) thought to be related to the study intervention.** This plan reflects the expected nature of the other AE, which are events of note but that do not necessitate stoppage of the trial due to safety concerns prior to the interim analysis.

SAE reporting

When all required information has been gathered in the reporting field site, the field site supervisor will forward 'blocked' copies (without patient, site or allocated intervention group identifiers) of all documentation and an updated 'Serious Adverse Event Form' to the National CLIP Trial Co-ordinating Centre. That centre will assess whether or not: (i) additional information is required, and (ii) the National Principal Investigator and Co-ordinator should be informed immediately, or at the next scheduled meeting. *The* ***documents to be reviewed*** *are: (i) all data forms (including those derived from the POM tool) for each women with a reported primary outcome (for mother or baby), masked to the cluster allocation, and (ii) copies of the relevant facility documents (if applicable) that serve as the 'source documents' and detail the outcome for the woman/baby. If the outcome were reported in the community with no associated facility admission, then the data forms should be reviewed for consistency and to ensure that the woman's story make sense from a clinical perspective.*

After review (and collection of additional information, as applicable) of a reported SAE by the relevant National CLIP PI and National CLIP Trial Co-ordinating Centre, the 'blocked' documentation (including the SAE Form and summary of the case) **should be reviewed by the in-country National Outcomes Adjudication Committee. That Committee will decide whether or not the reported SAE is a true SAE or a pre-specified Trial outcome. Then**, the 'blocked' documentation (including the SAE Form and summary of the case) and the Committee's opinion should be sent to the UBC Trial Co-ordinating Centre (TCC) for review by the UBC Outcomes Adjudication Committee; if UBC agrees that the reported SAE is a true SAE, UBC will send the report to the DSMB for urgent review. However, if UBC agrees that the reported SAE is a pre-specified Trial outcome, then the report will be sent to the DSMB at the time of the next analysis.

The UBC Outcomes Adjudication Committee decision will be reviewed by the CLIP Trial Steering Committee (SC) at the next scheduled quarterly teleconference, or earlier if the UBC Outcomes Adjudication Committee feels that this is appropriate. Possible reasons for urgent review by the SC are

because the reported SAE (i) is not a pre-specified trial outcome (i.e. it is deemed to be a true SAE), or because (ii) is a pre-specified trial outcome but the UBC Outcomes Adjudication Committee has further concerns. After review of the SAE by these groups, the UBC TCC will generate and distribute a summary report of the Serious Unexpected Event(s) to be reported and reviewed by the DSMB at the time of the interim analyses, or earlier if either the UBC Outcomes Adjudication Committee and/or SC have additional concerns.

The summary report should include the 'Serious Unexpected Event Form' received from the site, and a typed summary of all accumulated supporting documentation for each reported event. Ensure all patient, site or allocated intervention group identifiers have been removed. The DSMB can then request further information if they wish to take a closer look at any particular event.

For reported events ruled by the Outcomes Adjudication Committee to be pre-specified trial outcomes (and NOT true SAEs), the UBC TCC will: (i) communicate with the reporting site to let them know about the outcome of the evaluation of their reported Serious Unexpected Event by the UBC Outcomes Adjudication Committee; (ii) inform them that the reported event will also be reviewed by the SC and included in a summary report made to the DSMB at the time of the next scheduled analysis (after DSMB review, a written summary of all reported Serious Unexpected Events, and the DSMB's ultimate assessment of these events, will be distributed to their site (and all sites)); and (iii) invite the site to inform the TCC if they have any concerns.

For reported events ruled by the UBC Outcomes Adjudication Committee and SC to be true SAEs, UBC TCC will: (i) communicate with the reporting site to let them know about the outcome of the evaluation of their reported Serious Unexpected Event by the UBC Outcomes Adjudication Committee and Steering Committee; (ii) inform them that the reported event will also be included in a summary report made to the DSMB (either immediately or at the time of the next scheduled analysis, as decided by the Steering Committee) (after DSMB review, a written summary of all reported Serious Unexpected Events, and the DSMB's ultimate assessment of these events, will be distributed to their site (and all sites)); and (iii) invite the site to inform the UBC TCC if they have any concerns.

For all reported events, following review by the DSMB, the Principal Investigator will prepare a written summary of the reported Serious Unexpected Event(s), and the DSMB's assessment of these event(s). If the event(s) is (are) deemed to be a true SAE, the anonymised SAE Report Form(s) will also be attached. This report will be submitted to: (i) UBC REB. As the Clinical and Data Co-ordinating Centre, UBC will inform the UBC REB of all reported Serious Unexpected Events; (ii) the study sponsor, BMGF, will be informed through the routine monthly/alternate monthly dialogue; and (iii) all Site Investigators, including the reporting Investigator. Each Site Investigator will be responsible for reviewing this Serious Unexpected Event summary report and forwarding it to their local REB. It will be recommended that each Site Investigator check with their local REB for other local reporting requirements.

Membership of the DSMB is:

- **Professor Eileen Hutton (Chair)**

  Associate Professor, Department of Obstetrics and Gynaecology, Assistant Dean, Faculty Health Sciences, and Director, Midwifery Education Program, McMaster University, Hamilton, ON, Canada

  (http://fhs.mcmaster.ca/ceb/faculty_member_hutton.htm).

- **Lehana Thabane**

Professor and Associate Chair, Department of Clinical Epidemiology & Biostatistics, Director, Biostatistics Unit, Centre for Evaluation of Medicine, and Senior Scientist, Population Health Research Institute (PHRI), Hamilton Health Sciences, McMaster University (http://fhs.mcmaster.ca/ceb/faculty_member_thabane.htm).

- **Romano Byaruhanga**

  Consultant Obstetrician, Nsambya, Uganda and President of the Association of Obstetricians and Gynaecologists of Uganda.

  (http://www.sogc.org/aogu/index.aspx?contentID=41).

- **Mario Merialdi**

  Senior Director, Maternal and Newborn Health, Global Health, Becton, Dickinson and Company

- **Brian A. Darlow**

  CureKids Professor of Paediatric Research, University of Otago Christchurch, PO Box 4345 Christchurch 8140, New Zealand

  (http://www.otago.ac.nz/healthsciences/expertise/profile/index.html?id=229)

3.3 Outcome Adjudication committee

Each country will perform a masked review of all occurrences of the primary outcome that is embedded in the data collection process; the national team will review outcomes where there is uncertainty. *The **National Outcomes Adjudication Committee** will consist of an uneven number of members of the national CLIP Team, including but not limited to one obstetrician, one paediatrician, and one methodologist/trialist.* The UBC management team will provide final arbitration if required.

3.4 Trial registration

The CLIP Trial has been registered with clinicaltrials.gov (ID NCT01911494).

3.5 General assumptions

We assume that the results of the CLIP Feasibility Studies being conducted in Nigeria, Mozambique, Pakistan, and India will inform some of the details of the CLIP trial, including costs. Therefore, each Pilot CLIP Trial will not be started until the relevant CLIP Feasibility Study is completed.

We have assumed that the clinical and data co-ordinating centre will be located at UBC, Vancouver, where the statistical analyses will also be performed. We do not anticipate any delays related to gaining IRB approval for this intervention.

For the CLIP Feasibility Studies, we have received firm stakeholder support and involvement at the ministerial level of health care in the identified countries. We assume that each of the three stages of the relevant CLIP Feasibility Study will be completed according to the specified timelines and budget. In addition, we have assumed the established pattern of community level cRCTs in Nigeria, Mozambique, Pakistan, and India will support the CLIP Trial, including cHCP-based BP control.

It is our intention that the results of the household surveys and registries will provide a replete community-

level dataset for each participating centre. These datasets will span not only HDP-related deaths and morbidities, but also the other leading causes of maternal mortality (obstetric haemorrhage, obstructed labour, and puerperal sepsis), and will be invaluable resources for local communities, local investigators, demographers, and scientists. Whatever the results of the CLIP Trial, the data will be novel and important and we anticipate the acceptance of resulting paper(s) for publication. We have assumed that no natural or human-caused disaster will occur to curtail our work.

References

(1) Steegers EA, von Dadelszen P, Duvekot JJ, Pijnenborg R. Pre-eclampsia. *Lancet* 2010.

(2) Hutcheon JA, Lisonkova S, Magee LA, von Dadelszen P, Woo HL, Liu S et al. Optimal timing of delivery in pregnancies with pre-existing hypertension. *BJOG* 2011; 118(1):49-54.

(3) Duley L, Gulmezoglu AM, Henderson-Smart DJ, Chou D. Magnesium sulphate and other anticonvulsants for women with pre-eclampsia. *Cochrane Database Syst Rev* 2010;(11):CD000025.

(4) Duley L, Henderson-Smart DJ, Walker GJ, Chou D. Magnesium sulphate versus diazepam for eclampsia. *Cochrane Database Syst Rev* 2010;(12):CD000127.

(5) Duley L, Henderson-Smart DJ, Chou D. Magnesium sulphate versus phenytoin for eclampsia. *Cochrane Database Syst Rev* 2010;(10):CD000128.

(6) Duley L, Gulmezoglu AM, Chou D. Magnesium sulphate versus lytic cocktail for eclampsia. *Cochrane Database Syst Rev* 2010;(9):CD002960.

(7) Magee LA, Abalos E, von Dadelszen P, Sibai B, Easterling T, Walkinshaw S. How to manage hypertension in pregnancy effectively. *Br J Clin Pharmacol* 2011; 72(3):394-401.

(8) Bhutta ZA, Soofi S, Memon Z. Training for Lady Health Workers clarified. *Bull World Health Organ* 2008; 86(12):B.

(9) Bhutta ZA, Memon ZA, Soofi S, Salat MS, Cousens S, Martines J. Implementing community-based perinatal care: results from a pilot study in rural Pakistan. *Bull World Health Organ* 2008; 86(6):452-459.

(10) Bhutta ZA, Rizvi A, Raza F, Hotwani S, Zaidi S, Moazzam HS et al. A comparative evaluation of multiple micronutrient and iron-folic acid supplementation during pregnancy in Pakistan: impact on pregnancy outcomes. *Food Nutr Bull* 2009; 30(4 Suppl):S496-S505.

(11) Bhutta ZA, Lassi ZS. Empowering communities for maternal and newborn health. *Lancet* 2010; 375(9721):1142-1144.

(12) Bhutta ZA, Soofi S, Cousens S, Mohammad S, Memon ZA, Ali I et al. Improvement of perinatal and newborn care in rural Pakistan through community-based strategies: a cluster-randomised effectiveness trial. *Lancet* 2011; 377(9763):403-412.

(13) Elwyn G, Legare F, van der Weijden T, Edwards A, May C. Arduous implementation: does the Normalisation Process Model explain why it's so difficult to embed decision support technologies for patients in routine clinical practice. *Implement Sci* 2008; 3:57.

(14) May C. A rational model for assessing and evaluating complex interventions in health care. *BMC Health Serv Res* 2006; 6:86.

(15) May C, Finch T, Mair F, Ballini L, Dowrick C, Eccles M et al. Understanding the implementation of complex interventions in health care: the normalization process model. *BMC Health Serv Res* 2007; 7:148.

(16) Michie S, Johnston M, Abraham C, Lawton R, Parker D, Walker A. Making psychological theory useful for implementing evidence based practice: a consensus approach. *Qual Saf Health Care* 2005; 14(1):26-33.

(17) Thaddeus S, Maine D. Too far to walk: maternal mortality in context. *Soc Sci Med* 1994; 38(8):1091-1110.

(18) Payne B, Hutcheon JA, Qu Z, Haniff F, Bhutta Z, Biryabarema C et al. miniPIERS (Pre-eclampsia Integrated Estimate of RiSk): development of a clinical prediction model for use in low and middle income countries (LMICs) [abstract]. *Pregn Hypertens* 2012; 2(3):195-196.

(19) von Dadelszen P, Payne B, Li J, Ansermino JM, Broughton PF, Cote AM et al. Prediction of adverse maternal outcomes in pre-eclampsia: development and validation of the fullPIERS model. *Lancet* 2011; 377(9761):219-227.

(20) Cote AM, Firoz T, Mattman A, Lam EM, von Dadelszen P, Magee LA. The 24-hour urine collection: gold standard or historical practice? *Am J Obstet Gynecol* 2008; 199(6):625-626.

(21) Firoz T, Magee LA, Payne BA, Menzies JM, von Dadelszen P. The PIERS experience: research or quality improvement? *J Obstet Gynaecol Can* 2012; 34(4):379-381.

(22) Kozic JR, Benton SJ, Hutcheon JA, Payne BA, Magee LA, von Dadelszen P. Abnormal liver function tests as predictors of adverse maternal outcomes in women with preeclampsia. *J Obstet Gynaecol Can* 2011; 33(10):995-1004.

(23) Laskin S, Payne B, Hutcheon JA, Qu Z, Douglas MJ, Ford J et al. The role of platelet counts in the assessment of inpatient women with preeclampsia. *J Obstet Gynaecol Can* 2011; 33(9):900-908.

(24) Millman AL, Payne B, Qu Z, Douglas MJ, Hutcheon JA, Lee T et al. Oxygen saturation as a predictor of adverse maternal outcomes in women with preeclampsia. *J Obstet Gynaecol Can* 2011; 33(7):705-714.

(25) Payne B, Magee LA, Cote AM, Hutcheon JA, Li J, Kyle PM et al. PIERS proteinuria: relationship with adverse maternal and perinatal outcome. *J Obstet Gynaecol Can* 2011; 33(6):588-597.

(26) Payne B, Hodgson S, Hutcheon J, Joseph K, Li J, Lee T et al. Performance of the fullPIERS model in predicting adverse maternal outcomes in pre-eclampsia using patient data from the PIERS (Pre-eclampsia Integrated Estimate of RiSk) cohort, collected on admission. *BJOG* 2012.

(27) Payne BA, Kyle PM, Lisonkova S, Magee LA, Pullar B, Qu Z et al. An assessment of predictive value of the biophysical profile in women with preeclampsia using data from the fullPIERS database. *Pregnancy Hypertens* 2013; [in press].

(28) Yen TW, Payne B, Qu Z, Hutcheon JA, Lee T, Magee LA et al. Using clinical symptoms to predict adverse maternal and perinatal outcomes in women with preeclampsia: data from the PIERS (Pre-eclampsia Integrated Estimate of RiSk) study. *J Obstet Gynaecol Can* 2011; 33(8):803-809.

(29) Magee LA, von Dadelszen P. The management of severe hypertension. *Semin Perinatol* 2009; 33(3):138-142.

(30) Tuffnell DJ, Jankowicz D, Lindow SW, Lyons G, Mason GC, Russell IF et al. Outcomes of severe pre-eclampsia/eclampsia in Yorkshire 1999/2003. *BJOG* 2005; 112(7):875-880.

(31) National Collaborating Centre for Women's and Children's Health. Hypertension in pregnancy: the management of hypertensive disorders during pregnancy. CG107. 2010. London, National Institute for Health and Clinical Excellence.

Ref Type: Report

(32) Magee LA, Miremadi S, Li J, Cheng C, Ensom MH, Carleton B et al. Therapy with both magnesium sulfate and nifedipine does not increase the risk of serious magnesium-related maternal side effects in women with preeclampsia. *Am J Obstet Gynecol* 2005; 193(1):153-163.

(33) Chesley LC, TEPPER I. Plasma levels of magnesium attained in magnesium sulfate therapy for preeclampsia and eclampsia. *Surg Clin North Am* 1957; 37(2):353-367.

(34) World Health Organization. World Health Organization recommendations for preventation and treatment of pre-eclampsia and eclampsia. 2011. Geneva, World Health Organization.

Ref Type: Report

(35) Chiwuzie J, Okojie O, Okolocha C, Omorogbe S, Oronsaye A, Akpala W et al. Emergency loan funds to improve access to obstetric care in Ekpoma, Nigeria. The Benin PMM Team. *Int J Gynaecol Obstet* 1997; 59 Suppl 2:S231-S236.

(36) Essien E, Ifenne D, Sabitu K, Musa A, Alti-Mu'azu M, Adidu V et al. Community loan funds and transport services for obstetric emergencies in northern Nigeria. *Int J Gynaecol Obstet* 1997; 59 Suppl 2:S237-S244.

(37) Fofana P, Samai O, Kebbie A, Sengeh P. Promoting the use of obstetric services through community loan funds, Bo, Sierra Leone. The Bo PMM Team. *Int J Gynaecol Obstet* 1997; 59 Suppl 2:S225-S230.

(38) Shehu D, Ikeh AT, Kuna MJ. Mobilizing transport for obstetric emergencies in northwestern Nigeria. The Sokoto PMM Team. *Int J Gynaecol Obstet* 1997; 59 Suppl 2:S173-S180.

(39) Sadovsky E, Ohel G, Havazeleth H, Steinwell A, Penchas S. The definition and the significance of decreased fetal movements. *Acta Obstet Gynecol Scand* 1983; 62(5):409-413.

(40) Duley L, Henderson-Smart DJ, Walker GJ, Chou D. Magnesium sulphate versus diazepam for eclampsia. *Cochrane Database Syst Rev* 2010;(12):CD000127.

(41) Duley L, Henderson-Smart DJ, Chou D. Magnesium sulphate versus phenytoin for eclampsia. *Cochrane Database Syst Rev* 2010;(10):CD000128.

(42) Duley L, Gulmezoglu AM, Chou D. Magnesium sulphate versus lytic cocktail for eclampsia. *Cochrane Database Syst Rev* 2010;(9):CD002960.

(43) Duley L, Matar HE, Almerie MQ, Hall DR. Alternative magnesium sulphate regimens for women with pre-eclampsia and eclampsia. *Cochrane Database Syst Rev* 2010;(8):CD007388.

(44) Department of Making Pregnancy Safer. Pregnancy, childbirth, postpartum and newborn care: a guide for essential practice. B13. 2006. Geneva, World Health Organization. Integrated management of pregnancy and childbirth.
Ref Type: Serial (Book,Monograph)

(45) Begum MR, Begum A, Quadir E. Loading dose versus standard regime of magnesium sulfate in the management of eclampsia: a randomized trial. *J Obstet Gynaecol Res* 2002; 28(3):154-159.

(46) Begum R, Begum A, Johanson R, Ali MN, Akhter S. A low dose ("Dhaka") magnesium sulphate regime for eclampsia. *Acta Obstet Gynecol Scand* 2001; 80(11):998-1002.

(47) Bhattacharjee N, Saha SP, Ganguly RP, Patra KK, Dhali B, Das N et al. A randomised comparative study between low-dose intravenous magnesium sulphate and standard intramuscular regimen for treatment of eclampsia. *J Obstet Gynaecol* 2011; 31(4):298-303.

(48) Darngawn L, Jose R, Regi A, Bansal R, Jeyaseelan L. A shortened postpartum magnesium sulfate prophylaxis regime in pre-eclamptic women at low risk of eclampsia. *Int J Gynaecol Obstet* 2012; 116(3):237-239.

(49) Duley L, Matar HE, Almerie MQ, Hall DR. Alternative magnesium sulphate regimens for women with pre-eclampsia and eclampsia. *Cochrane Database Syst Rev* 2010;(8):CD007388.

(50) Malapaka SV, Ballal PK. Low-dose magnesium sulfate versus Pritchard regimen for the treatment of eclampsia imminent eclampsia. *Int J Gynaecol Obstet* 2011; 115(1):70-72.

(51) Regmi MC, Aggrawal A, Pradhan T, Rijal P, Subedi A, Uprety D. Loading dose versus standard regimen of magnesium sulphate in eclampsia--a randomized trial. *Nepal Med Coll J* 2010; 12(4):244-247.

(52) Al Haque N, Chowdhury ME, Huq NL, Ahmed A, DasGupta SK, Quaiyum MA. Baseline findings: Shahjadpur integrated maternal and neonatal health project. 2010. Dhaka, ICDDR,B.
Ref Type: Report

(53) Huq NL, Moran AC, Quaiyum MA, Chowdhury ME, Ahmed A, Al Haque N. Formative research findings: Shahjadpur integrated maternal and neonatal health project. 2010. Dhaka, ICCDR,B.
Ref Type: Report

(54) Lu JF, Nightingale CH. Magnesium sulfate in eclampsia and pre-eclampsia: pharmacokinetic principles. *Clin Pharmacokinet* 2000; 38(4):305-314.

(55) Koopmans CM, Bijlenga D, Groen H, Vijgen SM, Aarnoudse JG, Bekedam DJ et al. Induction of labour versus expectant monitoring for gestational hypertension or mild pre-eclampsia after 36 weeks' gestation (HYPITAT): a multicentre, open-label randomised controlled trial. *Lancet* 2009; 374(9694):979-988.

(56) Mozambique. *Popul Policy Compend* 1982;1-7.

(57) Chapman RR. Endangering safe motherhood in Mozambique: prenatal care as pregnancy risk. *Soc Sci Med* 2003; 57(2):355-374.

(58) Sevene E, Lewin S, Mariano A, Woelk G, Oxman AD, Matinhure S et al. System and market failures: the unavailability of magnesium sulphate for the treatment of eclampsia and pre-eclampsia in Mozambique and Zimbabwe. *BMJ* 2005; 331(7519):765-769.

(59) Bhutta ZA, Ali N, Hyder AA, Wajid A. Perinatal and newborn care in Pakistan: seeing the unseen. In: Bhutta ZA, editor. Maternal and child health in Pakistan: challenges and opportunities. Karachi: Oxford University Press; 2004.

(60) Duley L, Henderson-Smart DJ, Meher S. Drugs for treatment of very high blood pressure during pregnancy. *Cochrane Database Syst Rev* 2006; 3:CD001449.

(61) Magee LA, Cham C, Waterman EJ, Ohlsson A, von Dadelszen P. Hydralazine for treatment of severe hypertension in pregnancy: meta-analysis. *BMJ* 2003; 327(7421):955-960.

(62) Souza LM, Riera R, Saconato H, Demathe A, Atallah AN. Oral drugs for hypertensive urgencies: systematic review and meta-analysis. *Sao Paulo Med J* 2009; 127(6):366-372.

(63) Brown MA, Buddle ML, Farrell T, Davis GK. Efficacy and safety of nifedipine tablets for the acute treatment of severe hypertension in pregnancy. *Am J Obstet Gynecol* 2002; 187(4):1046-1050.

(64) Altman D, Carroli G, Duley L, Farrell B, Moodley J, Neilson J et al. Do women with pre-eclampsia, and their babies, benefit from magnesium sulphate? The Magpie Trial: a randomised placebo-controlled trial. *Lancet* 2002; 359(9321):1877-1890.

Table 4 CLIP interventions

Level of care/foci	**Care component**
**cHCPs**	
**Triage**	◊ Community education: symptoms, signs, & consequences of pre-eclampsia<br>◊ Community engagement & mobilization: transport funds; prior permissions from proxy decision-makers; health system managers & funders<br>◊ Menstrual calendars to be held by married women of reproductive age (South Asia) or all women of reproductive age (Africa)<br>◊ Urine dipstick at first antenatal encounter<br>◊ Symphysis-fundal height at first antenatal encounter ≥24^{+0} weeks to assess GA if unknown<br>◊ Ongoing antenatal surveillance (BP [& urine dipsticks, if indicated] as described in the protocol)<br>◊ Opportunistic screening & triage of symptomatic women<br>◊ Diagnostic & triage tool (miniPIERS)<br>◊ PIERS on the Move decision aid Android application
**Treatment**	◊ 750mg α-methyldopa p.o. for severe hypertension (sBP ≥160mmHg); single dose only<br>◊ 10g $MgSO_4$ IM loading dose (single dose only) for women with stroke, eclampsia (single or recurrent seizures), vaginal bleeding (presumed severe pre-eclampsia), severe hypertension (presumed severe pre-eclampsia), or a miniPIERS probability ≥25%
**Transport**	◊ Arrangements made for patient transfer to nearest referral centre<br>o High risk: within 4h<br>o Low risk: within 24h<br>◊ Discussion with referral centre<br>◊ Patient transferred
**Primary health centre**	
**Triage**	◊ Menstrual calendars to be held by married women of reproductive age (South Asia) or all women of reproductive age (Africa)<br>◊ Symphysis-fundal height at first antenatal encounter ≥24^{+0} weeks to assess GA<br>◊ Ongoing antenatal surveillance (BP and urine dipsticks every 4 weeks)<br>◊ Opportunistic screening & triage of symptomatic women<br>◊ Diagnostic & triage tool (miniPIERS)<br>◊ PIERS on the Move decision aid Android application
**Treatment**	◊ 750mg α-methyldopa p.o. for severe hypertension (sBP ≥160mmHg); single dose only (not repeated if already administered by cHCP in community)<br>◊ 10g $MgSO_4$ IM loading dose (single dose ONLY if not already administered by cHCP; no repeat dosing) for women with stroke, eclampsia (single or recurrent seizures), vaginal bleeding (presumed severe pre-eclampsia), severe hypertension (presumed severe pre-eclampsia), or a miniPIERS probability ≥25%
**Transport**	◊ Arrangements made for patient transfer to nearest EmOC facility<br>◊ Patient transferred
**Hospital providing CEmOC– unfunded by CLIP but expected standard of care (supported by planned facility enhancement)**	
**Triage**	◊ Triage tool (miniPIERS and/or fullPIERS)
**Treatment**	◊ 200mg labetalol p.o. or 10mg nifedipine p.o. or hydralazine 5mg IV for severe hypertension (sBP ≥160mmHg); maintenance therapy titrated against response; parenteral antihypertensives as required<br>◊ $MgSO_4$ loading dose (IV/IM if not already administered in the community or 10g IM MgSO4 was administered more than 4 hr prior to facility admission) for women with severe hypertension or vaginal bleeding (as indicators of severe pre-eclampsia) or eclampsia (single); MgSO4 dose of 2g IV for women who had a seizure after the 10g IM MgSO4 was administered in the community. Then, maintenance therapy (usually 1g/h) until 24 hours postpartum.<br>◊ Arrangements made for definitive therapy<br>◊ If >local threshold for viability (e.g., 28 weeks' by best clinical estimate), <34+0 weeks', and PIERS probability <25%: administer 12mg dexamethasone i.m. x 2 12h apart, wait 48h and deliver (transfer to regional centre, if relevant)<br>◊ If <viability, ≥34+0 weeks', or PIERS probability ≥25%: deliver
**Transport (as required)**	◊ Arrangements made for patient transfer to regional (e.g., Hyderabad DHQ) or supraregional (e.g., AKU Medical Centre/JPMC) referral centres, if indicated<br>◊ Patient transferred

AKU Aga Khan University; **BP** blood pressure; **CPD** continuing professional development; **DHQ** district headquarter hospital; **CEmOC** emergency obstetric care; **GA** gestational age;; **i.m.** intramuscularly; **JPMC** Jinnah Postgraduate Medical College; **p.o.** orally ; **sBP** systolic blood pressure

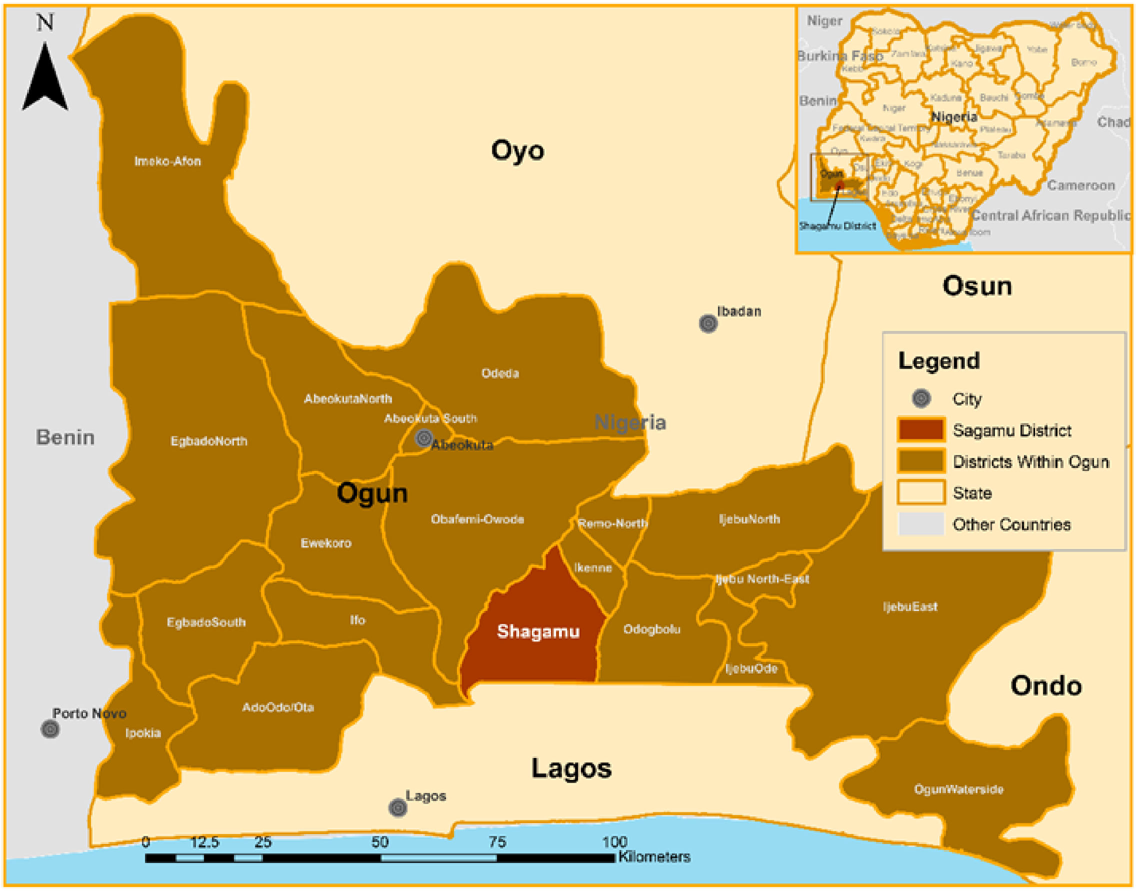

FIGURE 11. Ogun State, Nigeria

Putting the physical location of the CLIP Trial into context

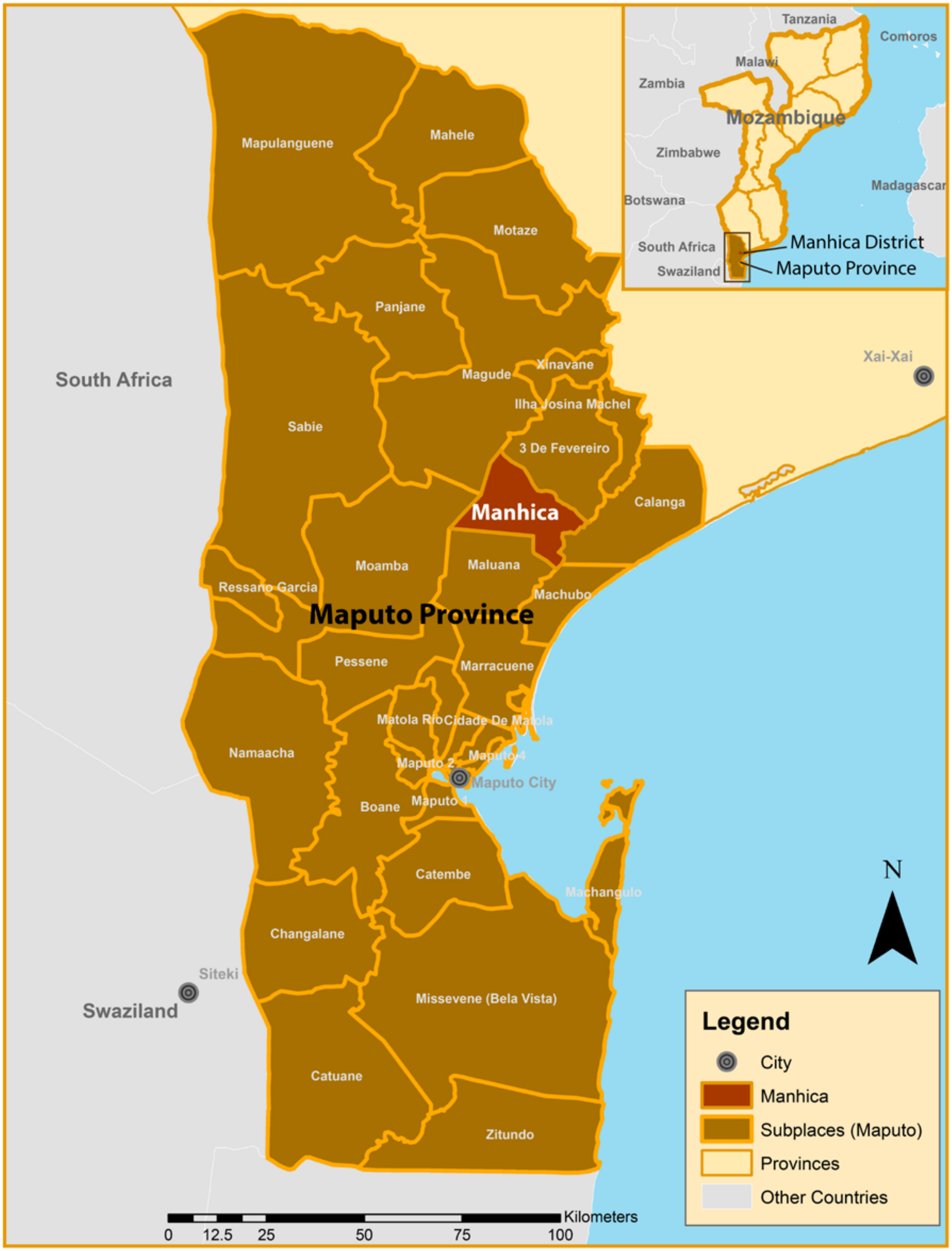

Figure 12 Maputo Province, Mozambique

Putting the physical location of the CLIP Trial into context

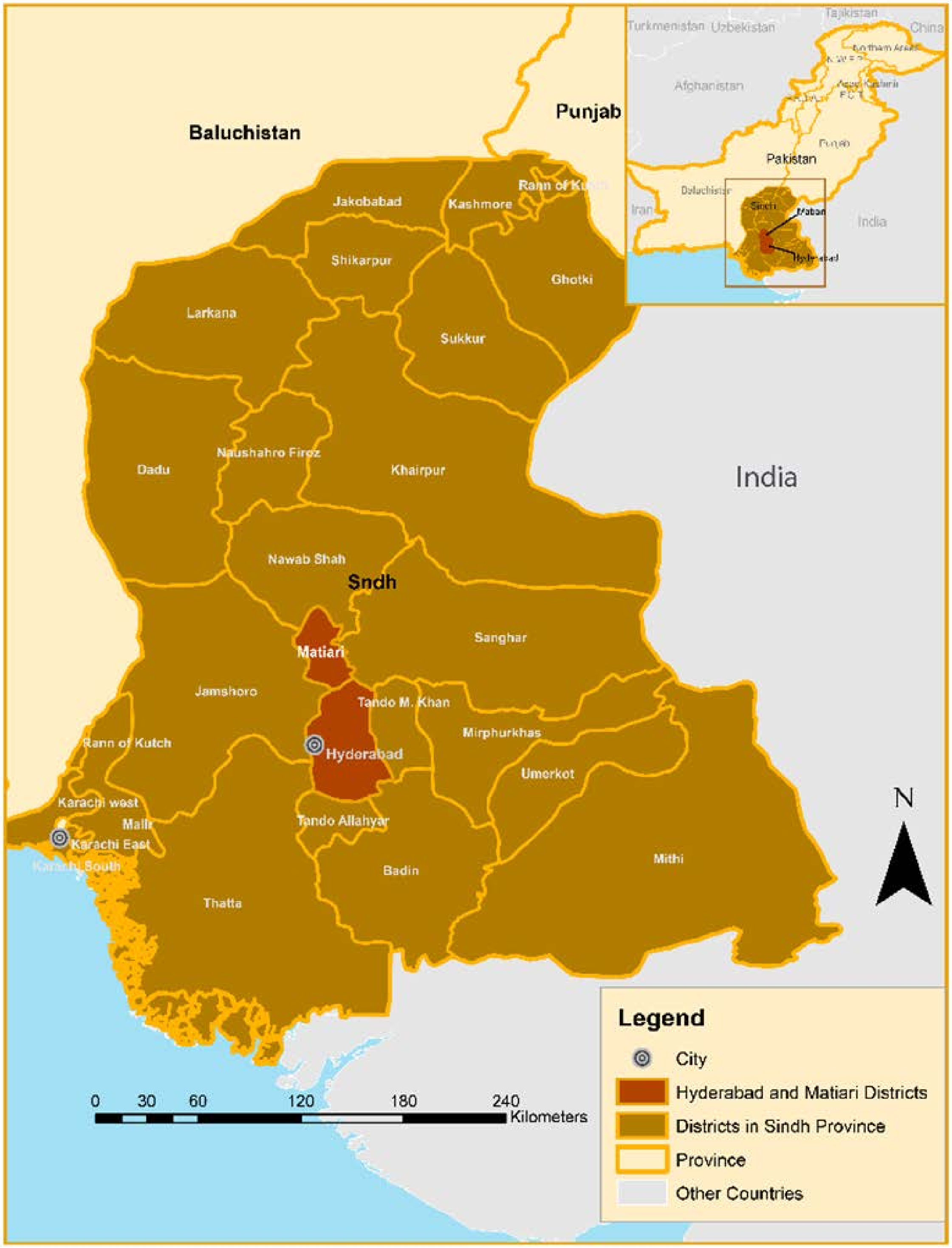

Figure 13. Hyderabad and Matiari Districts, Sindh Province, Pakistan

Putting the physical location of the CLIP Trial into context

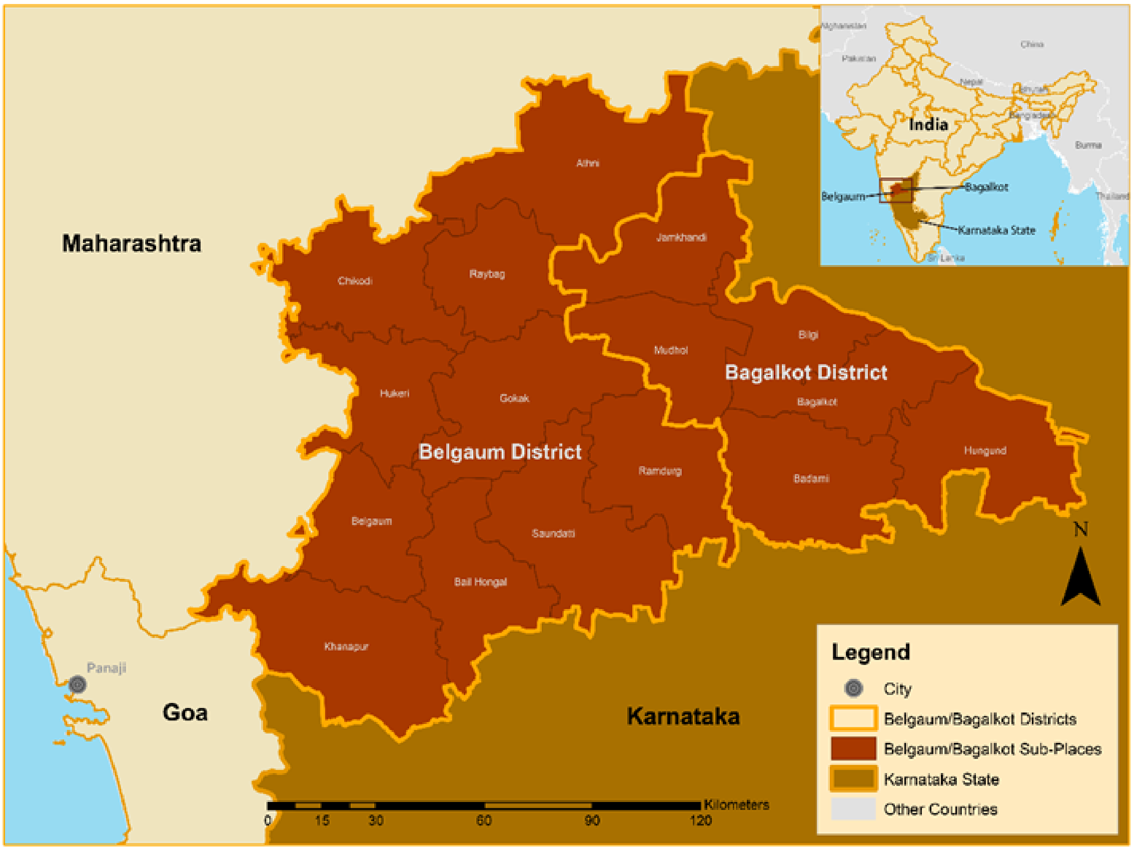

Figure 14. Belgaum and Bagalkot Districts, Karnataka State, India

Putting the physical location of the CLIP Trial into context

Appendix A – Summary of site visit and initial Feasibility Study findings

Partnerships

The following stakeholders have been engaged and have provided support for the trial:

Nigeria – Academic

Centre for Research in Reproductive Health, Sagamu, Ogun State, Nigeria.

Nigeria - Regional & national

Commissioner of Health, Ogun State; Ogun State Ministry of Health; Olabisi Onabanjo University Teaching Hospital; Medical Officers of Health, Ogun State LGAs; Society of Gynaecology and Obstetrics of Nigeria (SOGON); Association of Maternal and Fetal Medicine Specialists of Nigeria; Yewa South Local Government (Ilaro State Hospital, Leysley PHC, Igbo-gidi PHC); Remo North Local Government (General Hospital, Isara, Ode-Remo PHC, Ipara PHC, Idi-Aba Health Post; Sagamu Local Government (Ogijo PHC, Sagamu LGA) and Imeko Local Government (General Hospital, Imeko, Imeko PHC).

Mozambique - Academic

Manhiça Health Research Centre, Maputo, Mozambique

Eduardo Mondlane University, Maputo, Mozambique

Mozambique - Regional & National

Mozambique Ministry of Health; Maputo Municipal Council (responsible for health); Mozambique Association of Obstetricians and Gynecologists (AMOG); Eduardo Mondlane University, Maputo; Maputo Central Hospital; Clinton Foundation.

Pakistan - Academic

Aga Khan University and Jinnah Postgraduate Medical College, Karachi, Pakistan

Pakistan - Regional & national

Sindh Ministry of Health; National Lady Health Worker Programme; Society of Obstetricians and Gynaecologists of Pakistan; Isra University Hospital; Liaquat University Medical Health Sciences Hospital; Aga Khan Maternal & Child Care Centre, Hyderabad; Taluka Headquarter Hospital; Aga Khan University Hospital, Karachi; Jinnah Postgraduate Medical College Hospital, Karachi.

India - Academic

Jawaharlal Nehru Medical College, Karnataka Lingayat Education University, Belgaum, India.

India - Regional & national

District Health Officers, Belgaum and Bagalkot; Chief Executive Officer, Zilla Panchayat (local self government), Belgaum and Bagalkot; Programme Director, RCH, Ministry of Health and Family Welfare, Government of Karnataka, Bangalore; Reproductive Health and Nutrition Division, Indian Council of Medical Research, New Delhi; Representatives of Federation of Obstetric and Gynaecological Societies of India (FOGSI) and Indian Academy of Paediatrics (IAP).

World Health Organization Department of Maternal, Newborn, Child and Adolescent Health (MNCAH)

The chair of the PRE-EMPT objective 5 (knowledge translation) group is Dr Matthews Mathai, from this WHO department. The MNCAH group was instrumental in co-ordinating the development of the 2011 WHO pre-eclampsia guidelines, and will be instrumental in updating the guideline to reflect the findings of

the CLIP trial, and in promulgating the results of the CLIP trial to key decision makers in LMICs. Dr Lale Say is a member of the advisory group advising on maternal mortality and morbidity methods for CLIP.

UNDP/UNFPA/WHO/World Bank Special Programme of Research, Development and Research Training in Human Reproduction (RHR)

The WHO RHR group have been important partners in the PIERS research agenda (Mario Merialdi and Mariana Widmer), as well as for objectives 1 (Ana Pilar Bertràn) and 5 (Metin Gülmezoglu) of the PRE-EMPT project.

Royal College of Obstetricians and Gynaecologists (RCOG)

We have developed a partnership with the RCOG (London) International Office (Senior Vice-President Professor James Walker). An element of the RCOG's international mandate is to strengthen the capacity of inpatient facilities in LMICs in terms of the use of evidence-based care. We will co-ordinate our activities in Nigeria, Mozambique, Pakistan, and India so that the RCOG will support public and private facility capacity building in the referral centres in Nigeria (Sagamu), Mozambique (Manihça and Maputo), Pakistan (Matiari, Hyderabad and Karachi), and India (Belgaum and Bagalkot). This commitment of time, energy and expertise will ensure that women in the study clusters will receive timely and effective care in the centres to which they are referred.

Society of Obstetricians and Gynaecologists of Canada (SOGC)

We have developed a partnership with the SOGC International ALARM Course team. An element of the SOGC's international mandate is to strengthen the capacity of inpatient facilities in LMICs in terms of the use of evidence-based care. We will co-ordinate our activities in Nigeria, Mozambique, Pakistan, and India so that the SOGC will support public and private facility capacity building in the referral centres in Nigeria (Sagamu), Mozambique (Manihça and Maputo), Pakistan (Matiari, Hyderabad and Karachi), and India (Belgaum and Bagalkot). This commitment of time, energy and expertise will ensure that women in the study clusters will receive timely and effective care in the centres to which they are referred.

USAID/MCHIP (Maternal & Child Health Integrated Program)

We have developed a partnership with MCHIP, particulary through Jeffrey Smith (PRE-EMPT TAG member). MCHIP is the USAID Bureau for Global Health's flagship programme focussed on maternal, neonatal and child mortality reduction and acceleration of progress towards MDGs 4 and 5. An element of MCHIP's mandate is to strengthen the capacity of inpatient facilities in LMICs in terms of the use of evidence-based care. We will co-ordinate our activities in Nigeria, Mozambique, Pakistan, and India so that the MCHIP will support public and private facility capacity building in the referral centres in Nigeria (Sagamu), Mozambique (Manihça and Maputo), Pakistan (Matiari, Hyderabad and Karachi), and India (Belgaum and Bagalkot). This commitment of time, energy and expertise will ensure that women in the study clusters will receive timely and effective care in the centres to which they are referred.

Current practice - the provision of maternity care and pre-eclampsia interventions

Nigeria

Antenatal care is delivered at the primary health centre level. Most women attend antenatal care every 3-4 weeks up to 28 weeks gestation, every 2 weeks till 36weeks gestation and weekly thereafter. A typical antenatal visit does include blood pressure measurement and proteinuria testing by indication. Few clinics have a laboratory or ultrasound capacity. Much of obstetric services are paid out of pocket. Few PHCs have ambulance services for referral of complex cases.

Care is accessed through both public and private facilities throughout OGUN State with an estimated 40% of women accessing care through private institutions and 60% attending public facilities.

Community Health Extension Worker (CHEW) training & current scope of practice

Community Health Extension Workers (CHEW) are responsible for delivery of primary health care services in Nigeria. They work both in the community and clinic settings; however due to workload restraints CHEWs often remain stationed predominantly at the PHC. The training and regulation of practice is under the jurisdiction of Community Health Practitioners' Registration Board of Nigeria. The CHEW program includes certification in community health, practical exercises and examinations, supervised clinical experience and supervised community experience. The areas of focus for CHEW training relevant to CLIP are: English training, computer education, medical sociology, reproductive health, clinical skills, and referral system and outreach services. The current CHEW curriculum does not include specific training for management or diagnosis of pre-elcampsia/eclampsia or any other pregnancy complication.

Health Assistants (HA's) training & current scope of practice

The HA's are admitted into the health assistant training program with a secondary school certificate (high school qualification) either at registered private or government training institutions. They work almost entirely in private institutions after completion of their training. They undergo training for three years at these training institutions and assist the trained nurse midwife with care of patients and pregnant women in the community. They are trained to take vital signs, collect preliminary clinical history of symptoms and signs from patient and take pregnant history from patronizing pregnant women at private clinics, where they exist. At the private institutions, Health Attendants are trained to give intramuscular drugs/injections, take normal deliveries and they do give injections when prescribed by doctors in the private clinics.

Mozambique

The Mozambique health system is largely supported by foreign aid [56]. There are significant human resource shortages which require task shifting for obstetric care services. Countrywide, there is a low number of a available skilled birth attendants. Access to primary health care facilities, where antenatal care is delivered, is also low [56]. The first antenatal visit is typically delayed until the 2nd or 3rd trimester, with 84% coverage for at least one antenatal visit during pregnancy [57]. As a result of these constraints the majority of births are rural many without skilled attendants.

Magnesium sulphate has long been used in Mozambique for the management of pre-eclampsia and eclampsia, however, its use has been primarily at central hospitals and alternative anti-seizure medications have been used in the periphery [58].

Agente Communitarios de Saude (ACS) training & current scope of practice

Agente Communitarios de Saude (or basic multi-task health care helpers) help to promote health in their communities; constituting the link between them and the national health system. ACSs are recruited from within the community and receive six months' training to provide basic preventive (community health education) and curative community based-care for diarrhoea, pneumonia and malaria, focussing on the widespread use of diagnostics and appropriate treatment. The ACSs allow isolated populations to have access to basic health care and essential drugs for high burden diseases like malaria, and act as an entry point for referral to higher level health services, providing basic first aid before transfer where needed.

India

The Government of India has recently committed to the National Rural Health Mission. This initiative aims to obtain universal skilled birth attendance across the country. The National Rural Health Mission highlights the role of ANMs in providing antenatal care, timely referrals, and provision of treatment in emergency obstetric cases. Health care workers providing obstetric care at the community level are ANMs,

ASHAs and Anganwadi Workers. Each antenatal visit should include: a medical history, physical exam, abdominal exam, laboratory investigations, clinical interventions (IFA supplement, TT injection, malaria), and health promotion counselling. A minimum of four antenatal visits for all pregnancies is encouraged: before 12 weeks, 12-26 weeks, 28-34 weeks and 36 weeks-term.

Facilities are generally equipped to manage cases of pre-eclampsia and eclampsia. Due to concerns regarding safety and availability diazepam is frequently used in place of $MgSO_4$.

Auxiliary Nurse Midwife (ANM) training & current scope of practice

Training for Assistant Nurse Midwives includes a wide range of health competencies: understanding of holistic health/social determinants of health, ability to mobilize communities, provide emergency care, treatment of minor ailments, midwifery services and referral, basic neonatal care and referral, child services, guide and train TBAs and AWWs, health counselling, participate in National programmes, collaborate with community organizations, manage health care settings. Their midwifery services include care during pregnancy, intrapartum and postpartum.

They are expected to measure blood pressure at every visit and test for albumin in the cases of high blood pressure. If a woman is identified with high blood pressure the ANM is responsible to referral to a 24 hour PHC to initiate antihypertensive therapy. ANMs also follow-up and advise of the warning symptoms. In cases of eclampsia the ANM should ensure safety, give $MgSO_4$ (10g i.m.) and refer to a facility within two hours.

Accredited Social Health Activisit (ASHA) training & current scope of practice

ASHAs are local women trained to act as health educators and promoters in their communities. They receive a total period of 28 days' training in five episodes. However, this core training is supplemented by ongoing CPD activity in parallel with the development of necessary skills and expertise through on the job training. After a period of 6 months of functioning in her village, she is then sensitised to issues related to HIV, AIDS, and STIs (prevention and referral), as well as trained on newborn care.

Their tasks include motivating women to have a facility birth, bringing children to immunisation clinics, encouraging family planning, treating basic illness and injury with first aid, keeping demographic records, and improving village sanitation. In addition, ASHAs are central to communication between the health care system and rural populations.

Pakistan

Pakistan's public health system is centralized under the Federal Government and provincial Health Ministries. The public health sector employs LHWs and Lady Health Visitors (LHV) in rural sectors and nurses and doctors in health facilities and hospitals.[1] According to the 2008 Demographic and Health Survey: Pakistan, less than 30% of women receive four antenatal visits during pregnancy. In many rural settings, staffing levels are inadequate and referral systems function poorly. Almost 70% of births take place at home, usually attended by a Dai (traditional birth attendant [TBA]) [59]. Rural and urban public facilities provide free consultation for obstetric care, whereas consultation charges in private facilities range from $o.5-3USD in rural areas and $4-16USD in urban areas. Some public and private facilities provide ambulance services, additional ambulance services are provided by non-profit organizations in some regions. The majority of health facilities in Hydrabad and Matiari do not have protocols/guidelines for managing pre-eclampsia and eclampsia. There is awareness of $MgSO_4$ for the management of pre-eclampsia and eclampsia however, many continue to use diazepam.

Lady Health Worker (LHW) training & current scope of practice

The Lady Health Worker Programme commenced in 1994 and currently employs 96000 LHWs as part of the federal healthcare system. Each LHW has to cover a population of around 1000-1500 individuals. This

existing number of LHWs is insufficient as WHO suggests the requirement of 150,000 LHW's to cater to the country's healthcare needs.[9] The Government of Pakistan is the major contributor to the funds for this program, external sources only contribute around 11% of the total amount.[9] The role of LHWs is pivotal in Pakistan's healthcare system. They have 15 months of training in MCH [2]. The LHW role in antenatal care includes: health promotion, hospital referrals, and basic neonatal care. Currently LHW is not responsible for immediate management of eclampsia /pre-eclampsia and refers such patients. LHW don't carry any antihypertensive medicine for pregnant woman, and don't have BP apparatus, their role was found to be limited 'to advice to seek care from nearest referral facility' in case if a pregnant woman complained about severe headache with dizziness. An external evaluation of the LHW programme concluded that it was effective in delivering family planning services and immunization services, in the management of diarrhoea, and, most recently through a cRCT, reducing the burden of stillbirth in Sindh Province [9;12].

Appendix B – relevant systematic reviews

Antihypertensives for severe hypertension in pre-eclampsia and the other HDP

Short-acting parenteral agents, such as i.v. hydralazine and labetalol, have been most widely studied in RCTs, although systematic reviews have failed to reveal clear differences between agents [60;61]. We undertook a comprehensive search for RCTs of oral antihypertensive therapy to assess the effectiveness for treatment of severe pregnancy/postpartum hypertension. Also, we reviewed additional relevant RCTs of oral antihypertensive therapy for severe hypertension outside pregnancy to supplement effectiveness data in pregnancy (Firoz, submitted).

In pregnancy and postpartum, we identified 15 trials in pregnancy (914 women), one of which was a postpartum trial (38 women). Trials were generally small with a median of 50 women (range 20 to150) and fair in quality. There was a wide range in the type of HDP at inclusion and gestational age at enrolment. When specified, the BP treatment goal was usually a dBP<100-110mmHg and the success of treatment was evaluated over a short time frame ranging from 20-120 min. Here we summarise the analyses that are relevant to CLIP.

Nifedipine

Twelve RCTs in pregnancy compared oral/SL nifedipine capsules (8-10mg, 12 trials, 724 women) with another agent. Most compared nifedipine with i.v. hydralazine (5-20mg, 7 trials, 350 women) or i.v. labetalol (20mg, 2 trials, 100 women). Other trials compared short-acting nifedipine to oral nifedipine 10mg PA tablets (1 trial), oral prazosin 1mg (1 trial)), or i.v./i.m. chlorpromazine 12.5mg (1 trial). The postpartum RCT (38 women) compared SL nifedipine with i.v. hydralazine.

When short acting nifedipine was compared with i.v. hydralazine in pregnancy, there was no difference in effectiveness as seen by achievement of target BP [84% (nifedipine) vs. 79% hydralazine; RR1.07 95% CI 0.98, .17; 5 trials, 273 women] or time taken to achieve it [WMD -1.36 hours, 95% CI -6.64, 4.14], and the number of doses needed [51% vs. 55%; RR 0.97 95% CI 0.50, 1.88; 4 trials, 246 women]. There was also no difference in maternal mortality [as no studies reported maternal deaths RD 0.00 95%CI -0.03, 0.03; 3 trials, 96 women] or maternal hypotension [1.6% vs. 0%; RD 0.00 95% -0.02, 0.03; 4 trials, 246 women]. There were no differences in perinatal outcomes including adverse fetal heart rate effects, stillbirth and perinatal/neonatal death.

When short-acting nifedipine was compared with i.v. labetalol (N=2 trials), results were similar and there was no difference seen in achievement of target blood pressure, number of doses needed or the requirement for additional antihypertensive therapy. There was no difference in maternal hypotension, maternal mortality and other adverse effects. Similarly, there were no differences in perinatal outcomes including caesarean section [RR 1.13 95% CI 0.76, 1.96; 1 trial, 50 women], stillbirth¸ and perinatal death [WMD 0.00 95% CI -0.07, 0.07; 1 trial, 50 women].

Nifedipine capsules (10mg orally), compared with nifedipine PA tablets (10mg orally), were associated with more maternal hypotension at 90 minutes [35% vs. 9%; RD 0.26, 95% CI 0.07, 0.46, 1 trial, 64 women]. The absolute rate of hypotension with nifedipine capsules in this trial (35%) was higher than that seen in other nifedipine capsule trials of similar dosage (8-10mg) where the rate of maternal hypotension was 0.8%.

Labetalol and methyldopa

There was a single trial (74 women) that compared oral labetalol 100 QID with oral methyldopa 250 mg QID. There was no difference in achieving target BP [47% vs. 56%; RR 0.85 95% CI 0.54, 1.33], Caesarean section [50% vs. 59%; RR 0.85, 95% CI 0.56, 1.30], or perinatal death [5% vs. 0%; RD 0.05

95% CI -0.03, 0.14]. A three-arm trial compared oral methyldopa with either atenolol (50-200mg) or oral ketanserin (80-120 mg). This trial did not report on effectiveness in lowering BP. Perinatal outcomes did not differ between the groups including perinatal death [WMD -0.04 95% CI -0.10, 0.03; 2 trials, 192 women] and stillbirth [WMD 0.01 95% CI -0.03, 0.05; 2 trials, 192 women]

Other antihypertensive agents

One small trial (36 women) compared SL isosorbide with parenteral magnesium sulphate and found no difference between the groups with respect to additional antihypertensive therapy required but there was a significant difference in the rate of Caesarean section between the two groups [16% vs. 89%; RR 0.19, 95%CI 0.07, 0.53].

In summary, our results are similar to that of previous systematic reviews. The analysis of the evidence is complicated by the small numbers of patients, rare events as outcomes and variations in the drug administration regimens among other factors. We found that oral antihypertensive agents, particularly short-acting nifedipine, are a suitable option for treatment of severe hypertension in pregnancy/postpartum.

Oral antihypertensives in SEVERE NON-pregnancy hypertension

Thirty-four studies (1843 patients) met inclusion criteria. Generally, trials were small with a median of 36 patients (range 7 to 374). The dBP for inclusion in most studies was between 110-120 mmHg. When specified, the BP treatment goal was usually a dBP<100-110mmHg and the success of treatment was evaluated over a short time frame with between-trial variability in the time to reach the target BP ranging from 30 minutes to 24 hours. The quality of included studies was poor as there was an unclear risk of bias for sequence generation, allocation concealment, blinding, selective outcome reporting and incomplete outcome data.

Nifedipine

Eighteen trials (describing 21 comparisons) compared oral/SL (short-acting) nifedipine with either another antihypertensive or other doses/formulations of nifedipine. Compared with captopril, oral/SL nifedipine was associated with similar effectiveness in lowering BP: achievement of target BP at 60 minutes [88% (nifedipine) vs. 76% captopril; RR 1. 09 95% CI 0.87, 1.37; N=4 trials, 174 patients], number of doses of antihypertensive required [56% vs. 64%; RR 0.88 95% CI 0.56, 1.38; N=1 trial, 50 patients], or the need for additional antihypertensive therapy [50% vs. 70%; RR 0.71 95%CI 0.34, 1.50; N=1 trials, 20 patients]. The absolute rates of effectiveness were similar to that in pregnancy, but there was significant heterogeneity for achievement of blood pressure.

Only one trial evaluated hypotension and found that there were no episodes in either group. Also, there were no differences seen for other adverse effects including headache [4% vs. 10%; RR 0.86, 95% CI 0.16, 4.71; 4 trials, 145 patients] and cardiac arrhythmia [13% vs. 0%; RR 8.73, 95% CI 0.49, 155.62; 1 trial, 63 patients]. Nifedipine appeared to be associated with more flushing [13% vs. 0%; RR 6.75, 95% CI 1.26, 36.10; 3 trials, 133 patients].

In all other subgroups, nifedipine po/SL was compared with another drug or another dose/formulation of nifedipine in only one or two small trials that enrolled a median of 40 patients. No between-group differences in outcomes were seen.

Labetalol

Four trials (5 comparisons, 568 patients) compared oral labetalol to either: two other doses of oral labetalol (i.e., 100 mg vs. 200 mg vs. 300 mg/d)(66), either amlodipine or perindopril, clonidine, or nifedipine (as discussed above). There were no between-group differences in achievement of target BP or hypotension in any of these comparisons. There was no difference in achievement of BP when 200 mg of oral labetalol was

compared to 100 mg [58% vs. 75%; RR 0.78 95% CI 0.44, 1.39; 1 trial, 24 patients] and 300 mg [58% vs. 67%; RR 0.78 95% CI 0.44, 1.63; 1 trial, 24 patients] with no hypotension observed at either 100 mg, 200 mg and 300 mg doses.

Methyldopa

One trial (3 comparisons) compared oral methyldopa 500 mg with either oral captopril 50 mg, indapamide 2.5 mg, or placebo. Pre-treatment MAP in the methyldopa arm was 127.7 ± 6 mmHg and two hours after treatment, the MAP reduced to 119 ± 4 mmHg. This was comparable to the post-treatment MAP seen in the captopril (125.2 ± 13 mmHg), indapamide (119.3 ± 11 mmHg) and placebo (122.9 ± 12 mmHg) groups.

Other antihypertensive agents

There were one or two trials each of other comparisons including captopril versus place or other agents (methyldopa, indapamide, quinapril, telmisartan, nicardipine, urapidil), oral vs. aerosol isosorbide dinitrate, different doses of isradipine and ketanserin verus placebo or agents. These trials did not report on outcomes related to effectiveness but reported on adverse effects.

Our results are similar to that found in the 2009 systematic review by Souza *et al* with similar challenges included poor quality, small trials and few trials reporting outcomes of interest [62].

Summary

Based on RCTs in pregnancy/postpartum, oral nifedipine (10mg) is a suitable oral agent for treatment of severe hypertension in pregnancy/postpartum, with treatment success rates of at least 84%, low rates of maternal hypotension (upper 95% CI 3%), and similar maternal and perinatal outcomes compared with parenteral hydralazine or labetalol. Although it should be noted that the one 10mg nifedipine capsule vs. 10mg PA tablet trial that did report more hypotension with the capsule formulation found much higher rates of hypotension in *both* arms of the trial (35% in the capsule arm and 9% in the 10mg tablet arm) compared with the six other nifedipine capsule trials of similar dosage (<1%) [63]; also, that hypotension was not necessarily associated with adverse clinical effects.

The few, small comparative trials of other antihypertensive agents in pregnancy/postpartum preclude any firm conclusions. However, the limited data suggest that oral labetalol and methyldopa may be effective in approximately 50% of pregnant women, and each may be a reasonable alternative to nifedipine. Caution should be exercised if considering use of oral prazosin given its association with more Caesarean deliveries and, possibly, stillbirth.

The data from trials outside pregnancy lend further support to the effectiveness of short-acting nifedipine for treatment of severe hypertension (at least 88% success) without significant hypotension. Similar rates of success were also seen with SL captopril (76% success) with which nifedipine was most frequently compared. These data would suggest that oral captopril, which is acceptable for use in breastfeeding, should be considered for use postpartum in the absence of renal impairment.

To our knowledge, this is the first systematic review to specifically examine oral antihypertensive therapy for severe hypertension in pregnancy and postpartum. However, there are other meta-analyses of trials of short-acting parenteral agent or oral nifedipine in pregnancy/postpartum, and the results of the oral nifedipine vs. parenteral hydralazine subgroup are consistent with our analysis.

In the systematic review of oral antihypertensive therapy for severe hypertension outside pregnancy, captopril was associated with fewer minor side-effects than nifedipine [i.e., flushing (RR 0.22 95% CI 0.02, 0.72) and headache (RR 0.34 95% CI 0.13, 0,92)]; the difference in results may be explained by Souza *et al.*'s inclusion of several Spanish-language papers as well as a study (that we excluded) of several patients with phaeochromocytoma.

Our review presents reasonable options for oral antihypertensive therapy. Options are key as there may be contraindications to use of a given drug (or women may already be on high doses of an oral agent when they present with severe hypertension). For example, there are published concerns about heightened cardiovascular morbidity/mortality associated with use of short-acting nifedipine outside pregnancy, and neuromuscular blockade with contemporaneous use of magnesium sulphate and nifepidine in pregnancy (although the risk was estimated to be <1% in a controlled study that incorporated data from RCTs [32]). The usefulness of beta-blockers may be limited in areas where reactive airways disease is prevalent and air quality is poor (such as in Pakistan).

$MgSO_4$ for the prevention and treatment of eclampsia

Prevention of eclampsia

A Cochrane systematic review of 15 RCTs investigated the relative effects of $MgSO_4$ and other anticonvulsants when used for prevention of eclampsia [3]. Notable comparisons in this review were between $MgSO_4$ and placebo or no anticonvulsants (six trials, 11, 444 women); phenytoin (four trials, 2345 women); diazepam (two trials, 66 women); and nimodipine (one trial, 1750 women). One small trial (36 women) compared $MgSO_4$ with isosorbide and another trial (33 women) compared magnesium chloride with methyldopa.

$MgSO_4$ versus placebo or no anticonvulsant Six RCTs (11,444 women) including the large multicentre RCT [64] that involved 10,141 participants provided the evidence related to this comparison. About half of the women recruited into the Magpie trial had the maintenance regimen for $MgSO_4$ through the intravenous route (1g/hr) and the other half through the intramuscular route. Maintenance was strictly by intravenous route for four trials and intramuscular for one trial. For most trials, clinical monitoring for potential adverse effects were reported and none of the six trials reported using serum monitoring of $MgSO_4$.

When compared with placebo or no anticonvulsant, $MgSO_4$ was associated with statistically and clinically significant reduction in the risk of eclampsia by almost 60% (six trials, 11,444 women; RR 0.41 [0.29, 0.58]). This effect was consistent for women who were antepartum at trial entry (six trials, 10,109 women; RR 0.40 [0.27, 0.57]) but non significant for those who were postpartum at trial entry (one trial, 1335, RR 0.54 [0.16, 1.80]). The effect was also consistent and more pronounced among women who were ≥34 weeks' pregnant (two trials, 6498 women; RR 0.37 [0.24, 0.59]) and those who had received no anticonvulsants prior to trial entry (three trials,10,086 women; RR 0.33 [0.22, 0.48]). It was consistent regardless of the route of administration for $MgSO_4$ maintenance.

No statistically significant differences were observed between $MgSO_4$ and placebo regarding the risks of maternal death, any serious maternal morbidity, respiratory arrest and toxicity as shown by respiratory depression and absent tendon reflexes and calcium gluconate administration. Any reported side effects were significantly more common among women treated with $MgSO_4$ rather than placebo (one trial, 9992 women; RR 5.26 [4.59, 6.03]). For the baby, no clear difference were observed in the risks of stillbirth or neonatal death, admission to NICU and Apgar score <7 at 5min.

$MgSO_4$ versus phenytoin $MgSO_4$ was compared with phenytoin for prevention of eclampsia in four RCTs (2343 women). Compared with phenytoin, $MgSO_4$ significantly reduced the risk of eclampsia (three trials, 2291 women; RR 0.08 [0.01, 0.60]). No statistical differences were observed between the two groups in terms of stillbirth, neonatal death, Apgar score <7 at 5min and admission to NICU.

$MgSO_4$ versus diazepam A small trial involving 66 women compared $MgSO_4$ and diazepam for prevention of eclampsia. The sample size and the events recorded were too small for any reliable conclusions.

$MgSO_4$ versus nimodipine In the one trial comparing $MgSO_4$ with nimodipine (1650 women), there were fewer cases of eclampsia among women allocated $MgSO_4$ (RR 0.33 [0.14, 0.77]).

Treatment of eclampsia

$MgSO_4$ versus diazepam for women with eclampsia A Cochrane systematic review of seven RCTs, involving 1396 women, provided the evidence on the differential effects of $MgSO_4$ when compared with diazepam for the care of women with eclampsia [4]. Most women in the trials had eclampsia either before or after delivery and about half of them received an anticonvulsant before trial entry. All regimens used in the trials for both $MgSO_4$ and diazepam included loading and maintenance dose. $MgSO_4$ faired better than diazepam regarding priority maternal outcomes of death (seven trials; 1396 women; RR 0.59 [0.38, 0.92]) and recurrence of convulsions (seven trials; 1390 women; RR 0.43 [0.33, 0.55]). There were no statistical differences between the two drugs for any serious maternal morbidity or any of its proxies addressed in this comparison. Regarding fetal outcomes, no clear difference was demonstrated between the comparison groups for perinatal death and admission to NICU. $MgSO_4$ was associated with fewer cases of babies born with Apgar scores <7 at 5min (three trials, 643 infants; RR 0.70 [0.54, 0.90]).

Comparison of the two treatment groups according to route of administration of $MgSO_4$ maintenance showed that intramuscular maintenance significantly reduced the risks of maternal respiratory depression (two trials, 120 women; RR 0.30 [0.10, 0.13]) and maternal ventilation (two trials, 120 women; RR 0.20 [0.05, 0.88]) but no statistical difference for maternal cardiac arrest. The two contributing trials had moderate risk of bias, small sample size and few events and resultant imprecise observations.

$MgSO_4$ versus phenytoin for women with eclampsia Evidence related to the effects of $MgSO_4$ compared with phenytoin for care of women with eclampsia came from a Cochrane systematic review of six RCTs, involving a total of 972 women [5]. Most of the women had eclampsia before delivery and had received anticonvulsants prior to trial entry.

Compared with those treated with phenytoin, women treated with $MgSO_4$ were at reduced risk of recurrence of convulsions (six trials, 972 women; RR 0.34 [0.24, 0.49]), admission to intensive care (one trial, 775 women; RR 0.67 [0.50, 0.89]) and need for ventilatory support (two trials, 825 women; RR 0.68 [0.50, 0.91]). There were no statistically significant differences between the two treatment groups for maternal death, any serious maternal morbidity and the reported proxy outcomes for severe maternal morbidity. Babies born to women treated with $MgSO_4$, rather than phenytoin, were less likely to be admitted for NICU (one trial, 518 infants, RR 0.73 [0.58, 0.91]) but no clear differences was observed between the two treatment groups in the risks of perinatal death and Apgar score <7 at 5min.

$MgSO_4$ versus lytic cocktail for women with eclampsia The evidence on the differential effects of $MgSO_4$ compared with 'lytic cocktail' (usually a combination of chlorpromazine, promethazine and pethidine/meperidine) was derived from a Cochrane systematic review of three small trials involving a total of 397 women [6]. Compared with lytic cocktail, $MgSO_4$ was associated with significantly fewer cases of maternal death (three trials, 397 women; RR 0.14 [0.03, 0.59]), recurrent convulsions (three trials, 397 women; RR 0.06 [0.03, 0.12]), coma for >24h (one trial, 108 women; RR 0.04 [0.00, 0.74]) and respiratory depression (two trials, 198 women; RR 0.12 [0.02, 0.91]). No clear

differences were observed for any other proxy outcome for severe maternal morbidity. The risks of stillbirth and neonatal mortality were also similar between the two treatment groups.

Alternative regimens of $MgSO_4$ for treatment of pre-eclampsia and eclampsia Evidence related to the comparative effects of alternative $MgSO_4$ regimens for treatment of preeclampsia and eclampsia came from a Cochrane systematic review of six RCTs involving 866 women [49]. Two of the trials (451 women) compared regimens for eclampsia while the other four (415 women) compared regimens for pre-eclampsia. None of the trials used dosages shown to be effective in large RCTs demonstrating effectiveness of $MgSO_4$.

When loading dose alone was compared with loading dose plus maintenance regimen for women with eclampsia, one trial (401 women) showed no statistical differences in the critical outcomes of recurrent convulsions and maternal death and the proxy outcome for perinatal death, stillbirth. The loading dose employed in this trial was 4g intravenous (i.v.) plus 6g intramuscular (i.m.), while the maintenance was 2.5 g i.m. every 4h for 24h. The trial had very serious limitations in its quality and the observations were generally imprecise. A small trial (50 women) compared low dose (similar to the regimen above) with 'standard' regimen (4 g i.v. + 8 g i.m. as loading dose, then 4 g IM every 4 hours for 24 hours) for women with eclampsia). The only case of recurrent convulsion in the trial was reported among women treated with the low dose regimen thus generating highly imprecise and unreliable data for this critical outcome. No statistically significant difference was observed between the two treatment groups for admission to NICU and proxy outcomes of oliguria and any baby death.

One small trial (17 women) compared i.v. (2g hourly for 24 hours) and i.m. (5g. 4-hourly for 24 hours) maintenance regimens for women with pre-eclampsia. There was no case of eclampsia in either arm of the trials. The trial was too small to yield any reliable conclusions regarding other priority and proxy outcomes reported [$MgSO_4$ toxicity; renal failure; and stillbirth.

Three trials involving 398 women evaluated short versus 24h postpartum regimens for women with mild and severe pre-eclampsia or imminent eclampsia. Two of these trials, accounting for approximately two-thirds of the participants, were at low or no risk of bias while one was at moderate risk of bias. None of the women in both arms of these trials developed any of the critical outcomes addressed: eclampsia (two trials, 394 women); $MgSO_4$ toxicity (one trial, 196 women).

Summary of the trial evidence:

Therefore, we have identified that $MgSO_4$ is the agent of choice for the prevention and treatment of eclampsia – however, it has never been examined as a community-level intervention (either in isolation or as a package of care). In addition, we have identified that there is no clear choice of oral antihypertensive for the management of severe pregnancy hypertension – however, we are completing the relevant trial as part of the PRE-EMPT project in preparation for CLIP.

Appendix C – Community Engagement Mapping

Intervention Cluster (name/identifier)			
Activities related to:	Do current activities exist targeting this objective? Y/N<br>If yes, please describe current activities (what types of activities, for whom, how often, where, and who leads them)	Contact person	Source of information
Pregnancy-related complications			
General Maternal Health			
Household decision-making around pregnancy and childbirth (eg. husband's and mother in-law's permission to go to hospital when necessary)			
Transportation initiatives			
Fundraising, insurance schemes or other initiatives related to reducing the barrier of cost of transport and treatment			
Other activities at the individual, household or community level related to maternal health			

Appendix D – cHCP Working Protocol

THE CLIP (COMMUNITY LEVEL INTERVENTIONS FOR PRE-ECLAMPSIA) CLUSTER RANDOMIZED CONTROLLED TRIAL – cHCP WORKING PROTOCOL

SECTION 1: BACKGROUND

1.1 WHAT IS THE PROBLEM TO BE ADDRESSED?

The hypertensive disorders of pregnancy (HDP) are defined by high blood pressure in pregnancy. Globally, the HDP are the second leading direct cause of maternal death; every year the HDP are responsible for the death of an estimated 75,000 women and over 500,000 babies. Almost all of these deaths occur in Africa and South Asia and are preventable. Maternal deaths relate primarily to delays in triage (ability of care provider and women to identify who is severely ill and requires urgent care), transport (ability to get women to appropriate care when needed) and treatment (ability to provide appropriate treatment when care accessed).

The CLIP trial approach to reducing these delays involves task-shifting the monitoring for complications related to the HDP to community health care providers (cHCPs); a cadre of health workers that is currently available in low-resourced settings. The health workforce shortage is a significant global problem that is preventing many countries from reducing child and maternal mortality associated with HDP. Care of these pregnancy disorders previously relied on doctors and midwives in facilities. Treatment for HDP in facilities involves costly medication for reducing blood pressure and, because delivery is the only way to stop the disorder, it often requires emergency Caesarean section when the severity of the disorder is identified too late. By identifying those women and babies at highest risk of complications through community-level screening and monitoring, transportation and treatment can be targeted to those most in need and timed to allow induction of labour instead of Caesarean delivery. There are two key benefits for poor and vulnerable populations: first, at the individual level women are not suffering the cost and time away from their families for unnecessary referrals or interventions when safe, increased community surveillance would be appropriate; second, at the health systems level, moving monitoring from the facility to the community increases the potential for broad population based screening, and more efficient use of already burdened acute care facilities.

1.2 CLIP TRIAL HYPOTHESIS AND OBJECTIVES

Hypothesis

That implementing community-level evidence-based care will reduce pre-eclampsia-related maternal mortality and major morbidity.

Objectives

To reduce pre-eclampsia-related, and all-cause, maternal and perinatal mortality and major morbidity by 20% in intervention clusters in Ogun (Nigeria), Maputo and Gaza Provinces (Mozambique), Sindh (Pakistan) and in Karnataka (India).

1.3 DOCUMENT PURPOSE AND SCOPE

Purpose

This document provides full descriptions of daily CLIP activities to be performed by cHCPs in all intervention clusters.

Scope

This document covers the following cHCP activities:

- Collecting demographic information on new patients
- Measuring blood pressure
- Measuring SpO_2 (Mozambique and Pakistan only)
- Estimating gestational age using symphysis-fundal height measurement
- Measuring proteinuira
- Assessing symptoms of pre-eclamspia
- Administering treatment for severe hypertension (oral methyldopa)
- Administering treatment to prevent seizures (intramuscular $MgSO_4$)
- Providing recommendations for transport to hospital

SECTION 2: cHCP ANTENATAL VISIT PROTOCOL

The cHCPs will be encouraged to identify pregnant women in their area, will have pregnant women referred to them when identified by the surveillance team during regular surveillance cycles, and will provide CLIP assessment during regular antenatal visits that place women in one of three care trajectories: 1) usual antenatal care 2) non-urgent referral, or 3) urgent referral. The regular CLIP visits will occur: 4-weekly during pregnancy until 28 weeks, fortnightly from 28-35 weeks and weekly thereafter, within 24 hours of birth, and on approximately days 3, 7, and 14 after delivery.

This section provides a description of each component of a CLIP assessment. These steps are meant to be performed in addition to any regular antenatal or postnatal care processes routinely performed by the cHCPs.

NOTE: CLIP visits should only occur after the woman being assessed has registered for the trial and provided informed consent for her participation.

Figure 9 provides a flow-chart outlining the assessment protocol and decision points used to generate recommendations for care.

2. FIRST VISIT PROTOCOL

Overview

Each CLIP assessment visit will first involve an assessment of obvious signs of maternal risk including significant vaginal bleeding, stroke, lack of consciousness and seizures. All visits will also include a component of education by the cHCP regarding warning signs and symptoms of pregnancy.

On the first CLIP visit all women will have their blood pressure and proteinuria measured and, if antenatal, will have an assessment of gestational age performed based on last menstrual period dates and symphysis-fundal height measurement. If the woman is found to be hypertensive (systolic BP≥ 140 mmHg) she will also be asked targeted questions about symptoms as described below.

2.1.1 Step 1: Observe for overt signs of maternal compromise

- **signs of unconsciousness**

cHCP will observe to determine if the woman is unconscious at the time of the visit. If a woman is found to be unconscious her family members should be asked to determine if she had been observed to show signs of seizure or stroke prior to losing consciousness.

> PLEASE NOTE: Any woman found unconscious at the time of the cHCP visit will not be evaluated as described in the remaining protocol. The immediate concern when a cHCP encounters an unconscious woman will be to position the woman on her left side by placing a rolled blanket or hard pillow under her right hip and lumbar area. Once the woman is positioned this way the cHCP should attempt to measure the unconscious woman's blood pressure (see 2.3.5) and treat with $MgSO_4$ if sBP≥ 160mmHg before urgently referring for follow-up at a CEmOC facility. While transport is being arranged the cHCP should ensure the woman is placed in the recovery position on her left side and have nothing placed in the woman's mouth.

- **signs of stroke**

cHCP will visually assess for obvious signs of stroke at the beginning of the visit. Signs of stroke include either paralysis of one side of the body or a report of recent sudden loss of vision.

- **signs of eclampsia**

cHCP will visually assess for obvious signs of eclampsia at the beginning of the visit. Signs of eclampsia are defined as a sudden, violent, uncontrollable contraction of a group of muscles or a subtler sign as a brief loss of consciousness.

- **signs of vaginal bleeding**

The cHCP will visually assess for obvious signs of significant vaginal bleeding at the beginning of the visit defined as antepartum vaginal bleeding with pain before the onset of labour or rupture of membranes

2.1.2 Step 2: Introduction and greeting

The cHCP should introduce her/himself and provide an acceptable introduction to the purpose of the visit, as defined by local ethics board. At this stage the cHCP will use the warning signs and symptoms of pregnancy pictoral aide to describe the intervention and provide information to the woman and her family about signs and symptoms to watch out for. The woman should be encouraged to seek care from the C-HCP or at a primary health centre should they note any of the warning signs or symptoms during the pregnancy.

2.1.3 Step 3: Wash hands

The cHCP should wash their hands before performing any assessments on the woman.

2.1.4 Step 4: Record demographic data

Demographic data will be collected at the first visit only. If the woman has already been registered for the study by the surveillance team, the first step will be to locate this woman based on her surveillance ID in the CLIP-POM tool database. This will ensure new evaluations of the woman being evaluated are added to any existing data collected through CLIP trial activities. If the surveillance ID is unknown or the woman cannot be found, this information along with all demographic data can be manually entered.

1 Name
2 Age of pregnant woman
3 Phone number of pregnant woman or head of household
4 History of previous deliveries

2.1.5 Step 5: Assess maternal status

Determine if the woman is antepartum (has not delivered yet) or postpartum (has delivered a baby recently).

> PLEASE NOTE: Women who are visited for the first time postpartum are ineligible for inclusion in the trial and should be provided with routine postpartum care according to local protocols.

2.1.6 Step 6: Assess fetal status

cHCP asks:

- "Have you noticed fetal movements in the last 12 hours?"

2.1.7 Step 7: Measure proteinuria

1. Provide woman with small cup/bottle for urine sample collection
2. Pregnant woman urinates into a small cup provided, all samples should be "clean catch" and collected in a clean container
3. Immerse the dipstick completely in fresh urine and withdraw immediately, drawing edge along rim of container to remove excess
4. Hold dipstick horizontally before reading

*cHCPs should wear clean gloves when measuring proteinuria; gloves to be discarded when measurement complete

2.1.8 Step 8: Take blood pressure

* Let the woman rest (seated, no talking) for at least 5min before taking the first measurement.

1. Place cuff on either arm supported at heart level – on a table (or arm rest of the chair) with woman sitting with back against a chair, no tight clothing around upper arm, both feet on the floor, cuff 1-2cm above elbow.

**Women must remain still (i.e. no movement or talking) while the measurement is being taken for an accurate reading.

2. Turn on machine and inflate cuff by hand, the cuff will then deflate automatically. If the cuff has not been inflated to the correct pressure, the device will indicate this with a 'beeping' sound - inflate the cuff to 30mmHg higher than the previous inflation pressure. Try to keep the device as still as possible during cuff deflation or alternatively let it rest on the table during deflation.
3. Note measurement

4. Wait 1 minute (during which the woman should remain still i.e. no movement or talking) and repeat - all women will receive two blood pressure measurements; an average of the two readings will be taken.
5. If the second measurement differs significantly (>10mmHg) from the first, a third measurement is required. In this case the second and third measurements will be averaged to determine blood pressure.

***If the result is an error readout, repeat the above process

2.1.9 Step 9: Determine estimated date of delivery

1. Determine if ultrasound is available, if this information is available record it in the space provided.
2. Estimate LMP, if available record.
3. Measure symphysis-fundal height (SFH)
   1. Lie semi-recumbent on a firm surface
   2. Place tape at base of pubic bone
   3. Measure from pubic bone up to the top of the fundus and note measurement

   To be taken at every visit until 24 weeks gestation is identified. This measurement will be taken in all women hypertensive and non-hypertensive.

   The SFH will be used to estimate gestational age if an ultrasound is unavailable.

   **This will only be done at the first antepartum visits

2.1.10 Step 10: Assess warning symptoms of pre-eclampsia (if systolic blood pressure found to be ≥ 140 mmHg)

cHCP asks specifically about each warning symptom for pre-eclampsia:

"Are you experiencing the following?"

a. Headache: the patient has experienced any headache, as specifically stated to the community health care provider
b. Visual disturbances: the patient has experienced visual disturbances (blind spots, blurry vision, scintillations/flashing lights, transient blindness), as specifically stated to the cHCP
c. Chest pain: the patient has experienced chest pain or tightness, as specifically stated to the cHCP
d. Dyspnoea: the patient has experienced dyspnoea (difficult, laboured breathing or shortness of breath), as specifically stated to the cHCP
e. Abdominal pain: the patient has experienced abdominal pain (not described as right upper quadrant/epigastric pain), as specifically stated to the cHCP

2.1.11 Step 11: Measuring Blood Oxygen saturation using the pulse oximeter (Mozambique and Pakistan only)

As with measurement of blood pressure, while measuring the SpO2, women should be seated comfortably and asked to stay still while the device is in use. Movement can impact the validity of the measured result. To measure SpO2 the following steps should be followed:

1. Ensure that the sensor is plugged into the mobile phone correctly and the light within the sensor is on
2. Position the sensor on the woman's finger so that the light and the detector are directly opposite each other with the light passing through the middle of the finger
3. The sensor should fit snugly around the woman's finger so that it does not move around but not too snugly that it is impacting the blood flow through the finger

4. Wait until the waveform on the phone's display becomes consistent in shape and the background colour on the screen turns green, as this indicates good signal quality from the sensor – then press start to begin the measurement.
5. The measurement will take 60 seconds. Progress of the measurement will be indicated by a progress bar on the top part of the mobile phone screen. The progress bar will change between green and red depending on the quality of the signal coming from the finger sensor. If the majority of the measurement had poor signal quality (indicated by red background colour) the cHCP is required to repeat the measurement.
6. Once the measurement is complete at sufficient quality, the result will be displayed as a percentage.
7. Remove the sensor from the woman's finger once the measurement has been completed.
8. Clean the sensor by wiping all surfaces with a 70% isopropyl alcohol wipe.

NOTES

** all recording should take place in the cell phone as well as the woman's personal antenatal card and the cHCP log book

** this visit does not REPLACE an existing protocol for antenatal assessment but should be purely in addition to.

2.2 Subsequent visit protocol

Overview

Each CLIP assessment visit will first involve an assessment of obvious signs of maternal risk including significant vaginal bleeding, stroke, lack of consciousness and seizures. In addition, all visits will include a component of education by the cHCP regarding warning signs and symptoms of pregnancy.
On all subsequent CLIP visits (after the first visit) all women will have their blood pressure measured. If the woman is found to be hypertensive (systolic BP≥ 140 mmHg) she will also have her proteinuria measured and will be asked targeted questions about symptoms as described below.

2.2.1 Step 1: Observe for overt signs of maternal compromise

- **signs of unconsciousness**

cHCP will observe to determine if the woman is unconscious at the time of the visit. If a woman is found to be unconscious her family members should be asked to determine if she had been observed to show signs of seizure or stroke prior to loosing consciousness.

> PLEASE NOTE: Any woman found unconscious at the time of the cHCP visit will not be evaluated as described in the remaining protocol. The immediate concern when a cHCP encounters an unconscious woman will be to position the woman on her left side by placing a rolled blanket or hard pillow under her right hip and lumbar area. Once the woman is positioned this way the cHCP should attempt to measure the unconscious woman's blood pressure (see 2.3.5) and treat with $MgSO4$ if sBP≥ 160mmHg before urgently referring for follow-up at a CEmOC facility. While transport is being arranged the cHCP should ensure the woman is placed in the recovery position on her left side and ensure nothing is placed in the woman's mouth.

- **signs of stroke**

cHCP will visually assess for obvious signs of stroke at the beginning of the visit. Signs of stroke include either paralysis of one side of the body or a report of recent sudden loss of vision.

- **signs of eclampsia**

cHCP will visually assess for obvious signs of eclampsia at the beginning of the visit. Signs of eclampsia are defined as a sudden, violent, uncontrollable contraction of a group of muscles or a subtler sign as a brief loss of consciousness.

- **signs of vaginal bleeding**

cHCP will visually assess for obvious signs of significant vaginal bleeding at the beginning of the visit defined as antepartum vaginal bleeding with pain before the onset of labour or rupture of membranes

2.2.2 Step 2: Introduction and greeting

The ccHCPc should introduce her/himself and provide an acceptable introduction to the purpose of the visit, as defined by local ethics board. At this stage the ccHCPc will use the warning signs and symptoms of pregnancy pictoral aide to describe the intervention and provide information to the woman and her family about signs and symptoms to watch out for. The woman should be encouraged to seek care from the cHCP or at a primary health centre should they note any of the warning signs or symptoms during the pregnancy.

2.2.3 Step 3: Wash hands

The cHCP should wash their hands before performing any assessments on the woman.

2.2.4 Step 4: Assess maternal status

Determine if the woman is antepartum (has not delivered yet) or postpartum (has delivered a baby recently).

2.2.5 Step 5: Assess fetal status (if antenatal)

cHCP asks:

- "Have you noticed fetal movements in the last 12 hours?"

2.2.6 Step 6: Take blood pressure

* Let the woman rest (seated, no talking) for at least 5min before taking the first measurement.

1. Place cuff on either arm supported at heart level – on a table (or arm rest of the chair) with woman sitting with back against a chair, no tight clothing around upper arm, both feet on the floor, cuff 1-2cm above elbow.

**Women must remain still (i.e. no movement or talking) while the measurement is being taken for an accurate reading.

2. Turn on machine and inflate cuff by hand, the cuff will then deflate automatically. If the cuff has not been inflated to the correct pressure, the device will indicate this with a 'beeping' sound - inflate the cuff to 30mmHg higher than the previous inflation pressure. Try to keep the device as still as possible during cuff deflation or alternatively let it rest on the table during deflation.
3. Note measurement
4. Wait 1 minute (during which the woman should remain still i.e. no movement or talking) and repeat - all women will receive two blood pressure measurements; an average of the two readings will be taken.
5. If second measurement differs significantly (>10mmHg) from the first a third measurement is required. In this case the second and third measurements will be averaged to determine blood pressure.

***If the result is error readout, repeat the above process

PLEASE NOTE: If the woman has a systolic blood pressure <140 mmHg the visit is complete at this stage and no other CLIP assessments should be performed.

2.2.7 Step 7: Measure proteinuria (if systolic blood pressure found to be ≥ 140 mmHg)

1. Provide woman with small cup/bottle for urine sample collection
2. Pregnant woman urinates into a small cup provided, all samples should be clear catch and collected in a clean container
3. Immerse the dipstick completely in fresh urine and withdraw immediately, drawing edge along rim of container to remove excess
4. Hold dipstick horizontally before reading

*cHCPs should wear clean gloves when measuring proteinuria, to be discarded

2.2.8 Step 8: Assess warning symptoms of pre-eclampsia (if systolic blood pressure found to be ≥ 140 mmHg)

1. cHCP asks specifically about each warning symptom:
   "Are you experiencing the following?"

a. Headache: the patient has experienced any headache, as specifically stated to the community health care provider
b. Visual disturbances: the patient has experienced visual disturbances (blind spots, blurry vision, scintillations/flashing lights, transient blindness), as specifically stated to the community health care provider
c. Chest pain: the patient has experienced chest pain or tightness, as specifically stated to the community health care provider
d. Dyspnoea: the patient has experienced dyspnoea (difficult, laboured breathing or shortness of breath), as specifically stated to the community health care provider
e. Abdominal pain: the patient has experienced abdominal pain (not described as right upper quadrant/epigastric pain), as specifically stated to the community health care provider

2.2.9 Step 9: Measuring Blood Oxygen saturation using the pulse oximeter (Mozambique and Pakistan only)

As with measurement of blood pressure, while measuring the SpO2, women should be seated comfortably and asked to stay still while the device is in use. Movement can impact the validity of the measured result. To measure SpO2 the following steps should be followed:

1. Ensure that the sensor is plugged into the mobile phone correctly and the light within the sensor is on
2. Position the sensor on the woman's finger so that the light and the detector are directly opposite each other with the light passing through the middle of the finger
3. The sensor should fit snugly around the woman's finger so that it does not move around but not too snugly that it is impacting the blood flow through the finger
4. Wait until the waveform on the phone's display becomes consistent in shape and the background colour on the screen turns green as this indicates good signal quality from the sensor then press start to begin the measurement.
5. The measurement will take 60 seconds. Progress of the measurement will be indicated by a progress bar on the top part of the mobile phone screen. The progress bar will change between green and red depending on the quality of the signal coming from the finger sensor. If the majority of the measurement had poor signal quality (indicated by red background colour) the cHCP is required to repeat the measurement.
6. Once the measurement is complete at sufficient quality, the result will be displayed as a percentage.
7. Remove the sensor from the woman's finger once the measurement has been completed.
8. Clean the sensor by wiping all surfaces with a 70% isopropyl alcohol wipe.

NOTES

** all recording should take place in the cell phone as well as the woman's personal antenatal card and the cHCP log book, if these final two documents are in common use

** this visit does not REPLACE an existing protocol for antenatal assessment but should be purely in addition to.

SECTION 3: cHCP PROTOCOL FOR TREATMENT

As outlined in the CLIP visit flow-chart, several clinical measures will trigger recommendations for treatment and transport to be performed by the cHCP. This section provides instruction on how to administer the oral antihypertensive treatment (methyldopa), intramuscular magnesium sulphate, how to give advice on recommendations for referral to a facility and how to follow-up once these treatment or transport recommendations have been provided.

3.1 METHYLDOPA ADMINISTRATION INSTRUCTIONS

In the CLIP trial, any woman found to have a systolic blood pressure reading of ≥160 mmHg (or diastolic blood pressure ≥110 mmHg in Nigeria only) will be recommended to receive 750 mg of the oral antihypertensive, methyldopa. The following instructions outline how to provide this medication to an eligible woman:

1. Open the oral antihypertensive medication container contained in the CLIP intervention box
2. Check for expiry date and any changes in colour, consistency etc. of the medication.
3. Read the label
4. Explain to the woman the purpose of administration is to reduce her blood pressure, which was found to be very high and puts her and her baby at risk of severe complications in her pregnancy
5. Allow woman to ask any question about drugs.
6. Offer water or juice to help swallow medication.
7. Provide woman with 3 methyldopa tablets of 250mg each
8. Stay with the woman to arrange transport to a recommended facility, if possible .
9. Complete the referral card by selecting all conditions experienced by the woman and medications given. This card should be given to the woman or her accompanying family to be brought to the facility with her.
10. Save the tablet strip in the same box with woman's name, date of administration and the other details

***Pregnant women should not receive oral medication if unconscious

3.2 MAGNESIUM SULPHATE ADMINISTRATION INSTRUCTIONS

In the CLIP trial, all woman who are found to be experiencing signs of recent seizure or stroke, who have a systolic BP ≥160 mmHg (or diastolic blood pressure ≥110 mmHg in Nigeria only) or a miniPIERS predicted probability ≥25% will be recommended to receive two, 5g intramuscular injections of magnesium sulphate, one in each buttocks. The following instructions outline how to administer this drug to an eligible woman:

1. Explain the reason and procedure briefly to the woman or attendant (as appropriate)
2. Wash hands
3. Put on gloves
4. Take the pre-prepared syringe from the medicine box
5. Check for expiry date and any change in colour, consistency etc
6. Attach needle to syringe and ensure needle is securely attached x2
7. Fill syringe with vial contents x2
8. Clean the injection site
9. Inspect skin surface for bruises, oedema or inflammation
10. Have patient assume a position appropriate for the site selected
11. Administer intra-muscularly in upper and outer quadrant of buttock x2

12. Discard needle and syringe in the designated disposal container
13. Apply pressure to the injection site for 2 minutes
14. Store the used syringe in the box with the name of the woman and reason for administration
15. Stay with the woman asasas long as possible to help arrange transport, accompany the woman if possible.
16. Complete the referral card by selecting all conditions experienced by the woman and medications given. This card should be given to the woman or her accompanying family to be brought to the facility with her.

.

**Normotensive women with vaginal bleeding should not be given $MgSO_4$

3.3 FACILITY REFERRAL INSTRUCTIONS

Women in the CLIP trial will be recommended to go to a facility within 4 hours of a visit for follow-up care if they meet any treatment criteria outlined in sections 3.1 and 3.2 above or if there have been no fetal movements felt within the last 12 hours. In addition, any woman who has a systolic blood pressure measured ≥ 140 mmHg but who does not qualify for referral within 4 hours will be recommended to go to a facility for follow-up care within 24 hours of the visit. The process of recommending referral should include the following steps:

1 Refer to health facility
2 Assist the woman and family to identify the reason for referral by completing the referral card and providing it to them.
3 Have a discussion with the family in order to raise awareness of complications and danger signs and receive permission for transport from the pregnant woman and/or the family decision maker
4 Contact the appropriate/available mode of transport for the transfer
5 Carry out and report the assessment of the woman at the time of referral (blood pressure, proteinuria, seizures, and other signs and symptoms like frontal headache, visual changes, right upper quadrant pain). Enable completion of appropriate documentation and feedback to referring practitioner.
6 Contact referral facility to notify of transfer and current condition of pregnant woman

Appendix E – Microlife BP 3AS1-2 and N3 BP Validation test results and Qualitative Evaluation Plans

1.1 Background

Accurate and regular blood pressure (BP) monitoring is a cost-effective screening tool for the early identification and management of pre-eclampsia in pregnancy. In low- and middle-income countries (LMICS), pre-eclampsia is frequently under-detected not only because attendance to antenatal care is often low, but due to inadequate training in how to take accurate blood pressure measurements, and insufficient, poorly functioning equipment. Firstly, training in the use of a cheap yet technically challenging method, the traditional sphygmomanometer is often lacking amongst health care workers staffing antenatal clinics. Secondly, there is concern over the robustness and safety of these traditional devices (those containing mercury), while the next generation aneroid instruments ideally require six-monthly re-calibration to maintain accuracy. Thirdly, the use of 'manual' BP devices such as these which require manual inflation, ausculatory skills and the observer recording the BP from a calibrated scale is associated with user-bias resulting in inaccurate measurement. This has been attributed to terminal digit preference, wrong Korrotkoff sound interpretation, threshold avoidance, and incorrect deflation speeds. Third generation BP devices which automatically inflate the cuff and provide a digital reading, whilst circumventing some of these issues, also have problems since they depend on a reliable power supply or source of batteries and have poor resistance to shock and fluctuations in temperature. In addition, for all devices, staff may not always be trained in the interpretation of the BP readings, especially in those settings where it is seldom taken due to lack of equipment.

1.2 The Microlife 3AS 1-2 Model

The Microlife 3As 1-2 semi-automatic handheld blood pressure device will be used to measure all blood pressures for participants enrolled in the CLIP cluster randomized control trial. It has been designed and validated specifically for use in developing countries by the CRADLE (Community Blood Pressure Measurement in Rural Africa: Detection of Underlying Pre-Eclampsia, Kings College London, PI: Professor Andrew Shennan). This small handheld device can be used effectively by unskilled personnel after minimal training. We have shown it to be easy to use, reliable and accurate. It can be manufactured at low cost and the manual pump cuff inflation (rather than battery operated) means that battery supply (2 AA alkaline batteries) is only used to power the liquid crystal display showing the BP reading, allowing it to generate 1000-1500 readings on one battery set, with a lifetime use of >20,000 cycles i.e. > decade with daily use (x5). This device was uniformly acceptable to clinic health-care workers in Tanzania (formative research, CRADLE project) and functional after 3 years of extensive use in harsh environments.

The Microlife

1.2.1 Validation of the device

Whilst many BP monitors are validated for general adult populations, few are validated in pregnancy. Common electronic BP monitors have significant difficulties in measuring during pregnancy, and are thought to significantly underestimate pressure by up to 50 mm HG. This means that even when measurements are taken with these devices, it may not be accurate in a pregnant population. The CRADLE research group, in collaboration with the Kimberly Hospital Complex, South Africa, validated the Microlife 3AS1-2 has for use in a pregnant population according to the requirements of the British Hypertension Society

(BHS) protocol in a South African population. A total of 45 pregnant women were recruited. The first 30 women to fulfil the BHS Protocol criteria were selected for the analysis of systolic and diastolic pressures respectively. The Microlife 3AS1-2 device achieved an overall B/A grade in the analysis of 45 pregnant women (Table 4). Overall, the device achieved the AAMI standard for mean difference and standard deviation (<5±8 mmHg) in pregnancy. Mean-against-difference plots are used to illustrate the data graphically for systolic (Figure 1) and diastolic pressures (Figure 2). The poorer performance in the higher blood pressures is consistent with all BP devices and does not preclude its recommendation for use in pre-eclampsia

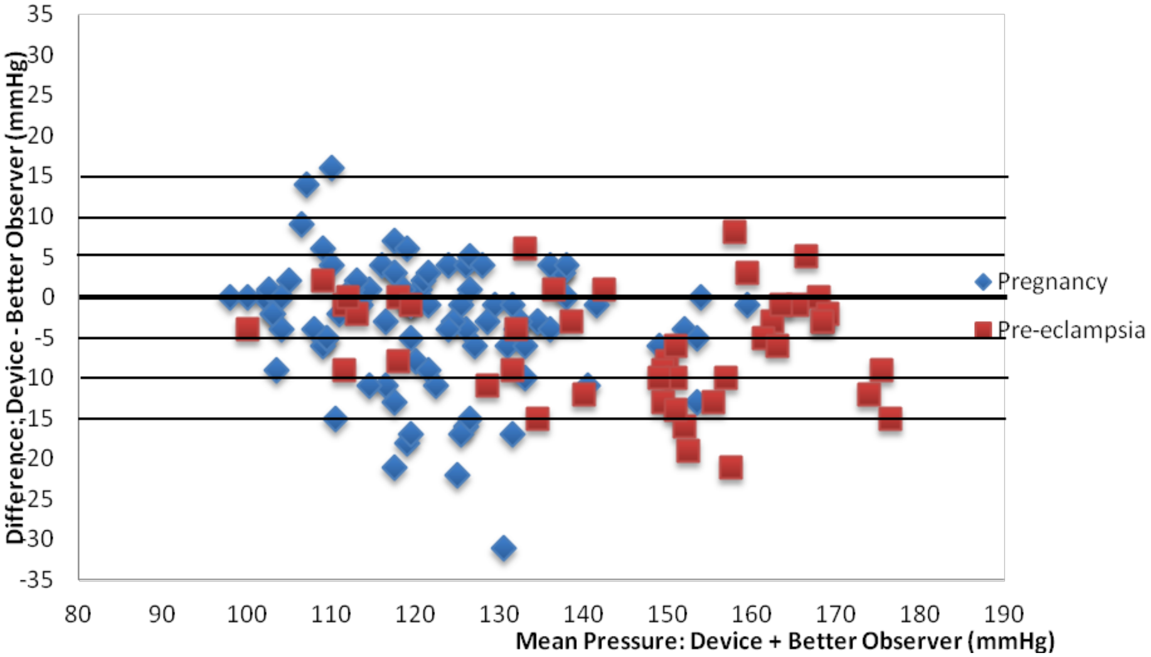

Figure 1. Mean-against-difference plot of the *systolic pressures* of the better observer and the device plotted against their difference in pregnancy and preeclampsia (n=135)

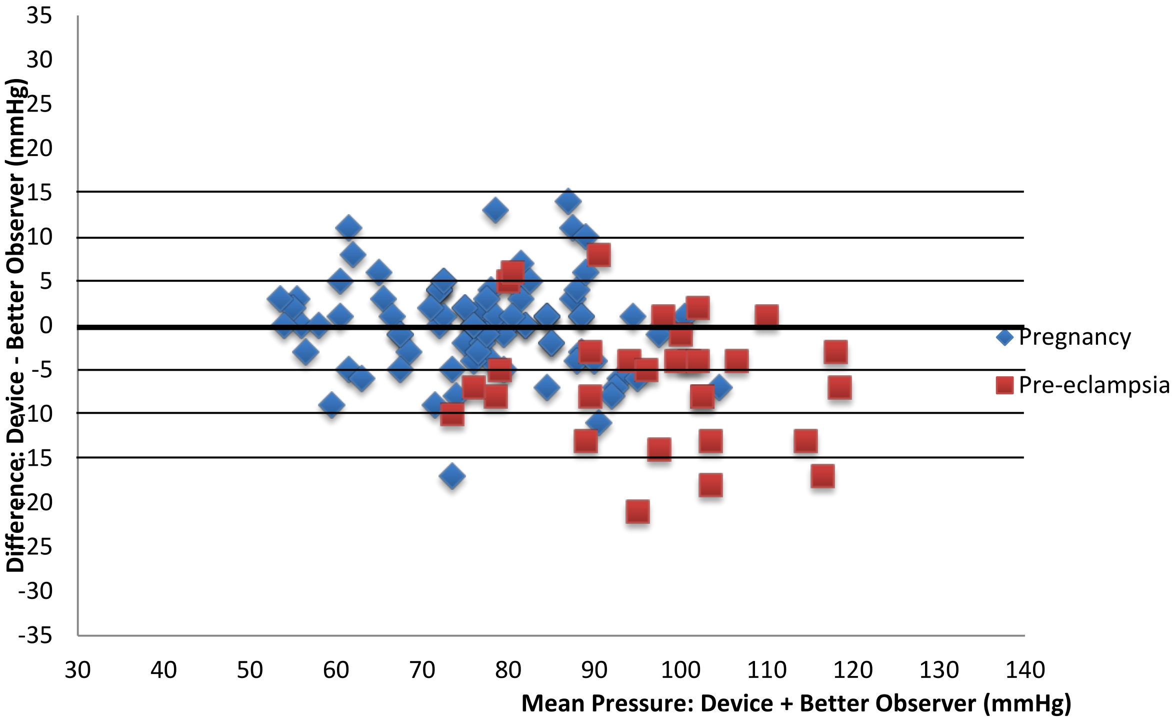

Figure 2. Mean-against-difference plot of the *diastolic pressures* of the better observer and the device plotted against their difference in pregnancy and preeclampsia (N-135)

1.2.2 Microlife N3 BP device

CLIP and CRADLE research groups have completed work with our partners at Microlife to adapt the 3AS1-2device to further suit the environment. The new device (Microsoft CRADLE Vital Signs Alert) will be used in Nigeria only during the definitive trial (Figure 2). The current handheld device has been equipped with a micro-USB adapter (generic) to allow the device to be charged with generic mobile phone charging technology. Rechargeable batteries have been sealed in the device to prevent theft or loss. We have adapted the monitor to include a ‘traffic light’ approach to BP readings whereby readings above particular thresholds are highlighted with red or amber LED displays to alert a provider to act on the result. A traffic light approach will greatly simplify the decision making process for community health care providers regardless of literacy or dialect spoken.
The new Microsoft CRADLE Vital Signs Alert device includes the following indicators for a warning light:

- If the (HR/SYS) ≧ 1.7, the **RED LED** will flash (1Hz) and show the arrow down ↓ flashing (1Hz) => severe shock (index > 1.7)
- If SYS ≧ 160 <u>and</u> (HR/SYS) < 1.7, the **RED LED** will flash (1Hz) and show the arrow up ↑ constantly (no flashing) => no severe shock, but severe hypertension or PE
- If DIA ≧ 110 <u>and</u> (HR/SYS) < 1.7, the **RED LED** will flash (1Hz) and show the arrow up ↑ constantly (no flashing) => no severe shock, but severe hypertension or PE
- If SYS≦159 and (HR/SYS) ≧ 0.9 & <1.7, the **YELLOW LED** will flash (1Hz) and show the arrow down ↓ flashing (1Hz) => high BP, shock (index < 1.7)
- If SYS ≧ 140 & ≦159 and (HR/SYS) < 0.9, the **YELLOW LED** will flash (1Hz) and show arrow up ↑ constantly => high BP, no shock

- If DIA ≧ 90 & ≦109 and (HR/SYS) < 0.9, the **YELLOW LED** will flash (1Hz) and show arrow up ↑ constantly => high BP, no shock
- If SYS <140 and DIA<90 and (HR/SYS) < 0.9, the **GREEN LED** will light constantly and don't show any arrow.

In the CLIP trial, only indicators for a red light will be included as triggers for immediate treatment or referral. This device is currently being validated for use in pregnancy.

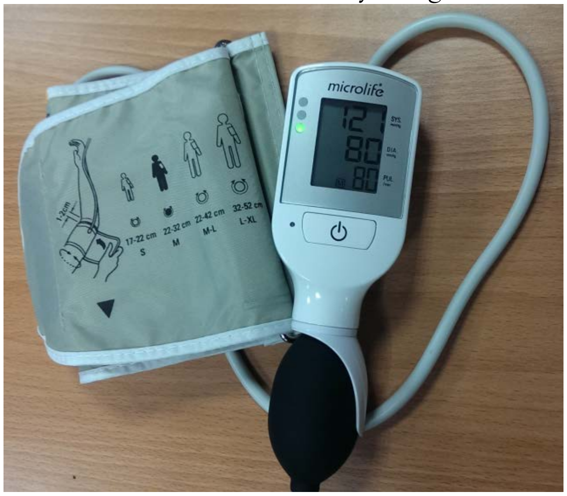

Figure 2: N3 BP device showing a normal (green light) blood pressure and heart rate reading.

CRADLE Qualitative Evaluation during CLIP Study

Community Blood Pressure Monitoring in Rural Africa and Asia: Detection of Underlying Pre-Eclampsia and Shock

Aim of Qualitative Evaluation

To assess the acceptability, usability, feasibility and fidelity of the Microlife 3AS1-2 blood pressure device to community health care providers (cHCPs) in a low-resource setting and the acceptability of the device to women and other community members.

The CRADLE team would like to explore the following:

1. **Do the cHCPs consistently use the device?**
   If not, are there particular reasons why?
2. **Do the cHCPs consistently act on the traffic light early warning system within the device?**
   If not, are there particular circumstances when/reasons why they do not?
3. **How easy is it to use the device?**
   Areas to explore: measuring blood pressure, reading the display, understanding traffic light early warning system, charging the device.
4. **What impact has the device had on the workload of the cHCP and referring HCPs?**
   Areas to explore: traffic light early warning system, referral pathway, impact of false positives/negatives
5. **What are the opinions of the women, family members, village elders, higher-level HCPs and stakeholders?**

Methods of Data Collection

One cluster site from each country (India, Pakistan, Mozambique, Nigeria) will be selected. From each cluster site, eight cHCPs will be selected to participate in the evaluation. Sites used in the pilot trial will be excluded, ensuring the cHCPs have not used the pilot BP device. The selection of cluster sites will be based on convenience and feedback from the site leads. The selection of cHCPs will be based on advice from the site leads.

Data collection will take place at 3 months (after initial training and sufficient practice with device) and 12 months (once users have become experienced with using the device) from the start of the definitive CLIP trial. At both time-points, data will be collected from observation, focus groups and semi-structured face-to-face interviews, over a one-week period.

Observation of cHCPs using the device in a clinic setting (lowest level healthcare facility) will occur at each of the selected cluster sites over a one-day period by a site researcher. **One focus groups discussion** will be run at each of the selected cluster sites and will comprise of eight cHCPs. The focus groups will be led by the site researchers in collaboration with CRADLE.

Per cluster site focus group discussion, four of the eight cHCPs will be selected to participate in **semi-structured face-to-face audio-recorded interviews**. The interviews will be led by the site researcher.
Respondent-driven (snowball) sampling may also take place, depending on feedback from cHCPs. Barriers to appropriate use of the device may exist at levels other than cHCP level. Therefore it may be necessary to perform interviews with women and families, members of the community, key policy-leaders and opinion leaders, and members of the referral unit, to explore these issues.

All participants will be asked to give written informed consent. Incentives, such as transport costs and refreshments, will be provided by CRADLE for all participants.

Appendix F – ECONOMIC EVALUATION ALONGSIDE THE CLIP TRIAL

1. BACKGROUND:

Pre-eclampsia / eclampsia imposes tremendous financial burden on the health care system and the family of the pregnant woman. Economic studies have determined that PE/E are the major reasons for antenatal admission to hospital (20%), and obstetric admissions to intensive care units (25%). Other studies determined that the hospitalization costs for the management of pre-eclampsia and associated complications were on average US$11,208 per woman. Financial costs to the health system aside, studies elsewhere found that the death or serious illness of a mother leads to reduced household income and increased risk of dying for children under 10 years of age.

Economic evaluation in health care can play a pivotal role in informing health decision / policy makers about maximizing health benefit given the set of resource constraints. Cost-effectiveness analysis, in particular, compares the costs (in monetary units) and benefits (in natural units, for example health effects) of interventions to inform whether a particular intervention is worth implementing at a health system or population level. Literature reveals that cost-effectiveness studies have focused only on the diagnostic and clinical management of Pre-eclampsia/eclampsia (PE/E), and less on the community based interventions in the context of high burden countries. This is mainly because of the knowledge gap in the area of comprehensive community based interventions for PE/E, as large scale research trials do not exist. Therefore, we propose to undertake an economic evaluation of the CLIP Trial to determine the cost-effectiveness of the intervention in reducing maternal and perinatal mortality and severe morbidity in the selected countries.

The primary research objectives are to:

a) Determine the costs and benefits of the CLIP Trial interventions to design the cost-effectiveness model;
b) Estimate the incremental cost-effectiveness ratio of the CLIP Trial interventions compared with standard care for reducing maternal and perinatal mortality and major morbidity.

The secondary research objectives are to:

a) Qualitatively identify the resources needed (cost drivers) during the trial implementation to inform design of the model;
b) Explore perceptions of community, health care providers, and policy makers related to the implementation challenges during the trial, and perceived cost-benefits of interventions;
c) Inform health decision / policy makers about the cost-effectiveness of the CLIP Trial for post-trial programmatic scale-up, and sustainability into existing maternal health policies in the CLIP countries.

2. METHODS AND RESEARCH PLAN (PAKISTAN AND MOZAMBIQUE)

2.1 *Research design:*

We propose to use cost-effectiveness analysis (CEA), in conjunction with qualitative analysis, alongside the trial to prospectively evaluate the economic impact of the CLIP Trial. Cost-effectiveness remains the design of choice in economic evaluations when any particular experimental intervention (non-standard) is proposed to have similar or better clinical outcomes at decreased or equal costs compared with the standard intervention. Specifically,

our CEA will be based on a societal perspective, accounting for both costs to families and the health care system. The CLIP Trial is being conducted in four countries, which have different health care delivery systems, healthcare financing, resource allocation interests, diversity of community beliefs surrounding PE/E, care seeking behaviours and treatment preferences. Therefore, the combined approach (CEA and qualitative) will help in designing the cost modeling, and will support interpretation of economic analysis for decision makers who are considering evidence of economic value along with the effectiveness of interventions.

2.2 *Research setting:*

The primary CLIP Trial is recruiting in four countries in two regions i.e., South Asian countries (Pakistan and India); and African countries (Nigeria and Mozambique). Given the budgetary limitations, the economic evaluation of the CLIP Trial will take place in two countries (i.e., Pakistan, and Mozambique) representing each of the two regions in the CLIP Trial. These sites are selected in consultation with lead organizations in each country, as well as through existing academic relationships. The experience of community-based maternal or perinatal health research and research infrastructure in the country was also taken into account when selecting study sites for the economic analysis of the CLIP Trial.

2.3 *Research duration:*

The pilot phase of the CLIP Trial began in January-February 2014 and will be followed by two years of the Definitive CLIP Trial across multiple sites. The cost estimation and modelling will be developed alongside the definitive CLIP Trial, and the country specific cost-effectiveness analysis will be completed within 5 months of the trial end date. The total duration of the proposed research will be 2.5 years (December 2014 – July 2017).

2.4 *Description of variables for designing the cost model*

2.4.1 *Costs to health care system:*

The intention is to provide as complete a picture as possible with respect to resource utilization in the health care system. The costs to the health system will comprise the cost of the CLIP Trial interventions including mHealth technology and infrastructure, blood pressure devices, urine dipsticks, community engagement sessions, and trainings of healthcare providers at community and health facility. In addition, the cost of follow-up household visits and time spent on blood pressure monitoring/ urine dipstick by cHCP in each of the selected sites, such as Agente Polivalente Elementar (community health agents, APE) in Mozambique, and Lady Health Workers (LHW) in Pakistan. Also, the cost of cHCP's additional time and transport costs, when accompanying any identified HDP woman to a referral health facility will be calculated. Health system costs including cost of managing triage, in-patient / out-patient services, diagnostics or drugs for the treatment of HDPs provided by hospital for will also be captured. Moreover, the cost of maternal health programmes (obstetrics clinics/hospitals and outreach services) for obstetric emergencies particularly HDPs, run by NGOs or charity-based organizations working on sites will also be included.

2.4.2 *Cost to family of pregnant women:* All relevant out of pocket expenses for ambulance, hospitalization (physician fees, bed charges, nursing services), drugs and diagnostic workup related to the care for HDPs from the referral health facility. Also, the cost for informal care (care provided by family/friend-who lost wages, or paid help) will be captured, as well as, cost of lost productivity resulting from morbidity or mortality of patients with or without paid jobs. The value of the lost wages will be estimated by using a mean wage rate to missed work time, obtained from country-specific standards.

2.4.3 ***Societal costs*: ** The total societal costs (i.e., combining of costs to the health care system and cost to the family) will be calculated for the intervention and control groups respectively.

2.5 *Methods for collecting resource utilization and unit costs data*

The information about resources utilization and costs will be collected from primary and secondary data sources in the intervention and control clusters. The consistent approach will be followed to collect these data in the intervention and control clusters. (See Table 1)

Table 1: Methods of collecting resource utilization and cost estimations in the intervention and control clusters

Types of data	Intervention Clusters	Control Clusters
**Resource utilization**	The quarterly surveillance tools, as follows:<br>A. Pregnancy registration (Form 1)<br>B. Regular community surveillance (Form 2)<br>C. Health facility patient admission chart review (Form 3)	
**Unit cost-estimation**	Review of hospital budget for maternal health services (costing for bed charges, consultant fees, diagnostic services, nursing services etc.)	
	Review of district level cHCP program budget (costing for cHCP Salaries)	
	Review of site specific CLIP Trial budget (costing for intervention package)	

2.5.1 *The quarterly surveillance tools*: The structured questionnaires will be administered during the quarterly CLIP Trial surveillance in all intervention and control clusters. These questionnaires will be translated into local language, and to be pilot tested before the start of definitive CLIP Trial, are as follows:

- Pregnancy registration questionnaire (Form 1 attached): will focus on resource utilization data, which will be useful to establish baseline resource utilization in the intervention and control clusters.

- Regular community surveillance questionnaire (Form 2 attached): will focus on hospitalization of mother for pregnancy related illnesses, delivery and or newborn (after delivery) over the last 3 months. This information will be useful to quantify resource utilization (frequency of hospital visits, type of health facility), level of health facility, in-patient / out-patient care for delivery or pregnancy complications, length of stay, diagnostic tests, and therapeutic management. Also, information will be collected about mode of transport used, number of accompanying family members, days of missed wages, and information about negative externalities resulting in family-borne costs. Moreover, we will ask the respondents about out-of-pocket cost of illness for each health

resource utilized in the current pregnancy to determine unit cost estimation in the intervention and control clusters.

- Health facility patient admission chart review (Form 3 attached): will focus on the diagnostic and therapeutic interventions utilized by mother or newborn, which will be useful to quantify hospital resources utilization in the intervention and control clusters.

2.5.2 *Review of hospital budget for health services:* The unit cost of hospital services (diagnostics and maternal and newborn intervention) at the referral health centre will be obtained in the intervention and control clusters. The referral health facilities include both public and private health facilities, where the current CLIP Trial participants are being referred. The cost variables will focus laboratory investigations, maternal and newborn interventions as outlined in the form 3).

2.5.3 *Review of district level cHCP program budget:* The salaries of cHCP who are currently involved in the CLIP Trial will be determined through review of district level program budget. In addition, the transport expenses will be calculated for the extra visits of cHCP.

2.5.4 *Review of site specific CLIP Trial budget:* The unit cost estimates for CLIP Trial intervention package include the cost of blood pressure device, urine dipstick, oxygen saturation prop, cost of community engagement sessions, cost of training doctors, nurses, midwives, and community health workers will be determined from the trial budget for each site in the CLIP Trial.

2.6 *Qualitative methods of data collection to inform design of cost modeling*

Focus group (FG) is commonly used method for data collection in qualitative research to gather group opinions. Specifically, the FGs in this study are aimed to better understand the contextual variations of intervention compliance, explore any additional resources utilized, and perceptions of benefits from community perspectives. The community perspectives will be obtained from women identified as hypertensive disorders of pregnancy, husbands / father-in-law of women identified at risk of HDP, cHCP, doctors at referral health facilities, and district health decision/policy makers. (Table 2)

The semi-structured guides have been developed for each focus group (see attached) to explore the community perspectives on the following priori themes:

- Theme I: Costs drivers and health resource utilization as result of the CLIP package of care.
- Theme II: Perceived benefits of the CLIP package of care and task-shifting to community health care providers.
- Theme III: Implementation challenges for the CLIP package of care.
- Theme IV: Strategies for knowledge translation of the CLIP package of care to the wider community.
- Theme V: Strategies for health policy advocacy and program scale-up of the CLIP package of care.

The FG guides will be translated into the local languages and will be pilot tested in a randomly selected intervention clusters before the actual data collection. The digital voice recorders and hand written notes will be used to record the participants' responses during

all FGs. The FG data will be transcribed into the local language, followed by translation into English. All the translations will be confirmed by researchers with back-translation of randomly selected data segments for quality control. We anticipate a total of 10 FGs inclusive of all groups at each site; however, the desired number of FGs will be determined by the data saturation.

Table 2: Number and distribution of FGs

**Number of Focus Groups with target population at each site**					**Total**
Women identified as HDP	Husbands / father –in-law of women identified as HDP	Community health care providers	Doctors at the referral health facilities	District health decision / policy makers	
2	2	2	2	2	10

2.7 *Participants' eligibility (Inclusion and exclusion criteria):*

The pregnant women aged 15 – 49 years recruited in the CLIP Trial in both intervention and control clusters will be eligible to take part in the economic data collection (i.e., Form 1, Form 2 and Form 3) during the quarterly surveillance rounds. For qualitative assessments, only women who were identified at risk to a HDP in the pilot phase of the CLIP Trial, those willing to participate in 45-60 session will be eligible for participating in FGs. Likewise, husbands/father-in-law of those women (identified at risk to a HDP), those willing to participate in 45-60 session will be eligible for FGs. The cHCP handling the CLIP Trial package of intervention, the medical doctors at the referral health facilities where CLIP Trial participants are referred, and district health decision makers those willing to participate in 45-60 session will be eligible to participate in the FGs. Participants will be excluded, those who are not recruited in the primary CLIP Trial and or refuse to take part in the economics data collection procedures. The participants for qualitative assessments will be selected with the help of project staff, who are making home and health facility visits for CLIP Trial surveillance.

2.8 Plan of data analysis*:*

The total cost (quantities of resource utilized multiplied by their unit costs) will be calculated to estimate the cost on health system and family, and it will be denoted as an annual equivalent cost in US dollars rate of 2015. The incremental cost-effectiveness ratio (ICERs) will be calculated for maternal deaths/adverse pregnancy outcomes to compare the CLIP package of interventions and standard care from the societal perspective. The ICER represents additional cost of a more expensive but more effective intervention above that of the less expensive but less effective intervention divided by the difference in effectiveness. This estimate will allow us to compare the cost per unit maternal deaths/adverse pregnancy outcomes averted when switching from standard care to the CLIP package of interventions. Given the uncertainties involved in calculating the costs and trial outcomes, we will use simplistic sensitivity analysis to plot cost-effectiveness ratios. A key literature recommended using simple sensitivity analysis for CEA of package of interventions, because of the complexity of inherent correlations. The confidence region surrounding the cost-effectiveness ratio will be estimated using appropriate statistical methods, including bootstrap and Monte-Carlo analyses.

The qualitative data will be analyzed using QSR NVivo v10 software, and responses will be coded to form similar categories. Data will be interpreted through close communication between site investigators to ensure accuracy. Thematic analyses will be performed to

underscore additional cost drivers for cost modeling, implementation challenges, and perceptions of cost-benefits.

3. METHODS AND RESEARCH PLAN (INDIA)

Background and need for additional data collection in India

The CLIP India Trial recruitment was completed as of October 2016; and data on health resource use was captured as part of Maternal and Newborn Health (MNH) data collection during the trial period. In addition, we have collected a wide range of maternal and newborn health services costs at 60 private health facilities; and six public health facilities. However, the data on patient level costs (i.e., out-of-pocket costs) are merely limited to health facility utilization.

Study objective: We aim to estimate societal costs, inclusive of opportunity costs as a result of HDPs- and other pregnancy- related illnesses and/or hospitalizations in order to complete CEA for the India CLIP trial in line with analysis completed in Pakistan and Mozambique, as described above.

Design/settings: A two-stage cluster rapid survey technique will be undertaken, using a population proportion to size sample in the study settings for definitive CLIP India Trial. This survey design has been widely used in expanded program on immunization (EPI) and endorsed by World Health Organization as 30X7 technique (i.e., 30 clusters; and 7 randomly selected households per cluster).

Data collection: A short-structured questionnaire will be administered to study eligible participants. The woman will be considered eligible, if she delivered (that resulted into a live and/or still birth(s)) during August to October 2016. Key variables will include, missed wage(s) by immediate care provider, missed wages by pregnant women; hired caretaker to do household chores; costs for meal procured during hospitalization, costs of anti-hypertensive medications at home, and transport cost (to and from health facility) related to HDPs and other pregnancy related illnesses-(other than HDPs). (Please refer to survey questionnaire)

A list of eligible households (i.e., pregnancies identified as HDP, and no-HDP in the intervention and control clusters) will be generated from MN-02 database for women who delivered during August to October 2016; and further stratified into women identified with HDPs and without HPDs. Later, we will group these eligible IDs into second-stage cluster(s) in respect to the geographical boundaries (i.e. distance of 1-2 kilometres between second-stage clusters); and randomly draw potential households for survey. A research assistant will administer the survey questionnaire. The total duration of face-to-face interview will be 15-20minutes/household.

Sample size: The CLIP cluster(s) serve as the primary-stage clusters, and Probability Proportion to Size (PPS) technique is applied to calculate number of second-stage clusters. Each cluster represents 7 households; and altogether 210 households will be needed to meet the desired sample size for this survey. Having added ~15% non-response rate to the sample size per cluster, we will have a total of 240 households (average 8 households per cluster). Out of 8 households in a given second-stage cluster, we will have a representative sample of 25% (~2 households/cluster) with HDPs; and 75% (~6 households/cluster) with no-HDPs. (Table 1)

Table 1: Desired sample size for household survey

Cluster #	# of preg. Recruited	# of clusters (PPS)	# of households (8 HH/Cluster)	HDP HH	No HDP HH
**3**	987	2	16	4	12
**6**	1291	3	21	5	16
**5**	1371	3	22	6	17
**8**	1111	2	18	5	14
**1**	1266	3	21	5	15
**12**	918	2	15	4	11
2	1065	2	17	4	13
4	1627	3	26	7	20
7	1045	2	17	4	13
10	1390	3	23	6	17
9	1678	3	27	7	20
11	1029	2	17	4	13
**Total**	**14778**	**30**	**240**	**60**	**180**

Ethics: Informed consent will be taken from participant(s) prior to administering survey questionnaire.

Data analysis:
Data analysis for the Indian CLIP trial CEA will be completed as described for both Mozambique and Pakistan in Section 2.8 above.

APPENDIX G:
A policy analysis of postpartum maternal health policies in Ogun State, Nigeria

1. Background

The WHO defines postpartum period as beginning immediately after childbirth and lasting a period of six weeks[1]. This period is considered to be most critical for newborn and mothers, marking the most deaths for mothers and babies. However, this is the most neglected period for provision of critical care1. In low income countries, an estimated 70% of women do not receive postpartum care[2]. Further it is estimated that an estimated 15% develop potentially life-threatening problems[2]. It is equally important to note that for every maternal death there are a large number of women who suffer illness and suffer long-term consequences of obstetric morbidity. From a purely clinical perspective, postpartum morbid consequences include problems such as postpartum infection, anaemia, perineal tears, urinary tract infection, and depression; others defined in the literature as long-term morbidities/disabilities include incontinence, fistula, pelvic inflammatory disease, genital prolapse, hypertension, haemorrhoids, nerve damage, pituitary failure, anaemia, and infertility[3,4].

Most of these deaths and morbidities are preventable, and a consequence of the poor health and nutritional status of the mother coupled with inadequate care before, during, and after delivery.

In a 2013 WHO systematic analysis of the causes of maternal deaths (in the years 2003–09), 480,000 or 19.7% maternal deaths worldwide occurred postpartum. In sub Saharan Africa alone, 15.2% maternal deaths occurred in the postpartum period, i.e. 42 days following delivery.[4]

The WHO guidelines on the postnatal care for the mother and the newborn1 (2013) provide clinical recommendations for the care of the mother and baby upto six weeks after birth. Recommendations beyond the 24 hour period after birth include that the health care provider enquire about the general well-being and assess the woman's micturition and urinary incontinence, bowel function, healing of any perineal wound, headache, fatigue, back pain, perineal pain and perineal hygiene, breast pain, uterine tenderness and lochia. Further, the guidelines strongly suggest that women should be asked about emotional well-being as part of an assessment of overall well-being two to six weeks after birth.[1]

Nigeria's maternal mortality contributes to 10% of the world's maternal death4,. However, data from the 2005 National HIV/AIDS and Reproductive Health Survey indicates that only 41.2% of the women receive postnatal care4 and data from 2008 Nigeria Demographic and Health Survey in 2008 shows that only 32% of women received postnatal care within 42 days of delivery[5].

2. Main aim

The main aim of the project is to perform policy analysis to evaluate whether the national policies in the state of Ogun, Nigeria respond to the needs of postpartum women i.e. how they address any health condition attributed to and/or aggravated by pregnancy and childbirth that has a negative impact on the woman's wellbeing following childbirth.

3. Objectives

The policy analysis has the following objectives:

1. Review and compare the existing guidelines on postnatal care produced by Nigeria and International knowledge professional organizations-WHO (World Health Organisation), and national organisations such as the SOGON (Society of Obstetricians and Gynecologists of Nigeria)
2. Determine the current/recent postpartum needs in Nigeria by:
3. undertaking a literature review using papers published over the last five years (2009-2015) and using the data from the Demographic Health Survey (DHS) Nigeria to determine "needs" using the following proxies:
   - The burden of ill health postpartum in women in Nigeria by examining the prevalence and mortality rates in the postpartum period using the DHS (Demographic Health Survey) data
   - The service utilisation of postnatal care services in Nigeria
   - The service provision of postnatal care services in Nigeria

4. Identify gaps in national and local policies to address women's needs for optimum postpartum care.
5. Interview stakeholders to triangulate findings from the literature and see what steps would be needed to address these gaps

6. Develop a set of evidence based policy recommendations for postpartum care for women in Ogun, Nigeria.

4. Methods

Review of guidelines

Buse Mays and Walt (2005) define 'policy' as a "broad statement of goals, objectives and means that create the framework for activity".[6] According to the WHO, 'guidelines' are systematically developed evidence-based statements which assist providers, recipients and other stakeholders to make informed decisions about appropriate health interventions (defined broadly to include not only clinical procedures but also public health actions).[7]

In this project, both international guidelines and national policies to be analysed will be sought via literature reviews, agencies such public health institutions, professional societies, and via in-depth interviews and surveys with expert personnel, policy makers, health practitioners and patient-group representatives. The guidelines included would provide some recommendations to address postpartum maternal health.

Identification of policies and guidelines

To identify policies, a comprehensive search for documents (2010-present) will be done via the following sources:

1) peer-reviewed publications by searching the following databases: PUBMED, MEDLINE, EMBASE, CINAHL

2) grey literature search using Google to identify technical meeting reports, workshop reports, and evaluations produced by interest groups including academic institutions, governmental and intergovernmental (eg WHO, UNFPA, SOGON) organisations

3) Policy actors will be identified from the relevant documents, and from the contacts gained via the CLIP Trial and via snowballing. Stakeholders will also be asked to share, where possible, relevant organization/office documental sources of additional information, This information will be added to secondary data sources review.

Determining postpartum needs using DHS data[10]:

Asadi-Lari et al (2003) define “needs” as “the requirement of individuals to enable them to achieve, maintain or restore an acceptable level of social independence or quality of life, as defined by a particular care agency or authority”

In the context of postpartum women this can be considered as what the women need/desire from health care services to improve overall health.

On a macro level, proxies to measure this need can be mortality rates, prevalence rates, socio economic status and service utilisation. Hence the postpartum needs would be determined via:

- The burden of ill health postpartum in women in Nigeria by examining the prevalence and mortality rates in the postpartum period using the DHS (Demographic Health Survey) data
- The service utilisation of postnatal care services in Nigeria
- The service provision of postnatal care services in Nigeria

5. Interviews and online questionnaires

Stakeholders will be asked to share information about any existing sub-national, institutional or national policies that has not been found prior to the interview/online survey.

Definition of Stakeholders

Stakeholders are defined as actors (individuals and organizations) involved in the development, adoption and/or implementation of policy related to postpartum health in Nigeria. These actors may be directly involved in work related to postpartum health or working in an area of global maternal health where they have had influence over the postpartum health policy in these countries. Stakeholders may be operating internationally or at the level of the country. Stakeholders’ institutional affiliations will be categorized (categories listed below). There is an understanding that some stakeholders may belong to more than one category; the stakeholders will be asked to self-identify with one of the categories below:

• global institution: Intergovernmental organisations who undertake activities to address global health issues

• academic institution: An educational institution dedicated to education and research with the ability to grant educational degrees

• government organization: An institution that is organisationally and financially not dependent on governments (non-state), non-profit orientation (non-market) and has a public benefit motive. These institutions deliver health interventions or lobby for change in policy to tackle health problems.

• non-governmental organisation/civil society organization: Associations of citizens (outside their families, friends and business) entered voluntarily to advance their interests, ideas and ideologies.

- Public health specialist institution: A public health agency or instutition dedicated to specialised health issues

• professional organization: A public health agency or instutition dedicated to specialised health issues (no reference)

6. Identification of Stakeholders

The identification of diverse and appropriate stakeholders will be imperative to the analysis of the data. I plan to identify the stakeholders by literature searches, and by personal contacts gained in the PRE-EMPT CLIP (Community Level Interventions for Pre-eclampsia) Study, and further via "snowballing". I plan to invite 40 stakeholders (knowing that some non-responses are expected) to complete in-depth interview in person in Lagos, via Skype or via the online questionnaire.

A sample size of 20 stakeholders (either via online questionnaires or interviews) would be large enough to give a broad range of perspectives on this topic and can be expanded if it feels necessary to explore in further details the issues being raised in earlier interviews.

-

7. Ethical considerations

Ethics approval from the University of British Columbia (UBC) and Children's and Women's Hospital and the local ethics board in Nigeria has been obtained for the CLIP Trial. To conduct interviews, an amendment to the CLIP Trial Protocol will be submitted to the UBC ethics board and to the local ethics board in Nigeria. I anticipate this will take one or two months for approval as this is an amendment.

The LSHTM Care Ethics application will be submitted along with this process; ethics approval from the ethics board at Olabisi Onabanjo Teaching Hospital, Nigeria has been obtained for stakeholder analysis, and for the amendment.

For the analysis of publically available policy documents, ethics approval is not required.

The online survey and in-depth interviews of stakeholders will be anonymised and will include the following statement about consent: "By competing this survey/questionnaire, you are giving consent to use the information for analysis and publication." Further,

participants will be advised that their individual comments will not be linked to their institutional affiliation, just the category. Other ethical considerations are listed below.

Online survey: No names or e-mail addresses will be collected for the online survey. The survey will be administered using a secure portal and survey responses will be downloaded and saved on a password-protect laptop. The responses will be deleted after completion of the study

Questionnaires: Names and e-mail addresses will be collected to contact the participants. The participant name will be anonymised, and individual comments would not indicate their institutional affiliation, rather just the category (as listed above). The questionnaires will be stored on a password-protected computer, and all files will be deleted after conclusion of the study.

In-depth interviews: Names and e-mail addresses will be collected to contact the stakeholder. The participant name will be anonymised, and individual comments would not indicate their institutional affiliation, rather just the category (as listed above). Interview data will be recorded and saved for transcription. This will be stored on a password-protected computer, and all files will be deleted after conclusion of the study.

8. How will the data be analyzed?

The qualitative data will be analysed using NVIVO. The framework for analysis will be decided after data collection has begun. A representative sample of the data set will be used to develop a thematic framework, and identify the major themes that emerge from the data. This thematic framework will be then developed into an index and applied to the full data set. Following this, the process of charting will be done to compare themes across cases. The data, and the charts will be reviewed to look for patterns across data and associations within the data.

To improve the quality of the qualitative analysis, the thoroughness of the analysis will be checked by comparison between cases, and within cases and also by being critical and testing of the emerging hypotheses.

The stakeholder interview will be used to triangulate findings from the literature review and the guidelines. The method of 'process tracing'[8] will be used to perform a within-case analysis to evaluate causal processes of decision making, and to enhance the comparative policy analysis.

9. References

1. WHO recommendations on postnatal care of the mother and newborn. Available from:http://apps.who.int/iris/bitstream/10665/97603/1/9789241506649_eng.pdf (accessed 17 December 2014)

2. Langlois EV, Miszkurka M, Zunzunegui MV, Ghaffar A, Zieglerc D, Karp I. Inequities in postnatal care in low- and middle-income countries: a systematic review and meta-analysis.

Bull World Health Organ 2015;93:259–270G| doi: http://dx.doi.org/10.2471/BLT.14.140996

3. Ashford L. *Hidden suffering: disabilities from pregnancy and childbirth in less developed countries*. Population Reference Bureau; 2002. Available from: http://www.prb.org/pdf/hiddensufferingeng.pdf[accessed 17 December, 2014].

4. Say L, Chou D, Gemmil A, Tunçalp Ö, Moller A, Daniels J, Gülmezoglu AM, Temmerman M, Alkema M. Global causes of maternal death: a WHO systematic analysis**.** *The Lancet Global Health*.2(6): e323-e333. DOI: http://dx.doi.org/10.1016/S2214-109X(14)70227-X

5. Babalola S, Fatusi A. Determinants of use of maternal health services in Nigeria–looking beyond individual and household factors. BMC Pregnancy Childbirth. 2009;9(1):43. doi: http://dx.doi.org/10.1186/1471-2393-9-43 PMID: 19754941

5. Rai RK, Singh PK, Singh L. Utilization of maternal health care services among married adolescent women: insights from the Nigeria Demographic and Health Survey, 2008. Womens Health Issues. 2012 Jul-Aug;22(4):e407–14. doi: http://dx.doi.org/10.1016/j.whi.2012.05.001 PMID: 22749200

6. Buse K, Mays N and Walt G (2005). Making Health Policy. Maidenhead: Open University Press, Chapter 1.

7. Global Programme on Evidence for Health Policy, World Health Organization. Guidelines for WHO guidelines. Available from: http://www.who.int/substance_abuse/activities/EIP_GPE_EQC_2003_1.pdf (accessed on 17 December 2014).

8. Collier D. Understanding Process Tracing. PS: Political Science & Politics. 2011; 44(4):823-30. Available from http://polisci.berkeley.edu/sites/default/files/people/u3827/Understanding%20Process%20Tracing.pdf (access on 15 January 2015).

9. The DHS Program Demographic and Health Surveys. Nigeria Demographic and Health Survey 2013 [Internet]. USAID [updated June 2014, cited 3 May 2015]. Available from:

http://dhsprogram.com/publications/publication-FR293-DHS-Final-Reports.cfm

Blood pressure thresholds in pregnancy for identifying maternal and infant risk: a secondary analysis of Community-Level Interventions for Pre-eclampsia (CLIP) trial data - Appendix

CLIP Trial Protocol – Summary of Changes

Community-Level Interventions for Pre-eclampsia (CLIP) Trial Protocol – Summary of changes

This supplement contains the following items:

- A summary of changes between the original protocol (dated 18 February 2013) and final protocol (dated 15 July 2017). There are nine different versions of the protocol with minor changes between each; these changes have been outlined below and are highlighted in red.

Version 1.3 (18 February 2013) **to Version 1.5** (27 June 2013)

**Subheading, page**	**Change(s)**	**V1.5**
Objective, **Pg. 2**	Addition of Gaza throughout the docoument	Maputo and Gaza Province (Mozambique
Pre-requisite knowledge: CLIP Feasibility Study **Pg. 3**	From D Sawchuck to P von Dadelszen throughout the document	(co-PIs: P von Dadelszen & R Qureshi)
The miniPIERS model (Figures 4 & 5) **Pg. 4**		The components of the miniPIERS model are: parity (muliparity vs. Nuliparity) gestational age at identification; symptoms (Y/N) of headache/ visual disturbances, chest pain/ dyspnoea, or abdominal pain with vaginal bleeding; systolic blood pressure (sBP); and dipstick proteinuria. The AUC ROC is 0.8788 [95% CI 0.4744, 0.80], decreasing to 73 on internal validation.
Figures 4 & 5 **Pg.5**		miniPIERS model ROC curve and calibration curve
**Pg. 10**		The trial will be phased from the Pilot CLIP trial to Definitive CLIP trial on the basis of a satisfactory rate of use (⩾50%) of the CLIP ‘package of care’ in appropriate women n all countries but Mozambique. Mozambique will be unique in that they will rely on an extended period of feasibility to pilot test all Trial systems and tools before directly beginning a definitive trial. Foregoing the Pilot in Mozambique was felt to be appropriate based on their experience with community-based surveillance and will ensure timelines of the trial are met within a manageable budget.<br><br>For all other countries, uuseu of the package in the Pilot phase

**Pg. 10**	Replacing HA with CHEWs	In Nigeria, these are Community Health Extension Workers (CHEWs)
Component 2a: cHCP training in home-based maternity surveillance<br>**Pg. 14**		b. Determination of gestational age (GA) by either ultrasound fetal biometry, last menstrual period dates, or measurement of symphysis-fundal height.
**Pg. 15**		We anticipate that CLIP training will take an initial 2-3 days and add an extra day for refresher training as needed or at a maximum of every 3 months, making a total of 8-9 extra days at most in each country.
**Pg. 15**	New paragraph added	These visits are not meant to supplement regular ANC clinical visits, but will be performed in coordination with those existing programs to achieve the frequency as previously described.
2b: Diagnosis & triage – miniPIERS & CLIP POM<br>**Pg. 15 and 16**	Removed<br>"When the final model was applied to the validation dataset, the discrimination ability was similar to that found in the development dataset, with an AUC ROC of 0.702 (0.643 – 0.761). The model retained good calibration ability. Validation did show some reduction in model performance based on the likelihood ratio (LR) associated with using 25% as a cut-off for a positive test (LR+ 4.98 [95% CI 3.35 – 7.42]) but this was not significant. The upper limit of the confidence interval for the positive LR remains > 5 showing the"	Added:<br>The final miniPIERS cohort used for analysis included 2081 women. The worst clinical values within the first 24hr after admission were used to develop the model. Variables included in the final miniPIERS model are parity (multiparity vs. nulliparity), gestational age on admission (or delivery if assessing a postpartum woman), the symptoms of chest pain and/or dyspnoea, headache and/or visual disturbances, vaginal bleeding with abdominal pain, and right upper quadrant pain; sBP; and dipstick proteinuria. The AUC ROC for this model was 0.6876868 (95% CI 0.37353 – 0.18011) which demonstrates a great ability to discriminate between women with and without adverse maternal outcomes<br><br>The stratification capacity of the model is good. Using a predicted probability cut-off of 25% resulted in a positive likelihood ratio of 5.09 [4.12, 6.29] and classified women with 85.000% accuracy, suggesting moderate utility of the model as a rule-in test for adverse maternal outcomes
2c: Lowering severe hypertension – oral methyldopa<br>**Pg. 18**	Remove:<br><br>•There are signs of recent stroke	

	•miniPIERS probability is ⩾ 25%.	
Clusters<br>**Pg. 21**		There will be no Pilot phase in Mozambique.definitive phase, we willwillwill include 12 (6<br>intervention and 6 control) clusters in the trial with a public and private sector referral facility in<br>the provinces of and GazaMaputoand Gaza.
**Pg 22**	2-3 paragraphs added	We will be using only one continuous will be maternalmaternal mortality and for the remaining three countries this will be Population size. The randomization algorithm ensures adequate balance on the stratification variable over the Intervention arm and the Control arm. Allocations that fail any of the balance criteria will be excluded, and only allocations that meet all criteria are eligible for consideration. The final allocation scheme is a random selection from the list of eligible allocations. The current algorithm is implemented using R Programming and can consider the following balance criteria: 1. Means ratios: the mean value over intervention clusters and the mean value over control clusters have a ratio no less than 0.9 and no greater than 1.1 (10%). 2. Mean relative to overall mean: the overall mean is the mean over all clusters; mean value in each arm is within 10% of overall mean. 3. Absolute difference in sums: the absolute difference between sums in the two arms is within 10,000. 4. Wilcoxin rank sum: Allocations where the Wilcoxin rank sum test have p-value
**Pg.22**		At the stage of analysis other cluster level factors identified as having an impact on the outcome will be adjusted for, including those used for stratification, such as (i) cHCP density by cluster, (ii) population density, (iii) distance to referral facilities (public or private)
Specifying and selecting study clusters<br>**Pg. 23**	Removed:<br>"The intervention and control clusters will be non-adjacent to reduce the risk of contamination."	

Maternal death<br>**Pg. 24**		(defined as the number of deaths during pregnancy or within 42 days of pregnancy (or last contact day if contact not maintained to 42 days) / 1,000 identified pregnancies), termed Maternal Death Rate.
Serious end-organ complications of pre-eclampsia:<br><br>**Pg.25**	Remove "intravenous/ injection sites"	Disseminated intravascular coagulation (DIC): abnormal bleeding from mucosa(mouth and/or ears)
A baseline household and facility survey<br>**Pg. 26**	New paragraph added	In all countries except India this will be performed based on retrospective information on pregnancies occurring in the previous year. In India, the baseline data will draw from the existing prospective Maternal and Neonatal Health Registry (MNH Registry).
Cross-sectional surveys of all households<br>**Pg. 27**		quarterly (Nigeria and Pakistan), 6-monthly (Mozambique) while in India, household surveys will continue prospectively using the MNH Registry
Facility-based chart audit<br>**Pg. 27**		throughout the Trial will occur in all but India where the MNH registry system already incorporates a facility chart review for each registered woman.
Geo-temporal analyses<br>**Pg. 32**	New paragraph added	Geospatial analysis will be conducted in each country by an individual from the PRE-EMPT Vancouver team with help from local GIS experts. This analysis will entail using the GPS tagged survey record to chart trends in maternal mortality and morbidity while cross referencing these outcomes to aspects of the built environment like transport and access to health care. Prior to this analysis, all personal identifiers would have been removed from the data. The data will be aggregated to describe frequencies for each of the captured variables at village level. This second set of aggregate data will be then be further analysed at UBC
Membership of the DSMB is<br><br>**Pg. 35**	Removal of:<br>•Mike English (TBC)<br>Reader, Department of Paediatrics, University of Oxford, Oxford, Oxon, UK and Honorary Lecturer, University of Nairobi, Nairobi, Kenya	

	(http://www.kemri-wellcome.org/people/researchers/mike-english).<br>•Md Abdul Quaiyum (TBC) Associate Scientist, Quality and Impact of Maternal Newborn Health Service Delivery Department, International centre for Diarrhoeal Disease Research, Bangladesh, Dhaka, Bangladesh.<br>(http://www.icddrb.org/media-centre/news/2190-icddrb-scientist-receives-grant-from-the-bill-and-melinda-gates-foundation-to-support-innovative-study-on-early-management-of-post-partum-haemorrhage).	
3.4 TRIAL REGISTRATION<br>**Pg. 36**	The CLIP Trial will be registered with ~~both ISRCTN and~~ clinicaltrials.gov.	The CLIP Trial will be registered with clinicaltrials.gov.
**Pg. 56**	Removed section on Health Assistamt (HA) Training and current scope of practice	
**Pg. 66**		NOTE: CLIP visits should only occur after the woman being assessed has registered for the trial and provided informed consent for her participation.
2.1.4 Step 4: Record demographic data<br>**Pg. 68**		Now will be collecting info about directins to household, age, phone number, history of previous deliveries.
Signs of unconsciousness<br>**Pg. 67**		While transport is being arranged the cHCP should ensure the woman is placed in the recovery position on her left side and ensure nothing is placed in the woman's mouth.
METHYLDOPA ADMINISTRATION INSTRUCTIONS<br>**Pg. 73**	Changes:	8. Stay with the woman to arrange transport to a recommended facility, if possible .<br>9. Complete the referral card by selecting all conditions experienced by the woman and medications<br>given. This card should be given to the woman or her accompanying family to be brought to the<br>facility with her.

		10. Save the tablet strip in the same box with woman's name, date of administration and the other<br>details
MAGNESIUM SULPHATE ADMINISTRATION INSTRUCTIONS		15. Stay with the woman asasas long as possible to help arrange transport, accompany the woman if<br>possible.<br>16. Complete the referral card by selecting all conditions experienced by the woman and medications<br>given. This card should be given to the woman or her accompanying family to be brought to the facility with her
3.3 FACILITY REFERRAL INSTRUCTIONS		2Assist the woman and family to identify the reason for referral by completing the referral card and providing it to them.

Version 1.5 (27 June 2013) **to Version 1.6** (09 December 2013)

**Subheading, page**	**Change(s)**	**V1.6**
Table 2 - CLIP-related maternity care service provision<br>**Pg. 7**	Multiple changes made within the table	
**Pg. 10**		In India, the Pilot phase will be shorter due to the difference in surveillance process as described later in this protocol.
**Pg. 11**		cHCPs will assess pregnant women with a target frequency of every 4 weeks at a minimum, and according to protocol (Appendix C: CLIP cHCP Working Protocol). These visits can occur in the home or PHC as both are considered part of the community for the purpose of the CLIP Trial.
Component 1: Community engagement<br>**Pg. 14**	More details added to The CLIP community engagement activities	
Component 2a: cHCP training in home-based maternity surveillance<br>**Pg. 15**	Education module for LHW was removed	
**Pg. 16**		4-weekly visits over the course of a pregnancy in women enrolled in the

		study will be considered a minimum standard for exposure to this aspect of the intervention; anything less than 4-weekly will be recorded as non-compliance with the intervention.
**Pg. 16**	Addition of Pakistan's emhamced education moduce	
**Pg. 18**		In Nigeria and Pakistan, women, their families and communities will be responsible for identifying and paying for transport to the CEmOC facility unless the woman is found in critical condition. In these cases an emergency trial transport fund will be made available to ensure no undue harm comes to women found in critical condition during a CLIP visit. The related fundraising activity will be a focus of the community engagement, particularly in these two countries to ensure in non-urgent situations women can get to care.
2d: Preventing & treating eclampsia – intramuscular MgSO4<br>**Pg. 19**	Addition to the optimal treatment protocol for CLIP	Through CPD activities, we will suggest optimal treatment protocols for CLIP; women referred to facilities. Following administration 10g i.m of MgSO4 in the community.
**Pg. 21**	Change from local council to PHC	In India, the unit of randomisation will be the PHC.local councils. All villages and PHCs within the PHC catchment
**Pg. 25**	Removed:<br>"We will identify the strata in which all study clusers fall in relation to population density and randomly select 2 clusters in each group (above and below the population median) to participate in the Pilot Trial phase, within each strata the clusters will then be randomly allocated to either intervention or control using a computer-generated random number."	
Inclusion/exclusion criteria<br>**Pg. 26**		Participants will be all consenting pregnant women aged 15-49 (except in Mozambique where reproductive age begins at 12)

Pilot CLIP Trials **pg. 27**	With the exception of India were assessment will occur at 6 months	To justify continuing on with the Definitive CLIP Trial, by 8 months into the 12 month Pilot (with the exception of India were assessment will occur at 6 months), at least 50% of eligible women (i.e., with a trigger) should have received the package of care.
Other outcomes **Pg. 29**	More details added on functional disability	Functional disability for any reason: Inability to perform usual daily duties at specific points in time during the postpartum period that would be acceptable and expected culturally (we are determining these normal limits through local focus group discussions; these will be defined for each country prior to the commencement of each Pilot CLIP Trial)
MEASURING THE OUTCOME MEASURES AT FOLLOW UP **Pg. 29 and 30**	More details about Nigeria added to baseline household and facility survey and cross sectional survey sections	
Data collection using mHealth technology **Pg. 30**	Removed paragraph on CLIP POM data and referral centers	Added: "The first will be used in all countries and the second and third will be used in Nigeria, Mozambique and Pakistan during the definitive trial phase"
OUTCOME ADJUDICATION COMMITTEE **Pg. 39**		The National Outcomes Adjudication Committee will consist of an uneven number of members of the national CLIP Team, including but not limited to one obstetrician, one paediatrician, and one methodologist/trialist. The UBC management team will provide final arbitration if required.
Mozambique **Pg. 52**	Agentes Polivalentes Elementares	Agente Communitarios de Saude

Version 1.6 (09 December 2013) **to Version 1.7 (** 06 August 2014)

**Subheading, page**	**Change(s)**	**V1.7**

The miniPIERS model (Figures 4 & 5)<br><br>**Pg. 5**	New paragraph added	In Mozambique and Pakistan an additional component to the miniPIERS mobile phone application will be the use of a low-cost mobile phone adapted pulse oximetry sensor to measure blood oxygen saturation (SpO2) in the pregnant population under study.
Tools & pre-requisite knowledge: Blood pressure monitoring in LMICs<br><br>**Pg. 5**	Change from Appendix D to E	See Appendix E for details on the validation study for the Microlife BP 3AS-2.
**Pg. 5**	New paragraph added	We will undertake qualitative analysis relating to the use of this tool in CLIP. Qualitative analysis plans are outlined in Appendix E of this protocol.
2.1 THE CLIP TRIAL DESIGN<br><br>**Pg. 9**	New sentence	The trial will be phased from the Pilot CLIP trial to Definitive CLIP trial on the basis of a satisfactory rate of use (⩾50%) of the CLIP ‘package of care’ in appropriate women n all countries but Mozambique (see section 2.2 intervention for details on the 'package of care').
**Pg. 9**	New sentence	Monitoring during the Pilot phase of the trial to determine rate of use of the CLIP package of care will be performed by the cHCPs using the POM mobile application in an ongoing manner after the Pilot trial begins.
**Pg. 9**	Minor changes throughout the document	BMGF changed to Bill and Melinda Gates Foundation (sponsor) and DSMB changed to Data Safety Monitoring Board (DSMB) throughout the document.
Component 1: Community engagement<br><br>**Pg. 12**	5. Transport and treatment funds<br><br>(minor changes within the first bullet point)	• Recognise the need to develop plans for financial resources when required in emergency conditions associated with pre-eclampsia/eclampsia. Funds may be personal or from the community. The community engager

		should facilitate the individual communities to form a plan for transport and treatment funds.
**Pg. 14**	Addition of a new bullet point	iv. SpO2 measurement using the LGT Medical audio oximeter connected to the mobile phone (Mozambique and Pakistan only)
**Pg. 15**		Removed this paragraph:<br>The CLIP POM data and clinical decisions will be transmitted to the referral CEmOC facilities so that those centres can both: (i) provide support, advice, and feedback to cHCPs, and (ii) anticipate and prepare for the arrival of women who are referred with severe disease through automatic notification by SMS texting.
**Pg. 15**		Added this paragraph:<br><br>In Mozambique and Pakistan additional CLIP triggers based on use of the audio oximeter will also be included in the POM decision aid. As with the original miniPIERS model, the enhanced model including SpO2 uses a risk threshold of $\geqslant$25% predicted probability to identify high-risk cases. Recommendations based on the updated miniPIERS model will include treatment with MgSO4 and urgent referral. An additional independent trigger of SpO2<93% will also be used in Mozambique and Pakistan to indicate urgent referral.
Lowering severe hypertension – oral methyldopa<br>**Pg. 16**	New paragraph added	Women will be given methyldopa if identified as having severe hypertension as defined above regardless of current medication use.
Component 3: Facility enhancement<br><br>**Pg. 18**	Minor changes within the sentence	In India and Pakistan, EmOC facilities may be shared by intervention and control clusters, whereas they are distinct in Nigeria and Mozambique.
**2.4 Method of Randomisation**<br><br>***Clusters***<br><br>**Pg. 19**	Minor changes within the sentence	There will be no Pilot phase in Mozambique. For definitive phase, we will include 12 (6 intervention and 6 control) clusters in the trial with a public and private sector referral facility in the provinces of Maputo (3 de Fevereiro, Maluana/Maciana, Ilha Josina/Calanga, Magude) and Gaza (Xilembene, Chicumbane, Nhancutse, Chibuto, Macia, Chissano, Mazivila, Messano).
2.6 INCLUSION / EXCLUSION CRITERIA	Minor changes within the sentence	In India and Mozambique, consent will be written

**Pg. 22**		
**Pg. 28**	Mozambique column has some changes<br><br>Before:<br>24, 000<br>n/a<br>4244,000<br>100 [81, 119]<br>50<br>44<br>10<br>TBD	After:<br>24,405<br>n/a<br>24,405<br>100 [81, 119]<br>50<br>44<br>10<br>170
Economic analyses<br>**Pg. 31**	Some more paragraphs added in this section	An economic evaluation of the intervention and its impact will be performed alongside the trial from a societal perspective (accounting for both costs to health care system and families) to guide health services decision-making for post-trial programme scale-up in the selected countries. (Full details of this plan are provided in Appendix F).
Qualitative analysis<br><br>**Pg. 32**	A new section added	We will use survey tools, FGDs and IDIs to explore feasibility and acceptability of use of the blood pressure device in CLIP in collaboration with the CRADLE study team. Full details of this plan are provided in Appendix E. In addition, a cross-sectional survey of cHCPs and physicians along with interviews of randomly selected women enrolled in the CLIP study and their families will be completed to understand the impact of the CLIP intervention package on cHCP decision making confidence.
Membership of the DSMB is:<br><br>**Pg. 34**	A few more names added	**Mario Merialdi**<br>Senior Director, Maternal and Newborn Health, Global Health, Becton, Dickinson and Company email: mario_merialdi@europe.bd.com

		**Brian A. Darlow**<br>CureKids Professor of Paediatric Research, University of Otago Christchurch, PO Box 4345 Christchurch 8140, New Zealand<br>(http://www.otago.ac.nz/healthsciences/expertise/profile/index.html?id=229
Appendix A<br><br>**Pg. 47**	New sentence under **Mozambique - Academic**	Manhiça Health Research Centre, Maputo, Mozambique
1.3 Document purpose and scope<br><br>**Pg. 59**	New bullet point added	• Measuring SpO2 (Mozambique and Pakistan only)
**Pg. 64**<br>**Pg. 68**	Adding section 2.11 and 2.29 on how to measure Blood Oxygen Saturation	Measuring Blood Oxygen saturation using the pulse oximeter (Mozambique and Pakistan only)
Appendix E<br>**Pg. 71**	Change in the title	Appendix E – Microlife BP 3AS2 Validation test results and<br>Qualitative Evaluation Plans
**Pg. 73**	Addition of a whole new section	CRADLE Qualitative Evaluation during CLIP Study
**Pg. 76**	Addition of a new appendix	Appendix F

Version 1.6 (06 August 2014) **to Version 1.8** (08 June 2014)

**Subheading**	**Change(s)**	**V 1.8**
Current practice - the provision of maternity care and pre-eclampsia interventions Nigeria<br>**Pg. 48**		Care is accessed through both public and private facilities throughout OGUN State with an estimated 40% of women accessing care through private institutions and 60% attending public facilities.

**Pg. 49**	Addition of Health Assistant section	Health Assistants (HA's) training & current scope of practice
3.2 MAGNESIUM SULPHATE ADMINISTRATION INSTRUCTIONS<br><br>**Pg. 71**		mmHg (or diastolic blood pressure ⩾110 mmHg in Nigeria only)

Version 1.8 (08 June 2014) **to Version 1.9** (16 December 2014)

**Subheading**	**Change(s)**	**V 1.9**
Figure 8: CLIP intervention diagram<br><br>**Pg. 11**	Additional triggers for Nigeria, Mozambique and Pakistan	In green (for Nigeria), there are additional triggers for severe diastolic hypertension and shock. In red (Mozambique and Pakistan), there is an additional trigger for SpO2 ⩽93%.
Tools & pre-requisite knowledge: Blood pressure monitoring in LMICs<br>**Pg. 6**	Minor changes to the sentence	Addition of Microlife CRADLE VSA, throughout the document.
2b: Diagnosis & triage – miniPIERS & CLIP POM<br><br>**Pg. 16**	Adding no.8 to the list	8. Shock index ⩾ 1.7 in Nigeria only
	Additional triggers added for Nigeria	In Nigeria, additional triggers will be included for severe diastolic blood pressure or severe shock index to coincide with the traffic light warning signs included in this device (see Appendix E).
2c: Lowering severe hypertension – oral methyldopa<br><br>**Pg. 17**	Addition of dBP	sBP is ⩾160 mmHg, or dBP ⩾110 mmHg (in Nigeria only)
Neonatal morbidity<br>**Pg. 24**	Minor changes and additions within the section	
Qualitative analyses<br><br>**Pg. 33**	Plan to undertake a policy analysis for Nigeria	We aim to undertake a policy analysis to evaluate whether the national policies in the state of Ogun, Nigeria respond to the needs of postpartum women.

**Pg. 10**<br>**Pg. 14**	Addition of Health Assistants for Nigeria throughout the document	In Nigeria, there are CHEWs and Health Assistants (Has);
Appendix A<br>**Pg. 50**	More information on access to care in Nigeria	40% of women accessing care through private institutions and 60% attending public facilities.
**Pg. 32**		Furthermore, to enhance the understanding of the health policy environment, we aim to undertake a policy analysis to evaluate whether the national policies in the state of Ogun, Nigeria respond to the needs of postpartum women i.e. how they address any health condition attributed to and/or aggravated by pregnancy and childbirth that has a negative impact on the woman's wellbeing following childbirth. Further details about this policy analyses can be seen in Appendix G.
**Pg. 83**	Addition of Appendix G	Appendix G

Version 1.9 (16 December 2014) **to Version 2.0** (07 July 2015)

**Subheading**	**Change(s)**	**V 2.0**
**2b: Diagnosis & triage – miniPIERS & CLIP POM**<br><br>**Pg.15**	GA>20 weeks was added to the treatment criteria	1. Unconsciousness (MgSO4 if sBP ⩾160 mmHg and gestational age is equal to or greater than 20 weeks (GA⩾20 weeks) [to be reasonably sure that the unconsciousness is associated with severe pre-eclampsia and not due to obstetric sepsis], urgent transport)<br>2. Signs of recent stroke or seizure (methyldopa if sBP ⩾160 mmHg [to ensure BP is not lowered too much], MgSO4 (if GA⩾20 weeks) urgent transport)<br>3. Significant vaginal bleeding (MgSO4 if sBP ⩾140 mmHg and GA⩾ 20 weeks [presumed abruption associated with severe pre-eclampsia] , urgent transport).<br>4. No fetal movements felt in the previous 12 hrs (urgent transport [a threshold for identifying at risk fetuses

		that are alive at the time of screening] 39)<br>5. sBP ⩾160 mmHg (or dBP ⩾ 110 mmHg in Nigeria only) (methyldopa, MgSO4 (if GA⩾20 weeks) urgent transport [consistent with severe pre-eclampsia])<br>6. Heavy proteinuria (⩾4+ by dipstick – predictive of stillbirth in miniPIERS cohort, urgent transport)<br>7. miniPIERS predicted probability ⩾25% (MgSO4 (if GA⩾20 weeks)urgent transport)<br>8. Shock index ⩾ 1.7 in Nigeria only (the Shock index is a ratio of pulse/sBP; high shock index is an indication of poor prognosis in women with postpartum haemorrhage)

Version 2.0 (07 July 2015) **to Version 2.1** (10 March 2016)

Subheading	Change(s)	V 2.1
**Figure 1**<br><br>**Pg.1**	Deleted the description under the figure:<br><br>The origins and consequences of pre-eclampsia<br>ARDS acute respiratory distress syndrome; ARF acute renal failure; ATN acute tubular necrosis; CVA cerebrovascular accident; DbM diabetes mellitus; DIC disseminated intrasvascular coagulation; EVT extravillous trophoblast; IUGR intrauterine growth restriction; LV left ventricular; PRES posterior reservible leukoencephalopathy syndrome; RIND reversible ischaemic neurological deficit; SNPs single nucleotide polymorphisms; TIA transient ischaemic attack	

1.3 RELEVANT CONVERGENT ACTIVITIES<br><br>***Pre-requisite knowledge: CLIP Feasibility Study***<br><br>**Pg. 3**	New sentence added:	A process evaluation may be conducted in all the sites to provide information on mechanisms of impact
2.16 Frequency of analyses<br>**Pg. 33**	Interim analyisis added	In the event that the site is unable to reach planned sample size, due to logistical and pragmatic challenges, then the interim analysis will be conducted once complete pregnancies (until 42 days postpartum) are expected for women making up half of the projected sample size for that country
The miniPIERS model<br><br>**Pg. 5**	New paragraph added after:	Raw photoplethysmogram (PPG) will be recorded to facilitate the optimal estimation of hearth rate, heart rate variability, pulse pressure variation, oxygen saturation and other parameters
	New paragraph added	Raw pressure waveforms will be recorded during the measurement of blood pressure
2.6 INCLUSION / EXCLUSION CRITERIA<br>**Pg. 23**	written consent and verbal concent added	written consent for surveillance (head of household/women of reproductive age) and verbal consent for the secondary visits for the entire CLIP trial
2.15 TYPES OF ANALYSES/STATISTICAL PLAN<br><br>**Pg. 31**	New sentence	A detail of analysis plan is included in the CLIP cRCT Statistical Analysis Plan.

Version 2.1 (10 March 2016) **to Version 2.2** (15 July 2017)

**Subheading, pg. no**	**Change(s)**	**V 2.2**
Table 2 CLIP-related maternity care service provision *(see table inserted below)*: **Pg. 7**		

	Nigeria	Mozambique	Pakistan	India
Community-based care				
HOME-BASED CARE				
cHCPs	Community Health Extension Workers (CHEWs; cover 2500 population each) Health Assistants (Has)	Agente Communitarios de Saude (APEs; cover 500-2000 population each)	Lady Health Workers (LHWs; cover 1000 population each)	Auxiliary Nurse Midwives (ANMs; cover 3000-5000 population each) Accredited Social Health Activists (ASHAs; cover 1000 population each)
Delivery in the home	70% births at home	30% births at home	40% births at home	5-10% births at home
PHC-based care				
Medical officers	Sometimes	No	Yes	Yes
Nurses	Yes	Yes	Yes	Yes
cHCPs	CHEWs, Health Assistants (HA)	ACSs	LHWs	ANMs, Lady Health Visitors (LHV)
BP device	Yes (93%)	Yes	Yes (100%)	Yes (100%)
Urine dipsticks	Sometimes (20%)	Yes	Few (37%)	Yes (90%)
Laboratory testing	Sometimes (11%)	Some	Yes (100%)	No
Ultrasound	Sometimes (5%)	No	Sometimes	No
Methyldopa	Yes (purchased by patient at pharmacy)	Yes	Yes	Yes
MgSO4	Availability of some anticonvulsant	Yes	Sometimes (38%)	Sometimes (56%)

	(82%)			
Attend deliveries	Yes	Yes	Yes	Yes
BEmOC	Varies	No	Yes	No
Hours of available care	Information pending	Information pending	Day time only	Some services are 24/7
Facility-based care				
Number of facilities serving CLIP clusters	936<br>(32 public, 904 private)	56	12<br>(3 public, 9 private)	96
BEmOC in CLIP clusters	Information pending	10	17<br>(14 public, 3 private)	Information pending
CEmOC in CLIP clusters		1	12	Majority
General practitioners/ medical officers	Yes	Yes	Yes	Yes
Specialists	Rarely	Rarely	At some highest level facilities	Yes
Nurses	Yes	Yes	Yes	Yes
cHCPs	Yes	Yes	No	ANMs
BP device	Yes	Yes	Yes (100%)	Yes (100%)
Urine dipsticks	Yes	Yes	Sometimes	Yes
Laboratory testing	Yes	Yes	Yes (100%)	Yes
Ultrasound	Yes	Yes	Yes	Yes
Fetal heart assessment	Pinard; US	Pinard, Doppler, US	Pinard, CTG, US	Pinard, Doppler, CTG, US
Methyldopa	Sometimes (30%)	Yes	Yes	Yes
Other antihypertensives	Sometimes (7%)	Yes	Yes	Yes
MgSO4	Sometimes (20% stock outs)	Yes	Some	Yes

3.2 Data Safety and Monitoring Board	The DSMB will be created, including methodological and content expertise and will be comprised mainly of investigators from LMICs.	The DSMB will be created, including methodological and content expertise.
Appendix E	Addition of N3 BP validation test	Appendix E – Microlife BP 3AS1-2 and N3 BP Validation test results and Qualitative Evaluation Plans
	1.2.2 Planned modifications to the device	1.2.2 Microlife N3 BP device

	Changes from future tense to past tense	CLIP and CRADLE research groups have completed work with our partners at Microlife to adapt the 3AS1-2device to further suit the environment.
	New addition of CRADLE device indicators for warning lights	The new Microsoft CRADLE Vital Signs Alert device includes the following indicators for a warning light:
	New addition	In the CLIP trial, only indicators for a red light will be included as triggers for immediate treatment or referral. This device is currently being validated for use in pregnancy.<br><br>Insert picture<br><br>Figure 2: N3 BP device showing a normal (green light) blood pressure and heart rate reading.
Appendix F<br>**Pg. 83**	Addition of:<br><br>METHODS AND RESEARCH PLAN (INDIA)	

Blood pressure thresholds in pregnancy for identifying maternal and infant risk: a secondary analysis of Community-Level Interventions for Pre-eclampsia (CLIP) trial data - Appendix

CLIP Trial Statistical Analysis Plan (Original)

Document Title:	**Statistical Analysis Plan**
Version:	0.1
Date:	October 16, 2015
Author:	
Protocol Number:	
Study Title:	Community level interventions for pre-eclampsia (CLIP)
Principal Investigator:	Peter von Dadelszen

This document has been reviewed and approved by:

	**Signature**	**Date**
Statistician		
Programme Head		
Principal Investigator		

Table of Contents

1 Introduction 3
  1.1 Study Description 3
  1.2 Primary Objectives 4
  1.3 Secondary Objectives 6
  1.4 Eligibility Criteria 7
2 Data Summaries and Analyses 8
  2.1 Trial Information and Patient Disposition 8
  2.2 Baseline and Demographic Variables 8
  2.3 Primary Analysis 9
    2.3.1 Sensitivity analyses 11
    2.3.2 Subgroup analyses 11
    2.3.3 Per-protocol analyses 12
  2.4 Secondary Analyses 12
  2.5 Interim Analysis 13
  2.6 Safety Data 14
    2.6.1 Adverse Events (AEs) 14
  2.7 Individual patient data meta-analysis (IPD) 14
3 Additional Methodological Details 15
  3.1 Statistical Tests 15
  3.2 Software to be Used 15

1 Introduction

1.1 Study Description

A cluster randomised trial designed to evaluate the implementation of community-level evidence-based care aimed at reducing pre-eclampsia-related maternal and perinatal mortality and major morbidity.

The CLIP intervention consists of the following three components:

(i) Community engagement including community leaders, the women of the communities themselves, and their mothers, husbands, and mothers-in-law, regarding pre-eclampsia, its origins, symptoms, signs, and potential consequences, pre-permissions for maternal transport, and fundraising activities around transport and treatment costs;

(ii) Provision of hypertensive disorders of pregnancy (HDP) oriented antenatal care through CLIP visits and use of CLIP Piers On the Move (POM) tool for risk stratification. Community-based health care providers (cHCPs) will assess pregnant women with a target frequency of every 4 weeks at a minimum, and according to protocol. These visits can occur in the home or primary health centre (PHC) as both are considered part of the community for the purpose of the CLIP Trial. They will be trained to enquire about women's symptoms (using country-specific pictograms), take women's blood pressure (BP) (using systolic blood pressure (sBP)), and check urine for protein using dipstick on the first visit or on any subsequent visits for sBP ≥140 mmHg. This will inform the diagnosis and risk assessment of women with pre-eclampsia.

(iii) Use of the CLIP 'package of care' for women with a CLIP 'trigger' as indicated on the POM tool (i.e., oral antihypertensive therapy when indicated, intramuscular (i.m.) $MgSO_4$ when indicated; and appropriate referral to an comprehensive emergency obstetric care (CEmOC) facility when indicated).

?? facility enhancement >> there seems to be some inconsistency in the protocol. protocol lists the 3 components above but in the details include facility enhancement as the third component. What do we want to use here?

The trial is designed to occur in two phases, a Pilot phase and a Definitive phase. The Pilot phase will involve four clusters per country, and the Definitive phase will include all clusters.

The design of Pilot is as follows:

	**Pakistan**<br>(Hyderabad and Matiari districts in Sindh Province)	**India**<br>(Belgaum and Bagalkot districts in Karnataka State)
**Timeline**	Feb 2014 – Dec 2014	Feb 2014 – Oct 2014
**Number of control clusters**	2	2
**Number of intervention clusters**	2	2
**Data collection**	Ongoing pregnancy registration;<br>Regular household surveys on quarterly basis;<br>Facility surveillance in public tertiary level hospitals only	Using MNH Registry with three time points of data collection:<br>Pregnancy identification;<br>Within 7 days of delivery;<br>42 days postpartum

The design of Definitive is as follows:

	**Mozambique**<br>(Maputo and Gaza Province)	**Pakistan**<br>(Hyderabad and Matiari districts in Sindh Province)	**India**<br>(Belgaum and Bagalkot districts in Karnataka State)
**Timeline**	Apr 2015 -	Mar 2015 -	Nov 2014 -
**Number of control clusters**	6	10	6
**Number of intervention clusters**	6	10	6
**Data collection**	Ongoing pregnancy registration;<br>Regular survey and facility surveillance every six months;<br>Maternal and Perinatal verbal autopsies for all deaths	Ongoing pregnancy registration;<br>Regular surveys on quarterly basis;<br>Facility surveillance in public tertiary level hospitals only;<br>Maternal and Perinatal verbal autopsies for all deaths	Using MNH Registry with three time points of data collection:<br>Pregnancy identification;<br>Within 7 days of delivery;<br>42 days postpartum

1.2 Primary Objectives

- Pilot Trial –
    - To implement the use of the CLIP 'package of care' and to observe at a minimum 50% referral (urgent or non-urgent, to a facility able to provide CEmOC) in women with a CLIP trigger.

- Definitive Trial –

- To reduce pre-eclampsia-related, and all-cause, maternal and perinatal mortality and major morbidity by 20% or more in intervention clusters.

The detailed breakdown of the components of the combined outcome is as follows:

Maternal death (defined as the number of deaths during pregnancy or within 42 days of pregnancy (or last contact day if contact not maintained to 42 days) / 1,000 identified pregnancies)

Maternal morbidity (defined as the number of women with one or more life-threatening complications of pregnancy during pregnancy or within 42 days of pregnancy or last contact day if contact not maintained to 42 days) / 1,000 identified pregnancies). These are the serious end-organ complications of pre-eclampsia, other major causes of maternal mortality, or life-saving interventions related to one of the aforementioned:

Serious end-organ complications of pre-eclampsia:

- *Eclampsia:* occurrence of generalised convulsions during pregnancy, labour or within 42 days of delivery in the absence of epilepsy or another condition predisposing to convulsions
- *Stroke:* hemiparesis and/or blindness developed during pregnancy or in the 42 days postpartum lasting greater than 48 hours
- *Coma:* prolonged unconsciousness ≥12 hours
- *Antepartum haemorrhage:* vaginal bleeding ≥ 15 mL with or without pain before the onset of labour
- *Disseminated intravascular coagulation* (DIC): abnormal bleeding from mucosa(mouth and/or ears)

Other major causes of maternal mortality:

- *Obstetric sepsis:* In the community, defined as fever and one of: abdominal/uterine tenderness, foul smelling vaginal discharge/lochia, productive cough and shortness of breath, dysuria or flank pain, headache and neck stiffness. In the facility, defined as presence of fever (>38°C), a confirmed or suspected infection (e.g., chorioamnionitis, septic abortion, endometritis, pneumonia) and at least one of the following: heart rate >90/min, respiratory rate >20/min, leukopoenia (total leukocyte count [TLC] $<4 \times 10^9$/L) or leukocytosis (TLC $>12 \times 10^9$/L)
- *Vesicovaginal or rectovaginal fistula:* continuous loss of urine and/or faeces after delivery

Life-saving interventions:

- *Cardiopulmonary resuscitation:* a set of emergency procedures including chest compressions and lung ventilation applied in cardiac arrest victims
- *Dialysis:* haemodialysis and/or peritoneal dialysis
- *Mechanical ventilation* (other than for Caesarean section): intubation and ventilation not related to anaesthesia
- *Blood transfusion:* ≥1 unit
- *Interventions for major postpartum haemorrhage:* brace sutures, external and internal uterine

compression, anti-shock garment use, internal iliac artery ligation and/or hysterectomy with or without transfusion

__Perinatal & late neonatal death__ (defined as stillbirth [≥20^{+0} and/or ≥500g], early neonatal mortality [d 0-7 of postnatal life] and late neonatal mortality [d 8-28 of postnatal life] / 1,000 identified pregnancies)

__Neonatal morbidity__ (defined as occurrence of a primary neonatal morbidity during d 0-28 of postnatal life / 1,000 identified pregnancies). The following are the primary neonatal morbidities:

- ***Feeding difficulty***
  - *Including inability to suckle normally or latch on to the mother's breast to feed even if the mother's milk is not let down*
- ***Breathing difficulty***
  - *Including grunting and in-drawing of the abdomen under the ribs*
- ***Seizure***
  - *Occurrence of any seizure event (fits)*
- ***Lethargy***
  - *Baby not appearing normally wakeful after activities such as feeding or sleeping*
- ***Coma***
  - *Not medically induced period of unconsciousness of any length*
- ***Hypothermia***
  - *Cold to touch*
- ***Umbilical cord infection***
  - *Characterized by discharge from and redness around the umbilical stump*
- ***Skin infection***
  - *Any appearance of abnormally red, black, swollen and blistered skin with pus*
- ***Bleeding***
  - *From anywhere*
- ***Jaundice***
  - *Yellow skin and eyes*
- ***Central nervous system related morbidity***
  - *Abnormal amount of vomiting as defined by the parents or caregiver with bulging or sunken fontanelle*

1.3 Secondary Objectives

- Pilot trial –

- To compare pre-eclampsia-related, and all-cause, maternal and perinatal mortality and major morbidity in intervention versus control clusters.

- Other outcomes will be the outcomes described below for the Definitive CLIP Trials.

• Definitive trial –

- To measure the impact of the CLIP intervention on the delays around triage and transport by analyzing birth preparedness and the number of women present for care at a CEmOC.

- Birth preparedness and complication readiness as measured by any three of the following: (1) arranged for transport; (2) obtained prior permission for transport should emergency arise; (3) saved money for obstetric care; (4) identified skilled birth attendant; (5) identified facility for delivery. This will evaluate the success of community engagement.

- Number of women presenting for care at a CEmOC facility and number of facility births should increase if triage (using the miniPIERS risk stratification and POM) is effective, and if there is compliance with transport to CEmOC facility.

- Other outcomes will be evaluated such as knowledge of pre-eclampsia/eclampsia among women of reproductive age; total number of seizures; number of pre-admission and post-admission seizures; adverse events; functional disability; and cost-effectiveness of the CLIP intervention. In addition, other outcomes for the perinatal morbidities are neonatal fever, congenital anomaly and birth injury.

- Other outcomes also include functional disability for any reason: Inability to perform usual daily duties at specific points in time during the postpartum period that would be acceptable and expected culturally (we are determining these normal limits through local focus group discussions; these will be defined for each country prior to the commencement of each Pilot CLIP Trial)

1.4 Eligibility Criteria

Participants will be all consenting pregnant women aged 15-49 in Pakistan and India, all consenting pregnant women aged 12-49 in Mozambique, identified by the community health care providers (cHCP) assigned to their community. No such pregnant women will be excluded from the analysis which will be by intention-to-treat.

2 Data Summaries and Analyses

2.1 Trial Information and Patient Disposition

The following information will be presented:

- Number of participants enrolled
- Number of antepartum surveys completed
- Number of postpartum surveys completed
- Number of POM visits completed
- Number of referrals recommended
- Number of referrals accepted
- Frequency table for loss to follow-up

2.2 Baseline and Demographic Variables

Summary statistics such as the mean, median, standard deviation, minimum, maximum and inter-quartile range for continuous variables and the number and percentage of patients with various levels of categorical variables will be calculated for all subjects randomised within each treatment group for each country for variables such as:

- Demographics: maternal age, parity, gestational age at enrollment, method of gestational age determination, maternal past medical history of hypertension or seizures/epilepsy, past obstetric history of hypertension, distance from facility
- Socioeconomic status (measured by poverty index), level of education
- Cluster level variables: cHCP density, population density
- Interventions: number of antenatal visits, type of care provider, route of birth, medications given

All summary statistics will be calculated for each treatment group individually as well as for all treatment groups combined.

CRF	Section	Field
Pregnancy registration	Demographic information	• Age
Pregnancy registration	Obstetric history	• Parity<br>• High blood pressure<br>• Seizures
Pregnancy registration	Gestational age assessment	• EDD / LMP
Pregnancy registration	Medical history	• High blood pressure<br>• Seizures
Regular surveillance	Cost of newborn interventions / admissions	• Time to facility
Baseline survey	Household information	• Poverty index
Pregnancy registration	Demographic information	• Years of education
Pregnancy registration	Demographic information	• Partner in house (Mozambique only)
Regular surveillance	Care-seeking behaviour	• Number of antenatal care visits
Regular surveillance	Care-seeking behaviour	• Care sought from health care provider
Regular surveillance	Delivery	• Route of delivery
Regular surveillance	Delivery	• Current antihypertensive medications
Piers on the Move	Visit	• Methyldopa given/accepted
Piers on the Move	Visit	• MgSO4 given/accepted

2.3 Primary Analysis

Pilot phase –

Referral acceptance is calculated as follows: 1) If the CLIP visit (the visit during which the referral was recommended) has a follow-up visit, acceptance is indicated by a “Yes” response to the question "Did you go to hospital"; 2) If the CLIP visit has no follow-up information, acceptance is indicated by a “Accepted” response immediately given following the recommendation ("Transport - Accepted"). The percentage of acceptance is defined by acceptance of all visits with the recommendation.

Refusal of acceptance is calculated as follows: 1) If the CLIP visit (the visit during which the referral was recommended) has a follow-up visit, refusal is indicated by a “No” response to the question "Did you go to hospital"; 2) If the CLIP visit has no follow-up information, refusal is indicated by a “Refused” response immediately given following the recommendation ("Transport - Refused").

Missing is calculated as follows: If the CLIP visit has no follow-up information and no answer given immediately following the recommendation.

Follow-up response (Did you go to hospital?):	Yes			No			Missing		
**Response given immediately following recommendation:**	**Accept**	**Refuse**	**Missing**	**Accept**	**Refuse**	**Missing**	**Accept**	**Refuse**	**Missing**
Acceptance	√	√	√	X	X	X	√	X	Missing

CRF	Section	Field
Piers on the Move	Visit	• Transport
Piers on the Move	Follow-up	• Went to hospital after last home visit

Definitive phase -

The primary outcome is the proportion of pregnancies resulting in at least one of maternal death, stillbirth, neonatal death, or severe morbidity in the mother or child. A hierarchical regression model will be used to take into account the clustering of women within the units of randomisation. The model will take into account key determinants of outcome, as defined by the investigators, at both the individual and cluster level.

CRF	Section	Field
Regular surveillance	Demographic information	• Deceased
Regular surveillance	Maternal conditions and interventions	• Cardiopulmonary resuscitation (CPR)<br>• Mechanical ventilation<br>• Dialysis<br>• Blood transfusion<br>• Disseminated intravascular coagulation (DIC)<br>• Antepartum haemorrhage<br>• Anti-shock garment<br>• Hysterectomy<br>• Stroke<br>• Seizure<br>• Coma

CRF	Section	Field
		• Obstetric sepsis
Regular surveillance	Perinatal outcomes	• Birth outcome<br>• Death within one week of birth<br>• Death between one week and one month of age
Regular surveillance	Perinatal outcomes	• Feeding difficulty<br>• Breath difficulty<br>• Seizure<br>• Lethargy<br>• Coma<br>• Hypothermia<br>• Umbilical cord infection<br>• Skin infection<br>• Bleeding<br>• Jaundice<br>• Central nervous system related morbidity

2.3.1 Sensitivity analyses

We will use multiple imputations to account for those lost to follow-up and examine whether this has any impact on the between-group comparisons.

2.3.2 Subgroup analyses

We will analyse the interventions and outcomes by country and locality. Other subgroup comparisons may include:

- Characteristics of the women
  - Primips vs. multips
  - Women who live close (vs. far) from the PHC
  - Literate (vs. illiterate)
- Characteristics of the clusters
  - Large (vs. small) clusters
  - Density of health care provider per population

- Effect of time

2.3.3 Per-protocol analyses

All analyses will be based on intention-to-treat basis, and may be repeated based on per-protocol basis (i.e. subjects that are compliant). Performing per-protocol analyses is reasonable because of challenges with application of the intervention. The following criteria may be used to define differing degrees of compliance:

- (i) Women who received a CLIP visit(s)
- (ii) Women who received CLIP visits at least once every 4 weeks and once postpartum
- (iii) Community engagement in cluster may consider factors such as:
    - Engagement of household male decision-makers
    - Frequent (vs less frequent) meetings
    - Large (vs. small) meetings
    - Engagement of community leaders (male or female)
    - Completeness of the topics covered

2.4 Secondary Analyses

Similar methods will be applied to the individual components of the primary outcome. In addition, the proportion of women achieving birth preparedness and complication readiness will be compared as will be the proportion of women presenting for care at an CEmOC facility, and the proportion of facility births.

Other outcomes -

Antenatal care sought; N times BP measured by health professional; received $MgSO_4$; antihypertensive therapy received; knowledge of pre-eclampsia and eclampsia; total number of seizures or number of pre-admission and post-admission seizures and cost-effectiveness of the intervention; functional disability.

CRF	Section	Field
Regular surveillance	Delivery information	• Location of delivery<br>• Blood pressure measured<br>• Antihypertensive medications
Regular surveillance	Birth preparedness	• Where to delivery<br>• Skilled birth attendant<br>• Transportation plan

CRF	Section	Field
		• Funds saved for emergencies<br>• Permission to go seek care
Regular surveillance	Care-seeking behaviour	• Care sought from health care provider<br>• Visits to primary health centres<br>• Visits to higher level facilities
Regular surveillance	Pre-eclampsia knowledge	• Abnormal bleeding after delivery<br>• High blood pressure during pregnancy<br>• High blood pressure can cause death of pregnant women<br>• Seizures during pregnancy<br>• Headache<br>• Visual disturbance<br>• Chest pain<br>• Shortness of breath<br>• Nausea and vomiting<br>• Abdominal pain<br>• Vaginal bleeding<br>• Unconsciousness<br>• Stroke<br>• Seizures<br>• Unusual swelling
Regular surveillance	Medications and adverse effects	• Received MgSO4
Regular surveillance	Functional disability	• Take care of newborn<br>• Wash babies' clothes<br>• Prepare meals<br>• Clean the house<br>• Get water<br>• Work in the fields<br>• Go to the market<br>• Return to work/paid employment

2.5 Interim Analysis

An interim analysis is planned for each of the four countries once complete data (until 42 days postpartum) has been received for women making up half of the planned sample size for that country. The stopping rule for both benefit and harm will require an observed difference between groups associated with an alpha <0.001 (power: 80%). Reporting and handling of adverse events will be in accordance with Good Clinical Practice (GCP) guidelines.

2.6 Safety Data

2.6.1 Adverse Events (AEs)

Adverse events will be tracked in intervention and control clusters related to:

- Methyldopa administration in the community: relative maternal hypotension on arrival at facility (defined as sBP<110 mmHg)
- MgSO4 administration in the community: either respiratory depression, coma or death during transport, as diagnosed upon arrival at facility
- Transport: Transport-related injury (life or limb) or death during transport
- injection site haematoma or infection (following either community or facility administration of i.m. MgSO4)
- ≥ 20% of women referred to facility being sent back to their communities without follow-up (monitoring community engagement and the CLIP POM).
- The following will be presented by treatment group: number of adverse events (overall and by type), number of women with one or more adverse event(s) (overall and by type).

CRF	Section	Field
Piers on the Move	Visit	• Methyldopa<br>• Blood pressure measurement<br>• MgSO4
Piers on the Move	Followup	• Physical injury during transport<br>• Haematoma at injection site<br>• Infection at injection site

2.7 Individual patient data meta-analysis (IPD)

Each country will be analysed separately, with a planned IPD meta-analysis (conducted at UBC) after the completion of all three trials. For the IPD meta-analysis, we will analyse data for women recruited

at ≥20 weeks, to standardise data to the latest public declaration of pregnancy (i.e., 20 weeks in Pakistan).

3 Additional Methodological Details

3.1 Statistical Tests

All statistical tests will be two-sided, with significance levels of 0.05, unless stated otherwise.

3.2 Software to be Used

SAS / R.

Blood pressure thresholds in pregnancy for identifying maternal and infant risk: a secondary analysis of Community-Level Interventions for Pre-eclampsia (CLIP) trial data - Appendix

CLIP Trial Statistical Analysis Plan (Final)

Document Title:	**Statistical analysis plan for CLIP Pilot and Definitive Trials**
Version:	1.7
Date:	Drafted 16 October 2015, amended last March 1, 2017
Authors:	Peter von Dadelszen, Tang Lee, Joel Singer, Hubert Wong, Sumedha Sharma, Beth Payne, Marianne Vidler, Zulfiqar Bhutta, Laura A Magee
Protocol Number:	ClinicalTrials.gov identifier NCT01911494
Study Title:	Community level interventions for pre-eclampsia (CLIP) Trials
Principal Investigator:	Peter von Dadelszen

This document has been reviewed and approved by:

Statistician<br>*Tang Lee*	[signature]	March 1, 2017
Program co-ordinator<br>Sumedha Sharma	[signature]	March 1, 2017
Principal Investigator<br>Peter von Dadelszen	[signature]	March 1, 2017

Table of Contents

1. Introduction 3
1.1 Study Description 3
1.1.1 Update to CLIP Nigeria 3
1.2 Trial Objectives..4
Primary Objectives 4
Secondary Objectives..4
1.3 Sample size calculations..4
2.0 Data summaries and analyses..5
2.1 Trial information and patient disposition 6
2.1.1. Consort diagram..6
2.1.2. Baseline and demographic variables 6
2.2 Primary analysis 8
2.2.1 Pilot Trials..8
2.2.2 Definitive Trials 8
2.2.2.1 Definition of the primary outcome..9
2.2 2.2 Calculation of the primary outcome 10
2.2.2.3 Analysis of the primary outcome..10
2.2.3 Sensitivity analyses 11
2.2.3.1 Completed pregnancies with postpartum follow-up..11
2.2.3.2 Complete postpartum follow-up (for all components of primary outcome) 11
2.2.3.3 Women whose EDD+3 weeks falls within the trial timeline..11
2.2.3.4 Women whose EDD+3 weeks plus 42 day follow up period falls within the trial timeline.11
2.2.3.5 Without adjustment..11
2.2.3.6 For women in India 11
2.2.3.7 Evaluating 'on treatment' effect:..11
2.3. Application of the intervention..11
2.4 Secondary Analyses..12
2.4.1 Secondary outcomes..12
2.4.2 Other Outcomes..13
2.4.3 Other Planned secondary analysis 13
2.5 Interim Analysis..15
2.6 Safety Data 15
2.6.1 Definition of Adverse Events (AEs)..15

2.6.2 Definition of Serious Adverse Events (SAEs) 16
2.7 Extraction of per-pregnancy data 16
2.8 Individual patient data meta-analysis (IPD) 16
3.0 Additional Methodological Details....17
3.1 Statistical Tests....17
3.2 Software to be Used....17
4. Appendix 18
4.1 CLIP country trials Sample Size Calculations 18
4.2 CLIP IPD Meta-analysis Power Calculations 20
4.3 Withdrawal....20
4.4 The Geo-Temporal analysis....20
4.5 Health Economics – Plan of analysis 21

Appended in the 'SAP Tables' document are the following

Table 1 Design of the Pilot Trial
Table 2 Design of the Definitive Trial
Table 3 Data fields used in the analysis of baseline and demographic variables
Table 4 Baseline outcome variables collected, by country
Table 5Decision rule for POM acceptance of referral
Table 6 Data fields used in the analysis of POM acceptance of referral
Table 7 Data fields used in the analysis of primary outcome
Table 8 Data fields used in the analysis of secondary outcomes
Table 9 Data fields used in the analysis of other outcomes
Table 10 Data fields used in the analysis of adverse events

1. Introduction

1.1 Study Description

A cluster randomised trial designed to evaluate the implementation of community-level evidence-based care aimed at reducing pre-eclampsia-related maternal and perinatal mortality and major morbidity.

The CLIP intervention consists of the following three components:

(i) **Community engagement** including community leaders, the women of the communities themselves, and their mothers, husbands, and mothers-in-law, regarding pre-eclampsia, its origins, symptoms, signs, and potential consequences, pre-permissions for maternal transport, and microfinancing activities around transport and treatment costs;

(ii) Provision of hypertensive disorders of pregnancy (HDP) oriented antenatal care through **CLIP visits** and use of CLIP PIERS On the Move (POM) tool for risk stratification. Community-based health care providers (cHCPs) will assess pregnant women with a target frequency of every 4 weeks at a minimum, and according to protocol. These visits can occur in the home or primary health centre (PHC) as both are considered part of the community for the purpose of the CLIP Trial. They will be trained to enquire about women's symptoms (using country-specific pictograms), take women's blood pressure (BP) (using systolic blood pressure (sBP), check urine for protein using dipstick and measure SpO2 (in Pakistan and Mozambique) on the first visit or on any subsequent visits for sBP ≥140 mmHg. This will inform the diagnosis and risk assessment of women with pre-eclampsia.

(iii) Use of the **CLIP 'package of care' for women with a CLIP 'trigger'** as indicated on the POM tool (i.e., oral antihypertensive therapy when indicated, intramuscular (i.m.) MgSO4 when indicated; and appropriate referral to an comprehensive emergency obstetric care (CEmOC) facility when indicated).

Facility enhancement and health worker training will be conducted in both intervention and control clusters. This activity is designed to enhance skills and promote evidence-based pre-eclampsia identification and management so that women sent to facility receive quality care.

The trial was designed to occur in two phases - a Pilot phase and a Definitive phase. The Pilot phase was designed to show that the intervention was feasible, and the data for the pilot phase form part of the definitive phase analysis. As shown in Tables 1 and 2, the Pilot trial involved four clusters per country, and the number of clusters was expanded in the Definitive phase. Surveillance data from the Pilot Trial was not analysed or reviewed and no outcomes were reviewed until after the end of the Definitive Trial. The trial protocol has been registered on clinicaltrials.gov with trial identifier NCT01911494.

1.1.1 Update to CLIP Nigeria

The CLIP Nigeria trial was originally designed to be undertaken in Ogun State, Nigeria as a pilot and then definitive cRCT, as in the three other CLIP countries. However, challenges with data collection and data entry prompted the decision to change the research design in Nigeria to one of process evaluation. To complement the effectiveness trials in Mozambique, India, and Pakistan, the process evaluation in Nigeria will serve to assure policy makers in Nigeria that the CLIP intervention can be undertaken successfully and is supported by both the community at large and health workers at various levels. The process evaluation in Nigeria will involve data collected electronically on the POM mobile health device. Excluded from analysis will be data that were initially collected on paper and then entered electronically,

as data entry was unreliable; these data involve the baseline household survey (feasibility study pre-pilot trial) and both pregnancy registration and trial surveillance (as part of the pilot and definitive trials).

1.2 Trial Objectives

Primary Objectives

- Pilot Trial (for each of Nigeria, Pakistan and India)– To implement the use of the CLIP 'package of care' and to observe at a minimum 50% acceptance of referral [urgent or non-urgent, to a facility able to provide CEmOC (Comprehensive Emergency Obstetric Care)] in women with a CLIP trigger
- Definitive Trial (for each of Mozambique, Pakistan and India) – To reduce pre-eclampsia-related, and all-cause, maternal and perinatal mortality and major morbidity by 20% or more in intervention clusters compared with control clusters.
- IPD meta-analysis (combining results from Mozambique, Pakistan and India)-
    - Maternal mortality & morbidity (primary outcome for the IPD meta-analysis): reduction by ≥ 20%
    - Maternal and fetal/neonatal mortality & morbidity: reduction by ≥ 20%
    - Maternal mortality: reduction by ≥30%

More details are in section 2.8 and power calculations for the IPD meta-analysis are in Appendix section 4.3

Secondary Objectives

Definitive Trial – (1) To measure the impact of the CLIP intervention on birth preparedness and complication readiness, the proportion of women who present for care at a CEmOC, and the proportion of women who deliver in facility.

1.3 Sample size calculations

Each country is independently powered to assess the chosen primary outcome. The data upon which the estimates have been made are routinely at the conservative end of the published or available range, and were generally provided by the site investigators. Sample size calculation for the primary outcome can be seen in Appendix 4.1. However, these calculations were revisited as planned originally, after data from the CLIP Feasibility Study and CLIP Pilot maternal and perinatal morbidity and mortality data were collected.

This review conducted independently by the Statistical Team provided insight that neonatal mortality rate in baseline varied greatly across clusters. At this point, it was also suggested by the Statistical team that adding more clusters would increase statistical power- however, this was not deemed feasible. A suggestion was then made by the statistical team to add neonatal mortality rate as a cluster level adjustment factor. More details on adjustment factors are in Section 2.1.2.

2.0 Data summaries and analyses

Participants will be all pregnant women who consent to CLIP data collection, are of reproductive age (i.e., 15-49 yr in Pakistan and India, and 12-49 in Mozambique), and are identified by the community health care providers (cHCPs) assigned to their community. Data will be analysed and reported separately for each country; an individual patient level (IPD) meta-analysis combining data from all countries will be conducted following completion of all individual trials (see Section 2.7). All data will be presented by intervention and control clusters unless otherwise specified, and for all countries combined. Section 2.3.2 outlines subgroup analyses that will include within-country analysis.

2.1 Trial information and patient disposition

2.1.1. Consort diagram

The following information will be presented:

- N women enrolled, as defined by consent to participate in trial surveillance.
- N women who withdrew
- N women who were lost to follow-up
- N women who were still on follow-up at the end of the trial
- N women who were followed up postpartum

These categories will be defined as follows:

- **Withdrawal** (from trial surveillance) **-** captured from the withdrawal log on REDCap and Monthly Reports, identifying women who have withdrawn from data surveillance. This is further described in appendix 4.3
- **Lost to follow-up** - women without trial surveillance, who were > 6 weeks postpartum more than one surveillance cycle from the end of the trial
- **Still on follow-up**
  - o Antepartum women, **or**
  - o Women, without postpartum trial surveillance, who were ≤ 6 weeks postpartum within one surveillance cycle† from the end of the trial, **or**
- **Followed-up** (i.e., have postpartum trial surveillance)†

  † *One surveillance cycle has been defined as 3 months (as in Pakistan), with 1 added to err on the side of caution.*

2.1.2. Baseline and demographic variables

Summary statistics (mean, median, standard deviation, minimum, maximum and inter-quartile range for continuous variables; the number and percentage of patients with various levels of categorical variables) will be calculated for each country, for:

- Each of intervention and control clusters; and
- Within each of intervention and control clusters, for women who were (i) lost to follow-up, (ii) still on follow-up at the end of the trial, and (iii) followed up. This analysis will enable us to see how women who were followed up may have differed from women who were not.

Adjustment will be undertaken to improve precision. Adjustment for individual level variables is likely to have less impact on the results. However, we will also include these variables to maintain consistency with how cluster level adjustment variables are being handled. The planned **adjustment variables** are at both individual and cluster-levels, in addition to any other characteristics that differ between groups at the individual and cluster level, as follows:

Individual level* (N=3): maternal age(as a continuous variable),* ***parity****(nulliparous/multiparous***),*** *and and* ***maternal basic education (****defined in Mozambique as attainment of Grade 5 or above, and in Pakistan as completion of 5 years or more of schooling and in India as 8 years or more of schooling. There is no formal*

*definition of literacy that can be applied across all settings, however, primary schooling was defined based on the country specific information provided by the site investigators)***.**

The following were considered by the committee and rejected for use:

- 'Distance from facility' (defined as primary health centre, PHC) - Average distance would have to be modelled 'as the crow flies' (i.e., using straight lines from home to PHC) for India and Pakistan because there, more precise travel distances based on the actual road network cannot be calculated because of the lack of accessibility of street datasets.
- Gestational age at enrolment - Women in intervention clusters were exposed to the intervention (community engagement) and may have booked earlier for antenatal care. By adjusting for gestational age at enrolment, we may adjust for treatment effect. It is also assumed that if women (in intervention clusters) enrol in the trial earlier, we may be able to capture greater number of outcomes such as miscarriages.
- Maternal past medical history of hypertension or seizures/epilepsy

Cluster level* (N=2): population density(population of cluster/unit size of cluster***) and the 'baseline' neonatal mortality rate (***from the baseline household survey)*

The following were considered by rejected for use:

- The poverty index as a measure of SES is no longer being considered, as BPL card in India is not considered a reliable measure of SES.
- cHCP density is no longer being considered.

Although baseline neonatal mortality rates were not considered in the design of either the original pilot or definitive trials, these rates were found to vary substantially across clusters in the analysis of the data gathered in the Feasibility Study. It was felt that their inclusion as an adjustment variable in the final analysis would substantially improve the power to detect a treatment effect. Without knowledge of the pilot trial results, the CLIP trial steering group elected to include based neonatal mortality from the pilot trial as an adjustment in the primary analysis.

2.2 Primary analysis

All analyses would be intention to treat. All statistical tests will be two-sided, with significance levels of < 0.05 for the primary outcome, and <0.01 for secondary and other outcomes. Comparisons will be by means of risk ratios and mean differences with 95% confidence intervals for each site.

2.2.1 Pilot Trials

At the end of the specified Pilot period per country (with the exception of Mozambique), the Piers On the Move data from intervention clusters was analysed for completed pregnancies to calculate referral acceptance. Surveillance data was not reviewed for intervention and control clusters except by statistical teams.

Referral acceptance was determined based on confirmation at a follow-up visit that the woman received treatment and/or went to facility. The concordance between these initial and follow-up compliance responses will be used to impute compliance for women for whom there was no follow-up visit. A minimum 50% acceptance of referral [urgent or non-urgent, to a facility able to provide CEmOC (Comprehensive Emergency Obstetric Care)] in women with a CLIP trigger was used to assess the transition of the study site from the Pilot to Definitive Trial.

These process data were shared with the CLIP Steering Committee to assess the progression of country from the Pilot to Definitive Trial. No outcome data from either intervention and control arms would be reviewed until after the end of the Definitive Trial. The pregnancies included in the Pilot trial were carried over into the Definitive Trial and will be analysed for primary outcome at the end of the Definitive Trial.

2.2.2 Definitive Trials

All analyses will be based on intention-to-treat principles. The primary outcome is the proportion of pregnancies resulting in at least one of maternal death, stillbirth, neonatal death, or severe morbidity in the mother or child.

Multiple imputation by chained equations (MICE) will be used to fill missing values, for both explanatory and outcome variables, before regression modeling of outcomes. Imputation will be undertaken for the composite primary outcome and then for each category of the primary outcome (i.e. maternal mortality, maternal morbidity, neonatal mortality and neonatal morbidity). This assumes that missing values are missing completely at random, that is, there are no systematic differences between the missing values and the observed values. Imputation will be repeated at least ten times, and outcome rates will be modeled in each of the derived dataset.

A hierarchical logistic regression model will be used to take into account the clustering of women within the units of randomisation. The model will adjust for key determinants of outcome, pre-specified by the investigators, at both the individual and cluster level.

Imputation will be based on adjustment variables (both individual and cluster level) and will include a term to account for any potential interaction between intervention clusters and time of enrolment (i.e. recruitment within the first 12 months of the trial vs. last 12 months).

2.2.2.1 Definition of the primary outcome

Maternal death (defined as the number of deaths during pregnancy or within 42 days of delivery (or the last postpartum contact day if contact not maintained to 42 days postpartum) / 1,000 identified pregnancies)

Maternal morbidity (defined as the number of women with one or more life-threatening complications of pregnancy during pregnancy or within 42 days of delivery or last postpartum contact day if contact not maintained to 42 days) / 1,000 identified pregnancies). These are the serious end-organ complications of pre-eclampsia, other major causes of maternal mortality, or life-saving interventions related to one of the aforementioned:

Serious end-organ complication of pre-eclampsia:

- Eclampsia: occurrence of generalised convulsions during pregnancy, labour or within 42 days of delivery in the absence of epilepsy or another condition predisposing to convulsions
- Stroke: hemiparesis and/or blindness developed during pregnancy or in the 42 days postpartum lasting greater than 48 hours
- Coma: prolonged unconsciousness ≥12 hours
- Antepartum haemorrhage: vaginal bleeding ≥ 15 mL with or without pain before the onset of labour
- Disseminated intravascular coagulation (DIC): abnormal bleeding from mucosa(mouth and/or ears)

Other major causes of maternal mortality:

- Obstetric sepsis: In the community, defined as fever and one of: abdominal/uterine tenderness, foul smelling vaginal discharge/lochia, productive cough and shortness of breath, dysuria or flank pain, headache and neck stiffness. In the facility, defined as presence of fever (>38°C), a confirmed or suspected infection (e.g., chorioamnionitis, septic abortion, endometritis, pneumonia) and at least one of the following: heart rate >90/min, respiratory rate >20/min, leukopoenia (total leukocyte count [TLC] <4 x 10^9/L) or leukocytosis (TLC >12 x 10^9/L)
- Vesicovaginal or rectovaginal fistula: continuous loss of urine and/or faeces after delivery

Life-saving interventions:

- Cardiopulmonary resuscitation: a set of emergency procedures including chest compressions and lung ventilation applied in cardiac arrest victims
- Dialysis: haemodialysis and/or peritoneal dialysis
- Mechanical ventilation (other than for Caesarean delivery): intubation and ventilation not related to anaesthesia
- Blood transfusion: ≥1 unit of any type of blood product
- Interventions for major postpartum haemorrhage: brace sutures, external and internal uterine compression, anti-shock garment use, internal iliac artery ligation and/or hysterectomy with or without transfusion

Perinatal & late neonatal death (defined as stillbirth [≥20^{+0} weeks and/or ≥500g in weight], early

neonatal mortality [d 0-7 of postnatal life] and late neonatal mortality [d 8-28 of postnatal life] / 1,000 identified pregnancies)

Neonatal morbidity (defined as occurrence of a primary neonatal morbidity during d 0-28 of postnatal life / 1,000 identified pregnancies). The following are the primary neonatal morbidities:

- Feeding difficulty: Including inability to suckle normally or latch on to the mother's breast to feed even if the mother's milk is not let down
- Breathing difficulty: Including grunting and in-drawing of the abdomen under the ribs
- Seizure: Occurrence of any seizure event (fits)
- Lethargy: Baby not appearing normally wakeful after activities such as feeding or sleeping
- Coma: Not medically induced period of unconsciousness of any length
- Hypothermia: Cold to touch
- Umbilical cord infection: Characterized by discharge from and redness around the umbilical stump
- Skin infection: Any appearance of abnormally red, black, swollen and blistered skin with pus
- Bleeding: From anywhere
- Jaundice: Yellow skin and eyes
- Central nervous system related morbidity: Abnormal amount of vomiting as defined by the parents or caregiver with bulging or sunken fontanelle

2.2 2.2 Calculation of the primary outcome

All enrolled pregnancies, with the exception of those who withdrew consent from the trial, will be included in the analysis of primary outcome. For women who were 'lost to follow up' or 'still on follow up' at the end of the trial, the primary composite outcome data will be imputed using a mixed imputation based that takes into account the risk associated with each woman depending on her personal characteristics (from pregnancy registration), cluster characteristics, and time of enrollment relative to the beginning of the trial.

2.2.2.3 Analysis of the primary outcome

The data fields used in the analysis of primary outcome composite is table 7, and primary outcome is calculated from the components using the following rules:

Primary outcome occurred: If ANY of the individual components were answered 'yes' (even if some components were missing answers). Define which components were missing, and the N (%) women who had one, two, three, etc, of the components missing.

Primary outcome did not occur: If none of the components were answered 'yes', and, ANY of the components was answered 'no' (even if some components were missing answers). Define which components were missing, and the N (%) women who had one, two, three, etc, of the components missing. Our assumptions can be checked by comparing patterns of responses among women who had complete and incomplete response sets.

Primary outcome is missing: If NONE of the components was completed as 'yes' or 'no' (only 'unknown' and missing were recorded). This women will be included in the imputation.

Denominator for the primary outcome rate will be all women included in the analysis (primary outcome occurred, primary outcome did not occur, and primary outcome missing).

These rules will also be used for the calculation of all other composite variables. Section 2.3.3 shows primary outcome and its components.

2.2.3 Sensitivity analyses

2.2.3.1 Completed pregnancies with postpartum follow-up:*This will assess the impact of imputation*. We will repeat the comparison between intervention and control groups using only cases with postpartum follow-up. This will involve no imputation.

2.2.3.2 Complete postpartum follow-up (for all components of primary outcome):*This will assess the impact of our assumptions about missing components of the primary outcome*. Pregnancies for whom we have complete outcome data (i.e., all questions were answered for the primary outcome). This will involve no imputation. However, if this is significant, then we will consider repeating 2.2.3.1 with the imputation based only for women with 42 day postpartum follow-up.

2.2.3.3 Women whose EDD+3 weeks falls within the trial timeline: *This will assess the impact of the intervention independent of gestational age at birth*. This includes women for whom the primary outcome was imputed.

2.2.3.4 Women whose EDD+3 weeks plus 42 day follow up period falls within the trial timeline: *This will assess the impact of incomplete postpartum follow-up that was <42 days postpartum*. This will involve all women for whom the primary outcome was imputed.

2.2.3.5 Without adjustment:*This will assess the impact of adjustment.* Sensitivity analyses without adjustment at either the cluster or individual levels will be conducted. This will involve all women for whom the primary outcome was imputed.

2.2.3.6 Inclusion of women enrolled postpartum *This will assess the impact of postpartum enrollment of women.* Such women in the intervention arm may have been exposed to community engagement, however will not have CLIP antenatal visits guided by POM application.

Note to file: This change was made when it was recognised that women were also enrolled postpartum in Mozambique because of six-monthly surveillance cycles and cultural barriers (such as late disclosure of pregnancy).

2.2.3.7 Evaluating 'on treatment' effect: An evaluation of treatment effect will be undertaken comparing women with one or more CLIP visits in intervention arm to women in the control arm. Assessment of the effect of the penetration of the intervention in terms of intensity of community engagement and of the CLIP visits will take place in the process evaluation within the intervention clusters. We will be unable to look at the third aspect of the intervention (i.e., effect of compliance with treatment recommendations) given small numbers.

2.3. Application of the intervention

Application of the intervention and challenges

This analysis is justifiable because of challenges with application of the intervention. The following criteria may be used to define differing degrees of compliance:

A. Community Engagement – There is no clear acceptable value for the measures of community engagement. As such, we will use the following measures to rate the degree of community engagement, according to 'higher' community engagement (i.e., median or higher event rate or value) or 'lower' community engagement (i.e., below median event rate or value) in intervention clusters:

   a. N (%) meetings held
   b. N (%) meetings at which each key individual topic was covered
   c. N attendees (i.e., large/small meetings)
   d. N meetings that included male decision-makers
   e. N meetings that included community leaders (male or female)

B. CLIP Visits and POM-guided management

Analyses will be done according to:

- N (%) of women who received a CLIP POM visit(s)
- N (%) women (of all those in the clusters who received CLIP POM visits at least once every 4 weeks and once postpartum, as specified in the study protocol
- N (%) women who accepted (as defined in Section 2.2.1) the POM-guided transport/treatment recommendation compared with those who were not, among women who were given a recommendation

Referral acceptance was determined based on confirmation at a follow-up visit that the woman received treatment and/or went to facility. The concordance between these initial and follow-up compliance responses will be used to describe compliance for women for whom there was no follow-up visit. Decision rule for POM acceptance of referral is shown in Table 5, data fields used in the analysis of POM acceptance of referral are shown in table 6.

Withdrawal from surveillance is described in Appendix 4.3.

2.4Secondary Analyses

2.4.1 Secondary outcomes

The secondary outcomes have been defined to measure the impact of the CLIP intervention on the delays around triage and transport.

(1) Birth preparedness and complication readiness, will assess the success of community engagement, and be measured by any *three* of the following, measured antenatally as close to delivery as available*:

(1) arranged for transport;

(2) obtained prior permission for transport should emergency arise;

(3) saved money for obstetric care;

(4) identified skilled birth attendant;

(5) identified facility for delivery.

**Any measurements taken postpartum will be disregarded*.

(2) Proportion of women presenting for care at a CEmOC facility and proportion of facility births: (This number should increase if triage (using the miniPIERS risk stratification and POM) is effective, and if there is compliance with transport to CEmOC facility.)

Methods applied to the individual components of the primary outcome will be used for the individual components of the Secondary outcome. In addition, the proportion of women achieving birth preparedness and complication readiness will be compared between intervention and control clusters, as will be the proportion of women presenting for care at anCEmOC facility, and the proportion of facility births.

Table 8 shows the data fields used in the analysis of secondary outcomes.

2.4.2 Other Outcomes

- Individual components of the primary outcome, including N seizures (overall and either pre-CLIP visit or post-CLIP visit , pre- or post- MgSO4 administration where applicable), maternal death or morbidity timing relative to administration of antihypertensive therapy for severe hypertension
- Adverse events (see 2.6.1 below)
- Knowledge of pre-eclampsia/eclampsia
- Total number of: antenatal care sought, N blood pressure measurements performed by health professionals, antihypertensive therapy received
- Gestational age at delivery
- Functional disability, which is defined as the inability to perform usual daily duties at specific points in time during the postpartum period. These are defined to reflect what would be acceptable and expected culturally in each country. The normative ranges will be defined by data from the control clusters (We hypothesise that a threshold of 90th centile would be reflect return to functional ability in each of the sites, however this would be determined from the data)
- Other perinatal morbidities: neonatal fever, congenital anomaly and birth injury.

2.4.3 Other Planned secondary analysis

- Cost-effectiveness of the CLIP intervention (See Appendix 4.5 for details)
- Geo-temporal analysis (MOMI) (See Appendix 4.4 for details)

We will analyse the following subgroups by country and within each of the three countries, by cluster:

- Country
  - **Cultural beliefs and practices** (alternative medicine, religious beliefs, beliefs about the medical system)
- Clusters
  - **Size** (large vs. small) - The size of the clusters would be obtained from national census data
  - **Geographical/seasonal challenges** (e.g. rivers)
- Women

- Parity (nulliparous vs. parous) - This analysis would be ascertained by evaluating parity (defined as deliveries at ≥20 wk and/or ≥500g)
- **Literacy** (literate vs. illiterate)
- **Distance that women live from the PHC** (close vs. far) - PHC Distance to the PHC may have to be ascertained using GIS mapping and analysis. This would limit this analysis to the time period of Definitive Trial and would be possible in two countries: Pakistan and Mozambique
- **Poverty index**
- **Gestational age at delivery**

- Community
    - **Community engagement**
        - **High vs. low** (defined as N meetings, N attendees/meeting, N topics covered)
    - **Community health workers**
        - Baseline level of education (defined as the entry requirement for the relevant training programme)
        - Intensity of initial training
        - Attendance at updates
    - **CLIP visits**
        - **Where the visits were conducted** (PHC vs. in the home)
        - **Intensity of CLIP visits**, measure of 'compliance' with the protocol, defined as women who had prescribed N antenatal visits based on gestational age at enrollment and delivery (vs. those who did not), and women who had prescribed N postnatal visits (vs. those who did not) - OR defined by quartiles of N visits/N weeks between enrollment and delivery.
        - **Clinical compliance** with CLIP recommendations for referral
            - Women who went to facility vs. those who did not
            - Women who were referred back to the community vs. those who were not
        - **Transport** (by ambulance, private car/taxi, other transport, walking)
- Health care system
    - **Density of community health care provider per population** – defined as the N population covered by each cHCP accounting for the new cHCP hired for CLIP
    - **Quality of care at PHC** - N health care providers at PHC-level
    - **Quality of care at referral facilities** - N attendees (total) at CPD events at referral facilities (total), N (%)

- Time (later vs. earlier)
    - **Definitive vs. pilot trials** (which would exclude Mozambique)
    - **Quartiles of study period**
    - **Temporal trends** (observed/expected)

2.5 Interim Analysis

An interim analysis is planned for each of the three sites once complete data (until 42 days postpartum) have been received for women making up half of the planned sample size for that country. In the event that the site is unable to reach planned sample size, due to logistical and pragmatic challenges, then the interim analysis will be conducted once complete pregnancies (until 42 days postpartum) are expected for women making up half of the projected sample size for that country. Projection of sample size for each country will be based on the observed recruitment rates in the Definitive trial as follows: 12 months of recruitment in Pakistan, 15 months of recruitment in India, and 11 months of recruitment in Mozambique. The average monthly recruitment will be calculated and applied to the remaining months of the trial, to obtain the final projected sample size. The interim analysis is reviewed by the Data Safety and Monitoring Board (DSMB).The stopping rule for both benefit and harm will require an observed difference between groups associated with an alpha <0.001. Reporting and handling of adverse events will be in accordance with Good Clinical Practice (GCP) guidelines.

2.6 Safety Data

Adverse events (AE) and Serious adverse events (SAE) will be compared between intervention and control clusters. Analysis will be for the composite (one/more of the events), as well as for each of the events.

2.6.1 Definition of Adverse Events (AEs)

- Following methyldopa administration in the community, a sBP<110mmHg on arrival at facility (maternal hypotension)
    - All cases of hypotension (reported in facility from community treatment in an intervention cluster) will be pulled and cross referenced with methyldopa administration in the same women to confirm if this AE was related to the treatment
- Following $MgSO_4$ administration in the community, either respiratory depression, coma or death during transport, as diagnosed upon arrival at facility
- Transport-related injury (life or limb) or death during transport
- Injection site haematoma or infection (following either community or facility administration of i.m. $MgSO_4$)
- ≥ 20% of women referred to facility being sent back to their communities without follow-up (monitoring community engagement and the CLIP POM)
    - This will be measured as follows:

- Include only POM visits with referral that occurred >14 days prior
- This may be antepartum or postpartum visit
- Calculate percentage of POM visits with referral that did not result in any subsequent POM visit

2.6.2 Definition of Serious Adverse Events (SAEs)

Adverse events that meet all of the following criteria:

(i) Serious

(ii) Unexpected (in nature, severity, or frequency), and

(iii) Thought to be related to the study intervention

2.7 Extraction of per-pregnancy data

Trial participants may provide data through multiple surveys. To analyze data on a per-pregnancy level, the following rules will be used to extract the data per pregnancy for analysis:

- For variables that record the presence ("Yes") or absence ("No") of a condition, the analyzed value will be "Yes" if this was answered in any of the forms, "No" if this and/or "unknown" was the only answer in any of the forms, and lastly "unknown" if this was the only answer in any of the forms
    - e.g. Dialysis (Yes/No/Unknown)
- For variables that record a choice out of a list of equally weighted options, and there can only be one answer per woman, the analysed value will be the last value entered
    - e.g. Delivery location (Home/Facility/On route/Other)
- For variables that record a choice out of a list of equally weighted options, and there can multiple answers per woman, each survey response will be analysed
    - e.g. Type of facility visited during the most recent admission
- For variables that record a numeric value, and there can only be one answer per woman, the analysed value will be the last value entered
    - e.g. Age
- For variables that record a frequency value, that is recorded at each survey, the analysed value will be the summation of all values entered
    - e.g. Number of visits to hospital

2.8 Individual patient data meta-analysis (IPD)

At the completion of all three trials (Mozambique, Pakistan and India), an individual patient data (IPD) meta-analysis will be conducted (at UBC). For the IPD meta-analysis, we will analyse data for women recruited at ≥20 weeks, to standardise data to the latest public declaration of pregnancy (i.e., 20 weeks in Pakistan). If baseline outcome data are available and consistent across all three countries, these data will be used for adjustment in analysis. For power calculation, see Appendix section 4.2.

3.0 Additional Methodological Details

3.1 Statistical Tests

Outcome rates will be compared using measures of effect size (difference in event rates, relative risks, etc) with 95% confidence intervals. Where needed, statistical tests will be two-sided, with significance levels of 0.05 for primary outcome, and 0.001 for secondary and other outcomes, unless stated otherwise.

The population standardized risk difference will be presented as an alternate summary of the benefit of the intervention rather than as a primary analytic endpoint.

3.2 Software to be Used

SAS / R.

4. Appendix

4.1 CLIP country trials Sample Size Calculations

Country	Mozambique	Pakistan	India
Unit of randomization	AP	Union Council	PHC
Population per unit of randomization	25,000	32,000	27,000
Annual birth rate (/1000/yr)	40	14	22
Births/2yr	2000	900	1200
MMR (/100,000 live births)	388	267	150
Intra-cluster co-efficient	0.002‡	0.002*	0.001**
Incidence of maternal & perinatal/neonatal M&M<br>control clusters<br>intervention clusters	 14.00%<br>11.10%	 9.60%<br>7.70%	 5.40%<br>4.30%
Number of clusters (total)	12	20	12
Number of births in Definitive CLIP Trial<br>(2 years)	24,000	18,000	14,400
Additional births from Pilot CLIP Trial<br>(4 clusters/1 year)	0	1800	2400
Total number of births<br>(Pilot & Definitive Trials)	24,000	19,800	16,800
Expected referrals at month 6 of Pilot Trial assuming 5% incidence of HDP	100 [81, 119]	45 [32, 58]	60 [45, 75]
Number of women to be referred at month 6 of Pilot Trial to meet 50% use target	50	23	30
Number of health facilities (total)<br>PHC/RHC<br>EmOC	56<br>10 BEmoc<br>1 CEmoc	 42	 22<br>22

**Number of cHCPs (total)**	135 APEs (71 prior to study start)	400 LHWs	260<br>(215 ASHAs, 45 ANMs)

ANM: assistant nurse midwife; **AM: Administrative Posts; ASHA**: accredited social health activist; **CHEW**: community health extension worker; **cHCPs**: community health care providers; **CHW** community health worker; **EmOC**, emergency obstetric care; **LGA**: Local Government Area; **LHW**: Lady Health Worker; **M&M**: morbidity and mortality; **MMR**: maternal mortality ratio; **PHC**: Primary Health Centre; **RHC**, rural health centre.

* calculated from Sindhi miniPIERS facility data; ‡ assumes same ICC as Sindh (urban population in Maputo); ** assumes half the ICC of Sindh. In these calculations, we have used risks at the lower end of the published ranges for that risk and milieu.

Assumptions:

- 10% loss of individual women to follow-up
- No loss of clusters to follow-up
- Alpha of 0.05, power ≥0.80
- Anticipated effect size of a 20% reduction in all cause maternal and perinatal/neonatal morbidity and mortality
- Also, we have assumed a ratio of maternal morbidity : mortality of 5:1 (may be as high as 10:1), and a ratio of perinatal/neonatal : maternal events of 5:1 (may be as high as 10:1 – permits overlap in outcomes)
- All sample sizes will be supplemented by the data collected in each single country Pilot CLIP Trial.

4.2 CLIP IPD Meta-analysis Power Calculations

The combined Definitive CLIP Trials (Mozambique, Pakistan and India) cohort of 60,600 deliveries in 44 clusters over 2 years, averaging 1377 deliveries per cluster per year, will provide the following power for the IPD meta-analysis:

- CLIP Definitive Trials Primary Outcome (Maternal & fetal/neonatal mortality & morbidity): assuming a 20% effect size (incidence reduced from 10.2% to 8.2%), we would have 80% power with an ICC of 0.006.
- Maternal mortality & morbidity (primary outcome for the IPD meta-analysis): assuming a 20% effect size (incidence reduced from 1.7% to 1.4%), we would have 60% power with an ICC of 0.001. To achieve 80% power will require an ICC of <0.001 which is unlikely.
- Maternal mortality: assuming a 30% effect size (incidence reduced from 1.7% to 1.2%), we would have 80% power with an ICC of 0.002. We hope to reduce the adverse maternal event rate by ≥30%, primarily through community mobilisation and antenatal care by cHCPs.

4.3 Withdrawal

- **Mozambique:** a woman who has received a Form D (pregnancy registration), who rejects further trial surveillance (Form E). In intervention clusters, there is then consent for POM and the package of care in response to a CLIP trigger, should one develop.
- **India:** Withdrawal is defined as a woman with a MN01 who declines having MN02. In intervention clusters, there is then consent for POM and the package of care in response to a CLIP trigger, should one develop.
- **Pakistan**: Withdrawal is defined as a woman with pregnancy registration who declines to have trial surveillance (antepartum/postpartum). In intervention clusters, there is then consent for POM, and then should a CLIP trigger occur, consent for the package of care

4.4 The Geo-Temporal analysis

The Geo-Temporal analysis will serve to; 1) elucidate the spatial variation in community level risk for adverse maternal outcomes, 2) Identify the factors that promote maternal resilience in the different communities under study, and 3) illustrate changing rates of adverse outcomes through the timeline of the CLIP trials. The population level estimates of variables of interest will be calculated using data from the surveillance cycles, while community rates of adverse outcomes will be calculated by combining the maternal deaths and severe morbidities recorded through facility surveillance. Geographically Weighted Regression (GWR) will be used showcase the spatial variation in associations between the choice variables, and rates of adverse maternal outcomes. Communities with less than expected rates of adverse outcomes, based on their community characteristics, will be further assessed using GWR against the predetermined community resilience variables to identify the factors that are associated with maternal resilience. Key place specific factors promoting maternal resilience will we weighted and combined into an index measuring maternal resilience. Summaries of predetermined indicators of both primary and secondary outcomes of the trial will be calculated and mapped using the ArcGIS software on a quarterly basis for the entire timeline of the trial data. This will enable the creation of geo-temporal visualizations and data indicating the changing rates of outcomes throughout the timeline of the trial.

4.5 Health Economics – Plan of analysis

Parameter estimates for costs and effectiveness will be derived from the CLIP surveillance questionnaire. The unit costs will be multiplied by identified health resource utilization to calculate the total cost per pregnancy, including both pregnant woman and newborns. The total cost will be calculated as the sum of the health resource utilization cost, cost of implementing the CLIP package of care, cost of routine pregnancy care and societal costs. The annual equivalent costs in local currency of selected CLIP countries will be converted to US dollar. The pregnancy outcomes (i.e. health of mother and baby) will be modelled as the effectiveness of the CLIP interventions. This will include no-adverse outcomes (healthy mother and newborn at the time of delivery) and adverse outcomes (death and/or disability of mother and baby) observed in the intervention and control groups. Using the data and parameter estimates specific to each CLIP country, the incremental cost-effectiveness ratios (ICER)s will be calculated first from a healthcare system perspective and then from a societal perspective. The country-specific ICER will be calculated as incremental cost per adverse pregnancy outcome. Comparison will be made between the country-specific ICER and the per capita value for the gross national income for the year 2015.

Given the uncertainties involved in CEA, we will use probabilistic sensitivity analysis to produce cost-effectiveness plots. The confidence region surrounding the cost-effectiveness ratio will be estimated using appropriate statistical methods, including bootstrap and Monte Carlo simulations. Life tables based on data from the World Health Organization's Southeast Asia and African regions or the West level-26 model will also be used in a sensitivity analysis. Country-specific health system budget impact analysis will be conducted to facilitate policy decisions for resource allocation.

Our primary analysis for this study will be model based, guided by previous work in high-income countries as no LMIC modelling in pre-eclampsia has been done. We will use parameter estimates for costs and effectiveness coming from the CLIP Trial (see Figure1). The unit costs will be multiplied by identified health resource utilization to calculate the total cost per pregnancy, including both pregnant woman and newborns. The total cost will be calculated as the sum of the health resource utilization cost, cost of implementing the CLIP package of care, cost of routine pregnancy care and societal costs. The annual equivalent costs in local currency of selected CLIP countries (PKR—Pakistani Rupee; INR—Indian Rupee, NGN—Nigerian Naira and MZN—Mozambican Metical) will be converted in US dollar exchange rate as of 2015. The pregnancy outcomes (i.e. health of mother and baby) will be modelled as the effectiveness of the CLIP interventions. This will include no-adverse outcomes (healthy mum and newborn at the time of delivery) and adverse outcomes (death and/or disability of mother and baby) observed in the intervention and control groups. Using the data and parameter estimates specific to each CLIP country, the ICERs will be calculated first from a healthcare system perspective and then from a societal perspective. The country-specific ICER will be calculated as Incremental cost per adverse pregnancy outcome. ICERs for the system perspective as the reference case will be of interest to country-specific health policy makers for resource allocation decisions, when switching from routine pregnancy care to CLIP plus routine pregnancy care, should CLIP be found effective. Critically, however, the ICER from a societal perspective will facilitate discourse on the full opportunity cost in the context of the selected CLIP country. In accord with the recommendation of the Commission for Macroeconomics and Health, we will compare the country-specific ICER with the per capita value for the gross national income of each of the four selected CLIP countries for the year 2015.

Given the uncertainties involved in CEA, we will use probabilistic sensitivity analysis to produce cost-effectiveness plots. The confidence region surrounding the cost-effectiveness ratio will be estimated

using appropriate statistical methods, including bootstrap and Monte Carlo simulations. Life tables based on data from the World Health Organization's Southeast Asia and African regions or the West level-26 model will also be used in a sensitivity analysis. Children in LMICs bear a disproportionately large share of the total disease burden, because of the cause structure of the disease burden by age could influence overall distribution of DALYs. As reported on previous cost-effectiveness studies in LMIC, no-age-weighting in the reference case was used on sensitivity analysis. Country-specific health system budget impact analysis will be conducted to facilitate policy decisions for resource allocation, when switching from routine pregnancy care to CLIP plus routine care, should the intervention be found effective.

Figure 1 (next page) Decision analytic tree model for economic evaluation of CLIP. HDP, hypertensive disorder of pregnancy

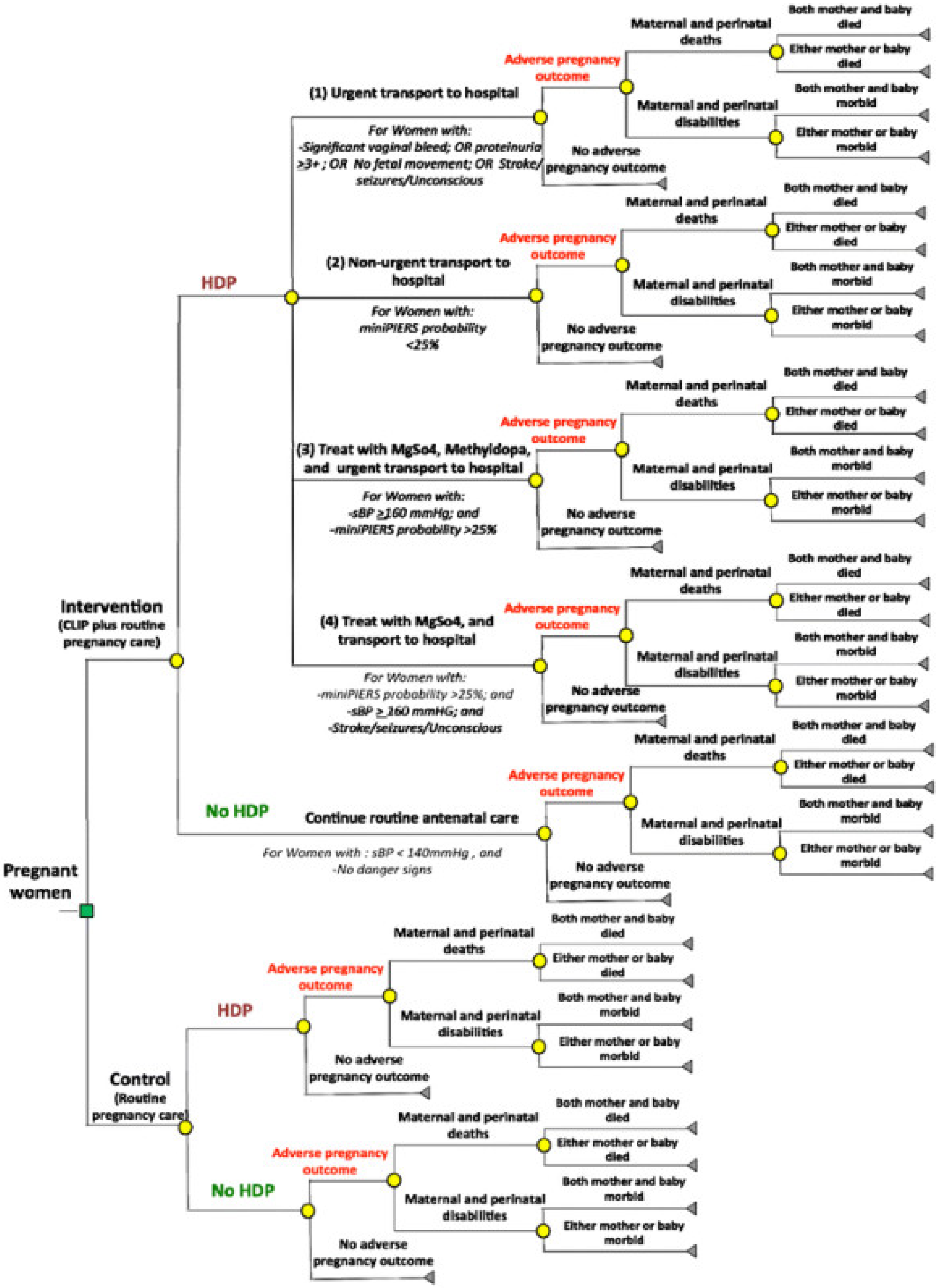

HDP = Hypertensive Disorder of Pregnancy

Tables in CLIP Statistical Analysis Plan

Table 1 Design of the Pilot Trial 1

Table 2 Design of the Definitive Trial... 2

Table 3 Data fields used in the analysis of baseline and demographic variables...................... 3

Table 4 Baseline outcome variables collected, by country 4

Table 6 Data fields used in the analysis of POM acceptance of referral.................................. 5

Table 7 Data fields used in the analysis of primary outcome.. 6

Table 8 Data fields used in the analysis of secondary outcomes 7

Table 9 Data fields used in the analysis of other outcomes.. 7

Table 10 Data fields used in the analysis of adverse events 9

Table1 Design of the CLIP Pilot Trials

	**Pakistan**<br>(Hyderabad and Matiari districts in Sindh Province)	**India**<br>(Belgaum and Bagalkot districts in Karnataka State)
**Timeline**	Feb 2014 – Dec 2014	Feb 2014 – Oct 2014
**Number of control clusters**	2	2
**Number of intervention clusters**	2	2
**Data collection**	Ongoing pregnancy registration;<br>Regular household surveys on quarterly basis;<br>Facility surveillance in public tertiary level hospitals only	Using MNH Registry with three time points of data collection:<br>Pregnancy identification;<br>Within 7 days of delivery;<br>42 days postpartum

Table 2 Design of the CLIP Definitive Trials

	**Mozambique**<br>(Maputo and Gaza Province)	**Pakistan**<br>(Hyderabad and Matiari districts in Sindh Province)	**India**<br>(Belgaum and Bagalkot districts in Karnataka State)
**Timeline**	Feb 2015 -	Mar 2015 -	Nov 2014 -
**Number of control clusters**	6	10	6
**Number of intervention clusters**	6	10	6
**Data collection**	Ongoing pregnancy registration;<br>Regular household survey every six months with ongoing facility data collection;<br>Maternal and Perinatal verbal autopsies for all deaths	Ongoing pregnancy registration;<br>Regular household surveys on quarterly basis;<br>Facility surveillance in public tertiary level hospitals only;<br>Maternal and Perinatal verbal autopsies for all deaths	Using MNH Registry with three time points of data collection:<br>Pregnancy identification;<br>Within 7 days of delivery;<br>42 days postpartum

Table 3 Data fields used in the analysis of baseline and demographic variables

CRF	Section	Field
Pregnancy registration	Demographic information	• Age<br>• Years of school<br>• Partner in house (Mozambique only)
Pregnancy registration	Obstetric history	• Parity<br>• High blood pressure<br>• Seizures
Pregnancy registration	Gestational age assessment	• EDD / LMP
Pregnancy registration	Medical history	• High blood pressure<br>• Seizures
Regular surveillance	Cost of newborn interventions / admissions	• Time to facility
Baseline survey	Household information	• Poverty index

Table 4 Baseline outcome variables collected, by country

	**Pakistan**	**Mozambique**	**India**
N MWRA (married women of reproductive age)	x	x	x
Age of MWRA	x	x	x
Number of MWRA with one or more delivery/ abortion reported in last 12 months (n(%))	x	x	x
Number of pregnancies resulting in a live birth (n(%))	x	x	x
Number of pregnancies resulting in a still birth (n(%))	x	x	x
Number of pregnancies resulting in a miscarriage (n(%))	x	x	x
Pregnancies complicated by high blood pressure (n(%))	x	x	x
Pregnancies complicated by eclampsia(n(%))	x		x
Pregnancies complicated by seizure(n(%))		x	
Pregnancies complicated by severe headache (n(%))	x		
MRWA deaths reported n(%)	x	x	x
Maternal deaths		x	x
Age of woman at death (mean +/- sd)	x	x	x
**Children deaths within 1 week (early neonatal death)**	**x**	**x**	**x**
**Children deaths between 1 week and 1 month (late neonatal death)**	**x**	**x**	**x**
Maternal morbidity			x
Blood transfusion			x
Hysterectomy			x
Antepartum haemorrhage			x
Coma			x
Failure to form clots			x
Fever & symptom			x
Headache and stiff neck			x
Cough and shortness of breath			x
Abd pain or uterine tenderness			x
Flank pain			x
Foul smelling vag discharge			x
Received Anti-shock garment			x
Received Bimanual uterine compression			x
Received Brace sutures			x
Received CPR			x
Received Dialysis			x
Received Internal iliac artery ligation/devascularisation procedure			x
Received Mechanical ventilation			x
Seizure (fits)			x
Stroke			x

Neonatal Morbidity			x
Breathing problems			x
Feeding problems			x
Lethargy			x
Coma (neonatal)			x
Seizure (neonatal)			x
Umbilical stump infection			x
Skin infection			x
Jaundice			x
Diarrhea/vomiting			x
Bleeding			x

Table 5 Decision rule for POM acceptance of referral

**Follow-up response (Did you go to hospital?):**	**Yes**			**No**		**Missing**			
**Response given immediately following recommendation:**	**Accept**	**Refuse**	**Missing**	**Accept**	**Refuse**	**Missing**	**Accept**	**Refuse**	**Missing**
Acceptance	√	√	√	X	X	X	√	X	Missing

Table 6 Data fields used in the analysis of POM acceptance of referral

CRF	Section	Field
PIERS on the Move	Visit	• Transport
PIERS on the Move	Follow-up	• Went to hospital after last home visit

Table 7 Data fields used in the analysis of primary outcome

CRF	Section	Field
Regular surveillance	Demographic information	• Deceased
Regular surveillance	Maternal conditions and interventions	• Cardiopulmonary resuscitation (CPR)<br>• Mechanical ventilation<br>• Dialysis<br>• Blood transfusion<br>• Disseminated intravascular coagulation (DIC)<br>• Antepartum haemorrhage<br>• Anti-shock garment<br>• Hysterectomy<br>• Stroke<br>• Seizure<br>• Coma<br>• Obstetric sepsis
Regular surveillance	Perinatal outcomes	• Birth outcome<br>• Death within one week of birth<br>• Death between one week and one month of age
Regular surveillance	Perinatal outcomes	• Feeding difficulty<br>• Breath difficulty<br>• Seizure<br>• Lethargy<br>• Coma<br>• Hypothermia<br>• Umbilical cord infection<br>• Skin infection<br>• Bleeding<br>• Jaundice<br>• Central nervous system related morbidity

Table 8 Data fields used in the analysis of secondary outcomes

CRF	Section	Field
Regular surveillance	Delivery information	• Location of delivery
Regular surveillance	Birth preparedness	• Where to deliver<br>• Skilled birth attendant<br>• Transportation plan<br>• Funds saved for emergencies<br>• Permission to go seek care
Regular surveillance	Care-seeking behaviour	• Care sought from health care provider<br>• Visits to primary health centres<br>• Visits to higher level facilities

Table 9 Data fields used in the analysis of other outcomes

CRF	Section	Field
Regular surveillance	Delivery information	• Location of delivery<br>• Blood pressure measured<br>• Antihypertensive medications
Regular surveillance	Pre-eclampsia knowledge	• Abnormal bleeding after delivery<br>• High blood pressure during pregnancy<br>• High blood pressure can cause death of pregnant women<br>• Seizures during pregnancy<br>• Headache<br>• Visual disturbance<br>• Chest pain<br>• Shortness of breath<br>• Nausea and vomiting<br>• Abdominal pain<br>• Vaginal bleeding

CRF	Section	Field
		• Unconsciousness<br>• Stroke<br>• Seizures<br>• Unusual swelling
Regular surveillance	Medications and adverse effects	• Received MgSO4
PIERS on the Move	Visit	• MgSO4
PIERS on the Move	Follow-up	• Followup_MgSO4<br>• Haematoma<br>• Infection
Regular surveillance	Functional disability	• Take care of newborn<br>• Wash babies' clothes<br>• Prepare meals<br>• Clean the house<br>• Get water<br>• Work in the fields<br>• Go to the market<br>• Return to work/paid employment

Table 10 Data fields used in the analysis of adverse events

CRF	Section	Field
PIERS on the Move	Visit	• Methyldopa<br>• MgSO4<br>• Visit date
PIERS on the Move	Follow-up	• Physical injury during transport<br>• Haematoma at injection site<br>• Infection at injection site
Facility Surveillance	Visit information	• Reason for seeking care<br>• Status on arrival
Facility Surveillance	Clinical characteristics on arrival	• Blood pressure
Facility Surveillance	MgSO4-related adverse effects	• Haematoma at injection site<br>• Infection at injection site

Blood pressure thresholds in pregnancy for identifying maternal and infant risk: a secondary analysis of Community-Level Interventions for Pre-eclampsia (CLIP) trial data - Appendix

CLIP Trial Statistical Analysis Plan – Summary of Changes

Statistical Analysis Plan for the CLIP trial - Summary of changes

Original version (Version 0.1 20151016) to final version (Version 0.2-20151022)

Subheading, page (in final SAP)	Change(s)	Original version (with change highlighted)
**1.1 Study Description**<br><br>**Pg. 3**	Changes made in ii) component	check urine for protein using dipstick and SpO2 (in Pakistan and Mozambique)
	Added a fourth component to CLIP intervention	(iv) Facility enhancement and health worker training to enhance skills and promote evidence-best pre-eclampsia identification and management.
**1.1 Study Description**<br><br>**Pg. 4**	Changes made within the table:	From Apr 2015 to Jan 2015<br><br>Household surveys
**1.2 Primary Objectives**<br><br>**Pg. 4**	Further clarified referral % of pilot trial	To implement the use of the CLIP 'package of care' and to observe at a minimum 50% acceptance of referral (urgent or non-urgent, to a facility able to provide CEmOC) in women with a CLIP trigger.
**1.3 Secondary Objectives**<br><br>**Definitive Trial**<br><br>**Pg. 7**	Removed a few sentences	(we are determining these normal limits through local focus group discussions; these will be defined for each country prior to the commencement of each Pilot CLIP Trial)
**2.2 Baseline and Demographic Variables**<br><br>**Pg. 8**	Changed one of the intervention variables	From route of birth to mode of delivery
**2.3.3 Per-protocol analyses**	One point added under criteria iii)	▪ Number of topics covered

**Pg. 12**		

Original version (Version 0.2-20151022) to final version (Version 0.3-20151117)

**Subheading, page (in final SAP)**	**Change(s)**	**Original version (with change highlighted)**
**1.1 Study Description**<br><br>**Pg. 3**	Removed the fourth component to CLIP intervention	
	Added more information about study design	Facility enhancement and health worker training will be conducted in both intervention and control clusters. This activity is designed to enhance skills and promote evidence-based pre-eclampsia identification and management<br><br>The trial is designed to occur in two phases, a Pilot phase and a Definitive phase. The Pilot phase will involve four clusters per country, and the Definitive phase will include all clusters, the number of which varies by country.
**The design of Definitive is as follows**<br><br>**Pg. 4**	Ongoing data collection in facilities along with surveys	Regular household survey every six months with ongoing facility data collection;
**1.1.1 Update to original protocol**<br><br>**Pg. 5**	Update to protocol:	Nigeria - we will add a section here explaining that we are approaching things differently in this country. This section will explain the difference between the published protocol and the new plan, and any implications on the countries that are continuing with the original plan.
		To reduce pre-eclampsia-related, and all-cause, maternal and perinatal mortality and major morbidity by 20% or more in

		intervention clusters compared with control clusters.
	Detail breakdown removed.	
**Definitive Trial**<br><br>**Pg. 6**	Removed the following bullet points under Definitive Trial:<br><br>-Birth preparedness and complication readiness as measured by any three of the following: (1) arranged for transport; (2) obtained prior permission for transport should emergency arise; (3) saved money for obstetric care; (4) identified skilled birth attendant; (5) identified facility for delivery. This will evaluate the success of community engagement.<br><br>-Number of women presenting for care at a CEmOC facility and number of facility births should increase if triage (using the miniPIERS risk stratification and POM) is effective, and if there is compliance with transport to CEmOC facility.<br><br>-Other outcomes will be evaluated such as knowledge of pre-eclampsia/eclampsia among women of reproductive age; total number of seizures; number of pre-admission and post-admission seizures; adverse events; functional disability; and cost-effectiveness of the CLIP intervention. In addition, other outcomes for the perinatal morbidities are neonatal fever, congenital anomaly and birth injury.<br><br>-Other outcomes also include functional disability for any reason: Inability to perform usual daily duties at specific points in time during the postpartum period that would be acceptable and expected culturally.	
**1.4 Eligibility Criteria Pg. 6**	Removed eligibility criteria	
**1. Data Summaries and Analysis**	Added some more information under data summaries and analysis	Participants will be all pregnant women who consent to CLIP data collection, are of reproductive age (il.e., 15-49 yr in Pakistan and India, and 12-49 in Mozambique), and

**Pg.7**		are identified by the community health care providers (cHCPs) assigned to their community. Data will be analysed and reported separately for each country; an individual patient level meta-analysis combining data from all countries will be conducted following completion of all individual trials (see Section 2.7). All data will be presented by intervention and control clusters unless otherwise specified, and for all countries combined. Section 2.3.2 outlines subgroup analyses that will include within-country analysis.
**2.1 Trial Information and Patient Disposition**<br><br>**Pg. 7**		• Number of POM visits completed in the intervention clusters.<br><br>• Number of referrals recommended in the intervention clusters.<br><br>• Number of referrals accepted in the intervention clusters.<br><br>• Frequency table for withdrawal and loss to follow-up, for the primary outcome and the secondary outcome.
**2.2 Baseline and Demographic Variables**<br><br>**Pg. 7**	Summary statistics will be calculated for each cluster (intervention and control)	•Cluster level variables<br><br>-cHCP density, population density<br><br>-Socioeconomic status (measured by poverty index), level of education<br><br>Individual level variables:<br><br>-Demographics: maternal age, parity, gestational age at enrolment, method of gestational age determination, maternal past medical history of hypertension or seizures/epilepsy, past obstetric history of hypertension, distance from facility.

	Removed the following from<br><br>CRF: Regular surveillance<br><br>Section: Care seeking behaviour, delivery<br><br>Field: Number of antenatal care visits, care sought from health care provider, route of delivery	
**2.3.2 Definitive Trial**<br><br>**Pg. 9**	Following additions have been made under this section:<br><br>-All analyses will be based on intention-to-treat basis.<br><br>-For pregnancies without complete follow-up and outcome data, outcomes will be determined by... ### describe imputation methods, what assumptions will be used	A hierarchical logistic regression model will be used to take into account the clustering of women within the units of randomisation. The model will adjust for key determinants of outcome, to be pre-specified by the investigators, at both the individual and cluster level. Adjustment variables currently under consideration are: maternal age, medical and obstetric history, [… complete the list] at the individual level and population density and baseline outcome rate (is this available for all countries??) at the cluster level.
**2.3.2.1 Definition of the primary outcome**<br><br>**Pg. 9-11**	Added a whole section to define the primary outcome:<br><br>**Maternal death** (defined as the number of deaths during pregnancy or within 42 days of pregnancy (or last contact day if contact not maintained to 42 days) / 1,000 identified pregnancies)<br><br>**Maternal morbidity** (defined as the number of women with one or more life-threatening complications of pregnancy during pregnancy or within 42 days of pregnancy or last contact day if contact not maintained to 42 days) / 1,000 identified pregnancies). These are the serious end-organ complications of pre-eclampsia, other major causes of maternal mortality, or life-saving	

	interventions related to one of the aforementioned:<br><br>Serious end-organ complication of pre-eclampsia:<br>▪ Eclampsia: occurrence of generalised convulsions during pregnancy, labour or within 42 days of delivery in the absence of epilepsy or another condition predisposing to convulsions<br>▪ Stroke: hemiparesis and/or blindness developed during pregnancy or in the 42 days postpartum lasting greater than 48 hours<br>▪ Coma: prolonged unconsciousness ≥12 hours<br>▪ Antepartum haemorrhage: vaginal bleeding ≥ 15 mL with or without pain before the onset of labour<br>▪ Disseminated intravascular coagulation (DIC): abnormal bleeding from mucosa(mouth and/or ears)<br><br>Other major causes of maternal mortality:<br>▪ Obstetric sepsis: In the community, defined as fever and one of: abdominal/uterine tenderness, foul smelling vaginal discharge/lochia, productive cough and shortness of breath, dysuria or flank pain, headache and neck stiffness. In the facility, defined as presence of fever (>38°C), a confirmed or suspected infection (e.g., chorioamnionitis, septic abortion, endometritis, pneumonia) and at least one of the following: heart rate >90/min, respiratory rate >20/min, leukopoenia (total leukocyte count [TLC] <4 x 10^9/L) or leukocytosis (TLC >12 x 10^9/L)<br>▪ Vesicovaginal or rectovaginal fistula: continuous loss of urine and/or faeces after delivery<br><br>Life-saving interventions:<br>▪ Cardiopulmonary resuscitation: a set of emergency procedures including chest compressions and lung ventilation applied in cardiac arrest victims<br>▪ Dialysis: haemodialysis and/or peritoneal dialysis<br>▪ Mechanical ventilation (other than for Caesarean delivery): intubation and ventilation not related to anaesthesia<br>▪ Blood transfusion: ≥1 unit of any type of blood product<br>▪ Interventions for major postpartum haemorrhage: brace sutures, external and internal uterine compression, anti-shock garment use, internal iliac artery ligation and/or hysterectomy with or without transfusion<br><br>**Perinatal & late neonatal death** (defined as stillbirth [$\geq 20^{+0}$ weeks and/or ≥500g in weight], early neonatal mortality [d 0-7 of postnatal life] and late neonatal mortality [d 8-28 of postnatal life] / 1,000 identified pregnancies)<br><br>**Neonatal morbidity** (defined as occurrence of a primary neonatal morbidity during d 0-28 of postnatal life / 1,000 identified pregnancies). The following are the primary neonatal morbidities:<br>➢ **Feeding difficulty**<br>• Including inability to suckle normally or latch on to the mother's

	breast to feed even if the mother's milk is not let down<br>➢ **Breathing difficulty**<br>• Including grunting and in-drawing of the abdomen under the ribs<br>➢ **Seizure**<br>• Occurrence of any seizure event (fits)<br>➢ **Lethargy**<br>• Baby not appearing normally wakeful after activities such as feeding or sleeping<br>➢ **Coma**<br>• Not medically induced period of unconsciousness of any length<br>➢ **Hypothermia**<br>• Cold to touch<br>➢ **Umbilical cord infection**<br>• Characterized by discharge from and redness around the umbilical stump<br>➢ **Skin infection**<br>• Any appearance of abnormally red, black, swollen and blistered skin with pus<br>➢ **Bleeding**<br>• From anywhere<br>➢ **Jaundice**<br>• Yellow skin and eyes<br>➢ **Central nervous system related morbidity**<br>• Abnormal amount of vomiting as defined by the parents or caregiver with bulging or sunken fontanelle
**2.3.2.2 Outcome of data extraction**<br><br>**Pg. 12**	Added a whole section to define outcome data extraction:<br><br>If there is information that relates to the primary outcome from more than one surveillance form, the following rules will be used to extract the final value for analysis:<br><br>• For variables that record the presence (Yes) or absence (No) of a condition, the analyzed value will be "Yes" if this was answered in any of the forms, "No" if this and/or "unknown" was the only answer in any of

	the forms, and lastly "unknown" if this was the only answer in any of the forms<br>▪ e.g. Dialysis (Yes/No/Unknown)<br><br>• For variables that record a choice out of a list of equally weighted options, the analysed value will be the last value entered<br>▪ e.g. Delivery location (Home/Facility/On route/Other)	
**2.3.3 Sensitivity analyses**<br><br>**Pg. 12**	Removed:<br><br>We will use multiple imputations to account for those lost to follow-up and examine whether this has any impact on the between-group comparisons.	Added:<br><br>We will repeat the comparison between intervention and control groups using only records with complete follow-up. This will assess the impact of imputation performed in the primary analysis.
**2.3.4 Subgroup Analysis**<br><br>**Pg. 13**	Original:<br><br>We will analyse the interventions and outcomes by country and locality. Other subgroup comparisons may include:	Revised:<br><br>We will analyse the following interventions and outcomes by country and within each of the three countries, by cluster:
**2.3.5.1**<br><br>**Application of the Intervention and Challenges**<br><br>**Pg. 13**	Added:<br><br>The section should be divided into community engagement and CLIP visits and POM-guided management. These are the issues raised in 2.3.3. We need to define them based on our collective experience and definitely before looking at the data. I suggest that we start with community engagement as that will facilitate the work in Nigeria.	
**2.3.5.2 Analysis**	Removed:	Added:

**Pg. 13**	All analyses will be based on intention-to-treat basis	Analyses for the intervention clusters will be repeated based on per-protocol basis
	Original criteria:<br><br>(i) Women who received a CLIP visit(s)<br><br>(ii) Women who received CLIP visits at least once every 4 weeks and once postpartum<br><br>(iii) Community engagement in cluster may consider factors such as:<br><br>-Engagement of household male decision-makers<br><br>-Frequent (vs less frequent) meetings<br><br>-Large (vs. small) meetings<br><br>-Engagement of community leaders (male or female)<br><br>-Completeness of the topics covered<br><br>-Number of topics covered	Revised criteria:<br><br>(i) Community engagement in cluster may consider factors such as:<br><br>- Engagement of household male decision-makers<br><br>-Frequent (vs less frequent) meetings<br><br>-Large (vs. small) meetings<br><br>-Engagement of community leaders (male or female)<br><br>-Number of topics covered<br><br>(ii) Women who received a CLIP POM visit(s)<br><br>(iii) Women who received CLIP POM visits at least once every 4 weeks and once postpartum<br><br>(iv) Women who were confirmed to have accepted the POM-guided recommendation compared with those who were not
**2.4 Secondary Analyses**<br><br>**Pg. 14**	There are three secondary outcomes:<br><br>• Birth preparedness and complication readiness<br><br>-Measured by any three of the following: (1) arranged for transport; (2) obtained prior permission for transport should emergency arise; (3) saved money for obstetric care; (4)	

	identified skilled birth attendant; (5) identified facility for delivery.<br><br>-This will evaluate the success of community engagement.<br><br>• N women who sought care at a CEmOC-enabled facility<br><br>-This will evaluable effectiveness of triage (using the miniPIERS risk stratification and POM), and compliance with transport to CEmOC facility.<br><br>• N births at a CEmOC-enabled facility<br><br>-This will evaluable effectiveness of triage (using the miniPIERS risk stratification and POM), and compliance with transport to CEmOC facility.
	Methods applied to the individual components of the Primary outcome will be used for the individual components of the Secondary outcome. In addition, the proportion of women achieving birth preparedness and complication readiness will be compared between intervention and control clusters, as will be the proportion of women presenting for care at an CEmOC facility, and the proportion of facility births.
	Removed the following from<br><br>Field: Blood pressure measured, antihypertensive medications
**2.4.2.1 Definition of other outcomes**<br><br>**Pg. 15**	Added the following:<br><br>o Knowledge of pre-eclampsia/eclampsia among women of reproductive age<br><br>o Total number of: seizures, pre-admission and post-admission seizures, MgSO4 administration, adverse events (haematoma or infection associated with MgSO4 injection)<br><br>o Total number of: antenatal care sought, blood pressure measurements performed by health professionals, antihypertensive therapy received

<table>
<tr><td></td><td colspan="2">o Functional disability, which is defined as the inability to perform usual daily duties at specific points in time during the postpartum period. These are defined to reflect what would be acceptable and expected culturally in each country

o Other perinatal morbidities: neonatal fever, congenital anomaly and birth injury.

o Cost-effectiveness of the CLIP intervention

o Geo-temporal analysis (MOMI)</td></tr>
<tr><td></td><td colspan="2">Added the following in the two rows and columns in the table:
<table><tr><td>PIERS on the Move</td><td>Visit</td><td>• MgSO4</td></tr><tr><td>PIERS on the Move</td><td>Follow-up</td><td>• Followup_MgSO4
• Haematoma
• Infection</td></tr></table></td></tr>
<tr><td>2.5 Interim Analysis

Pg. 16</td><td colspan="2">Planned for three countries instead of four.

Removed: (power:80%)</td></tr>
<tr><td>2.6 Safety Data

Pg. 17</td><td></td><td>Adverse events (AE) and Serious adverse events (SAE) will be compared between intervention and control clusters. Analysis will be for the composite (one/more of the events), as well as for each of the events.</td></tr>
<tr><td>2.6.1 Adverse Events (AEs)

Pg. 17</td><td>Removed:

• The following will be presented by treatment group: number of adverse events (overall and by type), number of women with one or</td><td>• Following methyldopa administration in the community, a sBP <110mmHg on arrival at facility (maternal hypotension)

• Following MgSO4 administration in the community, either respiratory depression, coma or death during transport, as diagnosed upon arrival at facility</td></tr>
</table>

<table>
<tr><td></td><td>more adverse event(s) (overall and by type)
<table><tr><td>Facility Surveillance</td><td>Visit information</td><td>• Reason for seeking care
• Status on arrival</td></tr><tr><td>Facility Surveillance</td><td>Clinical characteristics on arrival</td><td>• Blood pressure</td></tr><tr><td>Facility Surveillance</td><td>MgSO4-related adverse effects</td><td>• Haematoma at injection site
• Infection at injection site</td></tr></table>
Added the above two rows and columns in the table</td></tr>
<tr><td>2.6.2 Definition of Serious Adverse Events (SAEs)

Pg. 17</td><td>Added the following under this section:

Adverse events that meet all of the following criteria:

(i) Serious

(ii) Unexpected (in nature, severity, or frequency), and

(iii) Thought to be related to the study intervention</td></tr>
<tr><td>2.7 Individual patient data meta-analysis (IPD)

Pg. 18</td><td>An individual patient data (IPD) meta-analysis will be conducted (at UBC) after the completion of all three trials. For the IPD meta-analysis, we will analyse data for women recruited at ⩾20 weeks, to standardise data to the latest public declaration of pregnancy (i.e., 20 weeks in Pakistan). [Will not be able to adjust for baseline outcome rate if it is not available for every country.]</td></tr>
</table>

Original Version (Version 0.3-20151117) to final version (Version 0.3-20151118)

Subheading, page (in final SAP)	Change(s)

**1.2.2 Secondary Objectives**<br>**Pg. 6**	Deleted:<br><br>Pilot Trial<br><br>To compare pre-eclampsia related, and all-cause maternal and perinatal mortality and major morbidity in intervention versus control clusters.
**2.3.2 Definitive Trial**<br><br>**Pg. 10**	Deleted details about population density and baseline outcome rate:<br><br>(is available and consistent across countries)
	Inserted:<br><br>Table 6 Baseline outcome variables collected, by country
**2.6.1 Definition of Adverse Events (AEs)**<br><br>**Pg. 18**	Inserted:<br><br>All cases of hypotension (reported in facility from community treatment in an intervention cluster) will be pulled and cross referenced with methyldopa administration in the same women to confirm if this AE was related to the treatment.
	Inserted:<br><br>This will be measured as follows:<br><br>- Include only POM visits with referral that occurred > 14 days prior<br>- This may be antepartum or postpartum visit<br>- Calculate percentage of POM visits with referral that did not result in any subsequent POM visit
	Table 10<br><br>Added "Visit date" under the Field column

Original version (Version 0.3-20151118) to final version (Version 0.4-20160112)

**Subheading, page (in final SAP)**	**Change(s)**	**Original version (with change highlighted)**
**1.1.1 Update to CLIP Nigeria**<br><br>**Pg. 5**	Added:<br><br>The CLIP Nigeria trial was originally designed to be undertaken in Ogun State, Nigeria as a pilot and then definitive cRCT, as in the three other CLIP countries. However, challenges with data	

	collection and data entry prompted the decision to change the research design in Nigeria to one of process evaluation. The effectiveness data for the CLIP intervention will come from the three remaining cRCTs in Mozambique, India, and Pakistan; these trials will determine whether there is a causal relationship between CLIP intervention and health outcomes. To complement the effectiveness trials, the proposed evaluation in Nigeria will serve to assure policy makers that the CLIP intervention can be undertaken successfully and is supported by the community at large as well as the health workers at various levels.
**2.3.2 Definitive Trial**<br><br>**Pg. 9**	Deleted:<br><br>For pregnancies without complete follow-up and outcome data, outcomes will be determined by ###describe imputation methods, what assumptions will be used<br><br>Inserted:<br><br>For missing variables (variables associated with outcomes, or are outcomes themselves), multiple imputation may be used before regression modeling. This assumes that missing values are missing completely at random, that is, there are no systematic differences between the missing values and the observed values. Multiple imputation by chained equations (MICE) will be the method used to generate plausible values for the missing variables, where imputation will be repeated at least ten times, and outcome rates will be modeled in each of the derived dataset.
**2.3.3 Sensitive Analyses**<br><br>**Pg. 14**	We will repeat the comparison between intervention and control groups using only cases with complete follow-up. This will assess the impact of imputation performed in the primary analysis, and biases caused by data that are missing not at random may be detected here.
**Other in-depth analyses:**<br><br>**Pg. 18**	Added details about the cost-effectiveness of the CLIP Intervention.<br><br>Added Geo-temporal analysis (MOMI)

**4 Appendix**<br><br>**Pg. 22-23**	Added: Health Economics – Plan Analysis<br><br>Added: Figure 1: Decision analytic tree model for economic evaluation of CLIP, HDP, hypertensive disorder of pregnancy

Original version (Version 0.4-20160112) to final version (Version 0.5-20160113)

**Subheading, page (in final SAP)**	**Change(s)**	**Original version (with change highlighted)**
**Table 2 Design of the Definitive Trial**<br><br>**Pg. 5**	Mozambique timeline changed from Jan 2015 to Feb 2015	
**2.3.4 Subgroup analyses**<br><br>**Pg. 14**	Added to characteristics of the women:<br><br>• This analysis would be ascertained by evaluating parity (defined as deliveries at ≥20 wk and/or ≥500g)<br>o Women who live close (vs. far) from the PHC<br>Distance to the PHC may have to be ascertained using GIS mapping and analysis. This would limit this analysis to the time period of Definitive Trial and would be possible in two countries: Pakistan and Mozambique.<br>• In Pakistan and India, “literacy” is defined as “Ability to read and write in any language”<br>Maternal Level of Education will be ascertained by:<br>• Years of school completed (*for Pakistan*) - >0 number of years would be considered literate?<br>• *List (Mozambique): illiterate, grade 5, grade 7, grade 10, grade 12, Bachelor, graduate, post-graduate, not-applicable, other, don’t know*<br>• *List (India): No formal schooling, literate; no formal schooling, illiterate; DK in both versions of MNH forms (although 2011 is DK/NA). In 2011 version, “schooling” is described as: primary incomplete; secondary incomplete; university/college incomplete; primary complete; secondary complete;’ university/college complete. In 2013 version, schooling is yrs of schooling.*<br>• Characteristics of the clusters	

	o Large (vs. small) clusters<br>The size of the clusters would be obtained from national census data<br>o Density of health care provider per population<br>–We may need additional govt. data for this?<br>• Effect of time
**2.3.5.1 Application of the intervention and challenges**<br><br>**Pg. 15**	Added A. Community Engagement – table that records attendance of community engagement meetings and the topics covered<br><br>Added B. CLIP Visits and POM-guided management
**Geo-temporal analysis (MOMI)**<br><br>**Pg. 19 – 20**	Added a section to further describe the geo-temporal analysis.

Original version (Version 0.5-20160113) to final version (Version 0.5-20160126)

**Subheading, page (in final SAP)**	**Change(s)**	**Original version (with change highlighted)**
**2.3.4 Subgroup analyses**	• Country<br>o **Cultural beliefs and practices** (alternative medicine, religious beliefs, beliefs about the medical system)<br>o<br>• Clusters<br>o **Size** (large vs. small) - The size of the clusters would be obtained from national census data<br>o **Geographical/seasonal challenges** (e.g. rivers)<br><br>• Women<br>o **Parity** (primips vs. multips) - This analysis would be ascertained by evaluating parity (defined as deliveries at ≥20 wk and/or ≥500g)<br>o **Literacy** (literate vs. illiterate) - In Pakistan and India, "literacy" is defined as "Ability to read and write in any language"[SS1]<br>o Level of education for mother (none, some/completed primary, some secondary or more)<br>o Level of education for partner (none, some/completed primary, some secondary or more)<br>▪ Maternal Level of Education will be ascertained by: Years of schoolcompleted (for Pakistan) - >0 number of years	

	would be consideredliterate[SS2] ? List (Mozambique): illiterate, grade 5, grade 7, grade 10,grade 12, Bachelor, graduate, post-graduate, not-applicable, other, don't knowList (India): No formal schooling, literate; no formal schooling, illiterate;DK in both versions of MNH forms (although 2011 is DK/NA). In 2011 version,"schooling" is described as: primary incomplete; secondary incomplete;university/college incomplete; primary complete; secondary complete;'university/college complete. In 2013 version, schooling à yrs of schooling. [we need to separate literacy from education]<br>o **Distance that women live from the PHC** (close vs. far) - PHC Distance to the PHC may have to be ascertained using GIS mapping and analysis. This would limit this analysis to the time period of Definitive Trial and would be possible in two countries: Pakistan and Mozambique<br>• Community<br>o **Community engagement**<br>▪ **High vs. low** (defined as N meetings, N attendees/meeting, N topics covered)<br>o **Community health workers**<br>▪ Baseline **level of education** (defined as ##)<br>▪ Intensity of initial training<br>▪ Attendance at updates<br>o **CLIP visits**<br>▪ **Where the visits were conducted** (PHC vs. in the home)<br>▪ **Intensity of CLIP visits**, measure of 'compliance' with the protocol, defined as women who had prescribed N antenatal visits based on gestational age at enrollment and delivery (vs. those who did not), and women who had prescribed N postnatal visits (vs. those who did not) - we won't be able to do this because we know that compliance with prescribed visits was very low, so we should look at quartiles of N visits/N weeks between enrollment and delivery.<br>▪ **Clinical compliance** with CLIP recommendations for referral<br>▪ Women who went to facility vs. those who did not<br>▪ Women who were referred back to the community vs. those who were not<br>▪ **Transport** (by ambulance, private car/taxi, other transport, walking)<br>• Health care system<br>o **Density of community health care provider per population** – defined as the N population covered by each cHCP accounting for the new cHCP hired for CLIP

	o **Quality of care at PHC** - N health care providers at PHC-level<br>o **Quality of care at referral facilities** - N attendees (total) at CPD events at referral facilities (total), N (%)<br>• Time (later vs. earlier)<br>o ***Definitive vs. pilot trials** (which would exclude Mozambique)<br>o **Quartiles of study period**

Original version (Version 0.5-20160126) to final version (Version 0.6-20160126)

**Subheading, page (in final SAP)**	**Change(s)**	**Original version (with change highlighted)**
**2.3.2.2 Outcome data extraction**<br><br>**Pg. 13**	Deleted:<br><br>"If there is information that relates to the primary outcome from more than one surveillance form, the following rules will be used to extract the final value for analysis"<br><br>" For variables that record a numeric value, the analysed value will be the last value entered"<br><br>"For variables that record a frequency value, the analysed value will be the summation of all value entered"	
**2.7 Extraction of per-pregnancy data**<br><br>**Pg. 21 -22**	Trial participants may provide data through multiple surveys. To analyze data on a per-pregnancy level, the following rules will be used to extract the data per pregnancy for analysis:<br><br>• For variables that record the presence ("Yes") or absence ("No") of a condition, the analysed value will be "Yes" if this was answered in any of the forms, "No" if this and/or "unknown" was the only answer in any of the forms, and lastly "unknown" if this was the only answer in any of the forms<br>▪ e.g. Dialysis (Yes/No/Unknown)<br><br>• For variables that record a choice out of a list of equally weighted options, and there can only be one answer per woman, the analysed value will be the last value entered	

	▪ e.g. Delivery location (Home/Facility/On route/Other)<br><br>• For variables that record a choice out of a list of equally weighted options, and there can multiple answers per woman, each survey response will be analysed.<br>▪ e.g. Type of facility visited during the most recent admission<br><br>• For variables that record a numeric value, and there can only be one answer per woman, the analysed value will be the last value entered<br>▪ e.g. Age<br><br>• For variables that record a frequency value, that is recorded at each survey, the analysed value will be the summation of all values entered<br>▪ e.g. Number of visits to hospital

Version 0.6 20160126 (1) to Version 0.6 20160223 (2)

**Subheading, page (in revised SAP)**	**Change(s)**	**Original version (with change highlighted)**
**Section 2.3.5.1, A., p. 16**	Table number and caption were added to A. Community Engagement table.	**Table 1** Result table template for application of community engagement activities
**Section 2.5, p. 21**	Reference to 'data', changed to 'pregnancies'; 'has been received' changed to 'are expected'; 'planned' changed to 'projected'.<br><br>Addition of text on projected sample size and recruitment rates.	An interim analysis is planned for each of the three countries once complete pregnancies (until 42 days postpartum) are expected for women making up half of the projected sample size for that country.<br><br>Projection of sample size for each country will be based on the observed recruitment rates in the Definitive trial as follows: 12 months of recruitment in Pakistan, 15 months of recruitment in India, and 11 months of

		recruitment in Mozambique. The average monthly recruitment will be calculated and applied to the remaining months of the trial, to obtain the final projected sample size.
**Section 2.6.1, p.22**	Table number changed from 10 to 11.	**Table 2** Data fields used in the analysis of adverse events
**Section 2.8, p.23**	Reference to sample size calculation in the Appendix added.	For sample size calculation, see Appendix section 4.2.
**Section 4.1 – 4.2, p.25**	Section 4.1 CLIP country trials Sample Size Calculations added to Appendix.<br><br>Section 4.2 CLIP IPD Meta-analysis Sample Size Calculations	Due to length, please refer to p. 25 for CLIP country trials Sample Size Calculations table.<br><br>Assumptions:<br>• 10% loss of individual women to follow-up<br>• No loss of clusters to follow-up<br>• Alpha of 0.05, power ≥0.80<br>• Anticipated effect size of a 20% reduction in all cause maternal and perinatal/neonatal morbidity and mortality<br>• Also, we have assumed a ratio of maternal morbidity : mortality of 5:1 (may be as high as 10:1), and a ratio of perinatal/neonatal : maternal events of 5:1 (may be as high as 10:1 – permits overlap in outcomes)<br>• All sample sizes will be supplemented by the data collected in each single country Pilot CLIP Trial.<br><br>**4.2 CLIP IPD Meta-analysis Sample Size Calculations**<br><br>The combined Definitive CLIP Trials cohort of 60,600 deliveries in 44 clusters over 2 years, averaging 1377 deliveries per cluster per year, will provide the following power for the IPD meta-analysis:

		Maternal & perinatal/neonatal mortality & morbidity: assuming a 20% effect size (incidence reduced from 10.2% to 8.2%), we would have 80% power with an ICC of 0.006.<br><br>Maternal mortality & morbidity (primary outcome for the IPD meta-analysis): assuming a 20% effect size (incidence reduced from 1.7% to 1.4%), we would have 60% power with an ICC of 0.001. To achieve 80% power will require an ICC of <0.001 which is unlikely.<br><br>Alternatively, assuming a 30% effect size (incidence reduced from 1.7% to 1.2%), we would have 80% power with an ICC of 0.002. We hope to reduce the adverse maternal event rate by ≥30%, primarily through community mobilisation and antenatal care by cHCPs.
**Section 4.3, p.26**	Sub-header 4.1 Health Economics changed to 4.3 Health Economics.	**4.3 Health Economics – Plan of analysis**

Version 0.6 20160223 (2) to Version 0.6 20160307 – version to DSMB (3)

Subheading, page (in revised SAP)	Change(s)	Original version (with change highlighted)
**Section 2.5, p.20**	The word 'countries' was deleted, and replaced with 'sites'.<br><br>A statement around planned sample size and interim analysis was added.	An interim analysis is planned for each of the three sites once complete data (until 42 days postpartum) has been received for women making up half of the planned sample size for that country. In the event that the site is unable to reach planned sample size, due to logistical and pragmatic challenges, then the interim analysis will be conducted once complete pregnancies (until 42 days postpartum) are expected for women making

		up half of the projected sample size for that country.

Version 0.6 20160307 – Version to DSMB (3) to Version 0.7 20160824

**Subheading, page (in revised SAP)**	**Change(s)**	**Original version (with change highlighted)**
**Section 2.2, p.7**	Under Individual level variables, the word 'facility' was removed and 'PHC' inserted.	o Demographics: maternal age, parity, gestational age at enrolment, method of gestational age determination, maternal past medical history of hypertension or seizures/epilepsy, past obstetric history of hypertension, distance from PHC, level of education
**Section 2.3.2, p.9**	A new sentence was added to the Definitive Trial section (para. 3).	A hierarchical logistic regression model will be used to take into account the clustering of women within the units of randomisation. The model will adjust for key determinants of outcome, to be pre-specified by the investigators, at both the individual and cluster level. Adjustment variables at the individual level that are currently under consideration are: maternal age, parity, gestational age at enrolment, maternal past medical history of hypertension or seizures/epilepsy, past obstetric history of hypertension, distance from facility, level of education. Adjustment variables at the cluster level that are currently under consideration are: population density and baseline outcome rate. In addition, we will consider adjusting for any other characteristics that differ between groups at the individual and cluster level.
**Section 2.3.4, p.13**	Section 2.3.4 was moved down to Section 2.4.2 Other Outcomes, pp.18-20.	Due to the amount of text moved, please refer to pages 13 and 18-20 directly in each document.

Version 0.7 20160824 to Version 0.8 20160829

Subheading, page (in revised SAP)	Change(s)	Original version (with change highlighted)
**Table of contents**	Table of contents removed.	
**Section 2.3.2, p.10**	Text inserted into para. 3.	Adjustment variables at the individual level that are currently under consideration are: maternal age, parity, gestational age at enrolment, maternal past medical history of hypertension or seizures/epilepsy (not being considered currently), past obstetric history of hypertension (not being considered currently because not all women have past obstetric history), distance from facility (facility was defined as PHC, and we may not be able to ascertain this accurately in India and Pakistan, barring additional GIS work), level of education.
**Section 2.3.2.2, p.14**	Section 2.3.2.2, 'Inclusion of cases for primary outcome analysis' added.	Pregnancies included in the analysis of primary outcome are: pregnancies with at least one completed postpartum surveillance form, or, pregnancies still on follow-up with at least one completed antepartum surveillance form.
**Section 2.3.2.3, p.14**	Section 2.3.2.3, 'Calculation of the primary outcome' section added.	**2.3.2.3 Calculation of the primary outcome**<br>The composite primary outcome is calculated from the components using the following rules:<br><br><u>Primary outcome occurred</u>: If ANY of the individual components were answered 'yes' (even if some components were missing answers).

		Define which components were missing, and the N (%) women who had one,<br><br>two, three, etc, of the components missing.<br><br>Primary outcome did not occur: If none of the components were answered 'yes', and, ANY of the components was answered 'no' (even if some components were missing answers).<br><br>Define which components were missing, and the N (%) women who had one,<br><br>two, three, etc, of the components missing.<br><br>Primary outcome is missing: If NONE of the components was completed as 'yes' or 'no' (only 'unknown' and missing were recorded).<br><br>Denominator for the primary outcome rate will be all women included in the analysis (primary outcome occurred, primary outcome did not occur, and primary outcome missing).<br><br>These rules will also be used for the calculation of all other composite variables.
**Section 2.3.4, p.15**	Section number 2.3.5 'Per-protocol analyses' changed to 2.3.4.	Section 2.3.4 Per-protocol analyses
**Section 2.3.4.1, p.15**	Section number 2.3.5.1 'Application of the Intervention and challenges' changed to 2.3.4.1.	2.3.4.1 Application of the Intervention and challenges
**Section 2.3.4.2, p.16**	Section number 2.3.5.2 'Analyses' changed to 2.3.4.2.	2.3.4.2 Analyses

Version 0.8 20160829 to Version 1.2 20161115

Please note: Versions 0.9, 1.0, and 1.1, and 1.4 are not located in our files. The versions (0.9, 1.0, and

1.1) are thought to be identical versions of the same file. The version 1.4 is thought to have been accidentally skipped when numbering document versions.

Subheading, page (in revised SAP)	Change(s)	Original version (with change highlighted)
**Table of contents, p.3**	List of tables 'Appended in the 'SAP Tables' document are the following' updated to remove Table 8 'Result table template for application of community engagement activities'.	Table 1 Design of the Pilot Trial<br>Table 2 Design of the Definitive Trial<br>Table 3 Data fields used in the analysis of baseline and demographic variables<br>Table 4 Baseline outcome variables collected, by country<br>Table 5Decision rule for POM acceptance of referral<br>Table 6 Data fields used in the analysis of POM acceptance of referral<br>Table 7 Data fields used in the analysis of primary outcome<br>Table 8 Data fields used in the analysis of secondary outcomes<br>Table 9 Data fields used in the analysis of other outcomes<br>Table 10 Data fields used in the analysis of adverse events
**Section 1.1, p.4**	Section sub-header 1.1 'Study Description' added.<br><br>'Fundraising' replaced with 'microfinancing' in para. 1.<br><br>The word 'and' removed and 'measure' added to para. 2.	1.1 Study Description<br><br>**Community engagement** including community leaders, the women of the communities themselves, and their mothers, husbands, and mothers-in-law, regarding pre-eclampsia, its origins, symptoms, signs, and potential consequences, pre-permissions for maternal transport, and microfinancing activities around transport and treatment costs;<br><br>They will be trained to enquire about women's symptoms (using country-specific

		pictograms), take women's blood pressure (BP) (using systolic blood pressure (sBP), check urine for protein using dipstick and measure SpO2 (in Pakistan and Mozambique) on the first visit or on any subsequent visits for sBP ⩾140 mmHg. This will inform the diagnosis and risk assessment of women with pre-eclampsia.
	Para. 4 revised to include statement on quality care	This activity is designed to enhance skills and promote evidence-based pre-eclampsia identification and management so that women sent to facility receive quality care.
	Revisions and additions made to para. 5 to distinguish between pilot and definitive trial.	The trial was designed to occur in two phases - a Pilot phase and a Definitive phase. The Pilot phase was designed to show that the intervention was feasible, and the data for the pilot phase form part of the definitive phase analysis. As shown in Tables 1 and 2, the Pilot trial involved four clusters per country, and the number of clusters was expanded in the Definitive phase.
	Table 1 Design of the Pilot Trial, and Table 2 Design of the Definitive Trial removed from body of text.	
**Section 1.1.1, pp.4-5**	Section revised to reflect CLIP Nigeria updates.	To complement the effectiveness trials in Mozambique, India, and Pakistan, the process evaluation in Nigeria will serve to assure policy makers in Nigeria that the CLIP intervention can be undertaken successfully and is supported by both the community at large and health workers at various levels.<br><br>The process evaluation in Nigeria will involve data collected electronically on the POM mobile health device. Excluded from analysis will be data that were

		initially collected on paper and then entered electronically, as data entry was unreliable; these data involve the baseline household survey (feasibility study pre-pilot trial) and both pregnancy registration and trial surveillance (as part of the pilot and definitive trials).
**Section 1.2, p.6**	Primary Objectives sub-header section number 1.2.1 removed.<br><br>CEmOC abbreviation spelled out in text.<br><br>Secondary Objectives sub-header section number 1.2.2 removed.<br><br>Secondary Objectives section revised.	To implement the use of the CLIP 'package of care' and to observe at a minimum 50% acceptance of referral [urgent or non-urgent, to a facility able to provide CEmOC (Comprehensive Emergency Obstetric Care)] in women with a CLIP trigger.<br><br>Definitive Trial–<br>(1) To measure the impact of the CLIP intervention on birth preparedness and complication readiness, the proportion of women who present for care at a CEmOC, and the proportion of women who deliver in facility.
**Section 2.0, p.7**	Section number added to 'Data Summaries and Analyses' header.	2.0 Data Summaries and Analyses
**Section 2.1.1, p.7**	Section sub-header '2.1.1 Consort diagram' added.<br><br>Additions made to section content.	2.1.1. Consort diagram<br><br>▪ N women enrolled, as defined by consent to participate in trial surveillance.<br>▪ N women who withdrew<br>▪ N women who were lost to follow-up<br>▪ N women who were still on follow-up at the end of the trial

		▪ N women who were followed up postpartum<br><br>These categories will be defined as follows:<br><br>▪ **Withdrawal** (from trial surveillance) **-** captured from the withdrawal log on REDCap and Monthly Reports, identifying women who have withdrawn from data surveillance. This is further described in appendix 4.3.<br><br>▪ **Lost to follow-up** - antepartum women who had their last surveillance cycle more than one surveillance cycle from the end of the trial, or postpartum women who were 6 weeks postpartum more than one surveillance cycle from the end of the trial)<br><br>▪ **Still on follow-up**<br>o Antepartum women who had their last surveillance cycle within one surveillance cycle† from the end of the trial, **or**<br>o Postpartum women who were 6 weeks postpartum within one surveillance cycle from the end of the trial, **or**<br>o Women, either antepartum or postpartum, who were enrolled, and are within one surveillance cycle of the end of the trial (i.e. women who have no surveillance except pregnancy registration)<br><br>• **Followed-up** (i.e., have postpartum trial surveillance of any type)† *One surveillance cycle has been defined as 3 months (as in Pakistan), with 1 added to err on the side of caution.*
**Section 2.1.2, p.8**	Section number changed to 2.1.2.	**2.1.2. Baseline and Demographic Variables**<br><br>Summary statistics (mean, median, standard deviation, minimum, maximum and inter-

	Section revised with further details.	quartile range for continuous variables; the number and percentage of patients with various levels of categorical variables) will be calculated for each country, for:<br>• Each of intervention and control clusters; and<br>• Within each of intervention and control clusters, for women who were (i) lost to follow-up, (ii) still on follow-up at the end of the trial, and (iii) followed up. This analysis will enable us to see how women who were followed up may have differed from women who were not.<br><br>The planned **adjustment variables** are at both individual and cluster-levels, in addition to any other characteristics that differ between groups at the individual and cluster level, as follows:<br>***Individual level* (N=3): maternal age, parity, and level of education.**<br><br>The following were considered by the committee and rejected for use:<br><br>• 'Distance from facility' (defined as primary health centre, PHC) - Average distance would have to be modelled 'as the crow flies' (i.e., using straight lines from home to PHC) for India and Pakistan because there, more precise travel distances based on the actual road network cannot be calculated because of the lack of accessibility of street datasets.<br><br>• Gestational age at enrolment - Women in intervention clusters were exposed to the intervention (community engagement) and may have booked earlier for antenatal care. By adjusting for gestational age at enrollment, we may adjust for treatment effect. It is also assumed that if women (in intervention clusters) enroll in the trial earlier, we may be able to capture greater number of outcomes such as miscarriages.<br><br>o• Maternal past medical history of hypertension or seizures/epilepsy, - not being

		considered currently because not all women have past obstetric history<br><br>Cluster level (N=2): population density and the 'baseline' neonatal mortality rate from the baseline household survey.<br><br>The following were considered by rejected for use:<br><br>• The poverty index as a measure of SES is no longer being considered, as BPL card in India is not considered a reliable measure of SES.<br><br>• cHCP density is no longer being considered.
**Section 2.2, pp.8-9**	Section 2.2. Application of the intervention added.<br><br>Table 3. Data fields used in the analysis of baseline and demographic variables moved to appendix.	**Application of the intervention and challenges**<br><br>This analysis is justifiable because of challenges with application of the intervention. The following criteria may be used to define differing degrees of compliance:<br><br>A. Community Engagement – There is no clear acceptable value for the measures of community engagement. As such, we will use the following measures to rate the degree of community engagement, according to 'higher' community engagement (i.e., median or higher event rate or value) or 'lower' community engagement (i.e., below median event rate or value) in intervention clusters:<br><br>a. N (%) meetings held<br><br>b. N (%) meetings at which each key individual topic was covered<br><br>c. N attendees (i.e., large/small meetings)<br><br>d. N meetings that included male decision-makers

		e. N meetings that included community leaders (male or female)<br><br>B. **CLIP Visits and POM-guided management**<br><br>Analyses will be done according to:<br><br>• N (%) of women who received a CLIP POM visit(s)<br>• N (%) women (of all those in the clusters who received CLIP POM visits at least once every 4 weeks and once postpartum, as specified in the study protocol<br>• N (%) women who accepted (as defined in Section 2.2.1) the POM-guided transport/treatment recommendation compared with those who were not, among women who were given a recommendation<br><br>Referral acceptance was determined based on confirmation at a follow-up visit that the woman received treatment and/or went to facility. The concordance between these initial and follow-up compliance responses will be used to impute compliance for women for whom there was no follow-up visit . Decision rule for POM acceptance of referral is shown in Table 5, data fields used in the analysis of POM acceptance of referral are shown in table 6.<br><br>Withdrawal from surveillance is described in Appendix 4.3.
**Section 2.3, p.9**	Section '2.3 Primary analysis' content added around statistical tests.	All statistical tests will be two-sided, with significance levels of < 0.05 for the primary outcome, and <0.01 for secondary and other outcomes. Comparisons will be by means of risk ratios and mean differences with 95% confidence intervals for each site.
**Section 2.3.1, p.9**	Section revised and new additions added to text.	Referral acceptance was determined based on confirmation at a follow-up visit that the

	Table 4. Decision rule for POM acceptance of referral, and Table 5. Data fields used in the analysis of POM acceptance of referral moved to appendix.	woman received treatment and/or went to facility. The concordance between these initial and follow-up compliance responses will be used to impute compliance for women for whom there was no follow-up visit. A minimum of 50% acceptance of women with a CLIP trigger was used to assess the transition of the study site from the Pilot to Definitive Trial.
**Section 2.3.2, p.9**	Section revised and Table 6. Baseline outcome variables collected, by country moved to the Appendix.	All analyses will be based on intention-to-treat principles. The primary outcome is the proportion of pregnancies resulting in at least one of maternal death, stillbirth, neonatal death, or severe morbidity in the mother or child.<br><br>Multiple imputation by chained equations (MICE) will be used to fill missing values, for both explanatory and outcome variables, before regression modeling of outcomes. This assumes that missing values are missing completely at random, that is, there are no systematic differences between the missing values and the observed values. Imputation will be repeated at least ten times, and outcome rates will be modeled in each of the derived dataset.<br><br>A hierarchical logistic regression model will be used to take into account the clustering of women within the units of randomisation. The model will adjust for key determinants of outcome, pre-specified by the investigators, at both the individual and cluster level.
**Section 2.3.2.1, p.10**	'Postpartum' added to description of the primary outcome.	**Maternal death** (defined as the number of deaths during pregnancy or within 42 days of delivery (or the last postpartum contact day if contact not maintained to 42 days postpartum) / 1,000 identified pregnancies)<br><br>**Maternal morbidity** (defined as the number of women with one or more life-threatening complications of pregnancy during pregnancy or within 42 days of delivery or last

	Table 7. Data fields used in the analysis of primary outcome, moved to the appendix.	postpartum contact day if contact not maintained to 42 days) / 1,000 identified pregnancies).
**Section 2.3.2.2, p.11**	Revisions and additions made to text.	All enrolled pregnancies, with the exception of those who withdrew from the trial, will be included in the analysis of primary outcome. For women who were 'lost to follow up' or 'still on follow up' at the end of the trial, the primary composite outcome data will be imputed using a mixed imputation based that takes into account the risk associated with each woman depending on her personal characteristics (from pregnancy registration), cluster characteristics, and time of enrollment relative to the beginning of the trial.
**Section 2.3.2.3, pp.11-12**	Section revised to include further details.	The data fields used in the analysis of primary outcome composite is table 7, and primary outcome is calculated from the components using the following rules:<br><br>Primary outcome occurred: If ANY of the individual components were answered 'yes' (even if some components were missing answers). Define which components were missing, and the N (%) women who had one, two, three, etc, of the components missing.<br><br>Primary outcome did not occur: If none of the components were answered 'yes', and, ANY of the components was answered 'no' (even if some components were missing answers). Define which components were missing, and the N (%) women who had one, two, three, etc, of the components missing. Our assumptions can be checked by comparing patterns of responses among women who had complete and incomplete response sets.

		Primary outcome is missing: If NONE of the components was completed as 'yes' or 'no' (only 'unknown' and missing were recorded). This women will be included in the imputation.<br><br>Denominator for the primary outcome rate will be all women included in the analysis (primary outcome occurred, primary outcome did not occur, and primary outcome missing).<br><br>These rules will also be used for the calculation of all other composite variables. Section 2.3.3 shows primary outcome and its components.
**Section 2.3.3.1, p.12**	Section added and includes further details on postpartum follow-up.<br><br>Table 8. Result table template for application of community engagement activities removed from text.	**2.3.3.1 Postpartum follow-up:** *This will assess the impact of imputation.* We will repeat the comparison between intervention and control groups using only cases with postpartum follow-up.
**Section 2.3.3.2, p.12**	Section added.	***2.3.3.2 Complete postpartum follow-up (for primary outcome):*** *This will assess the impact of our assumptions about missing components of the primary outcome*. Pregnancies for whom we have complete outcome data (i.e., all questions were answered for the primary outcome). If this is significant, then we will consider repeating 2.2.3.1 with the imputation based only for women with 42 day postpartum follow-up.
**Section 2.3.3.3, p.12**	Section added.	***2.3.3.3 Women whose EDD+3 weeks falls within the trial timeline***: *This will assess the impact of the intervention independent of gestational age at birth*. This includes women for whom the primary outcome was imputed.
**Section 2.3.3.4, p.12**	Section added.	***2.3.3.4 Women whose EDD+3 weeks plus 42 day follow up period falls within the***

		trial timeline: *This will assess the impact of incomplete postpartum follow-up that was <42 days postpartum*. This will involve all women for whom the primary outcome was imputed.
**Section 2.3.3.5, p.12**	Section added.	***2.3.3.5 According to penetration of the intervention:*** Penetration will be defined as the: (i) intensity of community engagement (N sessions/N women enrolled in that that cluster as a continuous variable) andii) presence of an active community health worker within a community (compared between intervention and control clusters). Depending on the distribution of the N women reached and the N visits that they receive, we may be able to define penetration in terms of the intensity of CLIP visits in terms of the N women reached (N women with one or more CLIP visits/N women enrolled in that cluster as a continuous variable), villages reached by one/more CLIP visit, and the intensity of the visits (N visits/N women enrolled in that cluster as a continuous variable). We will be unable to look at the third aspect of the intervention (i.e., effect of compliance with treatment recommendations) given small numbers.
**Section 2.4.1, pp.12-13**	Section revised to include further details around secondary outcomes.	The secondary outcomes have been defined to measure the impact of the CLIP intervention on the delays around triage and transport.<br><br>**(1) Birth preparedness and complication readiness**, will assess the success of community engagement, and be measured by any *three* of the following, measured antenatally as close to delivery as available*:<br><br>(1) arranged for transport;<br><br>(2) obtained prior permission for transport should emergency arise;

	Table 8 moved to the appendix, and descriptive sentence of Table 8 revised.	(3) saved money for obstetric care;<br><br>(4) identified skilled birth attendant;<br><br>(5) identified facility for delivery.<br><br>** In India, this was at pregnancy registration, but in Pakistan and Mozambique, it could have been at pregnancy registration or antenatal surveillance. Any measurements taken in Pakistan postpartum will be disregarded.*<br><br>**(2) Proportion of women presenting for care at a CEmOC facility and proportion of facility births: (This number should increase if triage (using the miniPIERS risk stratification and POM) is effective, and if there is compliance with transport to CEmOC facility.)**<br><br>Table 8 shows the data fields used in the analysis of secondary outcomes.
**Section 2.4.2, p.13**	Section 2.4.2 Other Outcomes, revised to include further details.	o Individual components of the primary outcome, including N seizures (overall and either pre-CLIP visit or post-CLIP visit , pre- or post- MgSO4 administration where applicable), maternal death or morbidity timing relative to administration of antihypertensive therapy for severe hypertension<br>o Adverse events (see 2.5.1 below)<br>o Knowledge of pre-eclampsia/eclampsia<br>o Total number of: antenatal care sought, N blood pressure measurements performed by health professionals, antihypertensive therapy received<br>o Gestational age at delivery<br>o Functional disability, which is defined as the inability to perform usual daily duties at specific points in time during the postpartum period. These are defined to reflect what would be acceptable and expected culturally in each country. The normative ranges will be defined by data from the control clusters (We hypothesise that a

	Table 10. Data fields used in the analysis of other outcomes moved to appendix.	threshold of 90th centile would be reflect return to functional ability in each of the sites, however this would be determined from the data)
**Section 2.4.3, pp.13-14**	Section 2.4.3 'Other Planned secondary analysis' sub-header added; reference to appendix section number amended/ added; much of the text moved to the appendix.<br><br>Revisions to text.<br><br>Paragraph of options for responses to 'level of education questions revised' slightly.<br><br>Bullet sub-points under 'Women' revised.	**2.4.3 Other Planned secondary analysis**<br>o Cost-effectiveness of the CLIP intervention (See Appendix 4.5 for details)<br>o Geo-temporal analysis (MOMI) (See Appendix 4.4 for details)<br><br>We will analyse the following subgroups by country and within each of the three countries, by cluster:<br>In 2013 version, schooling is described as yrs of schooling.<br><br>o Gestational age at delivery
**Section 2.6.1, p.16**	Formerly Table 11. Data fields used in the analysis of adverse events, moved to appendix.	
**Section 3.1, p.17**	Section revised with respect to significance levels.	Where needed, statistical tests will be two-sided, with significance levels of 0.05 for primary outcome, and 0.001 for secondary and other outcomes, unless stated otherwise.
**Section 4.1, pp.18-19**	Section number updated from 5.1 to 4.1.<br><br>Number of health facilities updated, PHC/RHC updated, EmOC updated, and Number of	**4.1 CLIP country trials Sample Size Calculations**<br><br>**Number of health facilities (total)** 56<br><br>**PHC/RHC** 10 BEmoc<br><br>**EmOC** 1 CEmoc

	cHCPs (total) updated for Mozambique.	**Number of cHCPs (total)** 59(early 2013); 114 (late 2013)
**Section 4.2, p.19**	Section number updated from 4.13 to 4.2.	4.2 CLIP IPD Metal-analysis Sample Size Calculations
**Section 4.3, p.20**	Section added.	**4.3 Withdrawal**<br>o **Mozambique:** a woman who has received a Form D (pregnancy registration), who rejects further trial surveillance (Form E). In intervention clusters, there is then consent for POM and the package of care in response to a CLIP trigger, should one develop.<br>o **India:** Withdrawal is defined as a woman with a MN01 who declines having MN02. In intervention clusters, there is then consent for POM and the package of care in response to a CLIP trigger, should one develop.<br>o **Pakistan**: Withdrawal is defined as a woman with pregnancy registration who declines to have trial surveillance (antepartum/postpartum). In intervention clusters, there is then consent for POM, and then should a CLIP trigger occur, consent for the package of care
**Section 4.4, p.20**	Section 4.4 added, and text moved down from above.	**4.4 The Geo-Temporal analysis** will serve to; 1) elucidate the spatial variation in community level risk for adverse maternal outcomes, 2) Identify the factors that promote maternal resilience in the different communities under study, and 3) illustrate changing rates of adverse outcomes through the timeline of the CLIP trials. The population level estimates of variables of interest will be calculated using data from the surveillance cycles, while community rates of adverse outcomes will be calculated by combining the maternal deaths and severe morbidities recorded through facility surveillance. Geographically Weighted Regression (GWR) will be used showcase the spatial variation in associations between the choice variables, and rates of adverse maternal outcomes.

		Communities with less than expected rates of adverse outcomes, based on their community characteristics, will be further assessed using GWR against the predetermined community resilience variables to identify the factors that are associated with maternal resilience. Key place specific factors promoting maternal resilience will we weighted and combined into an index measuring maternal resilience. Summaries of predetermined indicators of both primary and secondary outcomes of the trial will be calculated and mapped using the ArcGIS software on a quarterly basis for the entire timeline of the trial data. This will enable the creation of geo-temporal visualizations and data indicating the changing rates of outcomes throughout the timeline of the trial.
**Section 4.5, p.20**	Section number changed from 4.3 to 4.5 and text moved down from above.	**4.34.5 Health Economics – Plan of analysis**<br><br>Parameter estimates for costs and effectiveness will be derived from the CLIP surveillance questionnaire. The unit costs will be multiplied by identified health resource utilization to calculate the total cost per pregnancy, including both pregnant woman and newborns. The total cost will be calculated as the sum of the health resource utilization cost, cost of implementing the CLIP package of care, cost of routine pregnancy care and societal costs. The annual equivalent costs in local currency of selected CLIP countries will be converted to US dollar. The pregnancy outcomes (i.e. health of mother and baby) will be modelled as the effectiveness of the CLIP interventions. This will include no-adverse outcomes (healthy mother and newborn at the time of delivery) and adverse outcomes (death and/or disability of mother and baby) observed in the intervention and control groups. Using the data and parameter estimates specific to each CLIP country, the incremental cost-effectiveness ratios (ICER)s will be calculated

		first from a healthcare system perspective and then from a societal perspective. The country-specific ICER will be calculated as incremental cost per adverse pregnancy outcome. Comparison will be made between the country-specific ICER and the per capita value for the gross national income for the year 2015.<br><br>Given the uncertainties involved in CEA, we will use probabilistic sensitivity analysis to produce cost-effectiveness plots. The confidence region surrounding the cost-effectiveness ratio will be estimated using appropriate statistical methods, including bootstrap and Monte Carlo simulations. Life tables based on data from the World Health Organization's Southeast Asia and African regions or the West level-26 model will also be used in a sensitivity analysis. Country-specific health system budget impact analysis will be conducted to facilitate policy decisions for resource allocation.

Version 1.2 20161115 to Version 1.3 20161125

**Subheading, page (in revised SAP)**	**Change(s)**	**Original version (with change highlighted)**
**2.1.2, p.8**	Asterisk added to 'Individual level' variables for the inclusion of note, and note/text added.	***Individual level**** Although baseline neonatal mortality rates were not considered in the design of either the original pilot or definitive trials, these rates were found to vary substantially across clusters in the analysis of the pilot trial results. It was felt that their inclusion as an adjustment variable in the final analysis would substantially improve the power to detect a treatment effect. Without knowledge of the pilot trial results, the trial steering group elected to include based neonatal mortality

		from the pilot trial as an adjustment in the primary analysis.<br><br>* Adjustment for individual level variables is likely to have less impact on the results. However, we will also include these variables to maintain consistency with how cluster level adjustment variables are being handled.
**2.3.3.5, p.12**	Sub-header added to comment on sensitivity analyses without adjustment.	2.3.3.5 Without adjustment: Sensitivity analyses without adjustment at either the cluster or individual levels will be conducted.
**3.1, p.18**	Additional information added to 3.1 Statistical Tests section.	The population standardized risk difference will be presented as an alternate summary of the benefit of the intervention rather than as a primary analytic endpoint.

Version 1.3 20161125 to Version 1.5 20161130

**Subheading, page (in revised SAP)**	**Change(s)**	**Original version (with change highlighted)**
**1.1, p.6**	Further details added around surveillance data and trial protocol (final paragraph).	The trial was designed to occur in two phases - a Pilot phase and a Definitive phase. The Pilot phase was designed to show that the intervention was feasible, and the data for the pilot phase form part of the definitive phase analysis. As shown in Tables 1 and 2, the Pilot trial involved four clusters per country, and the number of clusters was expanded in the Definitive phase. Surveillance data from the Pilot Trial was not analysed or reviewed and no outcomes were reviewed until after the end of the Definitive Trial. The trial protocol has been registered on clinicaltrials.gov with trial identifier NCT01911494.
**1.2, p.8**	Specific details around countries added, as well as	• Pilot Trial – (for each of Nigeria, Pakistan and India)– To implement the use of the CLIP 'package of care' and to observe at a minimum 50% acceptance of referral [urgent

	further details around IPD meta-analysis.	or non-urgent, to a facility able to provide CEmOC (Comprehensive Emergency Obstetric Care)] in women with a CLIP trigger<br><br>• Definitive Trial (for each of Mozambique, Pakistan and India) – To reduce pre-eclampsia-related, and all-cause, maternal and perinatal mortality and major morbidity by 20% or more in intervention clusters compared with control clusters.<br><br>• IPD meta-analysis (combining results from Mozambique, Pakistan and India)-<br><br>o Maternal mortality & morbidity (primary outcome for the IPD meta-analysis): reduction by $\geqslant$ 20%<br><br>o Maternal and fetal/neonatal mortality & morbidity: reduction by $\geqslant$ 20%<br><br>o Maternal mortality: reduction by $\geqslant$ 30%<br><br>More details are in section 2.8 and power calculations for the IPD meta-analysis are in Appendix section 4.3
**Section 1.3, p.9**	Section 1.3 'Sample size calculations' added.	Each country is independently powered to assess the chosen primary outcome. The data upon which the estimates have been made are routinely at the conservative end of the published or available range, and generally provided by the site investigators. Sample size calculation for the primary outcome can be seen in Appendix 4.1. However, these calculations were revisited as planned originally, after data from the CLIP Feasibility Study and CLIP Pilot maternal and perinatal morbidity and mortality data were collected.<br><br>This review conducted independently by the Statistical Team provided insight that neonatal mortality rate in baseline varied greatly across

		clusters. At this point, it was also suggested by the Statistical team that adding more clusters would increase statistical power- however, this was not deemed feasible. A suggestion was then made by the statistical team to add neonatal mortality rate as a cluster level adjustment factor. More details on adjustment factors are in Section 2.1.2.
**Section 2.1.2, pp.10-11**	Details revised and further details added to expand upon adjustment of variables (p.10), individual level variables (p.11), and cluster level (p.11).	Adjustment will be undertaken to improve precision.<br><br>Individual level (N=3): maternal age(as a continuous variable), parity(nulliparous/multiparous), and maternal basic education (defined in Mozambique as attainment of Grade 5 or above, and in Pakistan and India as completion of 6 years or more of schooling. There is no formal definition of literacy that can be applied across all settings, however, primary schooling was defined as 6 years (including level 0 and early childhood education) in the UNESCO International Standard Classification of Education (ISCED) 2011).<br><br>***Cluster level* (N=2): population density** *(population of cluster/unit size of cluster)* ***and the 'baseline' neonatal mortality rate*** (from the baseline household survey.)<br><br>Although baseline neonatal mortality rates were not considered in the design of either the original pilot or definitive trials, these rates were found to vary substantially across clusters in the analysis of the data gathered in the Feasibility Study. It was felt that their inclusion as an adjustment variable in the final analysis would substantially improve the power to detect a treatment effect. Without knowledge of the pilot trial results, the CLIP trial steering group elected to include based

		neonatal mortality from the pilot trial as an adjustment in the primary analysis.
**Section 2.2, p.12**	Sub-header section number 2.3 removed from section 'Primary analysis', and further details added.	All analyses would be intention to treat. All statistical tests will be two-sided, with significance levels of < 0.05 for the primary outcome, and <0.01 for secondary and other outcomes.
**Section 2.2.1, pp.12-13**	Section number changed from 2.3 to 2.2, and further details added to 'Pilot Trials' section (para. 1 and 3).	At the end of the specified Pilot period per country (with the exception of Mozambique), the Piers On the Move data from intervention clusters was analysed for completed pregnancies to calculate referral acceptance. Surveillance data was not reviewed for intervention and control clusters except by statistical teams.<br><br>These process data were shared with the CLIP Steering Committee to assess the progression of country from the Pilot to Definitive Trial. No outcome data from either intervention and control arms would be reviewed until after the end of the Definitive Trial. The pregnancies included in the Pilot trial were carried over into the Definitive Trial and will be analysed for primary outcome at the end of the Definitive Trial.
**Section 2.2.2.1, p.13**	Section number changed from 2.3.2.1 to 2.2.2.1	2.2.2.1 Definition of the primary outcome
**Section 2.2.2.2, p.15**	Section sub-header revised from 'Inclusion of cases for the primary outcome' to 'Calculation of the primary outcome'.<br><br>'Consent' inserted into first sentence of section.	2.2.2.2 Calculation of the primary outcome<br><br>All enrolled pregnancies, with the exception of those who withdrew consent from the trial, will be included in the analysis of primary outcome.

**Section 2.2.2.3, p.15**	Section number changed from 2.3.2.3 to 2.2.2.3, and sub-header revised from 'Calculation of the primary outcome' to 'Analysis of the primary outcome'.	2.2.2.3 Analysis of the primary outcome
**Section 2.2.3, p.15**	Section number changed from 2.3 to 2.2.3	**2.2.3 Sensitivity analyses**
**Section 2.2.3.1, p.15**	Section number changed from 2.3.3.1 to 2.2.3.1; sub-header changed from 'Postpartum follow-up' to 'Completed pregnancies with postpartum follow-up'; details regarding imputation added.	**2.2.3.1 Completed pregnancies with postpartum follow-up**<br><br>This will involve no imputation.
**Section 2.2.3.2, p.16**	Section number changed from 2.3.2 to 2.2.3.2; section sub-header revised; details regarding imputation added.	**2.2.3.2 Complete postpartum follow-up (for all components of primary outcome)**<br>This will involve no imputation. However, if this is significant, then we will consider repeating 2.2.3.1 with the imputation based only for women with 42 day postpartum follow-up.
**Section 2.2.3.3, p.16**	Section number changed from 2.3.3.3 to 2.2.3.3.	**2.2.3.3 Women whose EDD+3 weeks falls within the trial timeline**
**Section 2.2.3.4, p.16**	Section number changed from 2.3.3.4 to 2.2.3.4.	**2.2.3.4 Women whose EDD+3 weeks plus 42 day follow up period falls within the trial timeline**
**Section 2.2.3.5, p.16**	Section number changed from 2.3.3.5 to 2.2.3.5; details added around adjustment and imputation. Sub-header and section 'According to penetration of the intervention' removed.	**2.2.3.5 Without adjustment:** *This will assess the impact of adjustment.* Sensitivity analyses without adjustment at either the cluster or individual levels will be conducted. This will involve all women for whom the primary outcome was imputed.
**Section 2.2.3.6, p.16**	Section 2.2.3.6 'For women in India' added.	**2.2.3.6 For women in India:** This will assess the impact of postpartum enrollment of women in the MNH Registry. This will involve

		all women enrolled in CLIP India Trial (receiving MN01) antepartum. In India, due to the nature of the Maternal and Newborn Health Registry, women may be enrolled in the CLIP Trial postpartum if they deliver in the facility within the cluster in either arm of the trial. Such women in the intervention arm may have been exposed to community engagement, however will not have CLIP antenatal visits guided by POM application.
**Section 2.2.3.7, p.16**	Section added.	**2.2.3.7 Evaluating 'on treatment' effect:** An evaluation of treatment effect will be undertaken comparing women with one or more CLIP visits in intervention arm to women in the control arm. Assessment of the effect of the penetration of the intervention in terms of intensity of community engagement and of the CLIP visits will take place in the process evaluation within the intervention clusters. We will be unable to look at the third aspect of the intervention (i.e., effect of compliance with treatment recommendations) given small numbers.
**Section 2.3, pp.16-17**	Section number changed from 2.4 to 2.3; section 'Application of the intervention' moved down; sentence in the last paragraph of section amended.	The concordance between these initial and follow-up compliance responses will be used to describe compliance for women for whom there was no follow-up visit.
**Section 2.4, p.17**	Section number added; section header changed from 'Analyses' to 'Secondary Analyses'; text amended regarding birth preparedness and complication readiness.	~~** In India, this was at pregnancy registration, but in Pakistan and Mozambique, it could have been at pregnancy registration or antenatal surveillance. Any measurements taken in Pakistan**~~*Any measurements taken postpartum will be disregarded.*
**Section 2.4.2, p.18**	Adverse events reference to section 2.5.1 changed to 2.6.1	o Adverse events (see 2.6.1 below)

**Section 2.4.3, pp.18-20**	Section revised and text removed.	o **Literacy** (literate vs. ~~illiterate) – In Pakistan and India, "literacy" is defined as "Ability to read and write in any language"~~illiterate)<br><br>o ~~Level of education for mother (none, some/completed primary, some secondary or more)~~<br><br>~~o Level of education for partner (none, some/completed primary, some secondary or more)~~<br><br>~~o Maternal Level of Education will be ascertained by: Years of school completed (for Pakistan) - >0 number of years would be considered literate~~<br>o ~~[List (Mozambique): illiterate, grade 5, grade 7, grade 10,grade 12, Bachelor, graduate, post-graduate, not-applicable, other, don't know. List (India): No formal schooling, literate; no formal schooling, illiterate;DK in both versions of MNH forms (although 2011 is DK/NA). In 2011 version,"schooling" is described as: primary incomplete; secondary incomplete;university/college incomplete; primary complete; secondary complete;'university/college complete. In 2013 version, schooling is described as yrs of schooling.~~
**Section 2.5, p.20**	Sentence added to paragraph to reference Data Safety and Monitoring Board.	The interim analysis is reviewed by the Data Safety and Monitoring Board (DSMB).
**Section 2.8, p.22**	Further details added to section.	~~An~~At the completion of all three trials (Mozambique, Pakistan and India), an individual patient data (IPD) meta-analysis will be conducted (at UBC~~) after the completion of all three trials.~~). For the IPD meta-analysis, we

		will analyse data for women recruited at ⩾20 weeks, to standardise data to the latest public declaration of pregnancy (i.e., 20 weeks in Pakistan). If baseline outcome data are available and consistent across all three countries, these data will be used for adjustment in analysis. For ~~sample size~~power calculation, see Appendix section 4.2.
**Section 4.1, p.25**	CLIP country trials sample size calculations edited under ‘Number of cHCPs (total)’ for Mozambique and India.	**Mozambique**<br>~~59(early 2013); 114 (late 2013)~~135 APEs (71 prior to study start)<br><br>**India**<br>~~60 ANMs & 360~~260 (215 ASHAs, 45 ANMs)
**Section 4.2, p.26**	Sub-header revised; section Revised to include further details.	4.2 CLIP IPD Meta-analysis ~~Sample Size~~Power Calculations<br><br>The combined Definitive CLIP Trials (Mozambique, Pakistan and India) cohort of 60,600 deliveries in 44 clusters over 2 years, averaging 1377 deliveries per cluster per year, will provide the following power for the IPD meta-analysis:<br><br>• CLIP Definitive Trials Primary Outcome (Maternal & ~~perinatal~~fetal/neonatal mortality & morbidity~~:~~): assuming a 20% effect size (incidence reduced from 10.2% to 8.2%), we would have 80% power with an ICC of 0.006.<br><br>• Maternal mortality & morbidity (primary outcome for the IPD meta-analysis): assuming a 20% effect size (incidence reduced from 1.7% to 1.4%), we would have 60% power with an ICC of 0.001. To achieve 80% power will require an ICC of <0.001 which is unlikely.<br><br>• ~~Alternatively,~~Maternal mortality: assuming a 30% effect size (incidence reduced from 1.7% to 1.2%), we would have 80% power with an ICC of 0.002. We hope to

		reduce the adverse maternal event rate by ⩾ 30%, primarily through community mobilisation and antenatal care by cHCPs.
**Section 4.4, p.26**	Phrase 'The Geo-Temporal analysis' added to initial sentence of section.	The Geo-Temporal analysis will serve to; 1) elucidate the spatial variation in community level risk for adverse maternal outcomes, 2) Identify the factors that promote maternal resilience in the different communities under study, and 3) illustrate changing rates of adverse outcomes through the timeline of the CLIP trials.
**Section 4.5, pp.27-28**	Section number added to header 'Health Economics – Plan of analysis'; words add to reference Figure 1 (p.28).	**4.5 Health Economics – Plan of analysis** Figure 1: (next page) Decision analytic tree model for economic evaluation of CLIP. HDP, hypertensive disorder of pregnancy

Original version (Version 1.5 20151130) to final version (Version 1.6 20160123)

**Subheading, page (in final SAP)**	**Change(s)**	**Original version (with change highlighted)**
**2.1.1 Consort diagram**<br><br>**Pg. 7**	Original version:<br><br>These categories will be defined as follows:<br><br>▪ **Lost to follow-up** - antepartum women who had their last surveillance cycle more than one surveillance cycle from the end of the trial, or postpartum women who were 6 weeks postpartum more than one surveillance cycle from the end of the trial)<br><br>▪ **Still on follow-up**<br><br>o Antepartum women who had their last surveillance cycle within one surveillance cycle†	Revised version:<br><br>These categories will be defined as follows:<br><br>• **Lost to follow-up** - women without trial surveillance, who were > 6 weeks postpartum more than one surveillance cycle from the end of the trial<br><br>• **Still on follow-up**<br><br>o Antepartum women, or<br><br>o Women, without postpartum trial surveillance, who were ⩽ 6 weeks postpartum within one

	from the end of the trial, **or**<br>o Postpartum women who were 6 weeks postpartum within one surveillance cycle from the end of the trial, **or**<br>o Women, either antepartum or postpartum, who were enrolled, and are within one surveillance cycle of the end of the trial (i.e. women who have no surveillance except pregnancy registration)	surveillance cycle┼ from the end of the trial, or
**2.1.2 Baseline and demographic variables**<br><br>**Pg. 7**	***Individual level* (N=3): maternal age***(as a continuous variable), **parity***(nulliparous/multiparous)***, and *maternal basic education (****defined in Mozambique as attainment of Grade 5 or above, and in Pakistan and India as completion of 6 years or more of schooling. There is no formal definition of literacy that can be applied across all settings, however, primary schooling was defined as 6 years (including level 0 and early childhood education) in the UNESCO International Standard Classification of Education (ISCED) 2011).*	***Individual level (N=3): maternal age****(as a continuous variable),* ***parity****(nulliparous/multiparous), and and* ***maternal basic education*** *(defined in Mozambique as attainment of Grade 5 or above, and in Pakistan as completion of 5 years or more of schooling and in India as 8 years or more of schooling. There is no formal definition of literacy that can be applied across all settings, however, primary schooling was defined based on the country specific information provided by the site investigators).*

Original version (Version 1.6 20160123) to final version (Version 1.7-20170301)

Subheading, page (in final SAP)	Change(s)	Original version (with change highlighted)
**2.2.2 2 Calculation of the primary outcome**	Added:	

<table>
<tr><td>Pg. 11</td><td colspan="2">All enrolled pregnancies, with the exception of those who withdrew consent from the trial and those enrolled postpartum without antenatal POM visits, will be included in the analysis</td></tr>
<tr><td>2.2.3.6

Pg. 12</td><td>Original:

For women in India: This will assess the impact of postpartum enrollment of women in the MNH Registry. This will involve all women enrolled in CLIP India Trial (receiving MN01) antepartum.</td><td>Revised:

Women enrolled postpartum This will assess the impact of postpartum enrollment of women in the MNH Registry (India) and six monthly surveillance cycles (Mozambique). For the India Trial analysis, this will involve all women enrolled in CLIP India Trial (receiving MN01) postpartum.</td></tr>
<tr><td></td><td colspan="2">Added:

In Mozambique, due to various factors including six monthly surveillance cycle, cultural barriers around disclosing pregnancy and geographical barriers, women who have consented to CLIP surveillance antepartum may receive enrollment surveillance when they have already delivered. Some of these women may receive Piers On the Move guided antenatal visits. This sensitivity analysis will involve all women enrolled postpartum with no POM guided antenatal visits.</td></tr>
</table>

Blood pressure thresholds in pregnancy for identifying maternal and infant risk: a secondary analysis of Community-Level Interventions for Pre-eclampsia (CLIP) trial data - Appendix

CLIP Trial Data Sharing Statement

CLIP Trials Data Sharing Statement

The CLIP Trial data are de-identified participant-level data. Once the primary CLIP manuscripts, individual participant data meta-analysis, and papers based on the other pre-defined analyses are published as per the Statistical Analysis Plan (SAP), the data will be freely available to academically-active entities (e.g., universities, NGOs, multilaterals), with the CLIP Principal Investigator (Peter von Dadelszen) or named delegate as a named co-investigator, for the purposes of pregnancy-related research and within the limits of the informed consent obtained. Access will be through the CLIP Trials Data Access Committee*, contacted at 'PRE-EMPT@cw.bc.ca', as referenced on our website at 'https://PRE-EMPT.bcchr.ca'. A full data dictionary and all study documents will be available. Access will be through written application. When approved, a quote for the costs of preparing the data will be provided to the applicant.

By submitting an application form, the investigator agrees that s/he has read, understood and agrees to the terms and conditions below:

1. S/he is an academically-active researcher affiliated with an entity able to engage in a data transfer agreement;
2. S/he warrants that the information entered is to the best of her/his knowledge full and correct;
3. S/he agrees that the Data Sharing Agreement will only be used for the specific project outlined in the application;
4. S/he represents that s/he has obtained the necessary approvals to transfer the data and/or receive the data under this Data Sharing Agreement;
5. S/he understands that the responses provided will form part of a legally-binding document;
6. S/he understands that the Agreement is not valid until a fully-executed copy, with signatures from all parties, is emailed to PRE-EMPT@cw.bc.ca); and
7. S/he understands that no modifications can be made to the Data Sharing Agreement and if modifications are made, the Data Sharing Agreement will be rendered invalid.

There is no pregnancy-specific repository for us to access, but once the primary papers for the CLIP Trials have been published, we will be depositing a copy of our data in the HBGDki repository at the Bill & Melinda Gates Foundation, our funder. The permitted uses and disclosures of these data are as follows:

1. The Foundation will limit the use and disclosure of the CLIP data to conduct research related to achieving the goals of the Foundation as represented above. The Foundation may also de-identify the data set and aggregate it with other de-identified information.
2. The Foundation will restrict access to the CLIP data to individuals involved in the Foundation's research who have a need to access the CLIP data to carry out their duties as they relate to the Permitted Uses and Disclosures identified above, and any such access will be consistent with the assurances and obligations set forth in this Agreement. The Foundation will use appropriate safeguards to prevent use or disclosure of the CLIP data other than as permitted by this Agreement.
3. The Foundation will report to HBGDki Collaborator any use or disclosure of the CLIP data not provided for by this Agreement of which the Foundation becomes aware.

4. The Foundation will ensure that any agents, including subcontractors, to whom it provides the CLIP data, if any, agree to the same restrictions and conditions that apply to the Foundation with respect to such information.

** The **Data Access Committee** is made up of the following individuals: Peter von Dadelszen and Laura A. Magee (King's College London, UK); Zulfiqar A Bhutta (Aga Khan University, Karachi, Pakistan and the Hospital for Sick Children, Toronto, Canada); Rahat N Qureshi (Aga Khan University, Karachi, Pakistan); Ashalata A Mallapur (S Nijalingappa Medical College, Bagalkote, India); Mrutyunjaya B Bellad and Shivaprasad Goudar (KLE Academy of Higher Education and Research's JN Medical College, Belagavi, India); Khátia Munguambe, Charfudin Sacoor, and Esperança Sevene (Centro de Investigação em Saúde da Manhiça, Manhiça, Mozambique)*
